# Polynuclear Superhalogen Anions with Heterovalent Central Atoms

**DOI:** 10.3390/molecules31060933

**Published:** 2026-03-11

**Authors:** David Mekhael, Piotr Skurski, Iwona Anusiewicz

**Affiliations:** 1 Laboratory of Quantum Chemistry, Faculty of Chemistry, University of Gdańsk, Wita Stwosza 63, 80-308 Gdansk, Poland; d.mekhael.931@studms.ug.edu.pl (D.M.); piotr.skurski@ug.edu.pl (P.S.); 2 Department of Chemistry, University of Utah, Salt Lake City, UT 84112, USA

**Keywords:** molecular anions, polynuclear heterovalent superhalogens, ab initio calculations

## Abstract

This study explores a novel class of polynuclear superhalogen anions featuring heterovalent central atoms from groups 13 (B, Al) and 15 (P, As). The investigated species follow a modified general formula, (**X***_n_***Y***_n__’_*F_{(3*n*+5*n*_*_’_*_)+1}_)^−^ where **X** = B and/or Al, **Y** = P and/or As, and *n* + *n′* = 2–4. Low-energy isomers were identified using the Coalescence Kick method and subsequently optimized at the MP2/aug-cc-pVDZ level of theory. Electronic stability was assessed via the outer valence Green’s function (OVGF) approach with the same aug-cc-pVDZ basis set. All examined anions exhibit exceptional electronic stability, with vertical electron detachment energies (VDEs) ranging from 10.70 to 12.37 eV, significantly exceeding the superhalogen threshold of 3.65 eV. Thermodynamic analyses indicate that aluminum atoms play a crucial role in stabilizing larger clusters by acting as a structural “glue”, thereby suppressing fragmentation through the loss of neutral **X**F_3_ or **Y**F_5_ units. In contrast, larger non-metallic analogs show an increased propensity toward dissociation. The potential of the heterovalent polynuclear superhalogen anions as weakly coordinating anions (WCAs) was further evaluated through molecular electrostatic potential (ESP) analysis. The results demonstrate that combining different central atoms within boron-based frameworks leads to a more homogeneous charge distribution, enhancing weakly coordinating behavior.

## 1. Introduction

Modern chemistry increasingly seeks to probe and extend the limits of molecular behavior. Superspecies represent a distinct class of molecular systems whose physicochemical properties exceed those of conventional atoms, ions, and molecules. From superhalogens [1,2] and superalkalis [3,4,5] to superacids [6,7,8] and superbases [9,10], these entities defy established chemical expectations, exhibiting enhanced reactivity, stability, or electronic characteristics beyond standard theoretical predictions. Their study offers new opportunities for advancing catalysis, materials science, and the fundamental understanding of chemical bonding.

One of the most important classes of superspecies comprises superhalogens, defined as neutral radical systems whose electron affinities exceed that of the chlorine atom (3.6 eV) [1]. Importantly, this definition does not specify either composition or structure (in the sense of the presence of particular atoms, bonds, or functional groups), but relies solely on an energetic criterion related to how strongly the excess electron is bound. The anions formed upon attachment of an extra electron to neutral superhalogens are commonly referred to as superhalogen anions. In this case, however, the same energetic criterion pertains to the energy required to detach the excess electron from the anionic system, rather than to the electron attachment energy of the neutral species (i.e., its electron affinity).

Superhalogens are particularly attractive among superspecies due to their extreme electron affinities and oxidizing power. Superhalogen anions and other weakly coordinating anion (WCA) systems have recently attracted increasing attention in the context of energy storage technologies, as they constitute important components of advanced electrolyte materials [11,12,13,14]. Experimental investigation of such systems typically requires prior synthesis, which can be challenging and resource-intensive. Therefore, theoretical prediction and screening of superhalogen/WCA candidates represent an efficient initial strategy, enabling identification of systems with properties optimized for specific applications (e.g., batteries) before experimental validation. This theory-guided approach has been increasingly adopted in recent studies focused on the rational design of next-generation electrolyte materials [15,16,17,18].

Notably, many superhalogens follow simple structural design rules, commonly described by the general formula MX_k+1_ (where M is a central atom of valence k coordinated by electronegative ligands X) [1], enabling systematic and rational construction. This conceptual clarity makes computational design a particularly powerful strategy for discovering new species and guiding experimental efforts. Importantly, comparisons between theoretical predictions and experimental measurements have demonstrated that calculations performed on superhalogen anions (i.e., MX_k+1_^−^) provide more reliable and directly verifiable results than those on neutral counterparts [19,20,21]. While early theoretical work on superhalogens largely centered on superhalogen anions containing one (M) atom (e.g., NaCl_2_^−^, BeF_3_^−^, AlF_4_^−^ or SbF_6_^−^) [2,19,22,23,24], recent technological and methodological advances have opened the door to polynuclear superhalogen architectures. These emerging systems introduce new bonding patterns and electronic phenomena, expanding the design space of superhalogen chemistry. Notably, even for these more complex systems, simple design principles based on generalized M_n_X_kn+1_^−^-type formulas can still be applied, enabling rational structural construction. However, the exploration of their potential energy surfaces becomes significantly more challenging, requiring extensive computational searches to identify stable geometries and low-energy isomers. As a result, theoretical investigations of polynuclear superhalogen anions remain relatively scarce. Nevertheless, several representative polynuclear superhalogen anions have been reported in the literature, including Al_n_F_3n+1_^−^ (where n = 2–5) [25], Mg_2_Cl_5_^−^, Mg_3_Cl_7_^−^ [26], [M_2_(CN)_5_]^−^ (where M = Ca, Be) [27], Fe_2_(CN)_5_^−^, Fe_3_(CN)_7_^−^ [28], Ti_3_F_13_^−^ or Ge_3_F_13_^−^ [29], and most recently (Bi_n_F_5n+1_)^−^ (n = 2–4) [30] and (Sb_n_F_5n+1_)^−^ (with n = 2–80) [31]. Superhalogen anions are often regarded as a special class of weakly coordinating anions (WCAs). Their structures, containing multiple highly electronegative substituents X surrounding the core, allow for significant delocalization of the negative charge, a feature that underlies their classification as weakly coordinating anions (WCAs). This property can be strengthened either by enlarging the ligand in MX_k+1_^−^ (for example, using a bulkier X group such as: -OC(CF_3_)_3_, -C_6_F_5_) [32] or by adding further MX_k_ units to build polynuclear anions. In polynuclear superhalogen systems, the presence of multiple structural M centers allows incorporation of a larger number of X ligands and promotes enhanced charge delocalization, thereby further strengthening their weakly coordinating character.

Although many superhalogen anions are based on metals as central atoms, recent theoretical studies have explored systems featuring non-metallic central atoms. Examples include chain-like boron–nitrogen polynuclear anions (BF_3_(BN)_n_F_4n+1_)^−^ with alternating B and N centers [33], boron-centered superhalogen anions such as B_12_H_13_^−^ or CB_11_H_12_^−^ [34], and heterocyclic superhalogen species incorporating N, B, and S elements [35]. These works expand the scope of superhalogen chemistry beyond classical metal-centered motifs and illustrate the versatility of non-metal frameworks in achieving high electron detachment energies. Superhalogen anions with non-metallic central atoms can be particularly attractive for several reasons. Firstly, non-metals typically possess high electronegativity, which facilitates effective delocalization of the negative charge over the whole molecule. Secondly, their smaller size and accessible p orbitals enable the formation of stable polynuclear frameworks, further enhancing charge delocalization. Finally, using non-metallic centers allows the design of superhalogens with tailored chemical properties, such as increased Lewis acidity or potential applications as superacid precursors, opening new opportunities in catalysis and materials chemistry.

Another interesting approach is the design of superhalogen anions containing different central atoms. Recently, a study on this topic was published, demonstrating that this strategy may be useful for the development of superacids. In particular, this study focused on superhalogen BAlF_7_^−^ and AsSbF_11_^−^ anions as precursors for superacids [36]. It was shown that acids containing two different central atoms, such as HBAlF_7_^−^ and HAsSbF_11_^−^, exhibit higher acidity compared to the corresponding dinuclear systems with identical central atoms (i.e., HB_2_F_7_^−^, HAl_2_F_7_^−^, HAs_2_F_11_^−^, and HSb_2_F_11_^−^, respectively).

Motivated by these developments, we sought to combine both concepts in the design of new superhalogen anions. Specifically, we investigate systems featuring central atoms with different valencies (from groups 13 and 15 of the periodic table) to examine how these structural variations influence the geometry, stability, and weakly coordinating properties of the resulting anions. The present study employs a modified general formula for superhalogen anions, (**X***_n_***Y***_n’_*F_{(3*n*+5*n’*)+1}_)^−^, where **X** = B and/or Al, **Y** = P and/or As, and *n* + *n′* = 2–4, with at least one central atom originating from a different periodic group to ensure distinct valencies (i.e., neither *n* nor *n’* equals 0). This choice enables a systematic comparison between purely non-metallic superhalogens and related systems containing a metal or metalloid center.

## 2. Results and Discussion

### 2.1. Homovalent (X_n_F_3n+1_)^−^ and (Y_n_F_5n+1_)^−^ Anions (Where n = 2–4; X = B, Al and Y = P, as)

**Figure 1:**
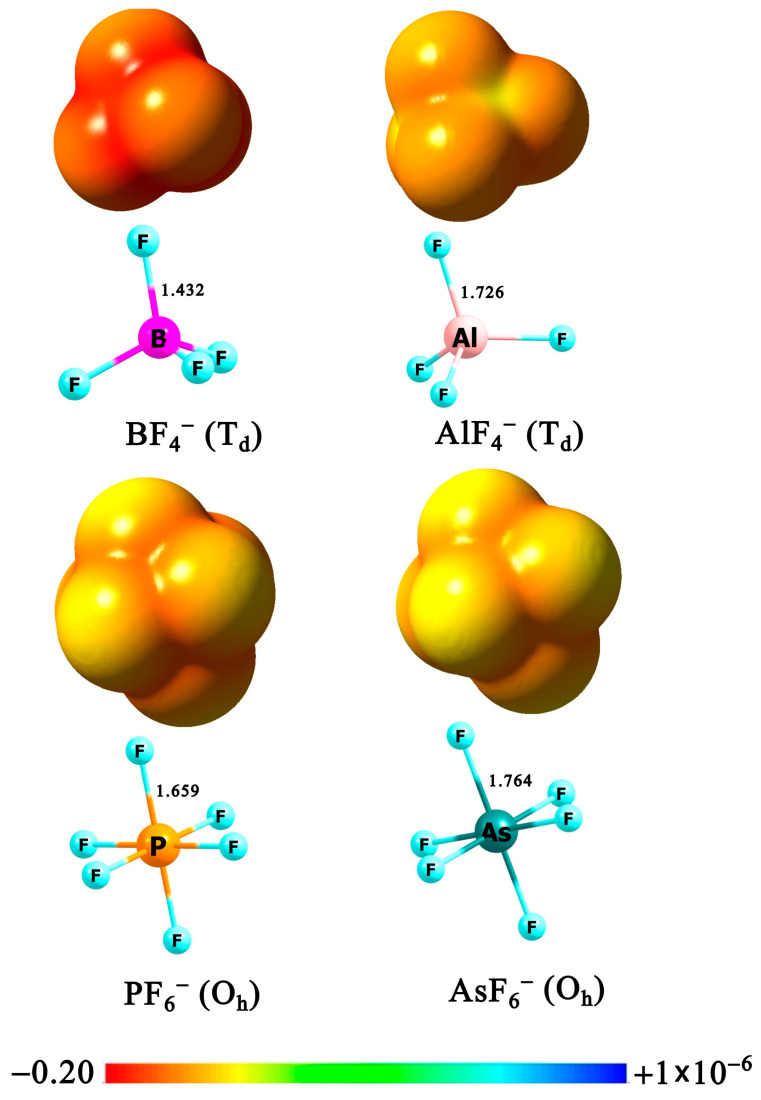
The equilibrium structures of the BF_4_^−^, AlF_4_^−^, PF_6_^−^, and AsF_6_^−^, along with the corresponding molecular electrostatic potential (ESP) maps computed from MP2 electron densities and plotted on the 0.001 e/bohr^3^ isodensity surface. Electrostatic potential values are given in atomic units (a.u.).

The mononuclear BF_4_^−^, AlF_4_^−^, PF_6_^−^ and AsF_6_^−^ are well described in the literature. Based on their structures, the first two display tetrahedral geometry, while the remaining two adopt an octahedral geometry [1,23,25,37,38,39,40]. The BF_4_^−^, PF_6_^−^, and AsF_6_^−^ are widely employed as counterions in coordination chemistry, catalysis, electrochemistry, and materials science, owing to their high electrochemical and thermal stability, and weak donor ability [41,42]. A prominent example of the importance of BF_4_^−^ and PF_6_^−^ is their widespread use in ionic liquids, which are used as electrolytes in lithium-ion batteries. The closely related AsF_6_^−^ anion is known as the conjugate base of the superacid HAsF_6_ [43]. In contrast, AlF_4_^−^ is predominantly used in biochemical and bioinorganic contexts as a structural and electronic mimic of phosphate, enabling mechanistic studies of phosphoryl transfer reactions in enzymes and G-proteins [44,45,46]. All these anions are characterized by high electronic stability (calculated VDEs at OVGF/6-31+G(3df)//MP2/6-31+G(d) for BF_4_^−^, PF_6_^−^, AsF_6_^−^, and AlF_4_^−^ are 8.98, 9.43, 10.54, and 9.79 eV, respectively [2]). The AlF_4_^−^ anion, however, is generally not considered a weakly coordinating anion (WCA) [32]. Unlike the other WCAs, AlF_4_^−^ can react with water and certain cations, whereas BF_4_^−^, PF_6_^−^, and AsF_6_^−^ are stable in solution and essentially chemically inert. Analysis of the electrostatic potential (ESP) reveals subtle differences in its spatial distribution. In AlF_4_^−^, the most negative potential is largely confined to the surface regions above the fluorine atoms, whereas the interligand regions above the Al center are noticeably less negative. In contrast, for BF_4_^−^, PF_6_^−^, and AsF_6_^−^, the ESP is more evenly distributed over the molecular surface, including the interligand regions, resulting in a more homogeneous potential and a reduced contrast between the fluorine regions and the central part of the surface. As shown in Figure 1, the electrostatic potential becomes increasingly uniform across the series BF_4_^−^, PF_6_^−^, and AsF_6_^−^, which is consistent with their classification as moderately, very, and extremely weakly coordinating anions, respectively [41]. It should be noted that a narrow potential scale (from −0.2 to 1 × 10^–6^ a.u.) was used to represent the ESP in order to capture subtle variations while still highlighting any local regions of positive potential.

**Figure 2:**
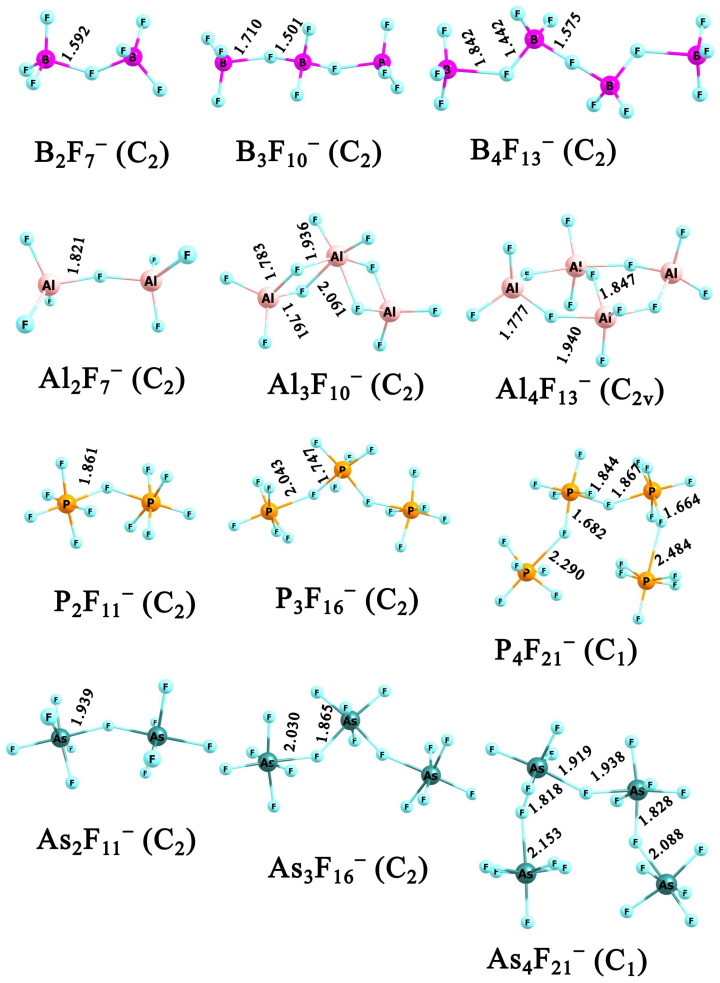
The equilibrium structures of (**X**_n_F_3n+1_)^−^ and (**Y**_n_F_5n+1_)^−^ anions (for n = 2–4; **X** = B, Al; and **Y** = P, As) obtained at MP2/aug-cc-pVDZ level.

Among the polynuclear (**X**_n_F_3n+1_)^−^ and (**Y**_n_F_5n+1_)^−^ anions (where n = 2–4; **X** = B, Al; and **Y** = P, As), dinuclear and trinuclear species have been previously reported. Some, such as B_2_F_7_^−^ [47,48,49], Al_2_F_7_^−^ [50], Al_3_F_10_^−^ [51], and As_2_F_11_^−^ [52,53,54], have been experimentally confirmed, while the others are known only from computational studies [2,25,55,56,57]. Among tetranuclear anions, only Al_4_F_13_^−^ has been theoretically explored [25,50]. To ensure a consistent comparison, we re-examined potential energy surfaces of (**X**_n_F_3n+1_)^−^ and (**Y**_n_F_5n+1_)^−^ anions (where n = 2–4; **X** = B, Al; and **Y** = P, As) and evaluated the electronic stability of the most stable structures. The lowest-energy isomeric structures are presented in Figure 2, whereas the detailed coordinates of the remaining isomers, whose relative energies do not exceed 10 kcal/mol, along with their corresponding relative energies, are provided in the Supplementary Materials (Table S1).

The geometries of the dinuclear and trinuclear structures obtained by us are generally consistent with previous studies. In particular, all the dinuclear anions and tri- and tetranuclear boron, phosphorous, and arsenic-based species feature a fluorine atom bridging two, three, or four **X**F_3_ or **Y**F_5_ units, resulting in chain-like structures. However, in the case of **Y**_4_F_21_^−^ anions, structures remain chain-like but adopt a U-shaped geometry. This is in agreement with earlier studies on the related bismuth clusters (Bi_4_F_21_^−^ [30]) with the same stoichiometry. Notably, the fully extended forms are only slightly less stable, by 2.42 for P_4_F_21_^−^ and 1.91 kcal/mol for As_4_F_21_^−^ (see Table S1 in the Supplementary Materials). In contrast, the structures of the Al_3_F_10_^−^ and Al_4_F_13_^−^ are quite different and become more compact. As shown in Figure 2, in the Al_3_F_10_^−^ anion, the three aluminum atoms are connected through two fluorine atoms, with the central aluminum adopting a quasi-octahedral coordination. In Al_4_F_13_^−^, the structure forms an eight-membered ring where each aluminum atom is bridged by a fluorine. Additionally, one F atom links two Al atoms across the ring, giving them a quasi-bipyramidal geometry. In the earlier works, this structure was never described as the lowest energy isomer. According to our findings, the Al_4_F_13_^−^ structures previously reported as the most stable are less favorable in energy by 2.4 kcal/mol (see Table S1 in the Supplementary Materials) and 12.8 kcal/mol (see in Ref. [25]).

The vertical electron detachment energies estimated for the most stable (B_n_F_3n+1_)^−^, (Al_n_F_3n+1_)^−^, (P_n_F_5n+1_)^−^, and (As_n_F_5n+1_)^−^ anions (for n = 2–4) are significant (spanning the 10.70–12.07, 10.87–12.37, 11.05–11.85, and 11.49–12.41 eV ranges, respectively; see Table S2 in the Supplementary Materials). Analysis of our calculations indicates that the electronic stability of studied (**X**_n_F_3n+1_)^−^ and (**Y**_n_F_5n+1_)^−^ anions generally increases with increasing n. However, within the series of (Al_n_F_3n+1_)^−^ anions, Al_3_F_10_^−^ is characterized by a lower VDE than Al_2_F_7_^−^ (by ~0.6 eV). This behavior can be attributed to the more compact structure of Al_3_F_10_^−^ relative to the more extended, chain-like structure of Al_2_F_7_^−^ (which is consistent with previous reports on the relationship between structure and electronic stability of polynuclear superhalogen anions; see Ref. [2] and publication cited therein). In addition, the largest increase in electronic stability (by 0.8–1.6 eV) occurs for smaller anionic clusters (n = 1–3), whereas the addition of another BF_3_ or **Y**F_5_ unit increases the stability by only 0.02–0.4 eV (the smallest change is observed for P_4_F_16_^−^ relative to P_3_F_11_^−^). This, in turn, might be related to the fact that as n increases, the number of fluorine atoms increases, while the **X** and **Y** atoms have relatively small atomic radii, and the repulsion between the accumulated fluorine atoms grows, rendering anion formation progressively less favorable. Again, an exception is found for Al-based polynuclear anions, where after an initial decrease for Al_3_F_10_^−^, electronic stability increases substantially by ~1.5 eV for Al_4_F_13_^−^ (relative to Al_3_F_10_^−^). This trend in electronic stability of (B_n_F_3n+1_)^−^ and (**Y**_n_F_5n+1_)^−^ anions is also reflected in the thermodynamic data (see Table S4 in the Supplementary Materials): larger anionic clusters are more prone to detachment of BF_3_ or **Y**F_5_, making P_3_F_11_^−^, P_4_F_16_^−^, and As_4_F_16_^−^ unlikely to form. It is worth mentioning that studied (Al_n_F_3n+1_)^−^ anions are expected to be thermodynamically stable, as indicated by the positive Gibbs free energy changes in the considered reactions under standard conditions at 298.15 K (ΔG_r_^298^) (ranging from 24.4 to 44.7 kcal/mol), suggesting that further growth of the cluster is possible (i.e., n > 4), along with a potential increase in its electronic stability.

### 2.2. Heterovalent XYF_9_^−^ Anions (Where X = B and/or Al, Y = P and/or as)

**Figure 3:**
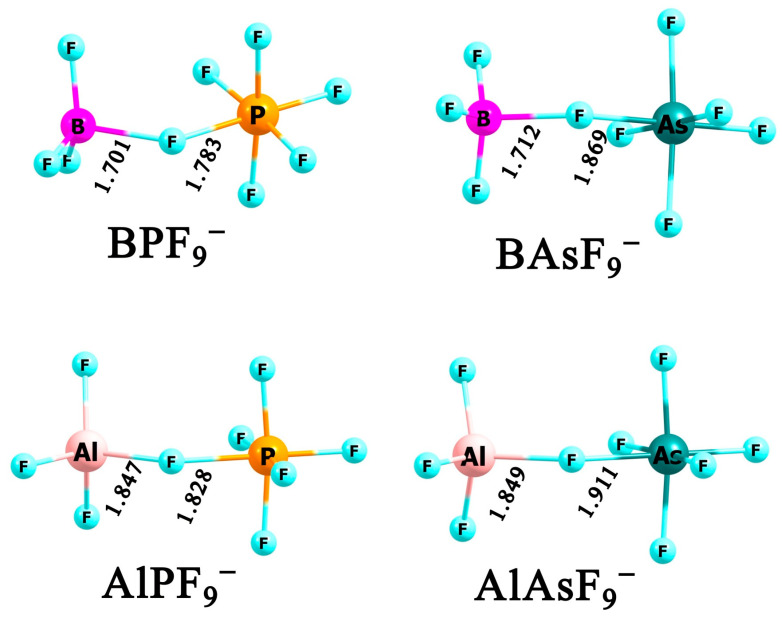
The equilibrium structures of **XY**F_9_^−^ anions (where **X** = B and/or Al, **Y** = P and/or As) obtained at MP2/aug-cc-pVDZ level.

In accordance with our goal of combining only central atoms of different valencies, there are only four possible dinuclear anions, which can be described by the formulas BPF_9_^−^, AlPF_9_^−^, BAsF_9_^−^, and AlAsF_9_^−^. Our calculations indicate that each of these anions forms a single stable isomer of C_S_ symmetry (see Figure 3), in which a fluorine atom bridges the **X**F_3_ and **Y**F_5_ units, thereby preserving tetrahedral and octahedral coordination environments of the **X** and **Y** centers, respectively.

To assess the susceptibility of the **XY**F_9_^−^ anions to fragmentation, we calculated the Gibbs free energies (ΔG_r_^298^) associated with the detachment of either **X**F_3_ or **Y**F_5_. Our results indicate that all dinuclear **XY**F_9_^−^ anions are thermodynamically stable, as evidenced by positive ΔG_r_^298^ values ranging from 5.2 to 34.3 kcal/mol (see Table S4 in the Supplementary Materials). Notably, for B-based anions, detachment of BF_3_ is energetically more favorable (ΔG_r_^298^ = 5.2–5.6 kcal/mol), whereas for Al-based anions, cleavage of the **Y**F_5_ is less endergonic (ΔG_r_^298^ = 16.2–25.8 kcal/mol).

**Figure 4:**
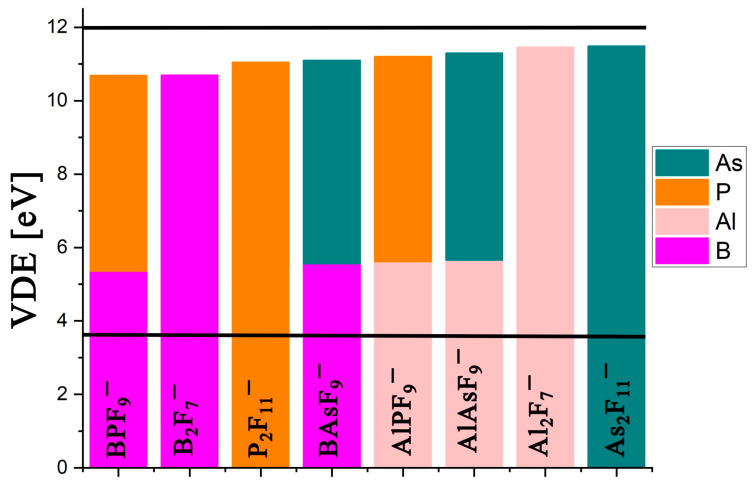
Calculated (at OVGF/aug-cc-pVDZ level) vertical detachment energies (VDEs, in eV) for the most stable **X**_2_F_7_^−^, **Y**_2_F_11_^−^, and **XY**F_9_^−^ anions (**X** = B and/or Al, **Y** = P and/or As), arranged in ascending order (for exact values see Table S2 in the Supplementary Materials).

As far as electronic stability is concerned, the vertical electron detachment energies estimated for the **XY**F_9_^−^ anions are significant (spanning the 10.7–11.3 eV range, see Table S2 in the Supplementary Materials). Figure 4 presents a graphical illustration (in bar chart form) of the calculated VDE values for the studied heterovalent and homovalent dinuclear anions. Two horizontal lines are included for reference: the superhalogen threshold (3.65 eV; lower line) and the average VDE value for four nuclear homonuclear superhalogen anions (12 eV; upper line). The upper line is purely a visual aid to highlight clusters with particularly high VDE values and does not represent a physical limit or indicate any correlation with thermodynamic stability. This chart allows comparison of the electron detachment energies across homovalent and heterovalent dinuclear anions.

As shown in (Figure 4), Al_2_F_7_^−^ and As_2_F_11_^−^ anions exhibit the highest VDEs, while the heterovalent AlAsF_9_^−^ superhalogen anion displays only slightly lower (by ~0.2 eV) value. Notably, the BPF_9_^−^ anion, despite having the lowest VDE in this group, shows electronic stability comparable to B_2_F_7_^−^, and only 0.35 eV below P_2_F_11_^−^. Therefore, BPF_9_^−^ represents an intriguing (as it is non-metallic and stable) dinuclear superhalogen anion.

To gain further insight into the structural and electronic stability of these dinuclear heterovalent anions, we performed NBO calculations to obtain atomic charges, Wiberg bond indices (WBI), and the composition of bonding orbitals (see Table S3 in the Supplementary Materials). The analysis indicates that all systems exhibit strong bond polarization, with the central atoms carrying high positive charges (B ~+1.55, Al ~+2.21, P ~+2.73, and As ~+2.86) and fluorine ligands are highly negative (−0.55 to −0.78), providing electrostatic stabilization. Notably, boron centers form stronger terminal and bridging bonds with F ligands (WBI ~0.64 and ~0.26, respectively) than aluminum (WBI ~0.35 and ~0.18, respectively). Although the Al–F bonds are weaker, the very high positive charge on aluminum (+2.21) allows it to act as a strong electrostatic linker, effectively connecting neighboring fragments. Differences between phosphorus and arsenic are more subtle: phosphorus forms slightly stronger terminal and bridging bonds with fluorine ligands (WBI ~0.53–0.54 and ~0.36–0.32, respectively) than arsenic (terminal WBI ~0.49–0.50, bridging WBI ~0.33–0.29), and in both cases, d-orbitals contribute significantly to hybridization (e.g., P sp^2.7^d^1.7^; see Table S3 in the Supplementary Materials).

**Figure 5:**
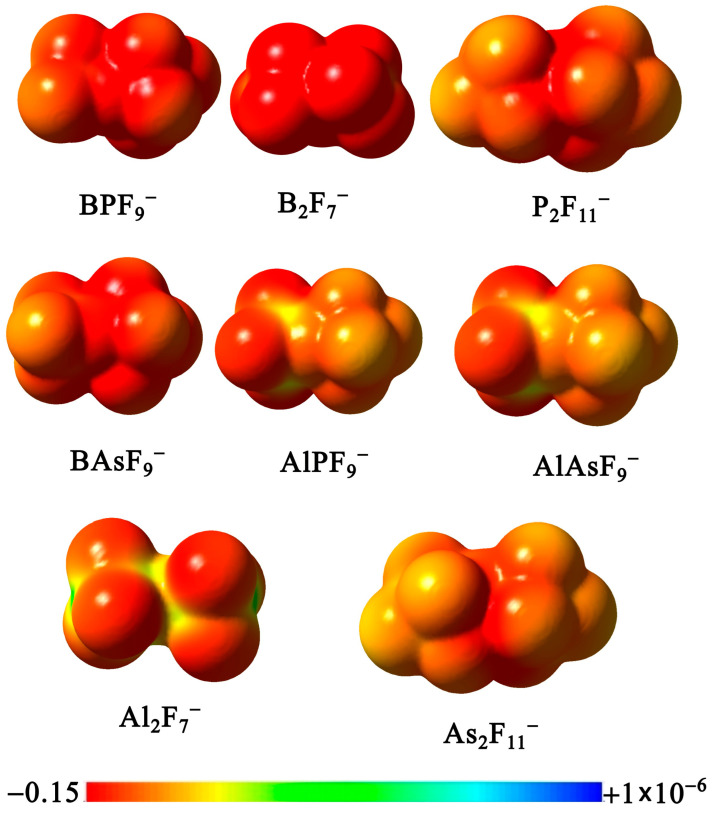
Molecular electrostatic potential (ESP) maps of **X**_2_F_7_^−^, **Y**_2_F_11_^−^, and **XY**F_9_^−^ anions (**X** = B and/or Al, **Y** = P and/or As) computed from MP2 electron densities and plotted on the 0.001 e/bohr^3^ isodensity surface. Electrostatic potential values are given in atomic units (a.u.).

As mentioned, an important feature of superhalogen anions is charge delocalization, which underlies their weakly coordinating nature. Figure 5 shows the ESPs for all studied dinuclear anions, arranged in order of increasing VDE. The ESP map shown corresponds to the same orientation of a given molecule as presented in Figure 2 (showing the structures of B_2_F_7_^−^, Al_2_F_7_^−^, P_2_F_11_^−^, and As_2_F_11_^−^) and Figure 3 (with the BPF_9_^−^, AlPF_9_^−^, BAsF_9_^−^, and AlAsF_9_^−^ structures).

At first glance, mixing central atoms does not appear to improve the uniformity of the electrostatic potential of the superhalogen anion. In particular, in heterovalent Al-based anions, the negative potential shifts toward the fluorine atoms bonded to Al. However, comparison with the ESP map of Al_2_F_7_^−^ shows that replacing one Al with P or As actually disperses the potential, making it slightly less gradual and smooth. In the case of the B**Y**F_9_^−^ anions, this effect may further enhance their weakly coordinating properties compared to B_2_F_7_^−^.

### 2.3. Heterovalent (X_n_Y_n’_F_{(3n+5n’)+1}_)^−^ Anions (Where n + n’ = 3 and X = B and/or Al, Y = P and/or as)

**Figure 6:**
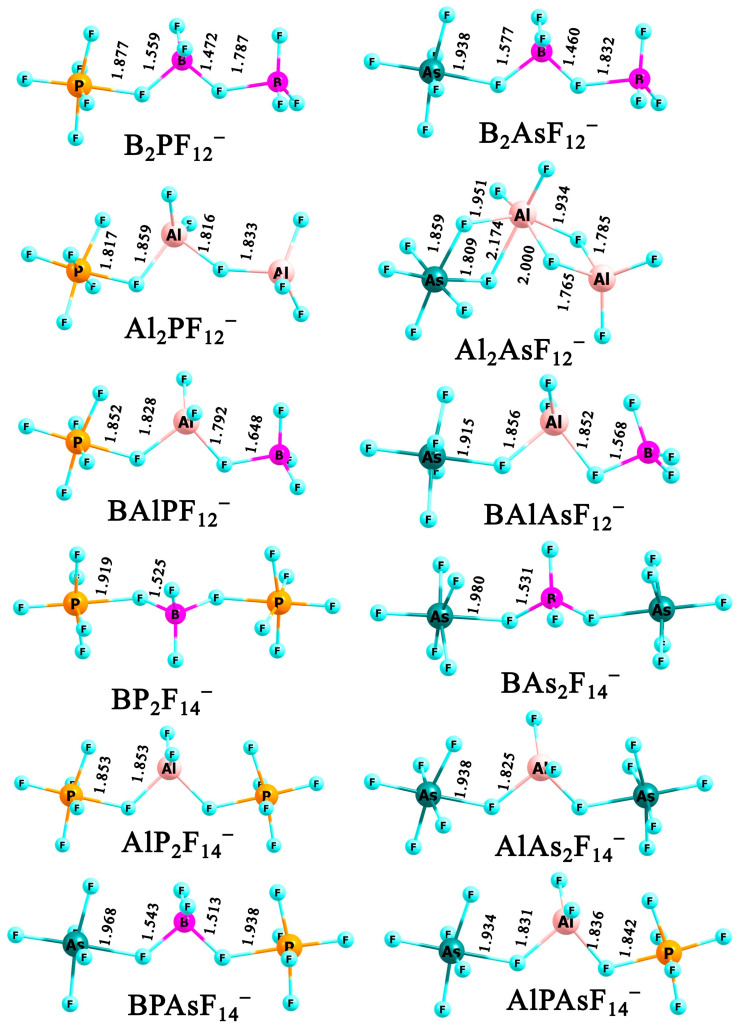
The equilibrium structures of (**X***_n_***Y***_n’_*F_{(3*n*+5*n*_*_’_*_)+1}_)^−^ anions (where *n* + *n’* = 3 and **X** = B and/or Al, **Y** = P and/or As) obtained at MP2/aug-cc-pVDZ level.

Within the class of trinuclear anions, in which at least one of the central atoms possesses a different valence state, twelve distinct compounds can be constructed. Their corresponding molecular formulas are as follows: B_2_PF_12_^−^, B_2_AsF_12_^−^, Al_2_PF_12_^−^, Al_2_AsF_12_^−^, BAlPF_12_^−^, BAlAsF_12_^−^, BP_2_F_14_^−^, BAs_2_F_14_^−^, AlP_2_F_14_^−^, AlAs_2_F_14_^−^, BPAsF_14_^−^, and AlPAsF_14_^−^. The most stable geometries identified for the aforementioned anions are presented in Figure 6.

The exploration of the potential energy surfaces of each of the twelve anions reveals the presence of a limited number of low-lying (within the considered energy range of 0–10 kcal/mol) isomers (two to five) (see Table S1 in the Supplementary Materials). Notably, in systems containing at least one boron atom, additional isomers arise from different relative arrangements of the BF_3_ unit with respect to the other central atoms, whereas in aluminum-containing species, the observed isomerism is associated with the ability of the aluminum atom to adopt an octahedral coordination geometry. Nevertheless, as shown in Figure 6, the most stable structures of nearly all studied heterovalent trinuclear anions exhibit elongated geometries, which can be attributed to the minimization of fluorine–fluorine repulsion. The exception is the structure of Al_2_AsF_12_^−^, in which the aluminum atom adopts an octahedral coordination, resulting in a compact geometry (its corresponding elongated isomer is, however, nearly isoenergetic, being only 0.3 kcal/mol higher in energy; see Table S1 in the Supplementary Materials). It is also noteworthy that the most stable **XY**_2_F_14_^−^-type structures exhibit C_2v_ symmetry when the two **Y** atoms are identical, whereas C_S_ symmetry is observed when the **Y** atoms are different. In contrast, the most stable **X**_2_**Y**F_12_^−^ anionic structures generally do not exhibit any symmetry, with the exception of Al_2_PF_12_^−^, which adopts C_S_ symmetry.

**Figure 7:**
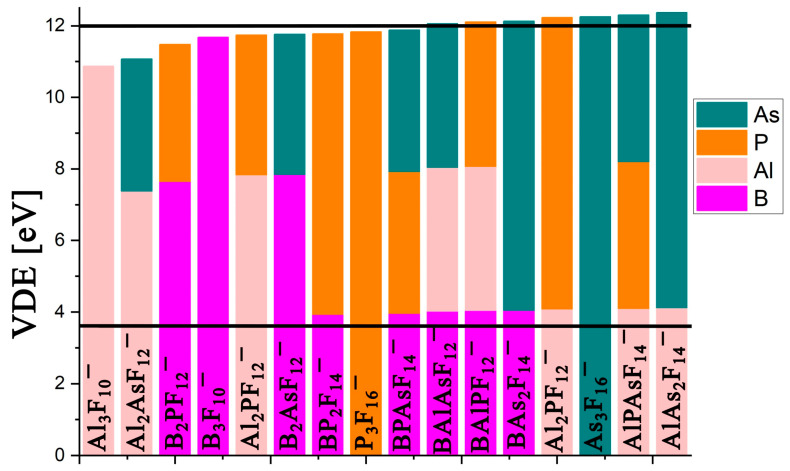
Calculated (at OVGF/aug-cc-pVDZ level) vertical detachment energies (VDEs, in eV) for the most stable **X**_3_F_10_^−^, **Y**_3_F_16_^−^, and (**X***_n_***Y***_n’_*F_{(3*n*+5*n’*)+1}_)^−^ anions (where *n* + *n’* = 3 and **X** = B and/or Al, **Y** = P and/or As), shown in ascending order (see Table S2 in the Supplementary Materials for exact VDE values).

As mentioned earlier, elongated structures of polynuclear superhalogen anions ensure high electronic stability. This trend is also observed for the heterovalent trinuclear anions investigated here, whose calculated vertical detachment energies (VDEs) are very high, ranging from 11.07 to 12.37 eV. The lowest VDE is determined for this most compact Al_2_AsF_12_^−^, whereas the highest VDE is found for the AlAs_2_F_14_^−^ anion. As shown in Figure 7, six heterovalent trinuclear anions exhibit electron-binding energies exceeding 12 eV, two of which bind the excess electron more strongly than the corresponding homovalent As_3_F_16_^−^ anion (see Table S2 in the Supplementary Materials for exact VDEs values).

Analysis of the NBO calculations performed for trinuclear heterovalent systems reveals trends in electronic and structural stabilization analogous to those observed in the dinuclear heterovalent anions. In particular, NBO analysis (see Table S3 in the Supplementary Materials) for these structures confirm the following observations. All trinuclear systems exhibit strong bond polarization, with the central atoms maintaining high positive charges (B ~+1.54, Al ~+2.20, P ~+2.73, and As ~+2.86) and fluorine ligands are highly negative (−0.54 to −0.78), providing significant electrostatic stabilization. Consistent with the smaller clusters, boron centers form stronger terminal (WBI ~0.64–0.66) and bridging bonds with F ligands (WBI ~0.30–0.45) than aluminum centers (terminal WBI ~0.35–0.38, bridging WBI ~0.15–0.22). Although individual Al–F bonds are weaker (low WBI), the very high positive charge on aluminum (~+2.22) allows it to function as a structural “glue” as it can coordinate multiple fluorine ligands simultaneously, effectively connecting distant PF_6_, AsF_6_, or BF_4_ fragments. Differences between phosphorus and arsenic remain subtle. Phosphorus forms slightly stronger terminal (WBI ~0.54–0.57) and bridging bonds (WBI ~0.28–0.40) than arsenic (terminal WBI ~0.50–0.51, bridging WBI ~0.27–0.35), with d-orbitals contributing significantly to hybridization (e.g., P: sp^2.6^d^1.5^; As: sp^2.8^d^1.7^; see Table S3 in the Supplementary Materials). Bridging bonds generally show increased p-character on the central atoms, particularly for aluminum, where hybridization in the bridges reaches sp^4.0^ to sp^5.9^, highlighting its role in reinforcing cluster connectivity through predominantly electrostatic interactions.

As far as thermodynamic stability is concerned, all systems containing at least one aluminum atom exhibit higher thermodynamic stability (ΔG_r_^298^ = 3.2–14.7 kcal/mol) than anions lacking this metal (ΔG_r_^298^ = −1.9–0.6 kcal/mol; see Table S4 in the Supplementary Materials). In anions containing boron, the most favorable process is almost always the detachment of BF_3_, whereas in aluminum-containing anions, the least endergonic reaction is the removal of **Y**F_5_. Analysis of the data presented in Table S4 indicates that aluminum atoms play a crucial role in establishing the thermodynamic stability of trinuclear heterovalent superhalogen anions. This is exemplified by two stoichiometrically analogous structures, BAs_2_F_14_^−^ and AlAs_2_F_14_^−^. Our calculations show that the former is thermodynamically unstable (ΔG_r_^298^ = −1.9 kcal/mol for BF_3_ detachment), whereas the latter is the most thermodynamically stable among all the heterovalent trinuclear systems studied, and exhibits the highest electronic stability (VDE = 12.37 eV). Although the B-based anions are only marginally stable (or even slightly unstable; its magnitude is small and therefore the conclusions should be treated with appropriate caution, considering the limited accuracy of the computational method, which does not allow for a fully definitive assessment in such cases), it is worth highlighting that the presented structures remain well-integrated. Moreover, when compared with their homovalent corresponding superhalogen anions, the **X**–F–**Y** distances are never larger than those observed in homovalent tetranuclear superhalogen anions and are (in a few cases) only slightly longer than in the corresponding trinuclear homovalent anions. This structural integrity, together with high electronic stability, suggests that the designed anions could potentially exist under special conditions (e.g., at lower temperatures) or in the presence of a suitable countercation.

**Figure 8:**
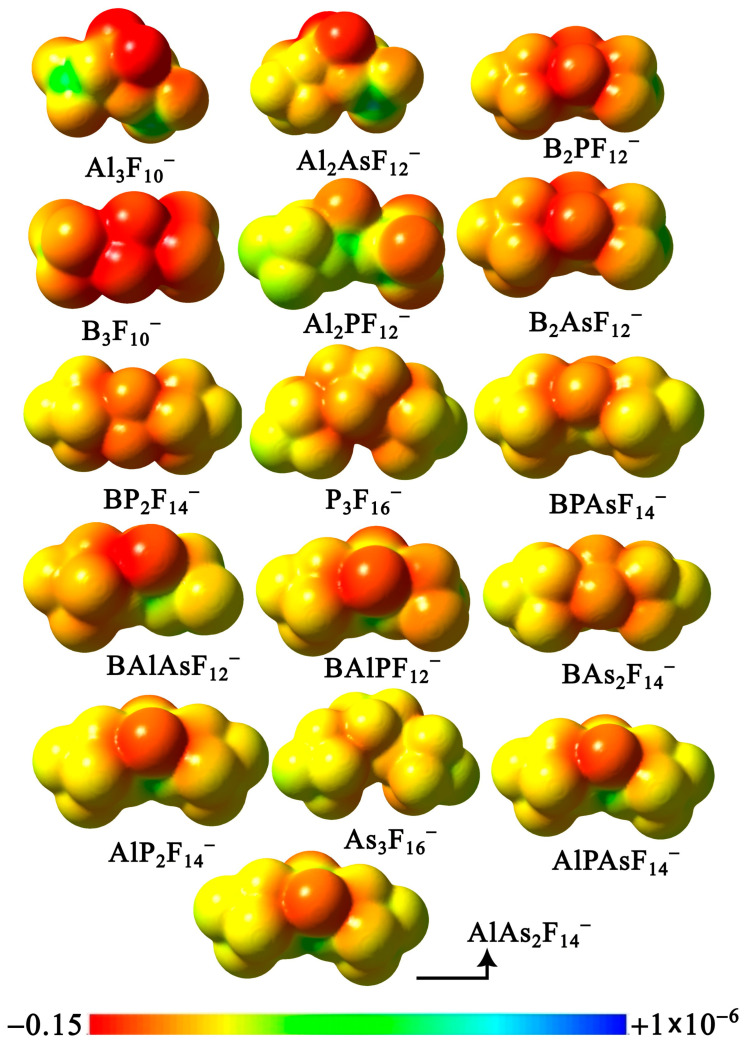
Molecular electrostatic potential (ESP) maps **X**_3_F_10_^−^, **Y**_3_F_16_^−^, and (**X***_n_***Y***_n’_*F_{(3*n*+5*n’*)+1}_)^−^ anions (where *n* + *n’* = 3 and **X** = B and/or Al, **Y** = P and/or As), computed from MP2 electron densities and plotted on the 0.001 e/bohr^3^ isodensity surface.

In Figure 8, we present the electrostatic potential on the molecular isodensity surfaces (in the same orientation as in Figure 2 and Figure 6) of the studied most stable **X**_3_F_10_^−^, **Y**_3_F_16_^−^, and (**X***_n_***Y***_n’_*F_{(3*n*+5*n’*)+1}_)^−^ anions (where *n* + *n’* = 3 and **X** = B and/or Al, **Y** = P and/or As), arranged in order of increasing VDE.

As can be seen, all molecules containing an aluminum atom are characterized by an uneven distribution of the electrostatic potential, with a significant shift in the negative regions toward the fluorine atoms bonded to aluminum and clearly defined electron-deficient areas (green lobes). It is worth emphasizing, however, that these green regions are considerably less pronounced in the anions identified as the strongest electron acceptors (see AlPAsF_14_^−^ and AlAs_2_F_14_^−^). On the other hand, it appears that, in anions containing a boron atom but lacking aluminum, mixing may favorably influence their non-coordinative character. For example, comparing the structures of B_3_F_10_^−^ with B_2_PF_12_^−^ or B_2_AsF_12_^−^ shows that the electrostatic potential is more evenly distributed across the heterovalent anions.

### 2.4. Heterovalent (X_n_Y_n’_F_{(3n+5n’)+1}_)^−^ Anions (Where n + n’ = 4 and X = B and/or Al, Y = P and/or as)

**Figure 9:**
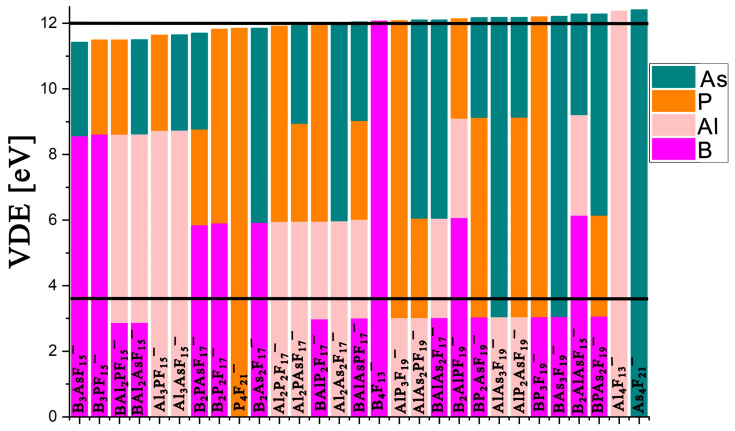
Calculated (at the OVGF/aug-cc-pVDZ level) vertical detachment energies (VDEs, in eV) for the most stable **X**_3_F_10_^−^, **Y**_3_F_16_^−^, and (**X***_n_***Y***_n’_*F_{(3*n*+5*n’*)+1}_)^−^ anions (where *n* + *n’* = 4 and **X** = B and/or Al, **Y** = P and/or As), shown in ascending order (see Table S2 in the Supplementary Materials for exact VDE values).

Based on the general formula (**X***_n_***Y***_n’_*F_{(3*n*+5*n’*)+1}_)^−^, with the constraint that *n* + *n’* = 4 (and with **X** = B and/or Al, **Y** = P and/or As), there are 25 possible superhalogen anions: B_3_PF_15_^−^, B_3_AsF_15_^−^, Al_3_PF_15_^−^, Al_3_AsF_15_^−^, B_2_AlPF_15_^−^, B_2_AlAsF_15_^−^, BAl_2_PF_15_^−^, BAl_2_AsF_15_^−^, B_2_P_2_F_17_^−^, B_2_As_2_F_17_^−^, B_2_PAsF_17_^−^, Al_2_P_2_F_17_^−^, Al_2_As_2_F_17_^−^, Al_2_PAsF_17_^−^, BAlP_2_F_17_^−^, BAlAs_2_F_17_^−^, BAlPAsF_17_^−^, BP_3_F_19_^−^, BAs_3_F_19_^−^, AlP_3_F_19_^−^, AlAs_3_F_19_^−^, BP_2_AsF_19_^−^, BPAs_2_F_19_^−^, AlP_2_AsF_19_^−^, and AlPAs_2_F_19_^−^. The most stable structures found for those 25 anions are presented in Figures S1–S3, while the detailed coordinates of all heterovalent tetranuclear superhalogen anions and their isomeric forms (within 0–10 kcal/mol) are provided in Table S1 in the Supplementary Materials. Structural analysis reveals that the majority of heterovalent boron-based tetranuclear anionic species do not retain their structural integrity, instead adopting geometries more consistent with complexes in which BF_3_ is coordinated to smaller heterovalent anionic species (in terms of the number of central atoms; see Figures S1–S3 in the Supporting Materials). Clearly, larger systems are prone to thermodynamic instability; therefore, our discussion begins with electronic stability, as this factor determines whether the formation of such anions is feasible relative to heterovalent trinuclear and dinuclear anionic forms. According to our findings, the vertical detachment energies (VDEs), estimated for all studied heterovalent tetranuclear anions, are very high, ranging from 11.42 to 12.28 eV. The lowest VDE is determined for B_3_AsF_15_^−^, whereas the highest VDE is found for the B_2_AlAsF_15_^−^ and BPAs_2_F_19_^−^ anions (see Table S2 in the Supplementary Materials). Figure 9 presents the calculated VDEs for the most stable structures of all 25 heterovalent tetranuclear superhalogen anions, together with B_4_F_13_^−^, Al_4_F_13_^−^, P_4_F_21_^−^, and As_4_F_21_^−^, which are included for comparison, arranged in ascending order of VDE values. As can be seen, none of the heterovalent superhalogen anions is electronically more stable than the homovalent Al_4_F_13_^−^ and As_4_F_21_^−^ species. However, it is worth noting that 12 out of 25 studied anions exhibit VDEs above 12 eV.

**Figure 10:**
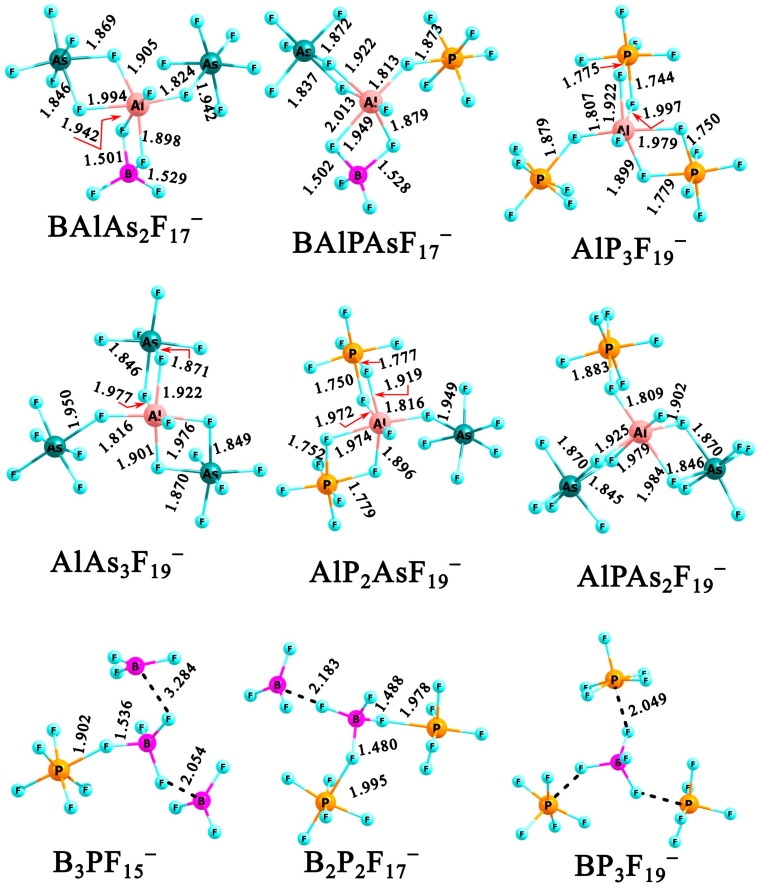
The equilibrium structures of selected (**X***_n_***Y***_n’_*F_{(3*n*+5*n*_*_’_*_)+1}_)^−^ anions (where *n* + *n’* = 4 and **X** = B and/or Al, **Y** = P and/or As) obtained at MP2/aug-cc-pVDZ level.

Regarding thermodynamic stability, only six of the anions with VDE > 12 eV are predicted to be resistant to **X**F_3_ or **Y**F_5_ detachment (see Table S4 in the Supplementary Materials). Figure 10 displays the structures of the six anions for which we calculated the lowest-energy detachment processes: BF_3_ loss in BAlAs_2_F_17_^−^ and BAlPAsF_17_^−^ (ΔG_r_^298^ = 1.45 and 2.40 kcal/mol, respectively), PF_5_ loss in AlP_3_F_19_^−^, AlP_2_AsF_19_^−^, and AlPAs_2_F_19_^−^ (ΔG_r_^298^ = 2.15–2.90 kcal/mol), and AsF_5_ loss in AlAs_3_F_19_^−^ (ΔG_r_^298^ = 11.3 kcal/mol). The final row of the Figure shows the B_3_PF_15_^−^, B_2_P_2_F_17_^−^, and BP_3_F_19_^−^ anions. These species are largely unstable towards BF_3_ or PF_5_ detachment; nevertheless, they are of interest as representative purely non-metallic clusters described by the general formula (**X***_n_***Y***_n’_*F_{(3*n*+5*n’*)+1}_)^−^ for *n* + *n’* = 4.

As shown, all six Al-containing structures are compact, featuring a central Al atom in an octahedral coordination environment that effectively acts as a structural “glue”. A similar trend is observed for the studied heterovalent tetranuclear anions characterized by VDE < 12 eV. In particular, the majority of thermodynamically stable structures are anions containing at least one Al atom (see Al_3_PF_15_^−^, Al_3_AsF_15_^−^, BAl_2_AsF_15_^−^, Al_2_P_2_F_17_^−^, Al_2_As_2_F_17_^−^, Al_2_PAsF_17_^−^, and BAlP_2_F_17_^−^; Figures S1 and S2 in the Supplementary Materials). As shown for heterovalent trinuclear anions, Al-containing species consistently exhibit enhanced thermodynamic stability. Accordingly, heterovalent tetranuclear anions appear viable only when at least one Al atom is present in the cluster. The structures of the B_3_PF_15_^−^, B_2_P_2_F_17_^−^, and BP_3_F_19_^−^ anions (see the bottom panel in Figure 10) can be viewed as complexes of smaller anions (i.e., BPF_9_^−^, BP_2_F_14_^−^, and BF_4_^−^) with neutral BF_3_ or PF_5_ molecules. Comparison of the VDE values of B_3_PF_15_^−^ (11.49 eV) and B_2_P_2_F_17_^−^ (11.82 eV) with those of BPF_9_^−^ (10.69 eV), BP_2_F_14_^−^ (11.78 eV), and B_2_P_12_^−^ (11.48 eV) reveals only a slight increase in electronic stability. In contrast, the BP_3_F_19_^−^ anion exhibits a higher VDE of 12.20 eV and can be described as a BF_4_^−^ core solvated by three PF_5_ molecules. The three PF_5_ units are arranged symmetrically around the BF_4_^−^ anion, resulting in a C_3_ symmetry complex. Considering that the VDE of BF_4_^−^ is 9.09 eV (calculated at the OVGF(full)/aug-cc-pVDZ level), this solvation results in an increase of approximately 3 eV in the electronic stability of the system.

Analysis of the NBO calculations (presented in Table S3 in the Supplementary Materials) for tetranuclear heterovalent systems, such as BAlAs_2_F_17_^−^_,_ BAlPAsF_17_^−^, AlP_3_F_19_^−^_,_ AlAs_3_F_19_^−^_,_ AlP_2_AsF_19_^−^_,_ and AlPAs_2_F_19_^−^, confirms the trends in stability and bonding observed in smaller systems. All tetranuclear systems exhibit pronounced bond polarization, which underpins their structural and electronic stability. Phosphorus and arsenic atoms maintain very high positive charges (P ~+2.73, As ~+2.86), while aluminum centers carry charges of ~+2.19 to +2.23. These strongly cationic centers, surrounded by fluorine ligands (−0.54 to −0.78), form a robust network of electrostatic interactions. In tetranuclear clusters, aluminum assumes a dominant role as a structural “glue.” In all the anionic clusters, the aluminum atom occupies a central position, acting as both a physical and electronic hub connecting PF_6_, AsF_6_, or BF_4_ units. Although individual Al–F bridging bonds have low Wiberg bond indices (~0.15–0.21), reflecting weak covalent character, the high positive charge on aluminum enables it to stabilize the cluster through multiple electrostatic interactions. Without aluminum, the strongly charged PF_6_ or AsF_6_ fragments would experience significant Coulombic repulsion. A distinctive feature of aluminum in these extended systems is its substantial involvement of d-orbitals in bonding, which is absent in boron. NBO data indicate that in complex fluorine bridges, aluminum hybridization includes a significant d-character, e.g., sp^2.77^d^1.35^, sp^2^.^89^d^1.61^, and in extreme cases sp^4.23^d^3.26^ or sp^3.57^d^2.54^ (see Table S3 in the Supplementary Materials). This additional d-orbital contribution allows aluminum to accommodate high coordination numbers and adapt flexibly to cluster geometry, reinforcing its role as a structural “glue.”

**Figure 11:**
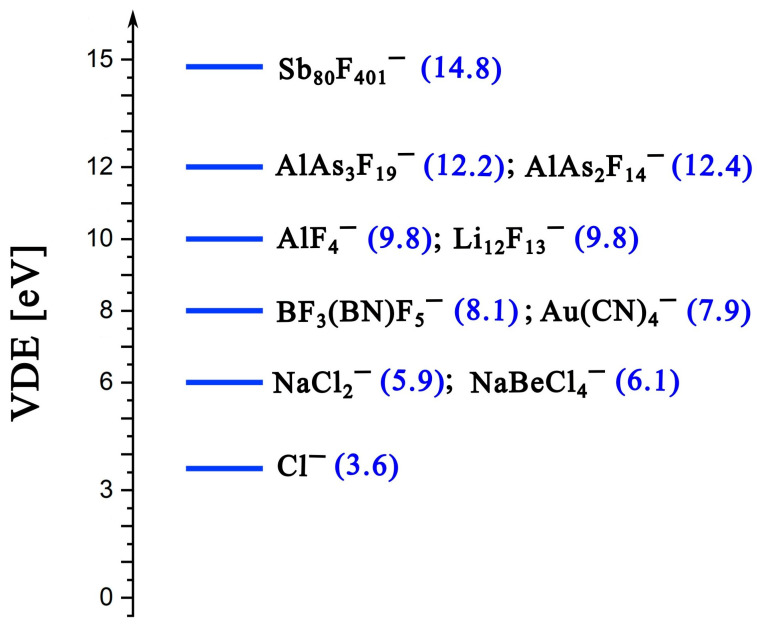
Vertical detachment energies (VDEs; highlighted in blue) of selected heterovalent polynuclear anions from this work, placed in the context of the superhalogen anion scale. Reference VDEs are taken from Ref. [58] for Cl^−^, Ref. [19] for NaCl_2_^−^, Ref. [59] for NaBeCl_4_^−^, Ref. [33] for BF_3_(BN)F_5_^−^, Ref. [60] for Au(CN)_4_^−^, Ref. [23] for AlF_4_^−^, Ref. [61] for Li_12_F_13_^−^, and Ref. [31] for Sb_80_F_401_^−^.

Because some of the anions discussed in this work exhibit large VDE values, it is useful to place these results in a broader context, that is, by comparing them with a set of other superhalogen anions arbitrarily selected from the existing literature. Such an indicative comparison is provided in Figure 11, where the most strongly electronically bound systems examined in the present study are positioned along a scale of increasing VDE. As can be seen, they rank relatively high, exceeding 12 eV, although it should be noted that the strongest currently known anions of this type can, in some cases, reach VDE values higher by almost 3 eV.

**Figure 12:**
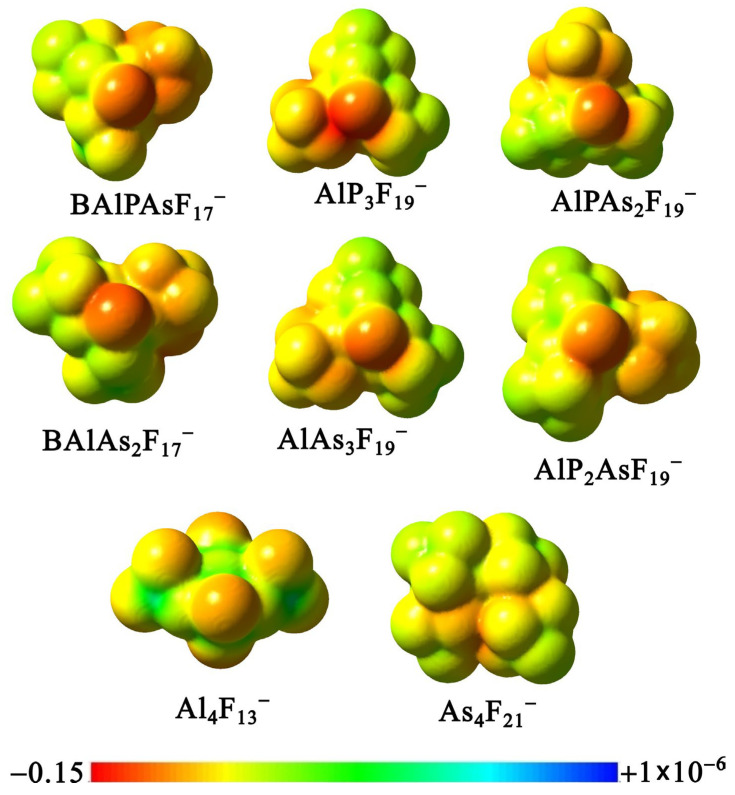
Molecular electrostatic potential (ESP) maps of selected (**X***_n_***Y***_n’_*F_{(3*n*+5*n*_*_’_*_)+1}_)^−^ anions (where *n* + *n’* = 4 and **X** = B and/or Al, **Y** = P and/or As) together with Al_4_F_13_^–^ and As_4_F_21_^–^ computed from MP2 electron densities and plotted on the 0.001 e/bohr^3^ isodensity surface.

The electronic and thermodynamic stability of these six anions, as established by our calculations, motivates an assessment of their potential as weakly coordinating anions. Figure 12 presents the electrostatic potential (ESP) maps for these species, arranged in order of increasing VDE, along with Al_4_F_13_^−^ and As_4_F_21_^−^, which exhibit the highest VDE values among the studied tetranuclear anions. Because the same potential scale is applied across all species and the number of fluorine atoms increases in the tetranuclear anions, the electrostatic potential becomes less negative (i.e., shifts toward the greener regions on this scale) relative to the di- and trinuclear anions. Nevertheless, the potential remains relatively inhomogeneous, with pronounced localization on the fluorine atom not bonded to any **X**F_3_ or **Y**F_5_ groups.

We also generated molecular electrostatic potential (ESP) maps for all remaining heterovalent tetranuclear anions, including those that are thermodynamically unstable. These ESP maps are presented in the Supplementary Materials (Figures S4 and S5), arranged in order of increasing VDE and corresponding to the molecular structures shown in Figures S1–S3 (in the Supplementary Materials). The maps clearly show that a more homogeneous potential distribution is observed in structures that do not contain aluminum.

## 3. Methods

The initial search for the low-energy isomeric structures of heterovalent (**X***_n_***Y***_n’_*F_{(3*n*+5*n’*)+1}_)^−^ anions (where *n* + *n’* = 2–4 and **X** = B and/or Al, **Y** = P and/or As) as well as for homovalent (**X***_n_*F_3*n*+1_)^−^ and (**Y***_n_*F_5*n*+1_)^−^ anions (*n* = 2–4) was carried out (for each *n* + *n’* and *n*) using the Coalescence Kick (CK) program [62,63] with the B3LYP [64,65] method and small split-valence 3–21G [66,67,68] basis set. The choice of the CK technique was dictated by the fact that numerous earlier reports have confirmed its usefulness and reliability (demonstrated by good agreement with experimental data) for studying the molecular structures of various cluster anions [62,69,70,71,72,73,74,75].

The lowest-energy isomers (with their relative energies (ΔE) within 20 kcal/mol) found by employing the CK-based search were then re-optimized, and frequencies were calculated at the second-order Møller–Plesset (MP2) [76,77,78] using the aug-cc-pVDZ [79,80] basis set. All MP2 calculations were performed using the frozen-core approximation, as is standard practice for systems of this size.

The Gibbs free reaction energies (at T = 298.15 K) for the fragmentation processes of the studied anions were calculated using the electronic energies, zero-point energy corrections, thermal corrections, and entropy contributions estimated at the MP2/aug-cc-pVDZ level of theory. We focused on the detachment of neutral XF_3_ and/or YF_5_ molecules from studied (**X***_n_***Y***_n’_*F_{(3*n*+5*n’*)+1}_)^−^, (**X***_n_*F_3*n*+1_)^−^, and (**Y***_n_*F_5*n*+1_)^−^ anions, as prior studies consistently demonstrate that these reactions are the least endergonic for polynuclear superhalogen anions [30,81,82]. Taking into account the overall calculated thermodynamic stability of (**X***_n_***Y***_n’_*F_{(3*n*+5*n’*)+1}_)^−^, (**X***_n_*F_3*n*+1_)^−^, and (**Y***_n_*F_5*n*+1_)^−^ anions, we decided to further present only those isomers whose relative energies did not exceed 10 kcal/mol.

Similar multistep approaches, involving CK(B3LYP/3-21G) pre-optimization followed by MP2/aug-cc-pVDZ calculations for final geometries and corresponding electronic and Gibbs free energies, have been successfully applied to superhalogen systems and related clusters (see, e.g., Refs. [30,31,82], and our previous work [83]). Benchmark tests using larger basis sets and/or higher-level methods consistently show very close agreement in optimized geometries and relative energies of isomers, making MP2/aug-cc-pVDZ a reliable and computationally efficient choice for determining equilibrium structures and harmonic vibrational frequencies of relatively large clusters

The relative energies of the isomers were determined with respect to the most stable isomer based solely on the electronic energies obtained at the MP2/aug-cc-pVDZ theory level (without including zero-point energy corrections). For selected representative isomers, single-point energies were also calculated at the CCSD(T)/aug-cc-pVTZ level to benchmark the MP2/aug-cc-pVDZ results, confirming that the relative energetic ordering and global minima are correctly reproduced.

The theoretical vertical electron detachment energies (VDE) of the most stable (**X***_n_***Y***_n’_*F_{(3*n*+5*n’*)+1}_)^−^, (**X***_n_*F_3*n*+1_)^−^, and (**Y***_n_*F_5*n*+1_)^−^ anions were calculated by applying the outer valence Green function (OVGF) method [84,85,86,87,88,89,90,91,92] and the same (aug-cc-pVDZ) basis set. Benchmark studies [30,93] confirm that this level of theory provides reliable electron-binding energies while enabling treatment of relatively large clusters. In addition, we performed calculations at the OVGF/aug-cc-pVTZ level for the selected most stable structures, and the differences in VDE values were found to be less than 0.05 eV, further justifying the use of OVGF/aug-cc-pVDZ for all clusters studied. During those calculations, all orbitals in the core and valence shells have been correlated. Since the quantitative measure of OVGF reliability is the pole strength (PS), which should be higher than 0.80 [94], we verified that for the studied anions, the PSs are close to 1 (i.e., 0.910–0.928), confirming that electron detachment is almost purely one-electron in character.

The Natural Bond Orbital analysis was performed with the Gaussian NBO 7 module [95].

All calculations were performed with the GAUSSIAN16 (Rev.C.02) package [96]. Molecular structure visualization was done using the Chemcraft [97], while electrostatic potential maps were generated using GaussView 6.0 [96].

## 4. Conclusions

On the basis of the MP2/aug-cc-pVDZ and OVGF(full)/aug-cc-pVDZ calculations performed for homovalent (**X***_n_*F_3*n*+1_)^−^ and (**Y***_n_*F_5*n*+1_)^−^ as well as heterovalent (**X***_n_***Y***_n’_*F_{(3*n*+5*n’*)+1}_)^−^ anions (where *n* + *n’* = 2–4 and **X** = B and/or Al, **Y** = P and/or As), we conclude the following:

- Homovalent polynuclear anions (**X***_n_*F_3*n*+1_)^−^ and (**Y***_n_*F_5*n*+1_)^−^ exhibit high electronic stability, with vertical electron detachment energies (VDEs) ranging from 10.70 to 12.41 eV. Electronic stability generally increases with the number of central atoms; however, aluminum-based clusters are particularly notable for their compact geometries (such as the eight-membered ring in Al_4_F_13_^−^), which provide high thermodynamic stability. In contrast, larger purely non-metallic phosphorus and arsenic clusters are more prone to fragmentation.
- Heterovalent dinuclear anions (BPF_9_^−^, AlPF_9_^−^, BAsF_9_^−^, and AlAsF_9_^−^) are all thermodynamically and electronically (VDE = 10.7–11.3 eV) stable and feature a single fluorine connecting **X**F_3_ and **Y**F_5_ units. In boron-based frameworks, heterovalent arrangements (e.g., B**Y**F_9_^−^) promote a more uniform charge distribution, enhancing their character as weakly coordinating anions (WCAs).
- Heterovalent trinuclear anions reach even higher electronic stabilities, with AlAs_2_F_14_^−^ reaching a VDE of 12.37 eV, exceeding that of any homovalent dinuclear analog. Purely non-metallic trinuclear heterovalent anions are marginally stable or unstable, although the presence of mixed valency still favors a more homogeneous electrostatic potential distribution in boron-containing species. In aluminum-containing anions, Al serves as a structural “glue”, ensuring thermodynamic stability for any cluster containing at least one Al atom.
- Heterovalent tetranuclear anions face increasing challenges in maintaining structural integrity. Most heterovalent boron-based species exist as complexes (e.g., a BF_4_^−^ core solvated by PF_5_ molecules) rather than fully integrated frameworks. Thermodynamic stability is observed only in aluminum-containing species, where the octahedral coordination of Al is crucial for stabilizing the larger cluster. Although the results indicate thermodynamic stability, its magnitude is small (1.45–2.90 kcal/mol); therefore, the conclusions should be interpreted with appropriate caution, given the limited accuracy of the computational method, which does not allow for a definitive assessment in such borderline cases. While twelve of these heterovalent anions exhibit VDEs above 12 eV, none surpass the electronic stability of homovalent counterparts such as Al_4_F_13_^−^ or As_4_F_21_^−^.

Overall, these results demonstrate that systematic introduction of heterovalent central atoms in polynuclear superhalogen anions provides a viable strategy to tune both electronic and thermodynamic stability, offering insights into the design of weakly coordinating anions and high-stability superhalogen species for potential applications in superacid chemistry, catalysis, and materials science. From an application-oriented perspective, the most stable members of the present superhalogen family may be particularly attractive as weakly coordinating anions for isolating and handling highly reactive cations. Their enhanced electronic and thermodynamic robustness suggests improved resistance toward decomposition under strongly oxidizing or highly acidic conditions, which is desirable for superacid media, for stabilizing carbocations, silylium-type species, and other electrophilic intermediates, and for supporting highly active homogeneous catalysts without direct anion participation. Moreover, such robust WCAs could serve as counterions in ionic liquids or low-volatility salts designed for harsh operating windows, where thermal and chemical stability are critical. Finally, the demonstrated ability to tune stability through heterovalent central-atom substitution provides a practical design handle for targeted optimization of counterion properties, encouraging future experimental synthesis and evaluation in superacid chemistry, catalysis, and functional materials.

## Data Availability

The original contributions presented in this study are included in the article. Further inquiries can be directed to the corresponding authors.

## Supplementary Materials

The following supporting information can be downloaded at: https://www.mdpi.com/article/10.3390/molecules31060933/s1. Table S1: Cartesian coordinates (in Å) of the isomeric structures of the (**X***_n_*F_(3*n*+1)_)^−^, (**Y***_n_*F_(5*n*+1)_)^−^ and (**X***_n_***Y***_n’_*F_{(3*n*+5*n’*)+1}_)^−^ (*n* + *n’* = 1–4 and **X** = B and/or Al, **Y** = P and/or As), determined at the MP2/aug-cc-pVDZ theory level. The ΔE (in kcal/mol) stands for relative energies for presented isomers with respect to their corresponding global minima; Table S2: The vertical electron detachment energies (VDE in eV) calculated with OVGF(full) method using the aug-cc-pVDZ basis set characterizing the most stable (**X***_n_*F_(3*n*+1)_)^−^, (**Y***_n_*F_(5*n*+1)_)^−^ and (**X***_n_***Y***_n’_*F_{(3*n*+5*n’*)+1}_)^−^ (*n* + *n’* = 1–4 and **X** = B and/or Al, **Y** = P and/or As) anions; Table S3: NBO atomic charges, Wiberg bond indices, and bonding orbital compositions for the studied **X***_n_***Y***_n’_*F_{(3*n*+5*n’*)+1}_)^−^ (*n* + *n’* = 1–4 and **X** = B and/or Al, **Y** = P and/or As) anions calculated at the MP2/aug-cc-pvdz level; Table S4: Gibbs free energies (ΔG_r_^298^ in kcal/mol; for T = 298.15 K) and electronic energies (ΔE in kcal/mol) predicted for the fragmentation processes of (**X***_n_*F_(3*n*+1)_)^−^, (**Y***_n_*F_(5*n*+1)_)^−^ and (**X***_n_***Y***_n’_*F_{(3*n*+5*n’*)+1}_)^−^ (*n* + *n’* = 1–4 and **X** = B and/or Al, **Y** = P and/or As) anions obtained at MP2/aug-cc-pVDZ level; Figure S1: The equilibrium structures of (**X**_3_**Y**F_15_)^−^ anions (where **X** = B and/or Al, **Y** = P or As) obtained at MP2/aug-cc-pVDZ level; Figure S2: The equilibrium structures of (**X**_2_**Y**_2_F_17_)^−^ anions (where **X** = B and/or Al, **Y** = P and/or As) obtained at MP2/aug-cc-pVDZ level; Figure S3. The equilibrium structures of (**XY**_3_F_19_)^−^ anions (where **X** = B or Al, **Y** = P and/or As) obtained at MP2/aug-cc-pVDZ level; Figure S4: Molecular electrostatic potential (ESP) maps **X**_4_F_13_^−^, **Y**_4_F_21_^−^, and (**X***_n_***Y***_n’_*F_{(3*n*+5*n’*)+1}_)^−^ anions (where *n* + *n’* = 4 and **X** = B and/or Al, **Y** = P and/or As) computed from MP2 electron densities, plotted on the 0.001 e/bohr^3^ isodensity surface, and arranged in order of increasing VDE. Visualization performed on the same scale as in Figure 5 (i.e., from −0.15 to +1 × 10^−6^ a.u.); Figure S5: Molecular electrostatic potential (ESP) maps **X**_4_F_13_^−^, **Y**_4_F_21_^−^, and (**X***_n_***Y***_n’_*F_{(3*n*+5*n’*)+1}_)^−^ anions (where *n* + *n’* = 4 and **X** = B and/or Al, **Y** = P and/or As) computed from MP2 electron densities, plotted on the 0.001 e/bohr^3^ isodensity surface, and arranged in order of increasing VDE (continuation). Visualization performed on the same scale as in Figure 5 (i.e., from −0.15 to +1 × 10^−6^ a.u.).
