# Supplementary material for: Polynuclear Superhalogen Anions with Heterovalent Central Atoms
Source: Molecules. 2026 Mar 11;31(6):933. doi: 10.3390/molecules31060933 (PMC13029721; doi:10.3390/molecules31060933)
Supplement: Supplementary file 1 [file molecules-31-00933-s001.zip › molecules-4166363-supplementary.pdf]

Supplementary Materials

Polynuclear Superhalogen Anions with Heterovalent Central Atoms.

David Mekhael[1], Piotr Skurski[1,2], Iwona Anusiewicz[1,*]

[1] *Laboratory of Quantum Chemistry, Faculty of Chemistry, University of Gdańsk, Wita Stwosza 63, 80-308 Gdańsk, Poland*

[2] *Department of Chemistry, University of Utah, Salt Lake City, Utah 84112, U.S.A.*

[*] corresponding author: iwona.anusiewicz@ug.edu.pl

Table of contents

Table S1. Cartesian coordinates (in Å) of the isomeric structures of the $(X_nF_{(3n+1)})^-$, $(Y_nF_{(5n+1)})^-$ and $(X_nY_{n'}F_{\{(3n+5n')+1\}})^-$ (*n*+*n'*=1-4 and X=B and/or Al, Y=P and/or As) determined at the MP2/aug-cc-pVDZ theory level. The ΔE (in kcal/mol) stands for relative energies for presented isomers with respect to their corresponding global minima....................3

Table S2. The vertical electron detachment energies (VDE in eV) calculated with OVGF(full) method using the aug-cc-pVDZ basis set characterizing the most stable $(X_nF_{(3n+1)})^-$, $(Y_nF_{(5n+1)})^-$ and $(X_nY_{n'}F_{\{(3n+5n')+1\}})^-$ (*n*+*n'*=1-4 and X=B and/or Al, Y=P and/or As) anions. 145

Table S3. NBO atomic charges, Wiberg bond indices, and bonding orbital compositions for the studied $(X_nY_{n'}F_{\{(3n+5n')+1\}})^-$ (*n*+*n'*=1-4 and X=B and/or Al, Y=P and/or As) anions calculated at the MP2/aug-cc-pvdz level....................147

Table S4. Gibbs free energies (ΔG_r^{298} in kcal/mol; for T=298.15 K) and electronic energies (ΔE in kcal/mol) predicted for the fragmentation processes of $(X_nF_{(3n+1)})^-$, $(Y_nF_{(5n+1)})^-$ and $(X_nY_{n'}F_{\{(3n+5n')+1\}})^-$ (*n*+*n'*=1-4 and X=B and/or Al, Y=P and/or As) anions obtained at MP2/aug-cc-pVDZ level. 173

Figure S1. The equilibrium structures of $(\mathbf{X}_3\mathbf{Y}F_{15})^-$ anions (where **X**=B and/or Al, **Y**=P or As) obtained at MP2/aug-cc-pVDZ level. 179

Figure S2. The equilibrium structures of $(\mathbf{X}_2\mathbf{Y}_2F_{17})^-$ anions (where **X**=B and/or Al, **Y**=P and/or As) obtained at MP2/aug-cc-pVDZ level. 180

Figure S3. The equilibrium structures of $(\mathbf{X}\mathbf{Y}_3F_{19})^-$ anions (where **X**=B or Al, **Y**=P and/or As) obtained at MP2/aug-cc-pVDZ level. 181

Figure S4. Molecular electrostatic potential (ESP) maps $\mathbf{X}_4F_{13}^-$, $\mathbf{Y}_4F_{21}^-$, and $(\mathbf{X}_n\mathbf{Y}_{n'}F_{\{(3n+5n')+1\}})^-$ anions (where n+n'=4 and **X**=B and/or Al, **Y**=P and/or As) computed from MP2 electron densities and plotted on the 0.001 e/bohr^3 isodensity surface and arranged in order of increasing VDE. Visualization performed on the same scale as in Figure 5 (i.e., from −0.15 to +1 × 10^{-6} a.u.). 182

Figure S5. Molecular electrostatic potential (ESP) maps $\mathbf{X}_4F_{13}^-$, $\mathbf{Y}_4F_{21}^-$, and $(\mathbf{X}_n\mathbf{Y}_{n'}F_{\{(3n+5n')+1\}})^-$ anions (where n+n'=4 and **X**=B and/or Al, **Y**=P and/or As) computed from MP2 electron densities and plotted on the 0.001 e/bohr^3 isodensity surface and arranged in order of increasing VDE (continuation). Visualization performed on the same scale as in Figure 5 (i.e., from −0.15 to +1 × 10^{-6} a.u.)....................183

Table S1. Cartesian coordinates (in Å) of the isomeric structures of the $(X_nF_{(3n+1)})^-$, $(Y_nF_{(5n+1)})^-$ and $(X_nY_{n'}F_{\{(3n+5n')+1\}})^-$ ($n+n'$=1-4 and X=B and/or Al, Y=P and/or As) determined at the MP2/aug-cc-pVDZ theory level. The ΔE (in kcal/mol) stands for relative energies for presented isomers with respect to their corresponding global minima.

Formula	structure / Cartesian coordinates	ΔE
BF_4^-	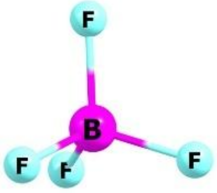	**0.0**
	B 0.000000000 0.000000000 0.000000000<br>F -0.826510000 0.826510000 -0.826510000<br>F 0.826510000 -0.826510000 -0.826510000<br>F 0.826510000 0.826510000 0.826510000<br>F -0.826510000 -0.826510000 0.826510000	
AlF_4^-	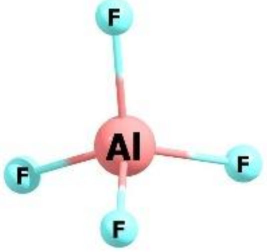	**0.0**
	Al 0.000000000 0.000000000 0.000000000<br>F -0.996455000 0.996455000 -0.996455000<br>F 0.996455000 -0.996455000 -0.996455000<br>F 0.996455000 0.996455000 0.996455000<br>F -0.996455000 -0.996455000 0.996455000	
PF_6^-	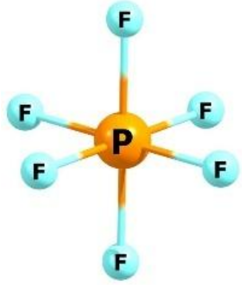	**0.0**
	P 0.000000000 0.000000000 0.000000000<br>F 0.000000000 0.000000000 1.658979000<br>F 0.000000000 1.658979000 0.000000000<br>F 0.000000000 0.000000000 -1.658979000<br>F 1.658979000 0.000000000 0.000000000<br>F -1.658979000 0.000000000 0.000000000<br>F 0.000000000 -1.658979000 0.000000000	

AsF_6^-	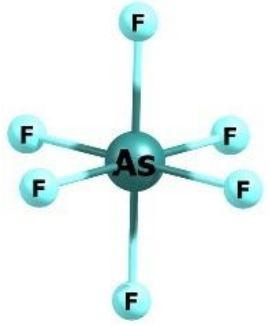	**0.0**
	As 0.000000000 0.000000000 0.000000000<br>F 0.000000000 0.000000000 1.763877000<br>F 0.000000000 1.763877000 0.000000000<br>F 0.000000000 0.000000000 -1.763877000<br>F 1.763877000 0.000000000 0.000000000<br>F -1.763877000 0.000000000 0.000000000<br>F 0.000000000 -1.763877000 0.000000000	
$B_2F_7^-$	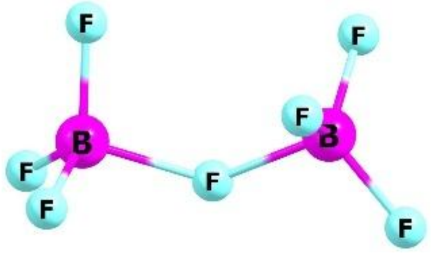	**0.0**
	B -1.431011000 0.001982000 -0.012142000<br>B 1.431694000 -0.001809000 -0.011933000<br>F -2.244327000 0.534024000 -1.012903000<br>F 2.242771000 -0.548545000 -1.006624000<br>F -0.000213000 -0.003832000 -0.708974000<br>F -1.703118000 -1.331571000 0.268208000<br>F 1.311262000 -0.797518000 1.116581000<br>F -1.311286000 0.812545000 1.106337000<br>F 1.704531000 1.334801000 0.250751000	
$Al_2F_7^-$	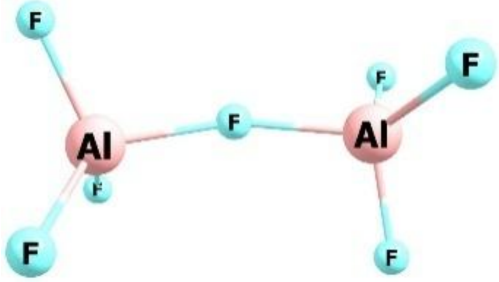	**0.0**
	Al -1.799613000 -0.000475000 -0.006196000<br>Al 1.799519000 0.000476000 -0.006601000<br>F -2.406673000 -1.031335000 -1.213736000<br>F 2.191351000 -1.619161000 0.324478000<br>F -0.000070000 -0.000267000 -0.287127000<br>F -2.008143000 -0.593386000 1.571351000<br>F 2.047991000 1.054800000 1.301394000<br>F -2.266209000 1.621542000 -0.203131000<br>F 2.441889000 0.567806000 -1.474744000	

$P_2F_{11}^-$	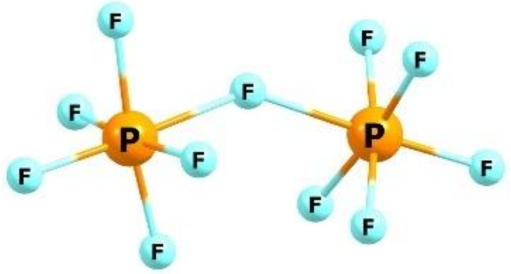	**0.0**

P	0.000000000	1.757467000	0.002212000
P	0.000000000	-1.757467000	0.002212000
F	1.479289000	-1.860117000	0.687605000
F	0.003527000	3.316740000	-0.469726000
F	1.479925000	1.456312000	-0.608193000
F	0.676390000	2.089941000	1.452379000
F	0.681456000	-1.225518000	-1.373624000
F	0.000000000	0.000000000	0.615743000
F	-0.681456000	1.225518000	-1.373624000
F	-1.479925000	-1.456312000	-0.608193000
F	-1.479289000	1.860117000	0.687605000
F	-0.003527000	-3.316740000	-0.469726000
F	-0.676390000	-2.089941000	1.452379000

$As_2F_{11}^-$	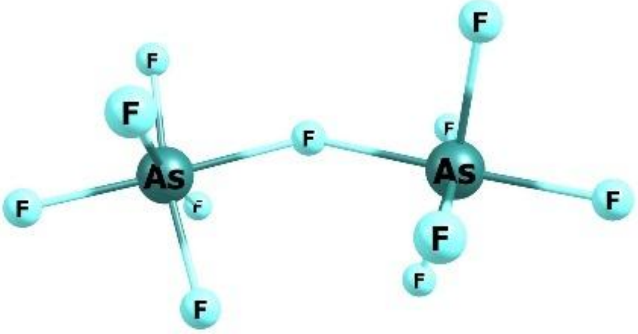	**0.0**

As	0.000000000	1.832768000	0.002703000
As	0.000000000	-1.832768000	0.002703000
F	1.577736000	-1.939019000	0.731954000
F	0.003336000	3.495915000	-0.500003000
F	1.580217000	1.511990000	-0.644323000
F	0.717700000	2.185077000	1.550701000
F	0.724639000	-1.262839000	-1.466137000
F	0.000000000	0.000000000	0.635797000
F	-0.724639000	1.262839000	-1.466137000
F	-1.580217000	-1.511990000	-0.644323000
F	-1.577736000	1.939019000	0.731954000
F	-0.003336000	-3.495915000	-0.500003000
F	-0.717700000	-2.185077000	1.550701000

$B_3F_{10}^-$	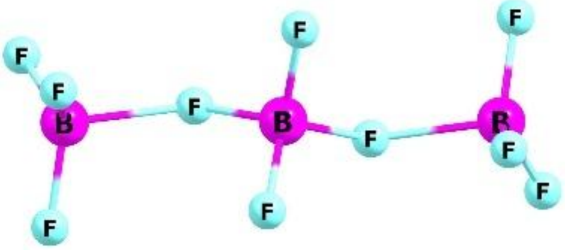	**0.0**

B	0.000000000	2.783990000	-0.218987000
B	0.000000000	-2.783990000	-0.218987000

	B	0.000000000	0.000000000	0.395476000	
	F	-0.210644000	-1.124726000	-0.575290000	
	F	-0.377720000	-2.865615000	1.097524000	
	F	1.333531000	-2.973720000	-0.485043000	
	F	0.377720000	2.865615000	1.097524000	
	F	0.210644000	1.124726000	-0.575290000	
	F	0.872212000	3.328001000	-1.134455000	
	F	-0.872212000	-3.328001000	-1.134455000	
	F	-1.151100000	0.210501000	1.109070000	
	F	-1.333531000	2.973720000	-0.485043000	
	F	1.151100000	-0.210501000	1.109070000	
**$B_3F_{10}^-$**	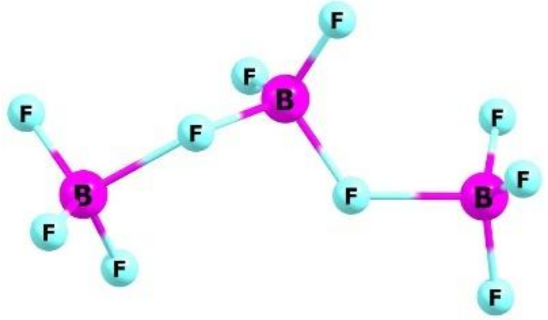				**0.2**
	B	-2.570645000	-0.408226000	-0.007968000	
	B	2.587125000	-0.417432000	-0.008797000	
	B	-0.005594000	0.817295000	0.042130000	
	F	1.233368000	0.327954000	0.729263000	
	F	3.252969000	0.652318000	-0.554212000	
	F	2.012291000	-1.296559000	-0.889650000	
	F	-2.852611000	0.662249000	-0.819191000	
	F	-0.855161000	-0.410750000	0.031497000	
	F	-2.789010000	-1.653126000	-0.548724000	
	F	3.169952000	-0.972784000	1.108233000	
	F	-0.569925000	1.772708000	0.853443000	
	F	-2.893593000	-0.270446000	1.318990000	
	F	0.285673000	1.193082000	-1.243741000	
**$Al_3F_{10}^-$**	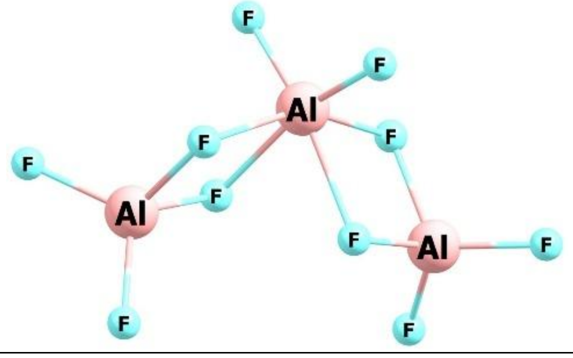				**0.0**
	Al	0.000003000	1.159034000	0.000000000	
	Al	-2.257734000	-0.636664000	0.085967000	
	Al	2.257731000	-0.636676000	-0.085975000	
	F	0.764131000	2.168361000	1.151469000	
	F	-3.728971000	-0.258052000	-0.648349000	
	F	-0.764132000	2.168321000	-1.151505000	
	F	-1.574084000	0.709652000	1.034187000	
	F	3.729019000	-0.258080000	0.648248000	
	F	-2.289430000	-2.079890000	0.961317000	

	F	-0.849186000	-0.457506000	-0.955654000	
	F	2.289312000	-2.079958000	-0.961233000	
	F	0.849265000	-0.457357000	0.955738000	
	F	1.574075000	0.709617000	-1.034208000	
	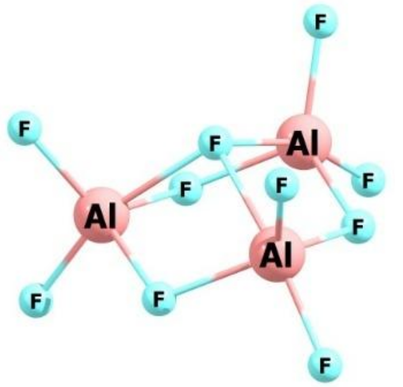				**0.3**
	Al	0.000000000	1.780093000	0.000920000	
	Al	1.541606000	-0.890047000	0.000920000	
	Al	-1.541606000	-0.890047000	0.000920000	
	F	-2.255041000	-1.301949000	1.480754000	
	F	-2.539936000	-1.466433000	-1.255142000	
	F	0.000000000	0.000000000	0.973530000	
	F	0.000000000	-1.749077000	-0.551450000	
**$Al_3F_{10}^-$**	F	0.000000000	2.932866000	-1.255142000	
	F	2.539936000	-1.466433000	-1.255142000	
	F	0.000000000	2.603897000	1.480754000	
	F	2.255041000	-1.301949000	1.480754000	
	F	1.514745000	0.874538000	-0.551450000	
	F	-1.514745000	0.874538000	-0.551450000	
	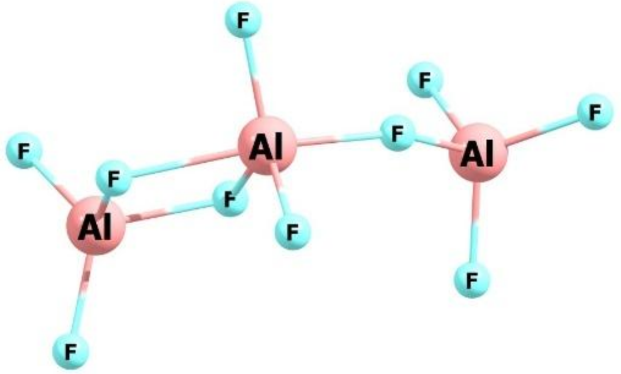				**0.9**
	Al	-0.520319000	-2.664176000	0.000000000	
	Al	-0.727681000	2.477829000	0.000000000	
	Al	1.244982000	0.354684000	0.000000000	
	F	2.022970000	0.420075000	1.500563000	
	F	1.027260000	2.399070000	0.000000000	
	F	0.281704000	-4.162470000	0.000000000	
	F	2.022970000	0.420075000	-1.500563000	
	F	-1.392788000	-2.348739000	1.423409000	
	F	-1.392788000	3.071341000	-1.426511000	
	F	-1.392788000	3.071341000	1.426511000	
	F	-0.606097000	0.676252000	0.000000000	
	F	-1.392788000	-2.348739000	-1.423409000	
	F	0.826702000	-1.441358000	0.000000000	

$Al_3F_{10}^-$	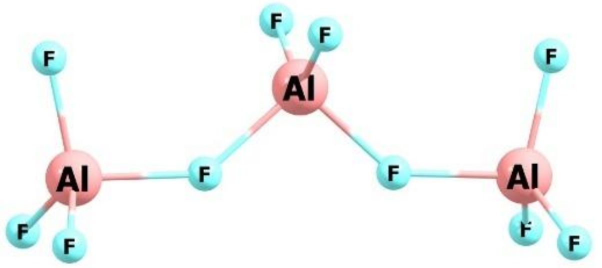				**2.2**
	Al	3.190137000	-0.418699000	0.000024000	
	Al	-0.000218000	0.821038000	0.000009000	
	Al	-3.190029000	-0.418681000	0.000023000	
	F	-0.000433000	1.644770000	-1.461450000	
	F	3.570229000	-1.250879000	-1.424207000	
	F	3.557039000	1.235686000	0.000386000	
	F	-0.000158000	1.645590000	1.461007000	
	F	3.570169000	-1.251710000	1.423786000	
	F	-3.555723000	1.235973000	0.000096000	
	F	-3.570307000	-1.250957000	-1.424097000	
	F	-3.570817000	-1.251358000	1.423769000	
	F	1.337873000	-0.366418000	0.000204000	
	F	-1.337713000	-0.367093000	0.000424000	
	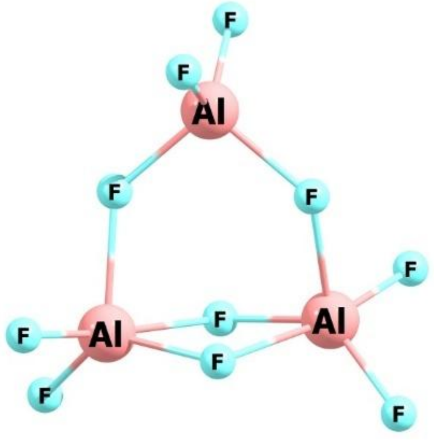				**4.1**
	Al	-1.493293000	0.155720000	-0.967251000	
	Al	1.493293000	-0.155720000	-0.967251000	
	Al	0.000000000	0.000000000	2.010516000	
	F	-0.291796000	1.398687000	2.899994000	
	F	0.000000000	1.151553000	-1.151177000	
	F	1.322301000	0.322834000	0.861819000	
	F	0.291796000	-1.398687000	2.899994000	
	F	2.569333000	-1.446582000	-0.626088000	
	F	-2.379393000	-0.827713000	-2.039447000	
	F	-1.322301000	-0.322834000	0.861819000	
	F	-2.569333000	1.446582000	-0.626088000	
	F	2.379393000	0.827713000	-2.039447000	
	F	0.000000000	-1.151553000	-1.151177000	

Species	Atom	x	y	z	
$Al_3F_{10}^-$	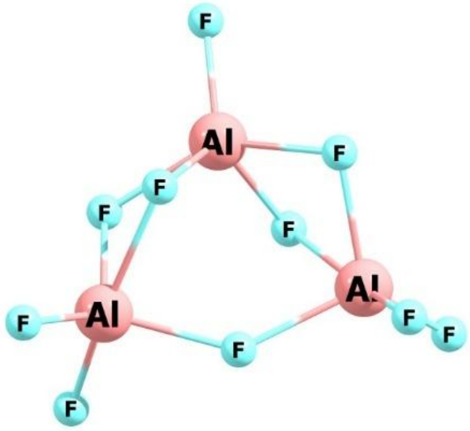				**7.0**
	Al	-1.739044000	-0.684727000	0.013595000	
	Al	1.738974000	-0.684805000	-0.013598000	
	Al	0.000084000	1.540137000	0.000025000	
	F	-0.000056000	-1.367322000	-0.000023000	
	F	-0.842101000	0.610049000	-1.273608000	
	F	-2.281294000	-1.552574000	1.382211000	
	F	0.000155000	3.223983000	0.000126000	
	F	2.827216000	-1.073017000	1.233070000	
	F	0.842167000	0.609777000	1.273575000	
	F	-1.586810000	0.964076000	0.864230000	
	F	1.586880000	0.964017000	-0.864283000	
	F	-2.827342000	-1.072792000	-1.233078000	
	F	2.281164000	-1.552627000	-1.382252000	
$P_3F_{16}^-$	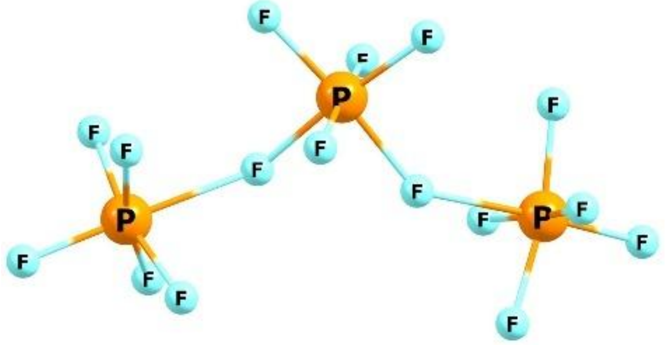				**0.0**
	Al	-1.739044000	-0.684727000	0.013595000	
	Al	1.738974000	-0.684805000	-0.013598000	
	Al	0.000084000	1.540137000	0.000025000	
	F	-0.000056000	-1.367322000	-0.000023000	
	F	-0.842101000	0.610049000	-1.273608000	
	F	-2.281294000	-1.552574000	1.382211000	
	F	0.000155000	3.223983000	0.000126000	
	F	2.827216000	-1.073017000	1.233070000	
	F	0.842167000	0.609777000	1.273575000	
	F	-1.586810000	0.964076000	0.864230000	
	F	1.586880000	0.964017000	-0.864283000	
	F	-2.827342000	-1.072792000	-1.233078000	
	F	2.281164000	-1.552627000	-1.382252000	

$P_3F_{16}^-$	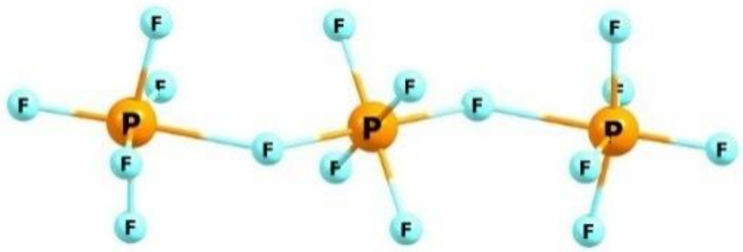 	**0.2**
	Al -1.739044000 -0.684727000 0.013595000<br>Al 1.738974000 -0.684805000 -0.013598000<br>Al 0.000084000 1.540137000 0.000025000<br>F -0.000056000 -1.367322000 -0.000023000<br>F -0.842101000 0.610049000 -1.273608000<br>F -2.281294000 -1.552574000 1.382211000<br>F 0.000155000 3.223983000 0.000126000<br>F 2.827216000 -1.073017000 1.233070000<br>F 0.842167000 0.609777000 1.273575000<br>F -1.586810000 0.964076000 0.864230000<br>F 1.586880000 0.964017000 -0.864283000<br>F -2.827342000 -1.072792000 -1.233078000<br>F 2.281164000 -1.552627000 -1.382252000	
$As_3F_{16}^-$	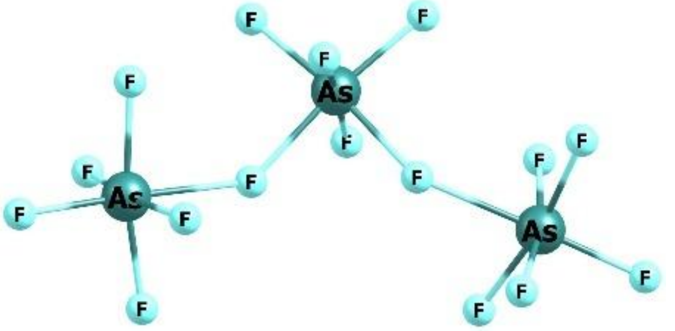 	**0.0**
	As 0.000000000 3.103160000 -0.639220000<br>As 0.000000000 0.000000000 1.293259000<br>As 0.000000000 -3.103160000 -0.639220000<br>F -1.441543000 -3.449349000 0.261236000<br>F 0.267546000 -4.731476000 -1.147880000<br>F 0.362524000 1.178588000 -0.106087000<br>F -1.653700000 0.461306000 1.144208000<br>F 1.653700000 -0.461306000 1.144208000<br>F 1.441543000 3.449349000 0.261236000<br>F -0.362524000 -1.178588000 -0.106087000<br>F 1.387120000 -2.448280000 -1.445126000<br>F 0.978557000 2.669311000 -2.003304000<br>F -1.387120000 2.448280000 -1.445126000<br>F 0.922851000 -3.232680000 0.818773000<br>F -0.978557000 -2.669311000 -2.003304000<br>F -0.922851000 3.232680000 0.818773000<br>F -0.267546000 4.731476000 -1.147880000<br>F -0.350879000 -1.226102000 2.451014000<br>F 0.350879000 1.226102000 2.451014000	

$Al_3F_{10}^-$	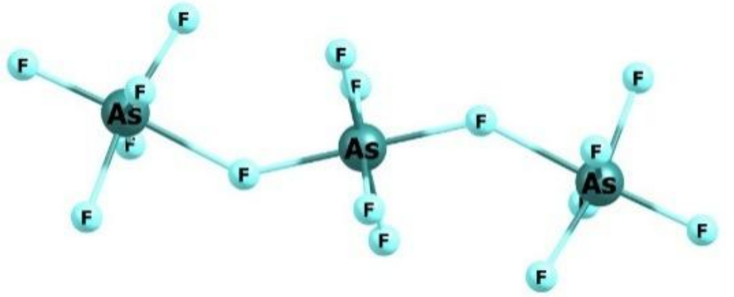	**0.3**

As	1.208878000	-3.447055000	0.020259000
As	0.000000000	0.000000000	0.000000000
F	1.108562000	-1.441040000	0.349644000
F	-0.057886000	0.329862000	1.694972000
F	0.057886000	-0.329862000	-1.694972000
F	-0.052884000	-3.212121000	-1.138030000
F	1.406337000	0.990668000	-0.148908000
F	2.376924000	-3.010596000	-1.185036000
F	2.452252000	-3.350373000	1.226628000
F	0.022533000	-3.556509000	1.278114000
F	1.351809000	-5.149769000	-0.221762000
F	-1.108562000	1.441040000	-0.349644000
F	-1.406337000	-0.990668000	0.148908000
As	-1.208878000	3.447055000	-0.020259000
F	-2.376924000	3.010596000	1.185036000
F	-2.452252000	3.350373000	-1.226628000
F	-0.022533000	3.556509000	-1.278114000
F	0.052884000	3.212121000	1.138030000
F	-1.351809000	5.149769000	0.221762000

$B_4F_{13}^-$	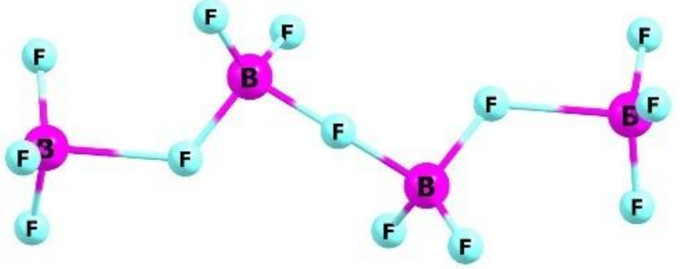	**0.0**

B	1.195557000	-0.731984000	0.213228000
B	-1.195718000	0.732915000	0.212763000
B	-3.955225000	-0.222471000	-0.202804000
B	3.955351000	0.221576000	-0.202903000
F	0.687649000	-1.339741000	-0.901377000
F	-4.250381000	-0.357491000	1.115066000
F	4.151516000	1.298487000	-1.008101000
F	4.046798000	-1.015197000	-0.757060000
F	1.716344000	-1.542954000	1.188008000
F	0.000094000	0.000762000	0.931433000
F	-4.047503000	1.014028000	-0.757488000
F	4.250387000	0.356954000	1.114937000
F	2.069446000	0.369881000	-0.107249000
F	-0.687966000	1.339872000	-0.902299000
F	-1.716350000	1.544505000	1.187096000
F	-4.150571000	-1.299836000	-1.007576000
F	-2.069446000	-0.369290000	-0.106659000

$B_4F_{13}^-$

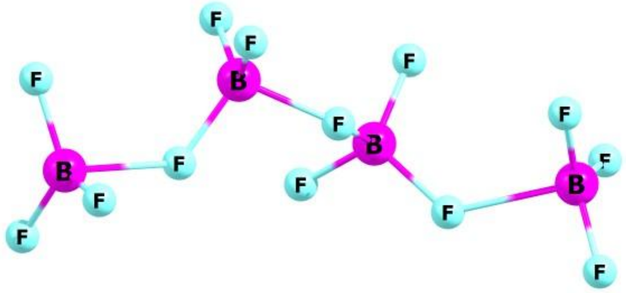

0.05

B	1.246526000	-0.748005000	-0.574227000
B	-3.773892000	0.576050000	-0.293716000
B	3.760711000	0.686342000	0.151921000
B	-1.211922000	-0.459825000	0.701305000
F	2.193022000	0.308162000	-0.852285000
F	0.264230000	0.000258000	0.400395000
F	3.239890000	0.634023000	1.402351000
F	-1.502805000	0.117311000	1.908786000
F	-4.020367000	0.502448000	-1.628480000
F	-1.937476000	0.153571000	-0.386390000
F	3.986243000	1.909929000	-0.397652000
F	-1.230391000	-1.825663000	0.636317000
F	-4.194727000	-0.451071000	0.490292000
F	-3.684875000	1.810853000	0.263582000
F	0.532172000	-1.071665000	-1.696713000
F	1.804527000	-1.774696000	0.136276000
F	4.538656000	-0.343772000	-0.268303000

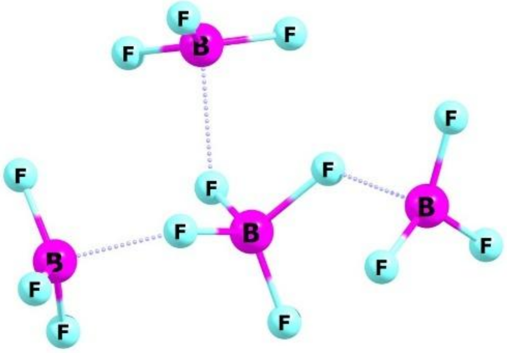

0.1

B	-0.195742000	-0.271456000	-0.621889000
B	2.205499000	-1.674995000	0.204374000
B	0.982962000	2.476380000	0.180859000
B	-2.956250000	-0.448882000	0.263016000
F	-2.613883000	-1.758118000	0.430066000
F	-1.325215000	0.310103000	0.106869000
F	-3.515675000	-0.090915000	-0.928232000
F	-0.612087000	-0.956937000	-1.732395000
F	2.824304000	-0.621420000	0.804026000
F	0.465237000	-1.146712000	0.339195000
F	2.207294000	2.730474000	-0.331917000
F	0.882810000	1.897022000	1.393181000
F	2.325124000	-1.788412000	-1.148765000
F	0.684211000	0.802167000	-0.939165000
F	-3.372735000	0.235065000	1.369813000
F	-0.070460000	3.172043000	-0.302487000
F	2.100814000	-2.829387000	0.925165000

$B_4F_{13}^-$

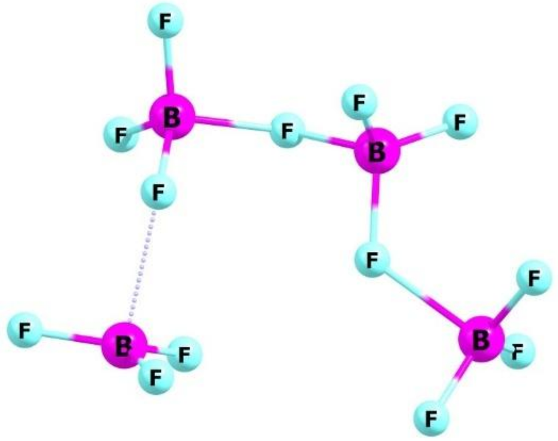

0.4

B	1.926877000	-1.244140000	0.046799000
B	-2.818264000	0.791539000	0.052397000
B	-0.878988000	-1.364254000	-0.070511000
B	1.817253000	1.913552000	-0.005564000
F	1.748056000	-0.088523000	-0.743094000
F	0.471691000	-1.499083000	0.680304000
F	1.145206000	2.403224000	-1.062797000
F	1.173787000	1.670496000	1.147870000
F	-1.765983000	-2.107976000	0.665917000
F	-1.142396000	0.062106000	0.042693000
F	2.211570000	-2.368068000	-0.688447000
F	2.705980000	-1.041737000	1.161835000
F	-0.681145000	-1.739705000	-1.371946000
F	-2.476061000	2.043347000	-0.361288000
F	-3.142220000	0.635759000	1.366279000
F	-3.440004000	-0.021506000	-0.849244000
F	3.165478000	1.997945000	-0.000926000

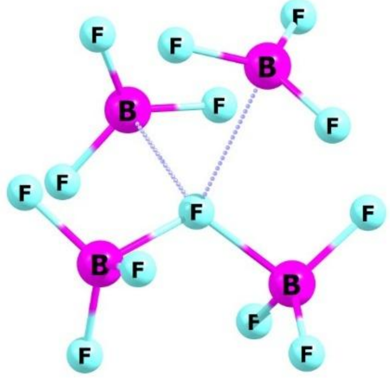

7.2

B	1.878795000	1.346354000	0.050262000
B	-1.937211000	1.270665000	-0.051483000
B	-0.035514000	-1.181415000	1.451640000
B	0.087665000	-1.180899000	-1.450965000
F	1.816395000	1.516669000	-1.276340000
F	2.757138000	0.484357000	0.573529000
F	0.012280000	-0.344668000	0.000632000
F	-1.370975000	-1.495570000	1.582903000
F	-1.880999000	1.444276000	1.274844000
F	-0.414699000	-0.237068000	-2.326052000
F	-2.777492000	0.371001000	-0.573572000
F	1.190867000	2.173253000	0.849892000

Species	Structure / Atom	x	y	z	Energy
	F	1.433924000	-1.442024000	-1.586524000	
	F	0.425607000	-0.218085000	2.328279000	
	F	-0.719767000	-2.274800000	-1.245784000	
	F	0.815268000	-2.242910000	1.250778000	
	F	-1.284066000	2.124067000	-0.852282000	
$Al_4F_{13}^-$	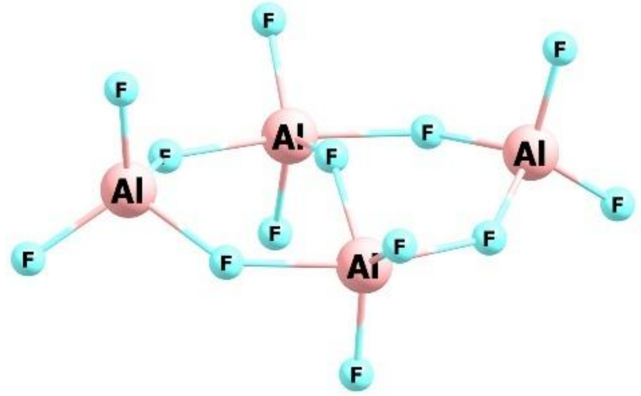				**0.0**
	Al	0.238745000	0.000056000	1.697001000	
	Al	0.238745000	0.000056000	-1.697001000	
	Al	-0.242678000	-2.933372000	0.000000000	
	Al	-0.242617000	2.933164000	0.000000000	
	F	-0.869776000	0.000283000	-2.969356000	
	F	-0.869776000	0.000283000	2.969356000	
	F	-1.908045000	3.193946000	0.000000000	
	F	-1.908017000	-3.194784000	0.000000000	
	F	-0.491165000	0.000231000	0.000000000	
	F	0.181826000	1.915385000	1.393064000	
	F	0.180906000	-1.915253000	-1.393078000	
	F	0.180906000	-1.915253000	1.393078000	
	F	0.181826000	1.915385000	-1.393064000	
	F	0.744142000	4.299669000	0.000000000	
	F	0.745083000	-4.299128000	0.000000000	
	F	1.921683000	-0.000313000	1.848099000	
	F	1.921683000	-0.000313000	-1.848099000	
	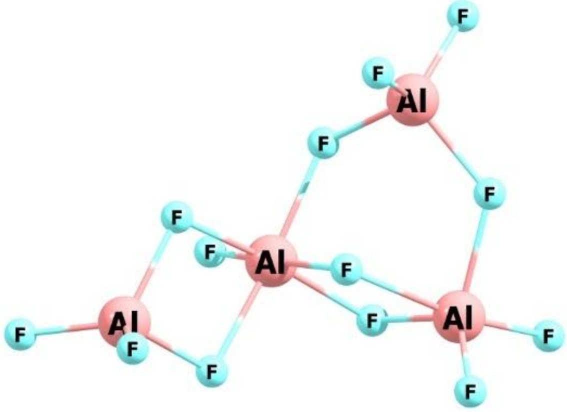				**0.7**
	Al	0.589662000	-0.078359000	0.865708000	
	Al	-1.571161000	1.668401000	-0.153762000	
	Al	-2.086236000	-1.568043000	-0.324521000	
	Al	3.126259000	-0.094025000	-0.407657000	
	F	-0.190399000	0.376331000	-0.744428000	
	F	-0.775996000	-1.415193000	0.873576000	
	F	3.365192000	0.235556000	-2.038549000	
	F	1.787649000	-1.239917000	-0.108248000	

$Al_4F_{13}^-$	F	1.183034000	-0.477084000	2.411300000	
	F	-0.554059000	1.309671000	1.345154000	
	F	-3.342063000	-2.417090000	0.414735000	
	F	4.517291000	-0.462188000	0.464432000	
	F	2.073736000	1.090655000	0.412278000	
	F	-1.149980000	2.695799000	-1.436233000	
	F	-2.825466000	2.461254000	0.694179000	
	F	-1.550649000	-2.186650000	-1.793516000	
	F	-2.622823000	0.132893000	-0.465457000	
	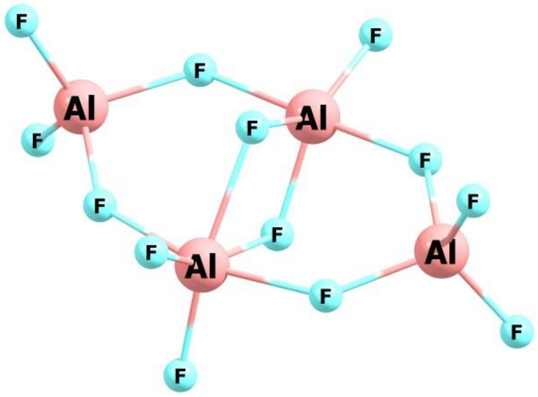				**1.6**
	Al	-1.509246000	-0.059264000	0.000000000	
	Al	0.113797000	0.060452000	3.049321000	
	Al	1.472355000	-0.065233000	0.000000000	
	Al	0.113797000	0.060452000	-3.049321000	
	F	0.181042000	1.151226000	0.000000000	
	F	-1.217453000	-0.056937000	-1.909188000	
	F	-1.217453000	-0.056937000	1.909188000	
	F	0.167991000	-1.291272000	4.054689000	
	F	0.152663000	1.561171000	-3.814110000	
	F	0.152663000	1.561171000	3.814110000	
	F	3.162729000	-0.117220000	0.000000000	
	F	1.479205000	-0.031550000	-1.904232000	
	F	0.180351000	-1.273333000	0.000000000	
	F	0.167991000	-1.291272000	-4.054689000	
	F	-2.482256000	1.342639000	0.000000000	
	F	1.479205000	-0.031550000	1.904232000	
	F	-2.482139000	-1.460948000	0.000000000	
	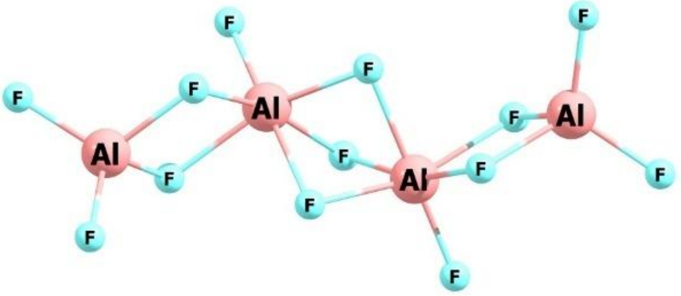				**2.4**
	Al	1.155880000	-0.591623000	0.495198000	
	Al	-1.156242000	0.593799000	0.493733000	
	Al	-3.660828000	-0.247565000	-0.520098000	
	Al	3.661100000	0.245376000	-0.520613000	
	F	-1.751596000	2.053670000	1.140450000	
	F	-0.000153000	0.002706000	1.844234000	
	F	0.472770000	1.072342000	-0.229387000	

$Al_4F_{13}^-$	F -2.248471000 0.672015000 -1.105093000<br>F 5.042664000 -0.696801000 -0.328815000<br>F -2.722153000 -0.438262000 0.984533000<br>F 2.722070000 0.441286000 0.983161000<br>F 3.899163000 1.689698000 -1.351175000<br>F -5.043363000 0.693815000 -0.331354000<br>F -3.897205000 -1.694336000 -1.346846000<br>F 2.248006000 -0.674266000 -1.103603000<br>F -0.473106000 -1.072008000 -0.226749000<br>F 1.751506000 -2.049841000 1.145437000	
	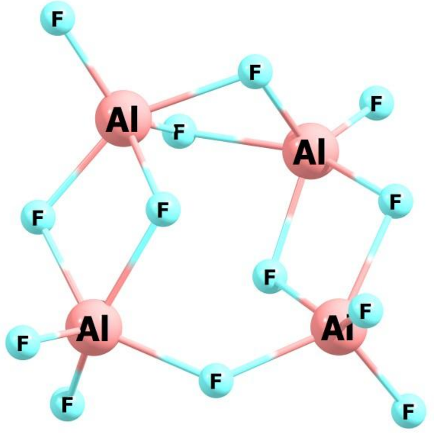	**5.6**
	Al 1.356543000 1.494293000 0.332573000<br>Al -1.353257000 1.496886000 -0.332551000<br>Al -1.653168000 -1.375502000 0.295091000<br>Al 1.650191000 -1.378772000 -0.295205000<br>F -2.368263000 0.386708000 0.687733000<br>F -0.907737000 -0.119311000 -1.013982000<br>F -0.345096000 1.994161000 1.096253000<br>F -2.794898000 -2.114540000 -0.715407000<br>F -1.751644000 -1.826282000 1.933182000<br>F 2.789212000 -2.120181000 0.716609000<br>F 0.906229000 -0.121004000 1.013080000<br>F 2.393570000 2.600662000 1.057451000<br>F 2.369868000 0.381822000 -0.686977000<br>F 0.349520000 1.992474000 -1.096581000<br>F -2.387684000 2.605817000 -1.057261000<br>F -0.002393000 -2.173028000 -0.000691000<br>F 1.748871000 -1.829494000 -1.933275000	
	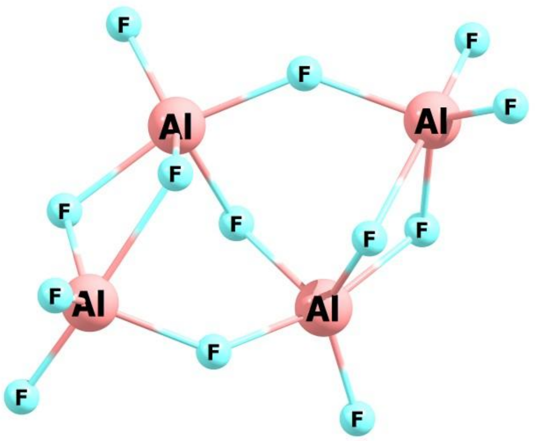	**6.3**
	Al -1.337256000 0.835836000 0.391770000	

	Al 1.337256000 -0.835836000 0.391770000<br>Al 1.834028000 1.950105000 -0.367935000<br>Al -1.834028000 -1.950105000 -0.367935000<br>F 0.000000000 1.991368000 -0.216448000<br>F -2.350455000 2.003852000 1.058768000<br>F -2.544573000 -2.243122000 -1.878996000<br>F -2.358858000 -0.692161000 0.865013000<br>F 0.000000000 0.000000000 1.345331000<br>F 2.358858000 0.692161000 0.865013000<br>F 2.167155000 3.316013000 0.601135000<br>F -2.167155000 -3.316013000 0.601135000<br>F -1.393058000 -0.022837000 -1.136566000<br>F 0.000000000 -1.991368000 -0.216448000<br>F 2.350455000 -2.003852000 1.058768000<br>F 2.544573000 2.243122000 -1.878996000<br>F 1.393058000 0.022837000 -1.136566000	
**$Al_4F_{13}^-$**	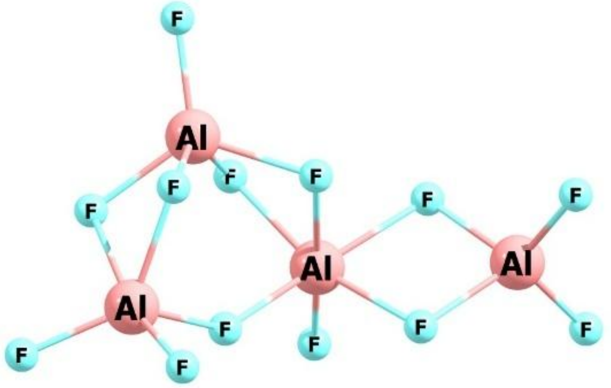	**6.8**
	Al -0.720757000 -0.086633000 -0.781952000<br>Al 1.398828000 1.508074000 0.102883000<br>Al 2.528853000 -1.057168000 0.282399000<br>Al -3.286684000 -0.221865000 0.422242000<br>F 0.839732000 -1.114098000 -0.485164000<br>F -1.187871000 -0.809687000 -2.249026000<br>F -4.586286000 -0.940996000 -0.366914000<br>F -2.377370000 0.926760000 -0.592214000<br>F 0.281165000 1.357514000 -1.393456000<br>F 2.622313000 0.632393000 -0.861576000<br>F 3.904174000 -1.590689000 -0.561099000<br>F -3.620031000 0.384822000 1.955628000<br>F -1.790626000 -1.197531000 0.365603000<br>F -0.159183000 0.819920000 0.802042000<br>F 1.672738000 3.129988000 0.456543000<br>F 2.288410000 -2.222310000 1.507470000<br>F 2.228045000 0.418215000 1.385226000	

$Al_4F_{13}^-$	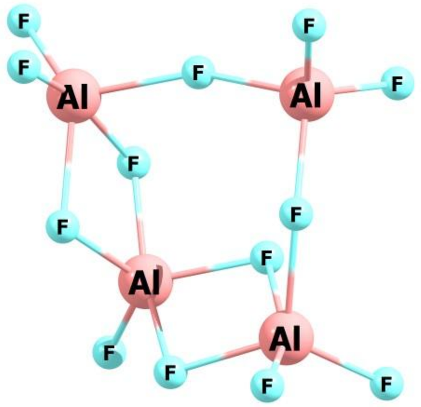	**8.3**
	Al 2.391842000 -0.134974000 0.322558000<br>Al -2.322683000 -0.347896000 0.346495000<br>Al 0.124276000 -1.842800000 -0.342779000<br>Al -0.178146000 2.262900000 -0.372969000<br>F 1.104047000 -1.290241000 1.094337000<br>F 1.299476000 1.264977000 -0.282402000<br>F -0.891374000 -0.538314000 -1.039449000<br>F -0.610139000 2.461388000 -1.984466000<br>F -1.376365000 -1.869380000 0.783757000<br>F -1.406687000 1.301115000 0.495815000<br>F 0.130623000 -3.444680000 -0.867017000<br>F 0.076734000 3.659658000 0.528902000<br>F -3.054121000 -0.154129000 1.872679000<br>F -3.524567000 -0.423401000 -0.843531000<br>F 2.709092000 0.571820000 1.844780000<br>F 3.921424000 -0.324832000 -0.392070000<br>F 1.599771000 -1.123311000 -1.143887000	
$P_4F_{21}^-$	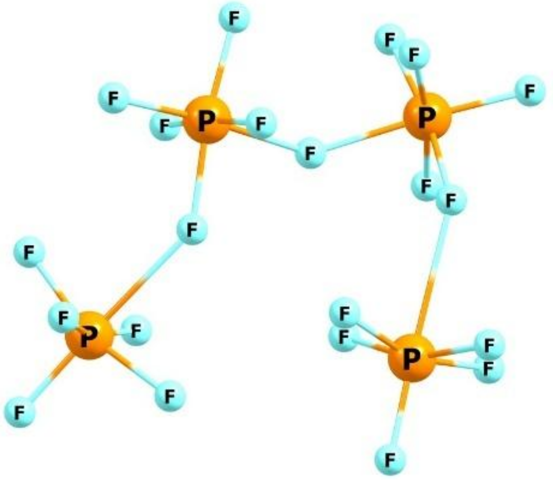	**0.0**
	P -2.693515000 -0.932960000 -0.435791000<br>P 0.518362000 -1.936208000 0.514130000<br>P 3.303794000 0.398249000 -0.361061000<br>F 2.508681000 0.381663000 -1.763023000<br>F 3.685202000 0.218802000 1.199221000<br>F -0.304425000 1.688331000 1.392791000<br>F 1.320994000 -0.463118000 0.395007000<br>F -2.738481000 -1.695970000 0.989758000<br>F -0.122475000 -1.399320000 1.899743000<br>F -2.412354000 -0.139326000 -1.823466000	

$P_4F_{21}^-$	F	1.032922000	-2.294951000	-0.981401000	
	F	-0.830611000	-1.025735000	-0.351960000	
	F	2.519279000	1.753246000	0.029209000	
	F	3.682148000	-1.151770000	-0.591601000	
	F	4.672851000	1.053823000	-0.864981000	
	F	-2.650195000	-2.351707000	-1.219727000	
	F	-4.300961000	-0.765986000	-0.531978000	
	F	1.776968000	-2.648336000	1.238132000	
	F	-0.424515000	-3.250348000	0.539023000	
	P	-1.096140000	2.544000000	0.287269000	
	F	-2.509288000	0.503014000	0.384020000	
	F	-0.231662000	3.880400000	0.288429000	
	F	-0.339325000	1.711148000	-0.855965000	
	F	-2.175822000	2.926645000	1.430798000	
	F	-2.213101000	2.947697000	-0.809607000	
	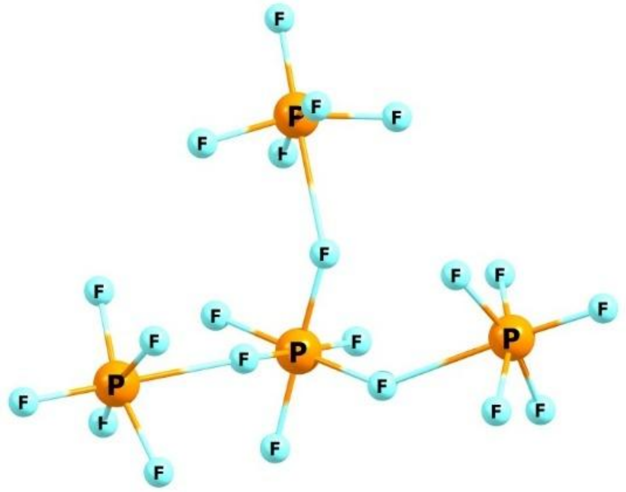				**0.1**
	P	-3.390651000	-0.812874000	-0.491951000	
	P	-0.187897000	-0.404546000	1.166649000	
	P	0.721462000	2.953591000	-0.243753000	
	F	2.278038000	2.572060000	-0.050673000	
	F	0.890125000	4.518048000	-0.530095000	
	F	-1.259513000	-0.792995000	-0.110793000	
	F	0.823574000	-1.589026000	0.509234000	
	F	-1.203562000	0.742878000	1.685238000	
	F	-3.537883000	-0.768553000	1.113900000	
	F	0.522268000	0.676139000	0.093935000	
	F	-0.881196000	2.850822000	-0.354231000	
	F	-3.207193000	0.789988000	-0.502081000	
	F	-2.837405000	-0.859326000	-2.012549000	
	F	0.578451000	3.013877000	1.361126000	
	F	0.824462000	2.418039000	-1.764156000	
	F	-3.161513000	-2.412932000	-0.397493000	
	F	-4.956560000	-0.851555000	-0.845407000	
	F	0.929606000	-0.052847000	2.288941000	
	F	-0.880191000	-1.547687000	2.086676000	
	P	2.888375000	-1.724819000	-0.452643000	
	F	2.153414000	-0.915466000	-1.636438000	
	F	3.144169000	-0.331396000	0.309683000	
	F	3.197031000	-2.500084000	0.933154000	
	F	2.206312000	-3.082665000	-1.006610000	

	Atom	X	Y	Z	
$P_4F_{21}^-$	F	4.325419000	-1.896239000	-1.135196000	
	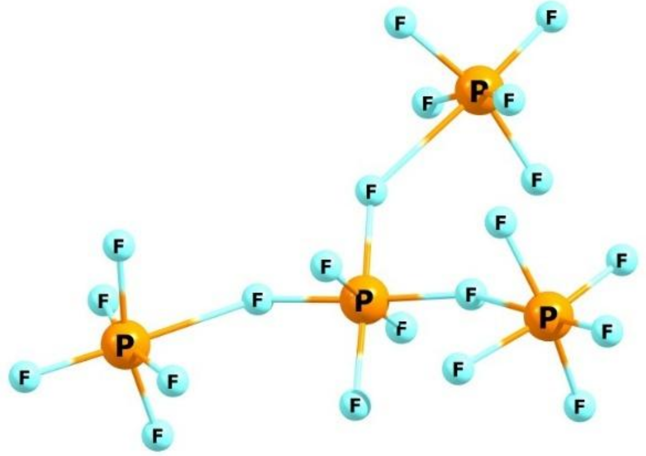				**0.3**
	P	1.974573000	2.652351000	-0.122574000	
	P	-0.426222000	-0.130588000	0.501686000	
	P	-3.964187000	-0.136670000	-0.216943000	
	F	-4.072707000	-0.607676000	1.327316000	
	F	-5.454251000	-0.542722000	-0.658151000	
	F	0.103118000	1.326999000	-0.168916000	
	F	-0.287240000	0.544185000	1.973344000	
	F	-0.563082000	-0.714829000	-1.003087000	
	F	2.295889000	1.858657000	1.241319000	
	F	-2.010681000	0.506016000	0.403474000	
	F	-3.478921000	0.448685000	-1.644211000	
	F	2.589545000	1.418445000	-0.954785000	
	F	1.267483000	3.170776000	-1.481223000	
	F	-3.250485000	-1.537271000	-0.571290000	
	F	-4.303336000	1.375910000	0.253940000	
	F	0.966697000	3.602386000	0.710701000	
	F	3.247093000	3.621566000	-0.142924000	
	F	-0.973537000	-1.512666000	1.144153000	
	F	1.170841000	-0.645243000	0.592470000	
	P	2.457459000	-2.371434000	-0.166582000	
	F	1.034403000	-3.076327000	-0.433911000	
	F	2.338567000	-2.804369000	1.386902000	
	F	3.411744000	-3.556504000	-0.662403000	
	F	3.597902000	-1.311113000	0.260897000	
	F	2.301588000	-1.587672000	-1.566262000	
	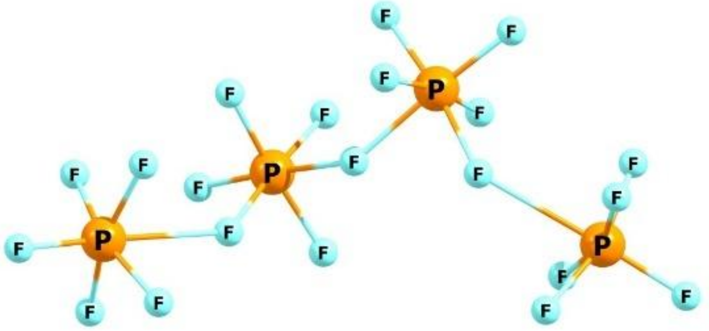				**1.9**
	P	1.052935000	-1.387441000	1.143044000	
	P	-1.052935000	1.387441000	1.143044000	
	P	0.200623000	4.060570000	-1.167978000	
	F	0.000000000	4.559712000	0.350267000	

	Atom	X	Y	Z	
	F	0.202573000	3.145494000	-2.498300000	
	F	-1.574543000	-3.349611000	-0.721101000	
	F	-0.701488000	2.066304000	-0.352812000	
	F	-0.317453000	-2.026892000	1.718008000	
	F	-2.275702000	0.600215000	0.424375000	
	F	2.275702000	-0.600215000	0.424375000	
	F	0.317453000	2.026892000	1.718008000	
	F	0.000000000	0.000000000	0.501665000	
	F	1.574543000	3.349611000	-0.721101000	
	F	-1.367623000	4.352631000	-1.427209000	
	F	0.802966000	5.401513000	-1.793865000	
	F	1.282255000	-0.553407000	2.507975000	
	F	1.967913000	-2.645417000	1.583386000	
	F	-1.967913000	2.645417000	1.583386000	
	F	-1.282255000	0.553407000	2.507975000	
	P	-0.200623000	-4.060570000	-1.167978000	
	F	0.701488000	-2.066304000	-0.352812000	
	F	-0.802966000	-5.401513000	-1.793865000	
	F	-0.202573000	-3.145494000	-2.498300000	
	F	0.000000000	-4.559712000	0.350267000	
	F	1.367623000	-4.352631000	-1.427209000	
$P_4F_{21}^-$	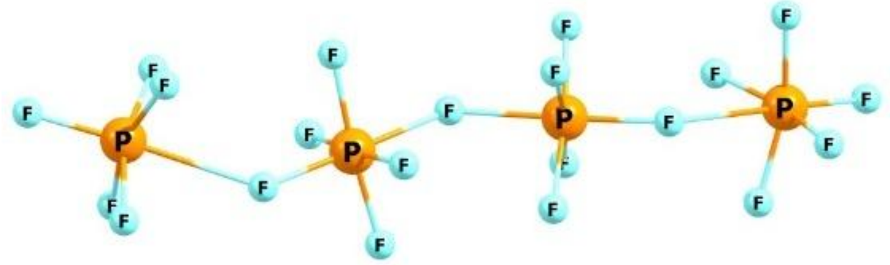				**2.4**
	P	0.000000000	-1.734828000	-0.138214000	
	P	0.000000000	1.734828000	-0.138214000	
	F	0.000000000	0.000000000	0.496120000	
	F	-1.469533000	1.448690000	-0.760132000	
	F	1.468941000	1.876002000	0.537286000	
	F	1.469533000	-1.448690000	-0.760132000	
	F	-0.682435000	2.111260000	1.286382000	
	F	0.682435000	-2.111260000	1.286382000	
	F	-1.468941000	-1.876002000	0.537286000	
	F	-0.684469000	-1.213358000	-1.508331000	
	F	0.014452000	-3.318473000	-0.660990000	
	F	-0.014452000	3.318473000	-0.660990000	
	F	0.684469000	1.213358000	-1.508331000	
	P	0.719503000	5.401616000	0.139424000	
	F	0.350521000	5.758231000	-1.392978000	
	F	2.135544000	4.830887000	-0.379197000	
	F	0.932137000	4.590793000	1.512186000	
	F	-0.850511000	5.521741000	0.496523000	
	F	1.193188000	6.848913000	0.619174000	
	P	-0.719503000	-5.401616000	0.139424000	
	F	-2.135544000	-4.830887000	-0.379197000	
	F	-0.932137000	-4.590793000	1.512186000	
	F	0.850511000	-5.521741000	0.496523000	
	F	-0.350521000	-5.758231000	-1.392978000	

	F -1.193188000 -6.848913000 0.619174000	
$As_4F_{21}^-$	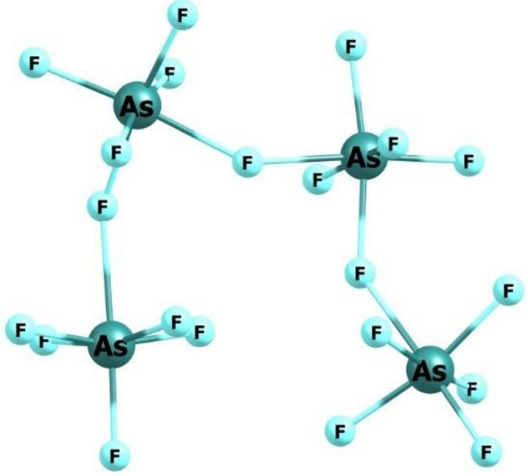	**0.0**
	As -2.594925000 -1.134592000 -0.471219000<br>As 0.800465000 -1.920494000 0.537992000<br>As 3.255318000 0.661917000 -0.365640000<br>F 2.457387000 0.520288000 -1.892757000<br>F 3.748326000 0.618197000 1.295567000<br>F -0.495183000 1.582019000 1.478240000<br>F 1.532066000 -0.253041000 0.377606000<br>F -2.565226000 -1.831439000 1.103603000<br>F 0.100652000 -1.331935000 1.993150000<br>F -2.373349000 -0.313343000 -1.967366000<br>F 1.342617000 -2.246576000 -1.061044000<br>F -0.680502000 -1.058164000 -0.367623000<br>F 2.347535000 2.093134000 -0.035766000<br>F 3.859811000 -0.949600000 -0.553438000<br>F 4.664428000 1.455734000 -0.956453000<br>F -2.446622000 -2.678264000 -1.225945000<br>F -4.314674000 -1.063939000 -0.540167000<br>F 2.203127000 -2.549255000 1.302910000<br>F -0.119149000 -3.375765000 0.595873000<br>As -1.491209000 2.363239000 0.306026000<br>F -2.536303000 0.482132000 0.358670000<br>F -0.713436000 3.892156000 0.324240000<br>F -0.553186000 1.645106000 -0.947401000<br>F -2.641437000 2.710711000 1.555646000<br>F -2.705592000 2.761592000 -0.863792000	
	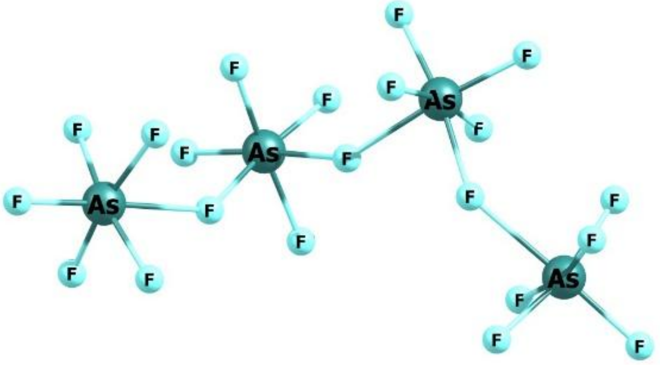	**0.9**
	As 1.076895000 -1.447408000 1.215528000<br>As -1.076895000 1.447408000 1.215528000	

$As_4F_{21}^-$

As	0.188596000	3.907146000	-1.200929000
F	0.000000000	4.556406000	0.391100000
F	0.208352000	2.949124000	-2.641930000
F	-1.673428000	-3.170379000	-0.710685000
F	-0.682526000	2.175851000	-0.422877000
F	-0.405396000	-2.094419000	1.792229000
F	-2.365051000	0.650875000	0.396894000
F	2.365051000	-0.650875000	0.396894000
F	0.405396000	2.094419000	1.792229000
F	0.000000000	0.000000000	0.519923000
F	1.673428000	3.170379000	-0.710685000
F	-1.456662000	4.333627000	-1.539229000
F	0.892326000	5.314453000	-1.898148000
F	1.340941000	-0.546885000	2.657153000
F	2.022669000	-2.809092000	1.662003000
F	-2.022669000	2.809092000	1.662003000
F	-1.340941000	0.546885000	2.657153000
As	-0.188596000	-3.907146000	-1.200929000
F	0.682526000	-2.175851000	-0.422877000
F	-0.892326000	-5.314453000	-1.898148000
F	-0.208352000	-2.949124000	-2.641930000
F	0.000000000	-4.556406000	0.391100000
F	1.456662000	-4.333627000	-1.539229000

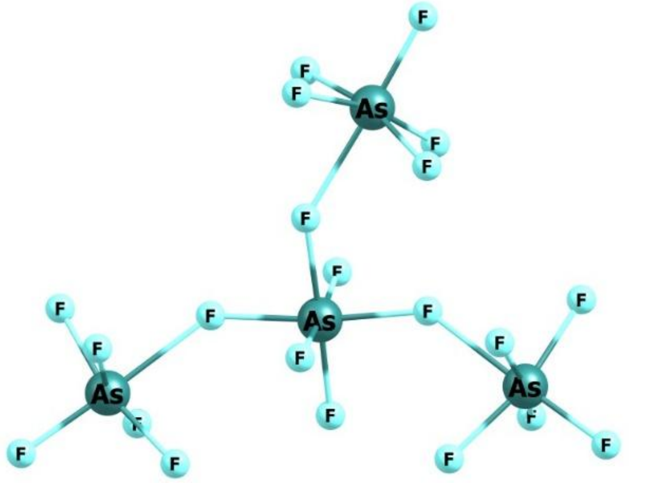

1.5

As	-1.670904000	2.817775000	0.091545000
As	0.361065000	-0.238417000	-0.413900000
As	3.981160000	-0.311336000	0.170328000
F	4.022007000	-0.985438000	-1.423464000
F	5.561072000	-0.808256000	0.632431000
F	-0.066263000	1.432880000	0.177957000
F	0.187461000	0.368888000	-2.013454000
F	0.510560000	-0.672515000	1.238558000
F	-2.190228000	1.913321000	-1.285495000
F	2.077777000	0.365743000	-0.427005000
F	3.581029000	0.482289000	1.654106000
F	-2.455283000	1.671284000	1.117060000
F	-0.846722000	3.459216000	1.472076000
F	3.151987000	-1.721479000	0.722252000
F	4.444104000	1.218491000	-0.494600000

	F	-0.576170000	3.690003000	-0.927442000	
	F	-2.937704000	3.979325000	0.063833000	
	F	0.811275000	-1.793947000	-0.975693000	
	F	-1.398495000	-0.631690000	-0.390647000	
	As	-2.668708000	-2.263596000	0.141158000	
	F	-1.216508000	-2.993967000	0.725635000	
	F	-2.271815000	-2.815487000	-1.450721000	
	F	-3.731256000	-3.553636000	0.541237000	
	F	-3.857085000	-1.216501000	-0.549497000	
	F	-2.809325000	-1.404755000	1.632724000	
**$As_4F_{21}^-$**	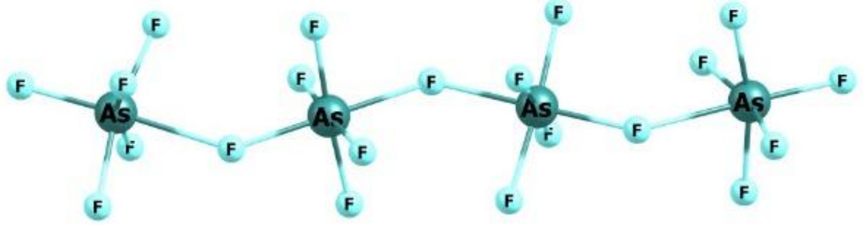				**1.9**
	As	-0.075120000	1.796816000	-0.053568000	
	As	0.075120000	-1.796816000	-0.053568000	
	F	0.000000000	0.000000000	0.621161000	
	F	1.623710000	-1.441671000	-0.726558000	
	F	-1.478230000	-2.043917000	0.652921000	
	F	-1.623710000	1.441671000	-0.726558000	
	F	0.825500000	-2.209032000	1.442251000	
	F	-0.825500000	2.209032000	1.442251000	
	F	1.478230000	2.043917000	0.652921000	
	F	0.677408000	1.275586000	-1.513960000	
	F	-0.159530000	3.497982000	-0.680006000	
	F	0.159530000	-3.497982000	-0.680006000	
	F	-0.677408000	-1.275586000	-1.513960000	
	As	0.000000000	-5.467885000	0.050517000	
	F	0.647962000	-5.822927000	-1.515382000	
	F	-1.578550000	-5.379805000	-0.653904000	
	F	-0.622442000	-4.733351000	1.483863000	
	F	1.607013000	-5.184680000	0.624383000	
	F	-0.116105000	-7.097010000	0.586997000	
	As	0.000000000	5.467885000	0.050517000	
	F	1.578550000	5.379805000	-0.653904000	
	F	0.622442000	4.733351000	1.483863000	
	F	-1.607013000	5.184680000	0.624383000	
	F	-0.647962000	5.822927000	-1.515382000	
	F	0.116105000	7.097010000	0.586997000	

$As_4F_{21}^-$

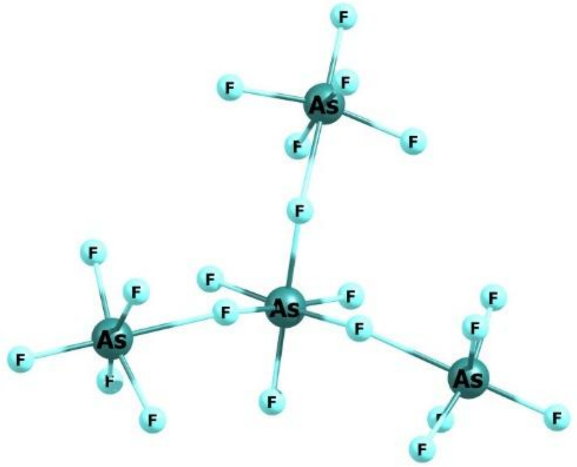

2.1

As	-3.551657000	-0.797768000	-0.313985000
As	-0.121011000	-0.141810000	0.850991000
As	0.997877000	3.193378000	-0.243782000
F	2.505754000	2.372582000	-0.061742000
F	1.628933000	4.792359000	-0.253687000
F	-1.450357000	-0.540821000	-0.328458000
F	0.881910000	-1.113830000	-0.315192000
F	-1.183946000	0.864669000	1.741748000
F	-3.373020000	-0.965018000	1.396673000
F	0.205793000	1.218166000	-0.310009000
F	-0.666011000	3.628426000	-0.426546000
F	-3.571241000	0.919723000	-0.127536000
F	-3.327226000	-0.588204000	-2.016913000
F	0.751106000	3.079301000	1.462915000
F	1.086282000	2.930339000	-1.951725000
F	-3.131359000	-2.466684000	-0.493749000
F	-5.256390000	-1.016001000	-0.362805000
F	1.275453000	0.260896000	1.755851000
F	-0.476872000	-1.567817000	1.736008000
As	2.672976000	-2.257544000	-0.287464000
F	2.590886000	-1.945361000	-1.987429000
F	3.428136000	-0.721924000	-0.063320000
F	2.405076000	-2.357998000	1.417055000
F	1.570656000	-3.573411000	-0.508186000
F	4.113091000	-3.195661000	-0.324071000

BPF_9^-

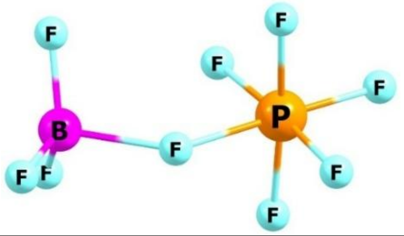

0.0

P	1.119897000	0.000009000	0.014058000
F	2.650461000	-0.000300000	-0.566950000
F	0.464284000	0.006700000	-1.482309000
F	1.052895000	-1.637190000	0.037777000
F	1.058260000	1.637255000	0.049997000
F	1.635536000	-0.006616000	1.572415000
B	-2.075476000	-0.000012000	-0.012227000
F	-2.107489000	-1.165476000	-0.736669000

	F	-2.110422000	1.169124000	-0.730662000	
	F	-0.528206000	0.000358000	0.693687000	
	F	-2.828773000	-0.003863000	1.146077000	
**$BAsF_9^-$**	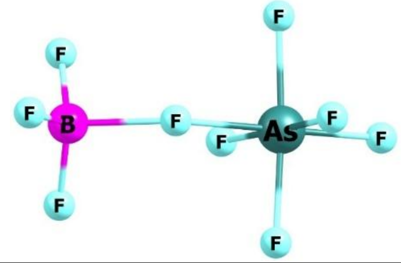				**0.0**
	As	0.972961000	0.000003000	0.010602000	
	F	2.611549000	-0.000201000	-0.591956000	
	F	0.290080000	0.008727000	-1.591556000	
	F	0.903829000	-1.744815000	0.034151000	
	F	0.910288000	1.744829000	0.050278000	
	F	1.505546000	-0.008629000	1.676277000	
	B	-2.315365000	-0.000018000	-0.010008000	
	F	-2.331494000	-1.164618000	-0.734274000	
	F	-2.335246000	1.169630000	-0.726143000	
	F	-0.761048000	0.000261000	0.707460000	
	F	-3.074715000	-0.005184000	1.142450000	
**$AlPF_9^-$**	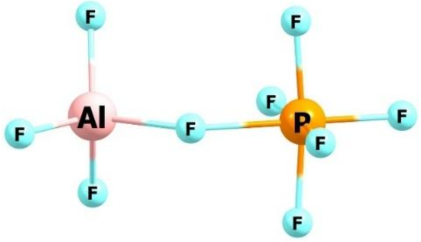				**0.0**
	P	1.389036000	0.000011000	0.018382000	
	F	2.890321000	-0.000989000	-0.609608000	
	F	0.658242000	0.004719000	-1.444288000	
	F	1.298560000	-1.629964000	0.054136000	
	F	1.304005000	1.630131000	0.061842000	
	F	1.916852000	-0.004494000	1.565479000	
	Al	-1.987462000	0.000005000	-0.004295000	
	F	-2.144599000	-1.428795000	-0.904294000	
	F	-2.148624000	1.433195000	-0.896667000	
	F	-0.293592000	0.001242000	0.732100000	
	F	-2.925446000	-0.005070000	1.416869000	
**$AlAsF_9^-$**	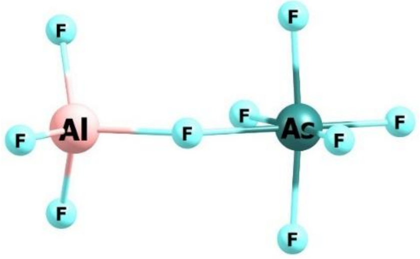				**0.0**
	As	1.225673000	0.000019000	0.012945000	
	F	2.846159000	-0.001049000	-0.612822000	
	F	0.484523000	0.006831000	-1.562251000	
	F	1.128909000	-1.737517000	0.049786000	
	F	1.135792000	1.737687000	0.061467000	

	F	1.739959000	-0.006542000	1.677824000	
	Al	-2.260221000	-0.000005000	-0.001349000	
	F	-2.409259000	-1.428262000	-0.902305000	
	F	-2.414424000	1.433800000	-0.892674000	
	F	-0.553330000	0.001359000	0.710303000	
	F	-3.187697000	-0.006369000	1.425153000	
$B_2PF_{12}^-$	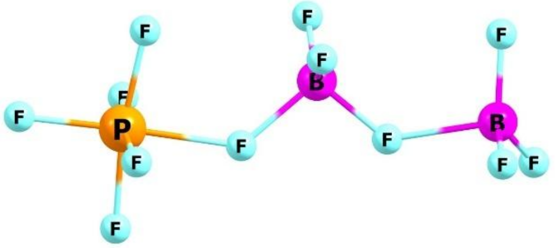				**0.0**
	B	3.528543000	-0.260235000	-0.041359000	
	B	0.746158000	0.479263000	0.069578000	
	P	-2.301669000	-0.134738000	-0.029563000	
	F	-3.897591000	0.071034000	0.188471000	
	F	1.805604000	-0.368743000	-0.501320000	
	F	3.572955000	-1.142131000	0.999460000	
	F	0.638539000	1.652253000	-0.619748000	
	F	-2.100969000	-0.958713000	1.362040000	
	F	-1.889421000	1.241846000	0.732956000	
	F	-0.467541000	-0.422933000	-0.307904000	
	F	0.835173000	0.535230000	1.429713000	
	F	-2.470920000	-1.547616000	-0.828127000	
	F	3.640353000	1.067559000	0.257724000	
	F	-2.278143000	0.654736000	-1.455157000	
	F	4.073242000	-0.679640000	-1.224513000	
	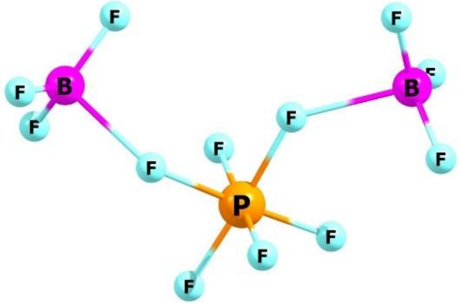				**1.3**
	P	-0.003764000	0.981557000	0.022600000	
	B	-2.742353000	-0.959627000	-0.028874000	
	B	2.750360000	-0.933508000	-0.007810000	
	F	1.314289000	1.701430000	-0.616692000	
	F	2.894515000	-0.742650000	-1.339383000	
	F	0.477050000	1.350922000	1.541576000	
	F	0.845091000	-0.491465000	0.154715000	
	F	2.591517000	-2.205526000	0.440373000	
	F	-3.352218000	-0.047977000	-0.821951000	
	F	-1.331747000	0.161398000	0.717675000	
	F	-3.304203000	-1.221119000	1.181760000	
	F	-0.897350000	2.347537000	-0.066408000	
	F	3.265584000	-0.017595000	0.843250000	
	F	-1.984007000	-1.922248000	-0.599458000	
	F	-0.516696000	0.503105000	-1.452743000	

$B_2PF_{12}^-$	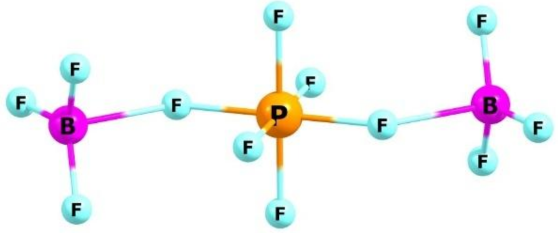	1.3

B	3.347102000	0.001135000	-0.031736000
P	0.000082000	-0.001244000	0.000200000
B	-3.347291000	0.001127000	0.031324000
F	-3.955284000	0.002455000	-1.185460000
F	1.546085000	-0.001126000	0.716532000
F	3.300986000	1.166606000	-0.716734000
F	0.000030000	-1.636838000	0.000390000
F	-3.300852000	1.166207000	0.717611000
F	-1.545936000	-0.001234000	-0.715974000
F	-0.669825000	-0.000991000	1.491015000
F	0.000067000	1.634281000	0.000063000
F	0.669886000	-0.001307000	-1.490671000
F	-3.304135000	-1.164618000	0.716633000
F	3.303347000	-1.163668000	-0.718007000
F	3.955601000	0.001050000	1.184497000

$B_2AsF_{12}^-$	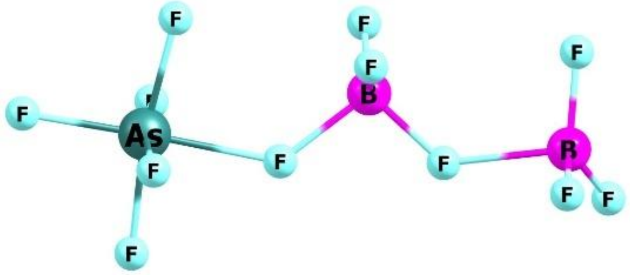	0.0

B	3.881778000	-0.267082000	-0.041703000
B	1.075202000	0.485943000	0.072028000
As	-2.060804000	-0.104998000	-0.023770000
F	-2.251180000	-1.602800000	-0.890860000
F	3.980191000	1.053558000	0.277079000
F	4.402242000	-0.667321000	-1.237731000
F	1.152212000	0.520539000	1.433184000
F	-2.042447000	0.752931000	-1.536580000
F	-0.168555000	-0.404508000	-0.313384000
F	-1.620621000	1.354835000	0.805942000
F	0.957587000	1.667660000	-0.599376000
F	-3.765772000	0.117446000	0.211122000
F	2.112735000	-0.359833000	-0.510382000
F	-1.859059000	-1.001034000	1.453079000
F	3.905070000	-1.168070000	0.978219000

$B_2AsF_{12}^-$	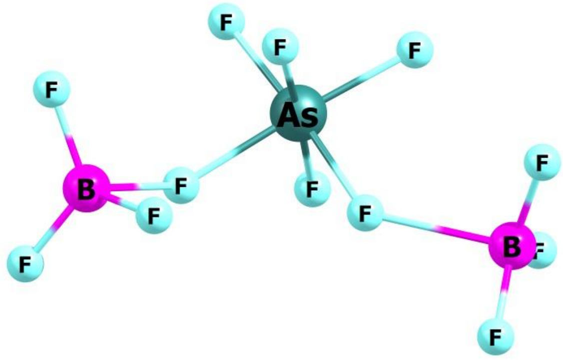				**0.6**
	As	0.002924000	-0.895751000	0.017262000	
	B	2.787797000	1.115014000	-0.026340000	
	B	-2.794408000	1.089501000	-0.007590000	
	F	-1.405489000	-1.672299000	-0.645384000	
	F	-2.937412000	0.886625000	-1.338322000	
	F	-0.488634000	-1.278344000	1.644320000	
	F	-0.900122000	0.662062000	0.158986000	
	F	-2.654267000	2.367699000	0.430819000	
	F	3.395251000	0.207796000	-0.827560000	
	F	1.409343000	-0.014166000	0.737677000	
	F	3.371469000	1.392456000	1.170861000	
	F	0.964396000	-2.345670000	-0.076520000	
	F	-3.303962000	0.174445000	0.849402000	
	F	2.008083000	2.066167000	-0.589314000	
	F	0.534293000	-0.387082000	-1.559407000	
	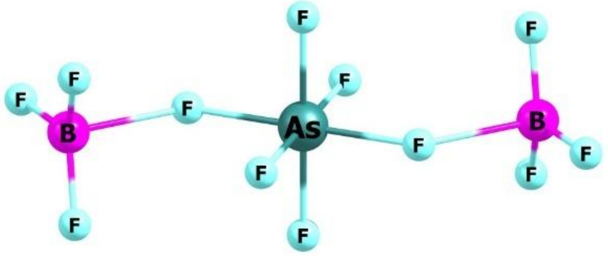				**0.7**
	B	3.422477000	0.000008000	-0.028823000	
	As	-0.000017000	0.000001000	0.000058000	
	B	-3.422292000	-0.000001000	0.028668000	
	F	-4.060711000	-0.000573000	-1.172825000	
	F	1.648461000	-0.000031000	0.738586000	
	F	3.366376000	1.165028000	-0.716415000	
	F	-0.000478000	-1.742218000	0.001022000	
	F	-3.366768000	1.165577000	0.715396000	
	F	-1.648534000	0.000007000	-0.738516000	
	F	-0.691322000	0.001064000	1.597977000	
	F	0.000410000	1.742222000	-0.000939000	
	F	0.691221000	-0.001084000	-1.597896000	
	F	-3.366388000	-1.165022000	0.716354000	
	F	3.366893000	-1.165551000	-0.715566000	
	F	4.060801000	0.000573000	1.172695000	

$Al_2PF_{12}^-$

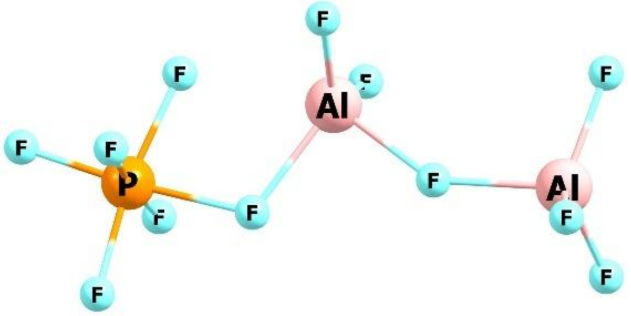

0.0

Al	3.492056000	-0.379418000	-0.000103000
Al	0.202326000	0.805102000	0.000182000
P	-2.613853000	-0.306505000	-0.000110000
F	-4.143916000	0.208068000	-0.000446000
F	1.661213000	-0.276798000	-0.000849000
F	3.852477000	-1.234204000	1.420859000
F	0.307740000	1.584274000	-1.486357000
F	-2.515851000	-0.304759000	1.622124000
F	-1.980055000	1.255631000	-0.000372000
F	-0.846971000	-0.729916000	0.000213000
F	0.308004000	1.582421000	1.487645000
F	-2.950641000	-1.890924000	0.000196000
F	3.989787000	1.241881000	0.000097000
F	-2.515393000	-0.305488000	-1.622297000
F	3.853698000	-1.234222000	-1.420745000

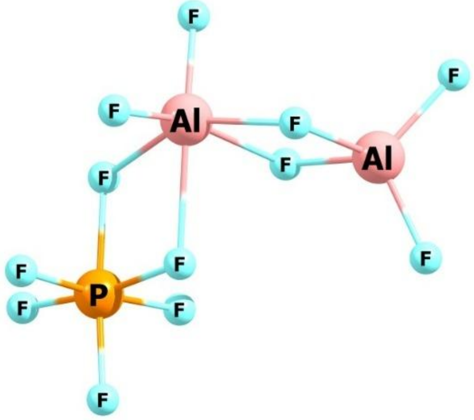

0.6

Al	-2.590666000	-0.723201000	0.033597000
Al	-0.470648000	1.230915000	0.050396000
P	2.051738000	-0.413883000	-0.065607000
F	3.303971000	-0.248962000	-1.087536000
F	-1.986391000	0.597145000	1.070575000
F	-2.511872000	-2.223650000	0.797524000
F	0.151201000	2.187941000	1.313864000
F	1.331767000	-1.462381000	-1.079997000
F	1.197760000	0.881513000	-0.901452000
F	0.622607000	-0.409561000	0.882315000
F	-1.194591000	-0.341116000	-0.972216000
F	2.694533000	-1.620588000	0.806159000
F	-1.198070000	2.270308000	-1.090867000
F	2.673094000	0.709708000	0.926138000
F	-4.081675000	-0.383918000	-0.676487000

$Al_2AsF_{12}^-$

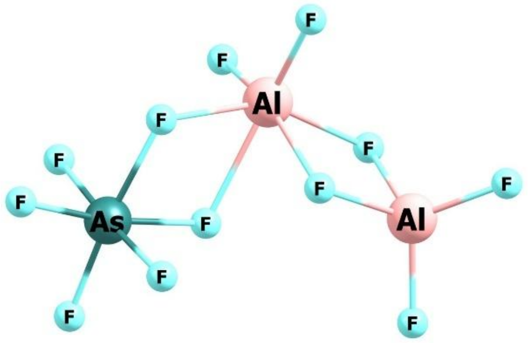

0.0

Al	-2.799464000	-0.782126000	0.039636000
Al	-0.786072000	1.275678000	0.043300000
As	1.872480000	-0.314035000	-0.049910000
F	3.187460000	-0.079401000	-1.156146000
F	-2.276372000	0.583291000	1.062721000
F	-2.646592000	-2.266041000	0.822801000
F	-0.186577000	2.251794000	1.302007000
F	1.153117000	-1.469648000	-1.126126000
F	0.892351000	1.007082000	-0.915168000
F	0.357104000	-0.342541000	0.938247000
F	-1.415553000	-0.335745000	-0.961215000
F	2.610493000	-1.570560000	0.885189000
F	-1.567711000	2.278796000	-1.093891000
F	2.502267000	0.911968000	0.997269000
F	-4.296638000	-0.530440000	-0.692484000

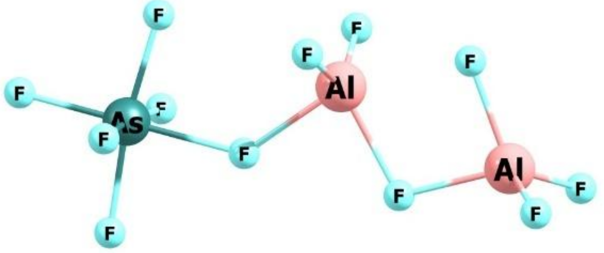

0.3

Al	3.697867000	-0.003756000	-0.364236000
Al	0.978289000	0.007051000	0.657844000
As	-2.370080000	-0.001909000	-0.178113000
F	-4.081249000	-0.001250000	0.117320000
F	1.982504000	-0.008162000	-0.931698000
F	4.488288000	-1.431828000	-0.777811000
F	0.799951000	1.541568000	1.332068000
F	-2.272183000	-1.739157000	-0.182621000
F	-1.951267000	0.015855000	1.514880000
F	-0.491180000	-0.002533000	-0.501336000
F	0.799550000	-1.514602000	1.360486000
F	-2.541713000	-0.019673000	-1.912332000
F	2.990432000	0.012140000	1.234727000
F	-2.276981000	1.735187000	-0.217835000
F	4.489694000	1.414696000	-0.806866000

<table>
<tr><td rowspan="4">$BAlPF_{12}^{-}$</td><td>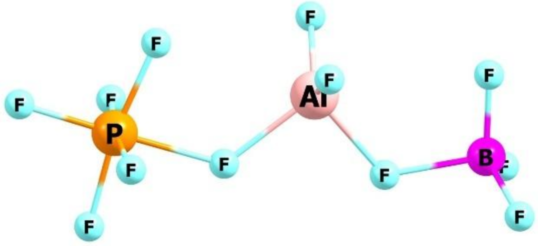
</td><td>0.0</td></tr>
<tr><td>B 3.555923473 -0.381765930 -0.000488960
Al 0.722167133 0.556743150 0.000601145
P -2.438372033 -0.200206275 -0.000249772
F -4.000315414 0.229783049 0.001086930
F 1.948311037 -0.750416202 -0.001436187
F 4.013333174 -0.957525065 1.162741060
F 0.748560907 1.365136012 -1.471268330
F -2.332832922 -0.226822832 1.625084281
F -1.883634993 1.347513351 0.003187764
F -0.644185986 -0.657518078 -0.001694955
F 0.748641871 1.359801905 1.475398030
F -2.731440448 -1.802272933 -0.003869873
F 3.498645327 1.007021130 0.001705009
F -2.333554191 -0.219330211 -1.625744165
F 4.013783504 -0.953785899 -1.165371071</td><td></td></tr>
<tr><td>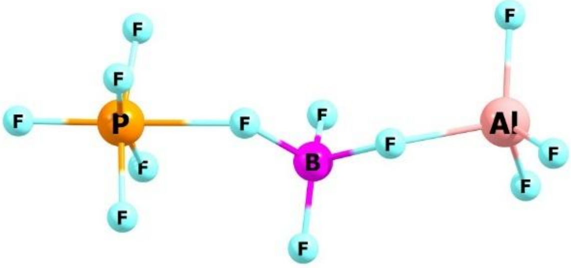
</td><td>7.4</td></tr>
<tr><td>Al 3.392321000 -0.246470000 0.031670000
B 0.420895000 0.601980000 -0.050853000
P -2.588938000 -0.169230000 0.025941000
F -4.185923000 -0.036169000 -0.180291000
F 1.603194000 -0.175989000 0.524382000
F 4.118270000 -0.715182000 1.491412000
F 0.578177000 0.684769000 -1.400830000
F -2.563062000 0.606339000 1.453641000
F -2.215578000 1.222968000 -0.722548000
F -0.666472000 -0.377756000 0.298645000
F 0.278312000 1.748456000 0.667564000
F -2.653518000 -1.589155000 0.815641000
F 3.754922000 1.331302000 -0.463173000
F -2.329859000 -0.970672000 -1.363100000
F 3.462584000 -1.425281000 -1.182070000</td><td></td></tr>
</table>

$BAlPF_{12}^-$

7.6

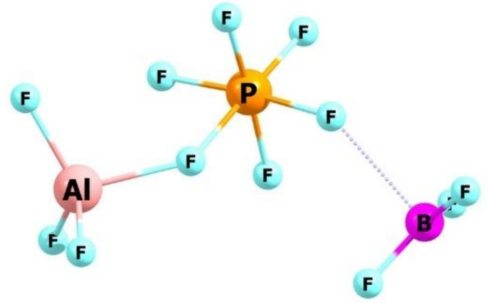

P	-0.234261000	1.123883000	0.026452000
B	-3.014768000	-1.139314000	-0.035956000
Al	2.545503000	-0.780624000	-0.003274000
F	1.081559000	1.785818000	-0.669140000
F	2.768966000	-0.740026000	-1.682136000
F	0.269586000	1.615070000	1.493960000
F	0.758264000	-0.362776000	0.295936000
F	2.590782000	-2.347828000	0.652374000
F	-3.574249000	-0.256938000	-0.878660000
F	-1.458009000	0.281073000	0.771384000
F	-3.529887000	-1.279451000	1.200571000
F	-1.164160000	2.435403000	-0.196201000
F	3.457374000	0.344115000	0.876448000
F	-2.137967000	-2.043537000	-0.491988000
F	-0.673790000	0.456460000	-1.391932000

7.7

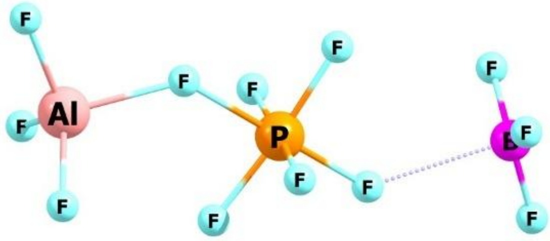

Al	-3.131397000	-0.000029000	0.047688000
P	0.235312000	0.000035000	-0.079944000
B	3.790389000	-0.000050000	0.119878000
F	4.341437000	0.000051000	-1.108654000
F	-1.389023000	-0.000002000	0.700455000
F	-3.312191000	-1.432445000	-0.837974000
F	0.173074000	1.626980000	-0.048275000
F	3.641132000	-1.161880000	0.773045000
F	1.727036000	0.000096000	-0.794079000
F	0.859711000	0.000125000	1.425388000
F	0.173211000	-1.626922000	-0.048196000
F	-0.534306000	-0.000013000	-1.517539000
F	3.641193000	1.161681000	0.773257000
F	-3.312202000	1.432336000	-0.838055000
F	-3.983903000	0.000005000	1.518386000

<table>
<tr><td rowspan="4">$BAlAsF_{12}^-$</td><td>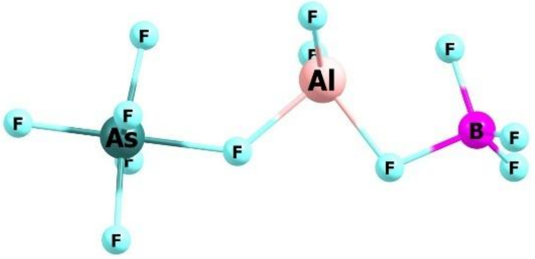
</td><td>0.0</td></tr>
<tr><td colspan="2">B 3.693634000 -0.437088000 -0.000796000
Al 1.212652000 0.589063000 0.001009000
As -2.148684000 -0.149086000 -0.000306000
F -2.365447000 -1.877036000 -0.001081000
F 3.346062000 0.963496000 0.002058000
F 4.284940000 -0.850590000 -1.166389000
F 1.100760000 1.333691000 1.506082000
F -2.049112000 -0.173107000 -1.736697000
F -0.271295000 -0.526271000 -0.001277000
F -1.675271000 1.528882000 0.000497000
F 1.100000000 1.340734000 -1.500496000
F -3.849196000 0.196368000 0.000843000
F 2.217558000 -0.966874000 -0.003171000
F -2.047270000 -0.174834000 1.735953000
F 4.283150000 -0.855852000 1.163786000</td></tr>
<tr><td>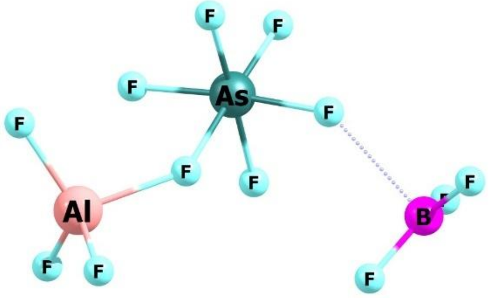
</td><td>7.1</td></tr>
<tr><td colspan="2">As -0.239619000 -1.062384000 -0.020508000
B -2.939361000 1.404438000 0.035681000
Al 2.604705000 0.932891000 0.003539000
F 1.158378000 -1.797973000 0.697818000
F 2.847619000 0.866428000 1.678587000
F 0.284355000 -1.546416000 -1.602365000
F 0.817477000 0.490565000 -0.264005000
F 2.630086000 2.506801000 -0.633494000
F -3.559393000 0.568352000 0.884277000
F -1.530111000 -0.128036000 -0.787311000
F -3.464387000 1.598361000 -1.189724000
F -1.249845000 -2.456104000 0.180270000
F 3.499904000 -0.183074000 -0.903460000
F -1.982841000 2.229436000 0.482905000
F -0.702011000 -0.380684000 1.506764000</td></tr>
</table>

$BAlAsF_{12}^{-}$

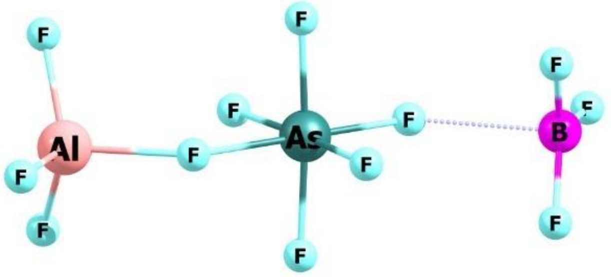

7.2

B	3.848144000	0.000830000	0.141342000
As	0.219730000	-0.000614000	-0.081734000
Al	-3.255060000	0.000540000	0.067722000
F	-4.100208000	0.003518000	1.541379000
F	1.826004000	-0.001439000	-0.805750000
F	3.684472000	-1.160072000	0.794344000
F	0.154459000	1.734178000	-0.050274000
F	-3.429329000	-1.432916000	-0.816599000
F	-1.503306000	0.000218000	0.696745000
F	-0.568797000	-0.003038000	-1.629179000
F	0.155017000	-1.735337000	-0.045160000
F	0.846516000	0.001859000	1.538649000
F	-3.427411000	1.431298000	-0.821306000
F	3.683695000	1.163737000	0.790402000
F	4.437107000	-0.000994000	-1.069905000

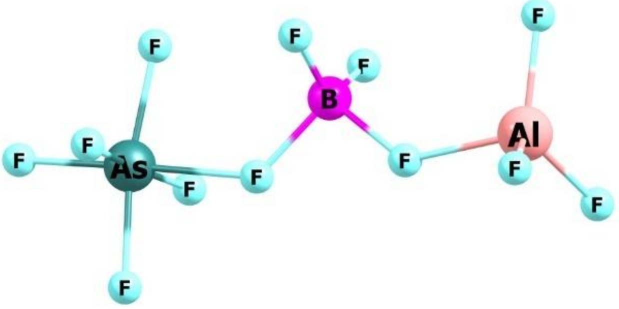

7.6

Al	3.760513000	-0.233848000	-0.000147000
B	0.759776000	0.555225000	0.000248000
As	-2.335566000	-0.125490000	-0.000094000
F	-4.046475000	0.121708000	0.000743000
F	1.910312000	-0.439010000	-0.001857000
F	4.226637000	-1.029199000	1.421135000
F	0.766822000	1.240403000	-1.172001000
F	-2.197010000	-0.151326000	1.729552000
F	-1.927053000	1.559673000	0.002712000
F	-0.375175000	-0.461135000	-0.001139000
F	0.767541000	1.236556000	1.174927000
F	-2.428984000	-1.859411000	-0.003054000
F	3.982402000	1.442935000	0.001656000
F	-2.197843000	-0.145372000	-1.729886000
F	4.228618000	-1.026367000	-1.422369000

$BP_2F_{14}^-$	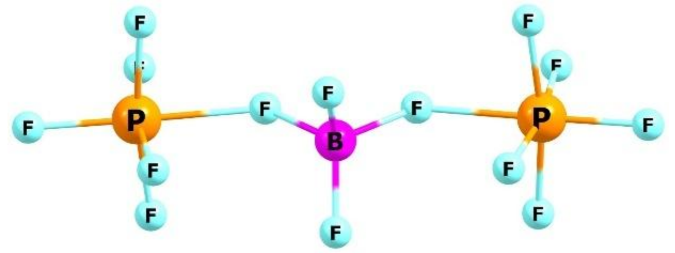				**0.0**
	F	1.779757000	3.119504000	0.000000000	
	F	0.175011000	2.893905000	1.621484000	
	F	-1.219350000	0.000341000	-1.172838000	
	F	-1.426626000	2.646327000	0.000000000	
	F	-1.426461000	-2.646297000	0.000000000	
	F	-0.078902000	-4.626198000	0.000000000	
	F	0.175011000	-2.893988000	-1.621323000	
	F	0.486187000	1.133936000	0.000000000	
	F	-1.219350000	0.000341000	1.172838000	
	F	1.779670000	-3.119326000	0.000000000	
	F	0.175011000	-2.893988000	1.621323000	
	F	0.486246000	-1.133058000	0.000000000	
	B	-0.534720000	-0.000228000	0.000000000	
	P	0.154453000	3.023481000	0.000000000	
	P	0.154408000	-3.024072000	0.000000000	
	F	0.175011000	2.893905000	-1.621484000	
	F	-0.078915000	4.625707000	0.000000000	
	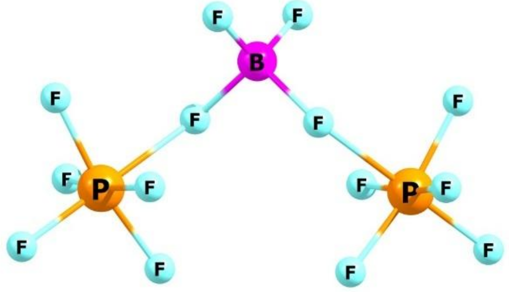				**1.1**
	F	3.655735000	-1.332499000	0.521843000	
	F	3.151731000	0.993093000	0.327671000	
	F	-1.658383000	-0.356874000	-1.423245000	
	F	0.965939000	0.658061000	-0.690751000	
	F	-0.626309000	2.299424000	-0.982635000	
	F	-3.655344000	-1.332828000	-0.522041000	
	F	-1.494726000	-1.678812000	0.440190000	
	F	1.658641000	-0.357002000	1.423324000	
	F	0.626136000	2.299293000	0.982719000	
	F	-2.969717000	-0.317535000	1.537806000	
	F	-3.151980000	0.992866000	-0.327783000	
	F	-0.966386000	0.658243000	0.690880000	
	B	-0.000092000	1.587296000	-0.000007000	
	P	2.425052000	-0.423296000	-0.006090000	
	P	-2.424943000	-0.423330000	0.006095000	
	F	1.495016000	-1.678925000	-0.440098000	
	F	2.969517000	-0.317293000	-1.537884000	

**$BP_2F_{14}^-$**	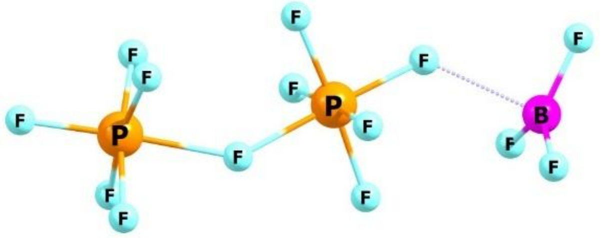	**2.1**

P	-0.670642000	0.208618000	-0.073116000
P	2.824918000	-0.094455000	0.029097000
F	0.979051000	-0.116899000	0.573056000
F	4.399751000	-0.093331000	-0.360042000
F	-1.193073000	-0.895053000	1.008809000
F	-0.435537000	-0.976609000	-1.162006000
F	2.722232000	-1.715265000	0.153068000
F	-2.213682000	0.497520000	-0.622240000
F	2.673905000	1.522967000	-0.018763000
F	3.091689000	-0.008244000	1.634688000
F	-0.040350000	1.292946000	-1.104100000
F	-0.793324000	1.368224000	1.067437000
F	2.304263000	-0.188270000	-1.502578000
B	-4.161357000	-0.224066000	0.093361000
F	-4.028154000	0.219775000	1.353298000
F	-4.801288000	0.545221000	-0.810764000
F	-3.944077000	-1.518772000	-0.188367000

**$BAs_2F_{14}^-$**	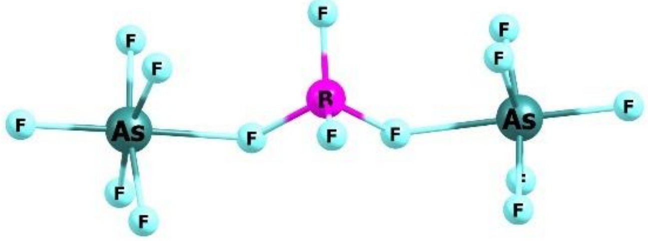	**0.0**

F	1.862141000	3.180291000	0.000000000
F	0.149245000	2.955958000	1.730595000
F	-1.249992000	-0.000004000	-1.173608000
F	-1.560714000	2.692447000	0.000000000
F	-1.561069000	-2.693639000	0.000000000
F	-0.111759000	-4.802472000	0.000000000
F	0.149245000	-2.955879000	-1.730662000
F	0.453457000	1.135882000	0.000000000
F	-1.249992000	-0.000004000	1.173608000
F	1.862335000	-3.180036000	0.000000000
F	0.149245000	-2.955879000	1.730662000
F	0.453145000	-1.136633000	0.000000000
B	-0.572698000	0.000255000	0.000000000
As	0.127612000	3.089622000	0.000000000
As	0.127726000	-3.089272000	0.000000000
F	0.149245000	2.955958000	-1.730595000
F	-0.112606000	4.802586000	0.000000000

$BAs_2F_{14}^-$	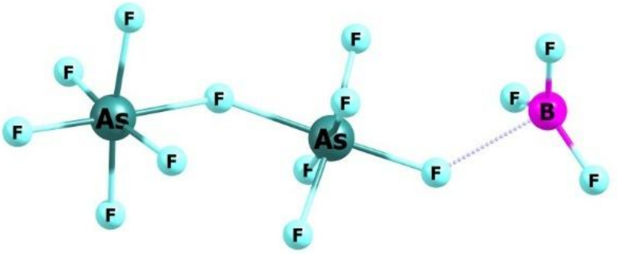	**0.6**

As	-0.918161000	0.185559000	-0.069655000
As	2.724000000	-0.084756000	0.030346000
F	0.836287000	-0.173315000	0.580699000
F	4.404037000	-0.027000000	-0.392466000
F	-1.446500000	-1.018273000	1.066966000
F	-0.672081000	-1.052913000	-1.258016000
F	2.672173000	-1.820548000	0.118712000
F	-2.564506000	0.501261000	-0.621588000
F	2.525068000	1.639357000	0.020465000
F	3.022389000	-0.027043000	1.743323000
F	-0.254563000	1.365721000	-1.148585000
F	-1.023194000	1.390639000	1.177729000
F	2.170575000	-0.160647000	-1.609412000
B	-4.505015000	-0.254083000	0.109828000
F	-4.344585000	0.160368000	1.376519000
F	-5.168024000	0.531716000	-0.761749000
F	-4.275700000	-1.537776000	-0.209481000

$BAs_2F_{14}^-$	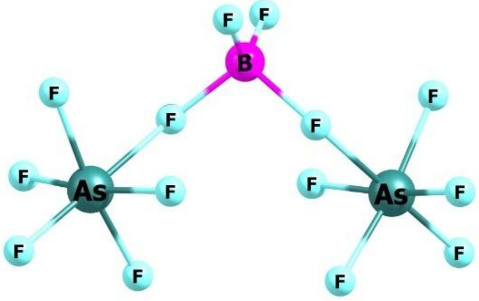	**1.2**

F	3.829309000	-1.290360000	0.549598000
F	3.251180000	1.178797000	0.339811000
F	-1.688435000	-0.282869000	-1.530720000
F	0.964243000	0.738551000	-0.690007000
F	-0.630702000	2.383331000	-0.981262000
F	-3.828984000	-1.290718000	-0.549795000
F	-1.521566000	-1.706216000	0.452530000
F	1.688834000	-0.282811000	1.530910000
F	0.630875000	2.383298000	0.981198000
F	-3.055307000	-0.226662000	1.644057000
F	-3.251393000	1.178596000	-0.340230000
F	-0.964643000	0.738995000	0.690237000
B	-0.000036000	1.681138000	0.000022000
As	2.491389000	-0.343989000	-0.004107000
As	-2.491392000	-0.343995000	0.004096000
F	1.521735000	-1.706488000	-0.452075000
F	3.054884000	-0.226802000	-1.644227000

$AlP_2F_{14}^-$	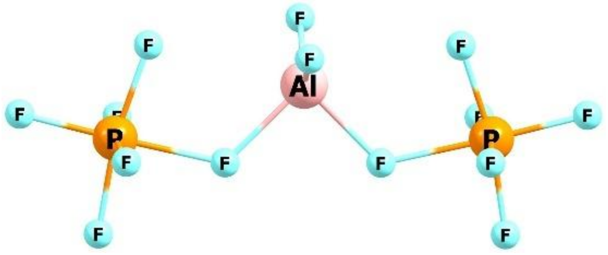	**0.0**
	F -1.845604000 -3.339692000 0.000000000<br>F -0.256423000 -2.979740000 1.624484000<br>F 1.440990000 0.000576000 -1.483041000<br>F 1.323806000 -2.562161000 0.000000000<br>F 1.322957000 2.558508000 0.000000000<br>F 0.162923000 4.654776000 0.000000000<br>F -0.256423000 2.979491000 -1.624476000<br>F -0.645071000 -1.278603000 0.000000000<br>F 1.440990000 0.000576000 1.483041000<br>F -1.845162000 3.342301000 0.000000000<br>F -0.256423000 2.979491000 1.624476000<br>F -0.647651000 1.279127000 0.000000000<br>Al 0.658832000 0.001370000 0.000000000<br>P -0.238097000 -3.087050000 0.000000000<br>P -0.238193000 3.086597000 0.000000000<br>F -0.256423000 -2.979740000 -1.624484000<br>F 0.159686000 -4.656137000 0.000000000	
	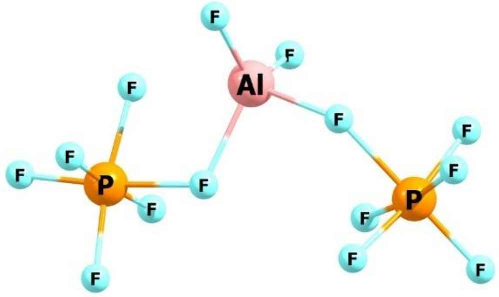	**0.2**
	P -2.602039000 -0.499881000 -0.001829000<br>P 2.692658000 -0.400441000 -0.020862000<br>Al -0.212911000 1.324997000 0.011387000<br>F 3.636142000 0.175562000 -1.220891000<br>F -0.833029000 -0.384853000 -0.358135000<br>F 1.584891000 -0.814976000 1.109274000<br>F 3.771615000 -1.514036000 0.458106000<br>F -2.179124000 -0.922252000 1.508296000<br>F -2.849124000 -0.010134000 -1.532523000<br>F 0.011154000 1.780122000 1.613601000<br>F -4.176670000 -0.452632000 0.357525000<br>F -0.591107000 2.447883000 -1.189162000<br>F -2.565888000 -2.046592000 -0.475603000<br>F 1.466364000 0.865299000 -0.573094000<br>F 1.966720000 -1.403938000 -1.083910000<br>F 3.272764000 0.749787000 0.978325000<br>F -2.358202000 1.117413000 0.429561000	

$AlP_2F_{14}^-$

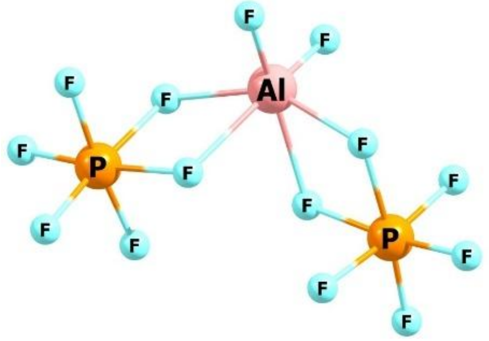

1.0

F	2.894969000	-1.670313000	0.931794000
F	3.082472000	0.656007000	0.876092000
F	-0.940049000	-0.272615000	-0.908993000
F	1.612860000	0.820292000	-0.952275000
F	-0.580621000	2.330904000	-1.254359000
F	-2.895069000	-1.670329000	-0.931724000
F	-1.542271000	-1.528103000	0.965050000
F	0.940000000	-0.272554000	0.908946000
F	0.580675000	2.330993000	1.254294000
F	-3.617772000	-0.501760000	1.060062000
F	-3.082500000	0.656027000	-0.876078000
F	-1.612801000	0.820318000	0.952234000
Al	0.000020000	1.343223000	0.000006000
P	2.363956000	-0.482734000	-0.030869000
P	-2.363962000	-0.482722000	0.030859000
F	1.542265000	-1.528137000	-0.965034000
F	3.617824000	-0.501846000	-1.060000000

$AlAs_2F_{14}^-$

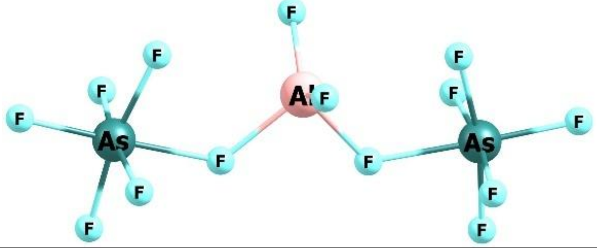

0.0

F	-1.915354000	-3.426241000	0.000000000
F	-0.218705000	-3.070910000	1.732250000
F	1.480737000	0.000448000	-1.483755000
F	1.462393000	-2.624649000	0.000000000
F	1.462120000	2.623310000	0.000000000
F	0.213128000	4.864910000	0.000000000
F	-0.218705000	3.070962000	-1.732341000
F	-0.590058000	-1.287421000	0.000000000
F	1.480737000	0.000448000	1.483755000
F	-1.915282000	3.427315000	0.000000000
F	-0.218705000	3.070962000	1.732341000
F	-0.590946000	1.288213000	0.000000000
Al	0.702974000	0.000460000	0.000000000
As	-0.196331000	-3.185673000	0.000000000
As	-0.196444000	3.185245000	0.000000000
F	-0.218705000	-3.070910000	-1.732250000
F	0.212114000	-4.865533000	0.000000000

$AlAs_2F_{14}^-$

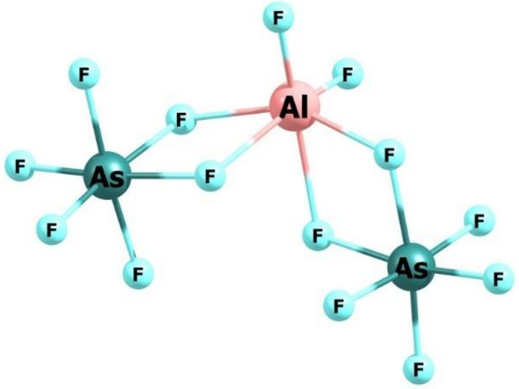

1.1

F	2.972606000	-1.680387000	1.007852000
F	3.198533000	0.795963000	0.943963000
F	-0.903794000	-0.154751000	-0.942000000
F	1.611136000	0.967764000	-0.973860000
F	-0.614627000	2.457316000	-1.238582000
F	-2.972651000	-1.680393000	-1.007821000
F	-1.548429000	-1.522901000	1.025581000
F	0.903792000	-0.154725000	0.941999000
F	0.614630000	2.457359000	1.238559000
F	-3.755978000	-0.424136000	1.129551000
F	-3.198522000	0.795963000	-0.943978000
F	-1.611125000	0.967774000	0.973850000
Al	0.000006000	1.470588000	0.000004000
As	2.421722000	-0.409360000	-0.025708000
As	-2.421721000	-0.409356000	0.025708000
F	1.548424000	-1.522903000	-1.025579000
F	3.755990000	-0.424168000	-1.129539000

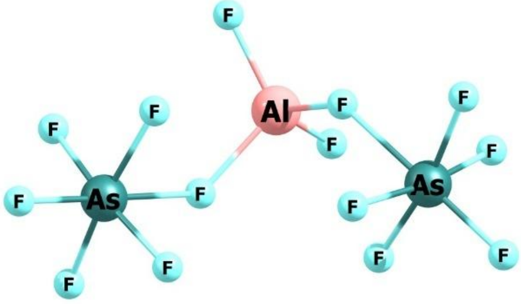

1.8

F	-0.701287000	-3.736999000	-1.512219000
F	-0.383090000	-3.417466000	0.987471000
F	1.460603000	1.619833000	-0.279454000
F	0.776030000	-1.196811000	0.725888000
F	1.250763000	0.693096000	2.698781000
F	0.701287000	3.736999000	-1.512219000
F	-0.492114000	1.503145000	-1.757230000
F	-1.460603000	-1.619833000	-0.279454000
F	-1.250763000	-0.693096000	2.698781000
F	-1.584385000	3.262392000	-0.453404000
F	0.383090000	3.417466000	0.987471000
F	-0.776030000	1.196811000	0.725888000
Al	0.000000000	0.000000000	1.815706000
As	0.000000000	-2.550043000	-0.469412000
As	0.000000000	2.550043000	-0.469412000
F	0.492114000	-1.503145000	-1.757230000

	F 1.584385000 -3.262392000 -0.453404000	
$BPAsF_{14}^-$	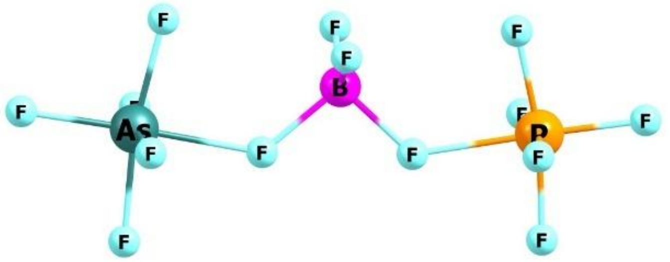	**0.0**
	F -1.873391000 -2.854898000 0.000000000<br>F -0.160239000 -2.622800000 1.731974000<br>F 1.232157000 0.331684000 -1.173095000<br>F 1.550813000 -2.355436000 0.000000000<br>F 1.439575000 2.986338000 0.000000000<br>F 0.093112000 4.970882000 0.000000000<br>F -0.160239000 3.233371000 -1.619602000<br>F -0.471629000 -0.810396000 0.000000000<br>F 1.232157000 0.331684000 1.173095000<br>F -1.763596000 3.456584000 0.000000000<br>F -0.160239000 3.233371000 1.619602000<br>F -0.471648000 1.460788000 0.000000000<br>B 0.550602000 0.345316000 0.000000000<br>As -0.138484000 -2.750128000 0.000000000<br>P -0.139124000 3.370521000 0.000000000<br>F -0.160239000 -2.622800000 -1.731974000<br>F 0.107166000 -4.463944000 0.000000000	
	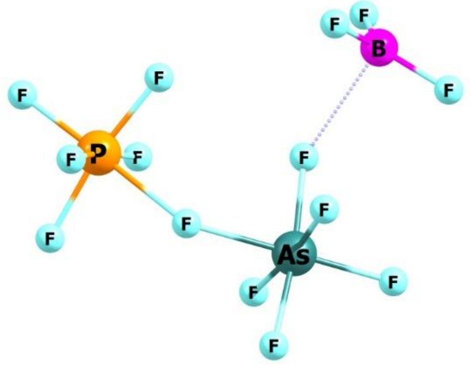	**0.7**
	P -2.316546000 0.449223000 0.002316000<br>As 0.876993000 -1.211448000 0.006654000<br>F -0.889857000 -0.752363000 -0.488203000<br>F 2.502933000 -1.643372000 0.433847000<br>F -2.784245000 0.220234000 -1.542059000<br>F -1.325153000 1.637985000 -0.485732000<br>F 1.345108000 -0.615076000 -1.557795000<br>F -3.550822000 1.434904000 0.366374000<br>F 0.239441000 -1.717380000 1.538785000<br>F 0.524737000 -2.771610000 -0.677372000<br>F -1.651319000 0.503503000 1.480557000<br>F -3.107403000 -0.912409000 0.421250000<br>F 1.058597000 0.419979000 0.649409000<br>B 2.282892000 2.154374000 -0.030820000<br>F 1.540078000 2.456638000 -1.103653000<br>F 2.059592000 2.790910000 1.134250000<br>F 3.415310000 1.444456000 -0.180798000	

$BPAsF_{14}^{-}$

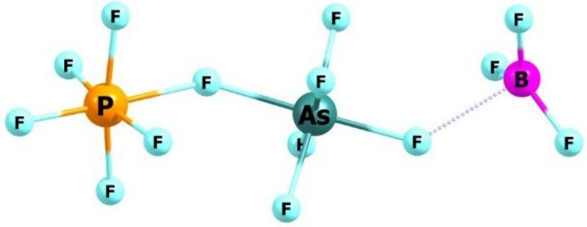

1.0

As	-0.614711000	0.175921000	-0.064454000
P	2.963622000	-0.104308000	0.035415000
F	1.118717000	-0.143258000	0.602567000
F	4.529425000	-0.086721000	-0.382073000
F	-1.155657000	-0.984698000	1.112308000
F	-0.372115000	-1.104818000	-1.209745000
F	2.883011000	-1.723603000	0.186210000
F	-2.266240000	0.462833000	-0.636476000
F	2.784509000	1.509294000	-0.033586000
F	3.251909000	0.010336000	1.634935000
F	0.030348000	1.322181000	-1.192429000
F	-0.744719000	1.431876000	1.131441000
F	2.411493000	-0.228445000	-1.483624000
B	-4.176563000	-0.238353000	0.101412000
F	-4.031691000	0.224834000	1.354262000
F	-4.841071000	0.510583000	-0.803228000
F	-3.963036000	-1.539173000	-0.159596000

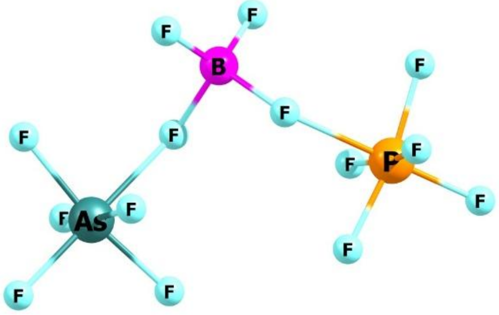

1.1

F	3.578196000	-1.241796000	0.552080000
F	2.957198000	1.211455000	0.323822000
F	-1.926147000	-0.369045000	-1.418553000
F	0.681429000	0.711134000	-0.689820000
F	-0.954172000	2.312849000	-1.000159000
F	-3.906314000	-1.388854000	-0.517734000
F	-1.735076000	-1.669439000	0.453165000
F	1.427286000	-0.270875000	1.537055000
F	0.305046000	2.371511000	0.961495000
F	-3.242258000	-0.336612000	1.535300000
F	-3.450668000	0.952070000	-0.338257000
F	-1.247917000	0.688290000	0.692668000
B	-0.323552000	1.634809000	0.001358000
As	2.218266000	-0.325739000	-0.005017000
P	-2.700353000	-0.448410000	0.004742000
F	1.280336000	-1.718356000	-0.433542000
F	2.779761000	-0.218834000	-1.647783000

<table>
<tr>
<td rowspan="2">$BPAsF_{14}^{-}$</td>
<td>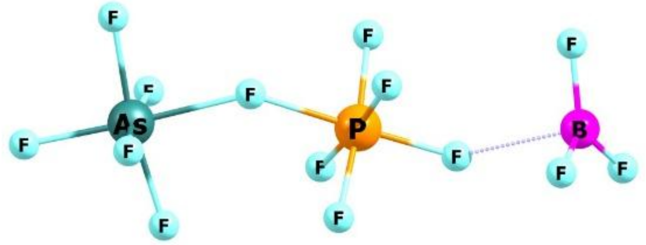
</td>
<td>1.6</td>
</tr>
<tr>
<td>P -0.990816000 0.213380000 -0.080186000
As 2.568075000 -0.074208000 0.025591000
F 0.682232000 -0.152704000 0.557816000
F 4.256343000 -0.028994000 -0.371722000
F -1.505184000 -0.922533000 0.969308000
F -0.752478000 -0.938722000 -1.201186000
F 2.504426000 -1.810965000 0.100113000
F -2.526129000 0.529647000 -0.614079000
F 2.390540000 1.652600000 0.023921000
F 2.849198000 -0.030893000 1.742669000
F -0.339264000 1.320505000 -1.070139000
F -1.088783000 1.331272000 1.100838000
F 2.046836000 -0.134655000 -1.625311000
B -4.507011000 -0.231618000 0.102049000
F -4.356212000 0.173849000 1.371756000
F -5.143168000 0.569084000 -0.774890000
F -4.279375000 -1.512351000 -0.225977000</td>
<td></td>
</tr>
<tr>
<td rowspan="2">$AlPAsF_{14}^{-}$</td>
<td>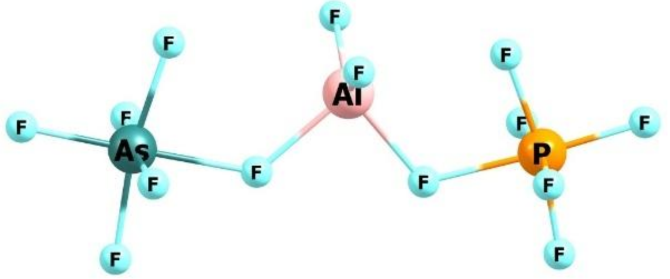
</td>
<td>0.0</td>
</tr>
<tr>
<td>F -1.943486000 -3.013410000 0.000000000
F -0.236455000 -2.718665000 1.733505000
F 1.463185000 0.354657000 -1.488261000
F 1.464056000 -2.350639000 0.000000000
F 1.319738000 2.780193000 0.000000000
F 0.272365000 4.939457000 0.000000000
F -0.236455000 3.296639000 -1.623498000
F -0.555105000 -0.927578000 0.000000000
F 1.463185000 0.354657000 1.488261000
F -1.810561000 3.735641000 0.000000000
F -0.236455000 3.296639000 1.623498000
F -0.690941000 1.619440000 0.000000000
Al 0.693073000 0.412332000 0.000000000
As -0.215169000 -2.831255000 0.000000000
P -0.221323000 3.400076000 0.000000000
F -0.236455000 -2.718665000 -1.733505000
F 0.120103000 -4.529482000 0.000000000</td>
<td></td>
</tr>
</table>

$AlPAsF_{14}^{-}$

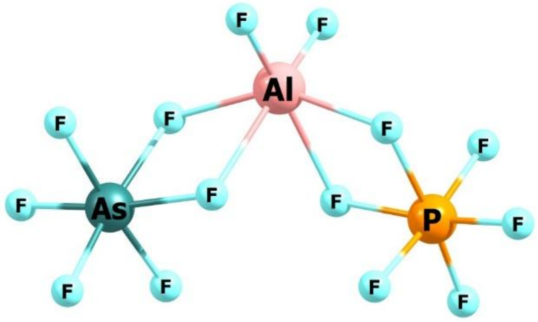

1.1

F	2.808115000	-1.605220000	1.017839000
F	2.922831000	0.879016000	0.922379000
F	-1.169336000	-0.264296000	-0.894029000
F	1.308013000	0.956844000	-0.978795000
F	-0.957810000	2.357257000	-1.256133000
F	-3.061965000	-1.746484000	-0.920568000
F	-1.739622000	-1.524745000	0.988565000
F	0.673955000	-0.173289000	0.957950000
F	0.238919000	2.416798000	1.235924000
F	-3.858861000	-0.588965000	1.050351000
F	-3.352080000	0.568656000	-0.893799000
F	-1.911631000	0.818242000	0.947663000
Al	-0.318709000	1.400950000	-0.005929000
As	2.188070000	-0.372043000	-0.022799000
P	-2.595888000	-0.526505000	0.034719000
F	1.353846000	-1.534243000	-1.000980000
F	3.509542000	-0.341503000	-1.142070000

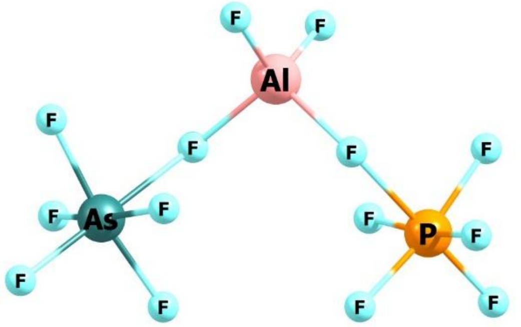

1.9

F	-3.766487000	-1.651847000	-0.656626000
F	-3.624705000	0.702755000	-0.333688000
F	1.387839000	-0.288975000	1.460235000
F	-1.509103000	0.615033000	0.798035000
F	0.306643000	2.655119000	1.236197000
F	3.558549000	-1.399717000	0.669046000
F	1.326855000	-1.771181000	-0.492770000
F	-1.863773000	-0.366392000	-1.347937000
F	-1.084764000	2.591577000	-1.257021000
F	2.994344000	-0.370561000	-1.608197000
F	3.096172000	1.077794000	0.357639000
F	0.878926000	0.690822000	-0.772254000
Al	-0.363364000	1.735456000	-0.002244000
P	-2.728581000	-0.612075000	0.019264000
As	2.304504000	-0.424512000	-0.014169000
F	-1.669701000	-1.753965000	0.472703000
F	-3.408150000	-0.660562000	1.497726000

$B_3PF_{15}^-$

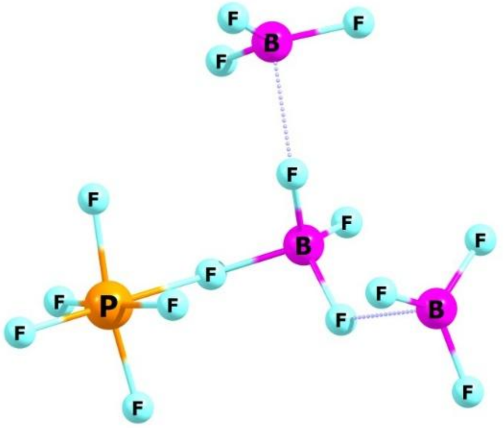

0.0

B	-2.714233000	-1.868582000	0.387858000
B	-0.648113000	-0.003326000	-0.880339000
B	-0.955128000	2.863686000	0.282326000
P	2.270428000	-0.515432000	0.115865000
F	-3.488026000	-0.847866000	-0.038701000
F	3.496803000	-0.911400000	1.099610000
F	-2.842165000	-3.074617000	-0.210207000
F	0.873718000	-0.041279000	-1.085125000
F	-1.041733000	-1.349671000	-0.685935000
F	-0.684261000	2.955965000	1.599075000
F	-2.156855000	-1.805230000	1.611185000
F	0.032188000	3.046859000	-0.613443000
F	-0.891544000	0.764360000	0.274004000
F	2.264420000	-1.950465000	-0.657939000
F	3.234142000	0.089985000	-1.051608000
F	1.110530000	-1.059647000	1.113982000
F	-1.143257000	0.546311000	-2.036299000
F	-2.237934000	2.964802000	-0.122654000
F	2.088525000	0.979959000	0.727700000

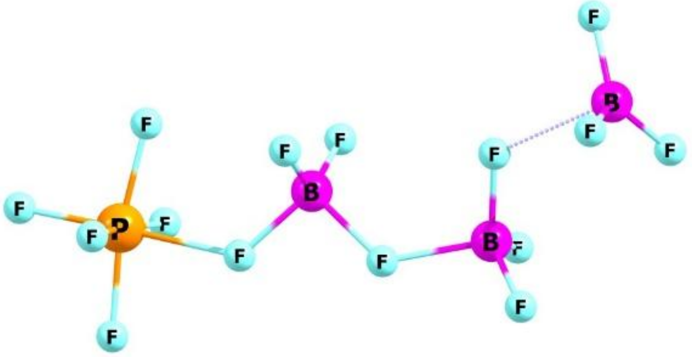

0.8

B	-2.160697000	1.122771000	-0.041823000
B	-4.658628000	-0.745957000	0.027290000
B	0.326310000	-0.171249000	-0.013694000
P	3.430503000	-0.109556000	-0.000129000
F	-4.500841000	-1.809028000	0.837407000
F	1.639737000	0.541362000	-0.289156000
F	0.150226000	-0.329806000	1.325441000
F	-5.103016000	0.416825000	0.543053000
F	-2.071033000	1.823477000	1.126222000
F	4.950698000	-0.615687000	0.225176000
F	-4.702060000	-0.934477000	-1.303700000

$B_3PF_{15}^-$	F	0.209251000	-1.257151000	-0.825655000	
	F	2.735556000	-1.451453000	0.596574000	
	F	-2.589368000	-0.210036000	0.102873000	
	F	3.304136000	0.593159000	1.460787000	
	F	-0.592762000	0.923880000	-0.523523000	
	F	-2.696862000	1.774810000	-1.119487000	
	F	3.305080000	-0.724611000	-1.500585000	
	F	3.850984000	1.327127000	-0.639530000	
	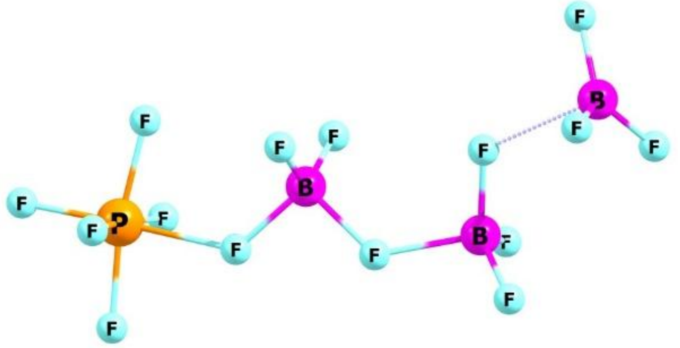				**1.0**
	B	-1.089201000	-0.724760000	-0.138072000	
	B	2.918871000	-1.766345000	0.007399000	
	B	-4.002413000	-0.210770000	0.084373000	
	P	1.205693000	1.411705000	0.016805000	
	F	-0.590988000	-1.300541000	0.991919000	
	F	0.387953000	2.556042000	-0.803207000	
	F	0.152368000	1.514974000	1.249175000	
	F	0.081838000	0.203545000	-0.749712000	
	F	4.055878000	-1.146814000	-0.355069000	
	F	-4.396082000	0.786946000	0.914362000	
	F	-4.223494000	-0.059814000	-1.243323000	
	F	2.150657000	1.139413000	-1.283142000	
	F	2.245774000	2.469826000	0.676814000	
	F	-3.916793000	-1.468165000	0.585590000	
	F	1.985580000	-2.037339000	-0.912991000	
	F	-2.120736000	0.234395000	0.117414000	
	F	-1.380623000	-1.544912000	-1.193555000	
	F	2.843366000	-2.323086000	1.228198000	
	F	1.922893000	0.123730000	0.775242000	
	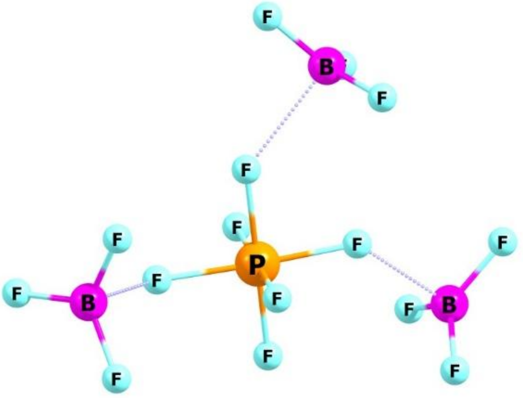				**1.1**
	B	3.070695000	-1.482368000	-0.206188000	
	B	-3.618774000	-0.462638000	-0.266690000	
	B	0.955979000	2.820016000	-0.084628000	

	Atom	X	Y	Z	
	P	-0.205709000	-0.441505000	0.285754000	
	F	-4.337125000	-0.470641000	0.879064000	
	F	2.637557000	-2.323834000	-1.163764000	
	F	-3.493280000	-1.612993000	-0.954858000	
	F	3.893099000	-0.469797000	-0.554007000	
	F	-1.803300000	-0.593310000	0.786050000	
	F	1.381822000	-0.218599000	-0.210604000	
	F	-0.674602000	-0.591969000	-1.272075000	
	F	1.774213000	2.484725000	0.927372000	
	F	-3.382261000	0.710780000	-0.879125000	
	F	3.002866000	-1.854542000	1.084812000	
	F	-0.005362000	3.743845000	0.130745000	
	F	0.016192000	-2.054405000	0.403856000	
	F	-0.439204000	1.216172000	0.164110000	
	F	0.247188000	-0.238616000	1.842200000	
	F	1.298433000	2.522909000	-1.350305000	
$B_3AsF_{15}^-$	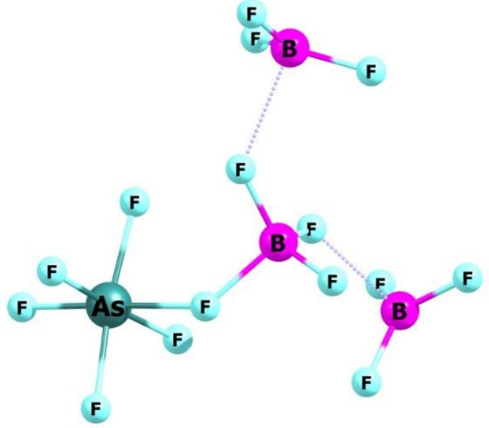				**0.0**
	B	2.850646000	2.029164000	0.408651000	
	B	0.921136000	-0.003056000	-0.914338000	
	B	1.373357000	-2.863343000	0.297191000	
	As	-2.092520000	0.336591000	0.089584000	
	F	3.692254000	1.055334000	0.007511000	
	F	-3.436559000	0.686677000	1.127098000	
	F	2.902032000	3.229486000	-0.206865000	
	F	-0.620244000	-0.069864000	-1.126672000	
	F	1.213553000	1.364963000	-0.749803000	
	F	1.101121000	-2.941288000	1.612801000	
	F	2.247120000	1.931180000	1.604337000	
	F	0.402932000	-3.103281000	-0.601319000	
	F	1.190148000	-0.733747000	0.253008000	
	F	-2.207902000	1.845386000	-0.770008000	
	F	-3.071914000	-0.414267000	-1.137480000	
	F	-0.910432000	1.038138000	1.146408000	
	F	1.440997000	-0.548065000	-2.060361000	
	F	2.661071000	-2.887002000	-0.101177000	
	F	-1.790014000	-1.222685000	0.789878000	

$B_3AsF_{15}^-$

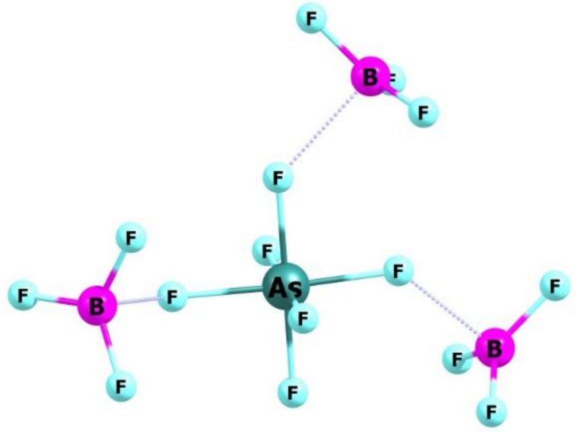

0.4

B	-3.181205000	-1.428370000	0.281560000
B	3.657568000	-0.444819000	0.336474000
B	-0.924976000	2.937127000	0.102914000
As	0.177826000	-0.421865000	-0.289841000
F	4.421557000	-0.476344000	-0.779260000
F	-2.715762000	-2.212539000	1.272391000
F	3.512127000	-1.578804000	1.048691000
F	-4.016836000	-0.414026000	0.591444000
F	1.888487000	-0.607941000	-0.763404000
F	-1.519770000	-0.151524000	0.175575000
F	0.623503000	-0.558420000	1.386333000
F	-1.742999000	2.633403000	-0.920470000
F	3.394151000	0.741904000	0.912778000
F	-3.133067000	-1.868583000	-0.989788000
F	0.040291000	3.863135000	-0.085533000
F	-0.075416000	-2.139269000	-0.397401000
F	0.442640000	1.336657000	-0.181800000
F	-0.244336000	-0.224074000	-1.966870000
F	-1.277372000	2.612189000	1.359537000

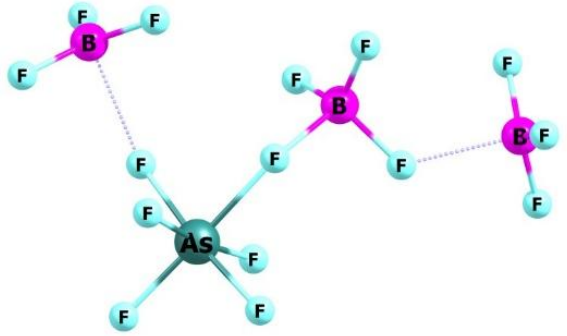

0.6

B	1.285129000	0.817163000	-0.142922000
B	-2.692763000	2.095360000	0.008528000
B	4.225832000	0.171216000	0.085045000
As	-1.185155000	-1.251645000	0.014261000
F	0.778551000	1.416785000	0.970869000
F	-0.378282000	-2.531421000	-0.840259000
F	-0.089277000	-1.416327000	1.347044000
F	0.064582000	-0.069608000	-0.774350000
F	-3.876322000	1.572710000	-0.360455000
F	4.545829000	-0.841375000	0.922330000
F	4.409970000	-0.005539000	-1.241244000
F	-2.160507000	-0.912786000	-1.384121000

	F	-2.368653000	-2.309975000	0.713055000	
	F	4.183620000	1.431211000	0.575226000	
	F	-1.730908000	2.273885000	-0.906394000	
	F	2.255195000	-0.184911000	0.130492000	
	F	1.618418000	1.617514000	-1.200768000	
	F	-2.587320000	2.672908000	1.218248000	
	F	-1.884994000	0.163106000	0.805450000	
**$B_3AsF_{15}^-$**	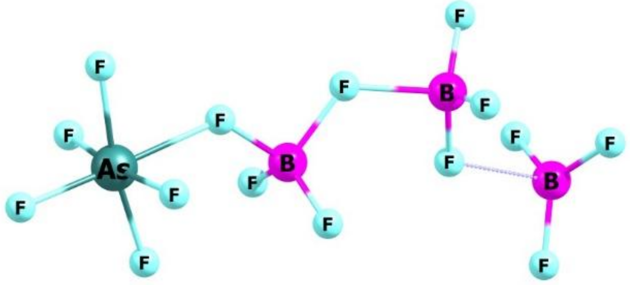				**1.0**
	B	2.543292000	1.133091000	0.042336000	
	B	5.050568000	-0.762600000	-0.025873000	
	B	0.047934000	-0.151998000	0.008433000	
	As	-3.125357000	-0.089513000	0.001629000	
	F	4.880993000	-1.813509000	-0.847264000	
	F	-1.285705000	0.561417000	0.286644000	
	F	0.207070000	-0.304756000	-1.332121000	
	F	5.494106000	0.405284000	-0.528255000	
	F	2.451715000	1.842771000	-1.118060000	
	F	-4.756365000	-0.619354000	-0.230736000	
	F	5.078605000	-0.961556000	1.302791000	
	F	0.148013000	-1.241066000	0.815907000	
	F	-2.401994000	-1.527078000	-0.644946000	
	F	2.953744000	-0.200085000	-0.106588000	
	F	-3.007406000	0.662366000	-1.558999000	
	F	0.951118000	0.938365000	0.519875000	
	F	3.065614000	1.774755000	1.130496000	
	F	-2.998137000	-0.754321000	1.600913000	
	F	-3.567171000	1.443597000	0.690542000	
**$Al_3PF_{15}^-$**	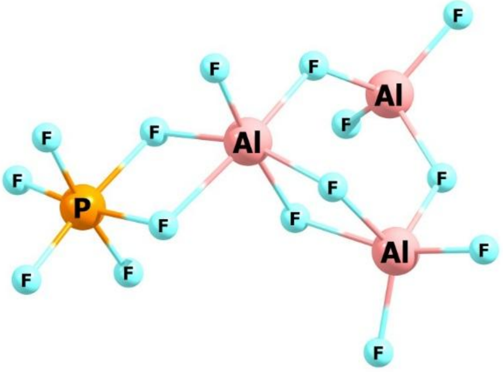				**0.0**
	Al	2.412684000	-1.592331000	-0.315163000	
	Al	1.947950000	1.660971000	-0.212661000	
	Al	-0.183049000	-0.031699000	0.939051000	
	P	-2.848807000	-0.072967000	-0.281836000	
	F	0.943973000	1.392224000	1.320248000	
	F	-1.497961000	-1.160074000	0.040504000	
	F	-0.686504000	-0.366002000	2.523754000	
	F	3.225489000	2.466264000	0.585246000	

$Al_3PF_{15}^-$	F 3.666268000 -2.450472000 0.415827000<br>F -3.956583000 1.085706000 -0.480328000<br>F 1.517829000 2.633062000 -1.532671000<br>F 0.542233000 0.359709000 -0.703627000<br>F -1.740619000 1.068838000 0.465858000<br>F 2.973361000 0.098440000 -0.480431000<br>F -3.390496000 -0.417643000 1.204970000<br>F 1.124906000 -1.411281000 0.909881000<br>F 1.836296000 -2.218645000 -1.763998000<br>F -2.145445000 0.271259000 -1.697445000<br>F -3.699026000 -1.283131000 -0.932055000	
	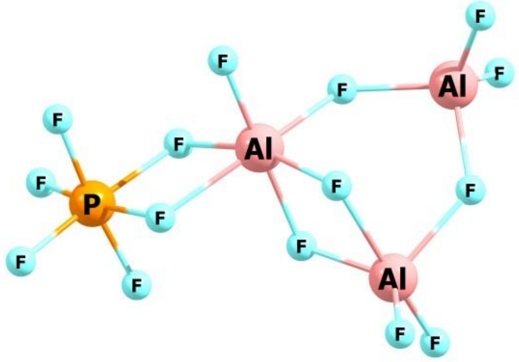	**1.5**
	Al -1.663346000 1.793675000 -0.097313000<br>Al -2.921009000 -1.264721000 0.324724000<br>Al 0.186061000 -0.471130000 -0.545890000<br>P 2.992023000 -0.082253000 0.232084000<br>F -1.040739000 0.485325000 -1.461469000<br>F 1.842402000 0.527346000 -0.946576000<br>F -0.207895000 0.941366000 0.639280000<br>F 4.168200000 0.830012000 -0.395992000<br>F -3.024188000 0.503056000 0.100167000<br>F -1.149478000 -1.440586000 0.397464000<br>F 1.561174000 -0.978790000 0.746245000<br>F 3.305364000 -1.296069000 -0.790315000<br>F 2.515558000 1.111326000 1.218333000<br>F -3.552737000 -1.680076000 1.827836000<br>F -2.095652000 2.621626000 1.333670000<br>F -3.572775000 -2.039222000 -1.016482000<br>F 0.519318000 -1.716308000 -1.650018000<br>F 3.868775000 -0.769356000 1.403297000<br>F -1.770942000 2.953914000 -1.332223000	
	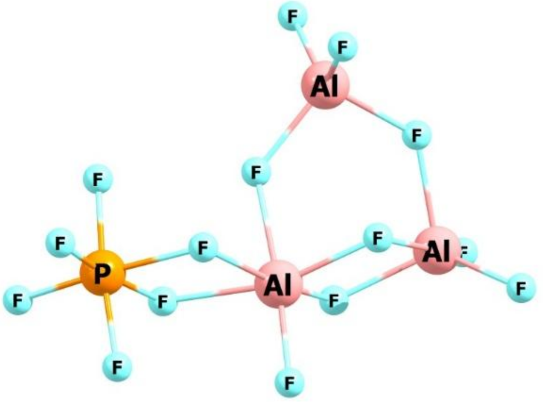	**3.8**
	Al 0.130339000 -0.889234000 0.130391000	

$Al_3PF_{15}^-$

Al	-2.782560000	-1.028424000	-0.135862000
Al	-1.562840000	1.985483000	0.129120000
P	2.914260000	0.020337000	-0.096095000
F	0.590639000	-2.456273000	0.598203000
F	-3.515610000	-1.973902000	1.063623000
F	-2.928589000	0.836242000	-0.034090000
F	-1.366927000	2.477856000	1.725029000
F	1.630720000	-0.654601000	-1.106021000
F	-1.209610000	-1.355089000	-1.058939000
F	-3.777950000	-1.152410000	-1.518479000
F	-0.242240000	0.942574000	-0.430200000
F	3.280824000	-1.490959000	0.340881000
F	3.946794000	-0.027730000	-1.338045000
F	-1.301692000	-0.600181000	1.189470000
F	-1.803169000	3.205441000	-1.008039000
F	1.610961000	-0.020068000	1.088789000
F	2.390238000	1.492299000	-0.526833000
F	3.926934000	0.644937000	0.996204000

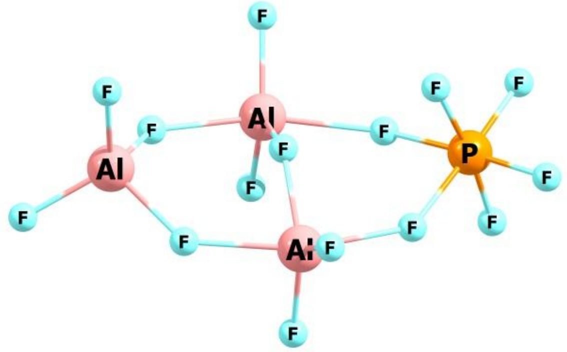

5.9

Al	-0.391153000	-1.658769000	0.256031000
Al	-0.391320000	1.659223000	0.255883000
Al	-3.303957000	-0.000456000	-0.277380000
P	2.728672000	-0.000128000	-0.168671000
F	-0.433246000	0.000175000	-0.493999000
F	-0.392766000	1.837571000	1.931578000
F	-0.397599000	-1.833894000	1.932087000
F	1.571606000	-1.209055000	0.323874000
F	3.746237000	-1.190084000	-0.586313000
F	-2.308416000	1.407499000	0.174141000
F	-2.308403000	-1.410433000	0.167697000
F	1.572416000	1.211659000	0.318500000
F	-0.253141000	2.917245000	-0.851902000
F	-3.518394000	0.003156000	-1.948425000
F	-4.690008000	-0.002448000	0.680360000
F	-0.248289000	-2.918138000	-0.849623000
F	2.019041000	-0.003214000	-1.630271000
F	3.248689000	0.002918000	1.366177000
F	3.747107000	1.187259000	-0.591535000

$Al_3PF_{15}^-$

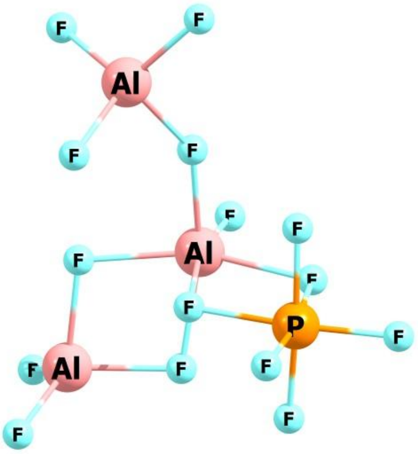

5.9

Al	2.185290000	-2.138410000	0.300903000
Al	-0.475991000	-0.259416000	-0.921736000
Al	-2.922778000	-0.764708000	0.432039000
P	0.720091000	2.227091000	0.121157000
F	1.731858000	-3.725407000	-0.095819000
F	1.036451000	2.752478000	1.608807000
F	3.765193000	-1.737331000	-0.164084000
F	0.267302000	1.442029000	-1.409010000
F	1.120852000	-1.098673000	-0.774159000
F	-3.211132000	-0.284194000	2.013871000
F	1.741600000	-1.663094000	1.866772000
F	-2.213077000	0.527324000	-0.572724000
F	-1.308736000	-1.525658000	0.209965000
F	2.133784000	1.448870000	0.011285000
F	1.294167000	3.501983000	-0.685574000
F	0.029406000	0.741417000	0.731033000
F	-0.903728000	-0.770751000	-2.479776000
F	-4.155589000	-1.608453000	-0.334927000
F	-0.775700000	2.855746000	0.145115000

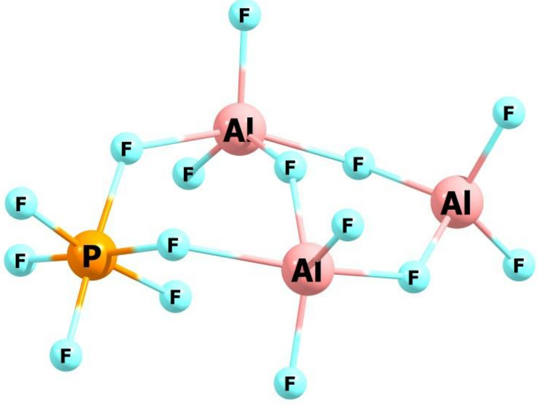

6.8

Al	0.284154000	1.743196000	0.296565000
Al	0.284753000	-1.743710000	0.296614000
Al	3.108618000	0.000285000	-0.337001000
P	-2.488355000	-0.000008000	-0.177859000
F	2.023506000	-1.411060000	-0.450908000
F	-1.194990000	0.000854000	-1.206203000
F	-0.218092000	-2.607298000	-1.065593000
F	3.872613000	0.002864000	1.162141000
F	0.600845000	-2.624033000	1.700231000

$Al_3PF_{15}^-$	F	2.020929000	1.409480000	-0.454519000	
	F	0.530829000	-0.000370000	0.835259000	
	F	-1.617456000	1.197924000	0.731870000	
	F	-3.224292000	1.180320000	-0.996136000	
	F	-0.220268000	2.609629000	-1.063277000	
	F	-3.588955000	-0.000938000	1.007620000	
	F	0.602669000	2.621865000	1.700691000	
	F	4.088963000	-0.000407000	-1.707589000	
	F	-3.223830000	-1.179591000	-0.997613000	
	F	-1.617194000	-1.198896000	0.730425000	
	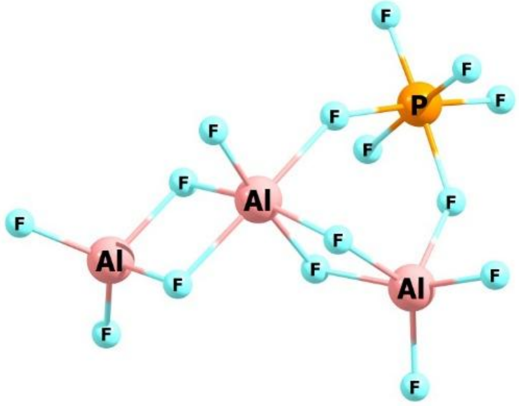				**7.3**
	Al	-3.433342000	-0.407097000	-0.403383000	
	Al	0.985829000	2.045069000	-0.100850000	
	Al	-0.914315000	0.052393000	0.796389000	
	P	2.214398000	-1.184189000	-0.222274000	
	F	0.547117000	3.167336000	-1.296479000	
	F	-4.675523000	-1.147264000	0.455016000	
	F	2.250980000	2.781816000	0.774514000	
	F	-2.626818000	0.900093000	0.512340000	
	F	2.143547000	0.546075000	-0.540544000	
	F	2.202838000	-2.762164000	0.131185000	
	F	3.027026000	-0.810255000	1.126248000	
	F	-1.865576000	-1.251783000	-0.253765000	
	F	0.011445000	1.547823000	1.390941000	
	F	-3.802048000	0.025907000	-1.985061000	
	F	-0.318547000	0.789467000	-0.763631000	
	F	3.564951000	-1.245521000	-1.109900000	
	F	0.757965000	-0.989188000	0.725493000	
	F	1.267242000	-1.390861000	-1.517983000	
	F	-1.319290000	-0.629471000	2.300079000	

$Al_3PF_{15}^-$

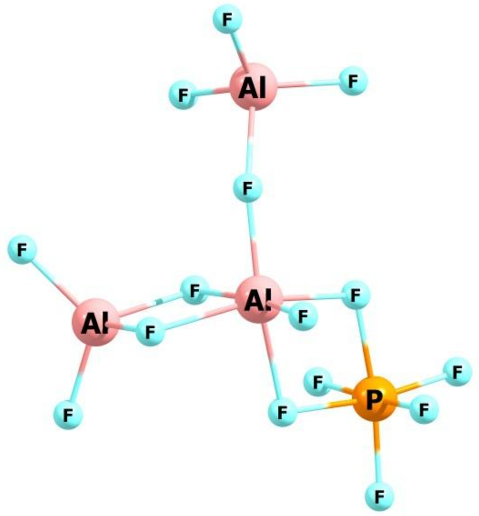

7.7

Al	-0.876860000	2.573873000	-0.201855000
Al	-2.826979000	-1.587570000	-0.308074000
Al	0.001850000	0.091220000	0.927973000
P	2.573596000	-0.628356000	-0.286375000
F	2.078805000	0.056670000	-1.668399000
F	0.251403000	3.752573000	-0.603050000
F	3.019549000	-1.976636000	-1.049418000
F	-4.156453000	-1.831825000	0.715386000
F	3.996052000	0.131686000	-0.347356000
F	0.359220000	-0.427803000	2.499003000
F	-1.627047000	-0.690533000	0.757482000
F	-3.105165000	-0.511229000	-1.588053000
F	-0.541474000	1.844409000	1.410527000
F	-0.398777000	0.953743000	-0.765866000
F	-2.493942000	2.979570000	-0.371756000
F	1.843269000	0.727557000	0.559974000
F	2.903436000	-1.267322000	1.162472000
F	0.924597000	-1.250045000	-0.096348000
F	-1.995482000	-2.999976000	-0.741147000

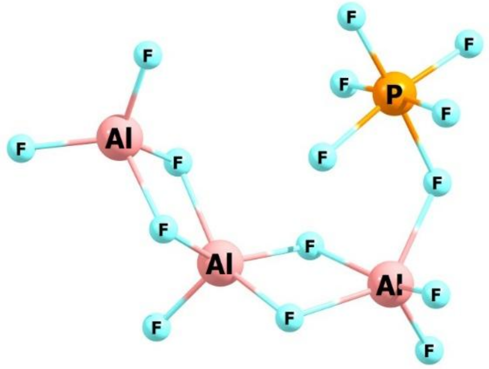

8.1

Al	2.910655000	0.955015000	0.202763000
Al	1.311128000	-1.354592000	-0.226173000
Al	-1.520398000	-1.711815000	0.223629000
P	-1.697932000	1.455768000	-0.155874000
F	-0.463476000	0.594045000	0.617309000
F	-2.275173000	-1.560653000	1.738918000
F	2.385859000	2.460944000	0.706530000
F	2.229292000	-0.420026000	1.143782000

Species	Atom	X	Y	Z	
$Al_3PF_{15}^-$	F	0.182908000	-2.043973000	1.005512000	
	F	-2.125596000	-3.062255000	-0.606997000	
	F	2.419181000	-2.526105000	-0.718337000	
	F	-0.191847000	-1.330423000	-1.174569000	
	F	-2.359248000	-0.170688000	-0.522143000	
	F	-2.546895000	1.408889000	1.225156000	
	F	-1.027402000	2.883181000	0.210605000	
	F	1.963549000	0.194454000	-1.115274000	
	F	4.563471000	0.772013000	-0.041127000	
	F	-0.858052000	1.340170000	-1.546710000	
	F	-2.968685000	2.083935000	-0.952071000	
	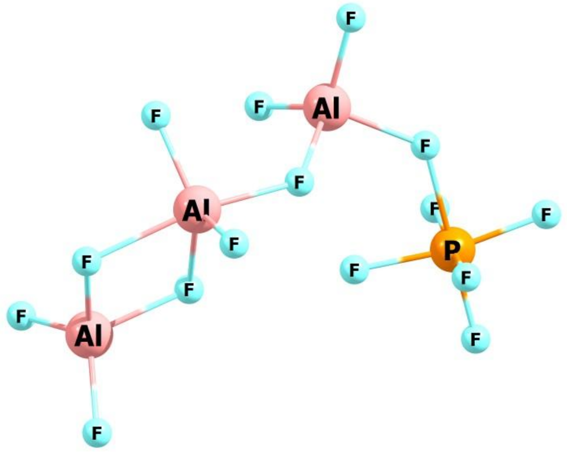				**9.7**
	Al	1.049956000	1.709701000	0.488262000	
	Al	-1.981332000	0.957225000	-0.684510000	
	Al	-3.497552000	-1.241146000	0.366738000	
	P	2.932977000	-0.973863000	-0.120718000	
	F	-3.899384000	-1.360606000	1.992244000	
	F	-1.785758000	-0.729420000	0.102854000	
	F	2.575234000	0.872803000	0.016228000	
	F	3.233339000	-2.557693000	-0.238247000	
	F	-3.926006000	-2.531443000	-0.620091000	
	F	-2.133437000	2.281277000	0.359154000	
	F	1.394956000	-1.165402000	0.404479000	
	F	-0.104464000	1.037238000	-0.684109000	
	F	3.433312000	-0.864473000	1.424537000	
	F	-2.186599000	0.953118000	-2.359449000	
	F	1.354503000	3.322059000	0.108876000	
	F	4.408886000	-0.508218000	-0.624295000	
	F	0.617340000	1.309707000	2.059728000	
	F	-3.849844000	0.341223000	-0.341506000	
	F	2.376968000	-0.836525000	-1.645468000	

$Al_3AsF_{15}^-$

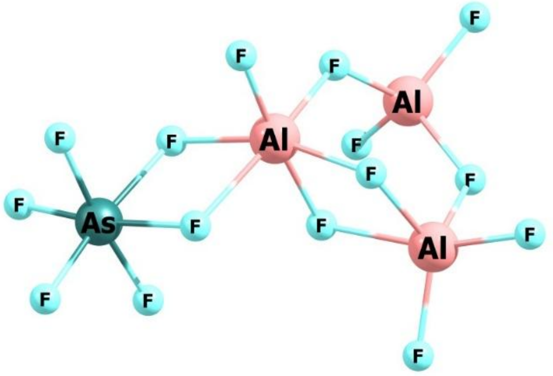

0.0

Al	2.679260000	-1.595664000	-0.334666000
Al	2.219907000	1.659778000	-0.221106000
Al	0.112726000	-0.029973000	0.975748000
As	-2.647417000	-0.055512000	-0.222768000
F	1.260883000	1.381773000	1.338070000
F	-1.205777000	-1.181228000	0.101809000
F	-0.372859000	-0.367877000	2.565245000
F	3.521158000	2.458947000	0.543854000
F	3.952279000	-2.449661000	0.366478000
F	-3.821265000	1.196372000	-0.404494000
F	1.755103000	2.638951000	-1.523878000
F	0.793501000	0.365349000	-0.682331000
F	-1.436627000	1.112319000	0.552694000
F	3.231507000	0.095431000	-0.523351000
F	-3.205325000	-0.426733000	1.369946000
F	1.423439000	-1.406424000	0.921437000
F	2.062809000	-2.226991000	-1.764603000
F	-1.926371000	0.313481000	-1.747085000
F	-3.564661000	-1.349484000	-0.903608000

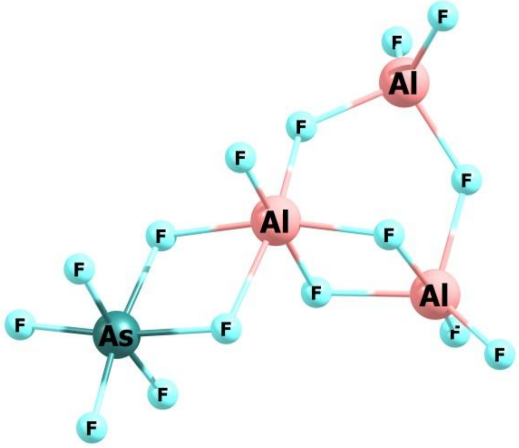

1.4

Al	-1.946764000	1.792608000	-0.103657000
Al	-3.210391000	-1.257825000	0.344074000
Al	-0.111918000	-0.480591000	-0.563034000
As	2.777433000	-0.062236000	0.182356000
F	-1.359223000	0.462085000	-1.464205000
F	1.519445000	0.538374000	-1.034207000
F	-0.477776000	0.944695000	0.613319000
F	4.011064000	0.914866000	-0.526428000
F	-3.310346000	0.512145000	0.132716000
F	-1.438852000	-1.439459000	0.403336000

	Atom	X	Y	Z	
$Al_3AsF_{15}^-$	F	1.264905000	-0.992733000	0.732027000	
	F	3.110711000	-1.368891000	-0.895480000	
	F	2.282476000	1.222484000	1.227246000	
	F	-3.831889000	-1.678320000	1.850069000	
	F	-2.348426000	2.637513000	1.326002000	
	F	-3.874699000	-2.023466000	-0.995942000	
	F	0.221843000	-1.736621000	-1.654521000	
	F	3.725390000	-0.781624000	1.433400000	
	F	-2.067660000	2.938870000	-1.349969000	
	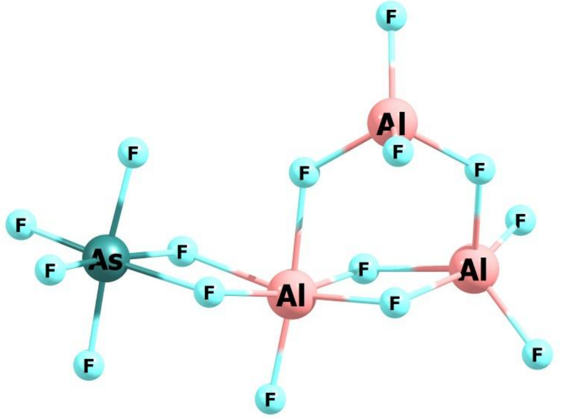				**3.4**
	Al	-0.160767000	-0.907572000	0.136785000	
	Al	-3.074256000	-1.011565000	-0.141965000	
	Al	-1.815789000	1.990079000	0.133264000	
	As	2.698567000	0.013012000	-0.076139000	
	F	0.304794000	-2.475375000	0.596302000	
	F	-3.827806000	-1.950875000	1.049619000	
	F	-3.192665000	0.855021000	-0.035182000	
	F	-1.620444000	2.476915000	1.730651000	
	F	1.329975000	-0.690177000	-1.121848000	
	F	-1.500999000	-1.359068000	-1.057293000	
	F	-4.063341000	-1.115547000	-1.530477000	
	F	-0.505394000	0.929732000	-0.419013000	
	F	3.097206000	-1.600744000	0.382400000	
	F	3.796587000	-0.034235000	-1.407193000	
	F	-1.594379000	-0.612692000	1.190859000	
	F	-2.036945000	3.212480000	-1.004517000	
	F	1.299738000	-0.048385000	1.141902000	
	F	2.147326000	1.588580000	-0.528431000	
	F	3.767218000	0.674187000	1.106385000	
	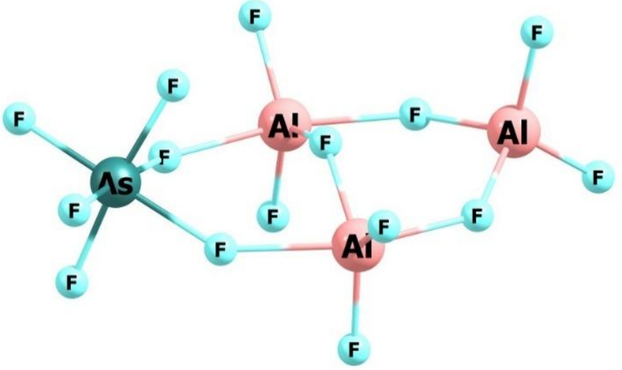				**4.3**
	Al	-0.660748000	-1.670684000	0.278101000	
	Al	-0.660968000	1.671080000	0.277526000	

Al	-3.568765000	-0.000481000	-0.302485000
As	2.536050000	-0.000149000	-0.139453000
F	-0.657564000	-0.000025000	-0.451507000
F	-0.691680000	1.849163000	1.953215000
F	-0.694272000	-1.842834000	1.954381000
F	1.310811000	-1.280195000	0.358391000
F	3.629544000	-1.269504000	-0.570066000
F	-2.570492000	1.401903000	0.157317000
F	-2.570492000	-1.404141000	0.153264000
F	1.310961000	1.280418000	0.357006000
F	-0.528589000	2.926195000	-0.835188000
F	-3.767458000	0.001816000	-1.975149000
F	-4.962550000	-0.001697000	0.643209000
F	-0.526296000	-2.928637000	-0.831160000
F	1.794560000	-0.001047000	-1.704902000
F	3.059014000	0.000655000	1.508337000
F	3.629678000	1.268599000	-0.571469000

$Al_3AsF_{15}^-$

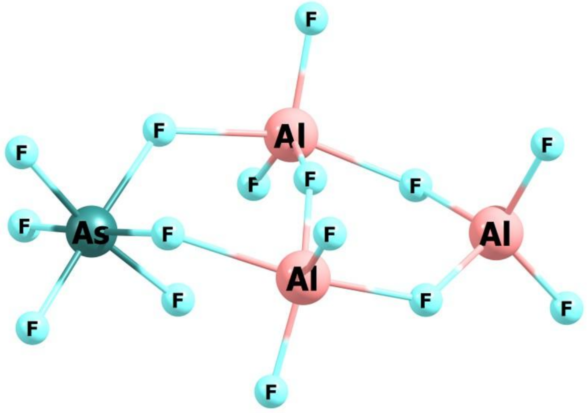

5.8

Al	0.559966000	1.760556000	0.252576000
Al	0.559321000	-1.760326000	0.252684000
Al	3.404980000	-0.000214000	-0.290828000
As	-2.368458000	0.000074000	-0.112650000
F	2.327724000	-1.415366000	-0.430904000
F	-1.126807000	-0.000237000	-1.337695000
F	0.076641000	-2.554408000	-1.155662000
F	4.119123000	-0.001012000	1.233181000
F	0.884703000	-2.655262000	1.643361000
F	2.328767000	1.415966000	-0.429594000
F	0.743917000	0.000204000	0.730043000
F	-1.346332000	1.278402000	0.729738000
F	-3.264131000	1.260086000	-0.879562000
F	0.077320000	2.553344000	-1.156475000
F	-3.370407000	0.000174000	1.294997000
F	0.884083000	2.656247000	1.643033000
F	4.425006000	0.000069000	-1.632080000
F	-3.264127000	-1.260256000	-0.879070000
F	-1.346185000	-1.278243000	0.730004000

$Al_3AsF_{15}^-$

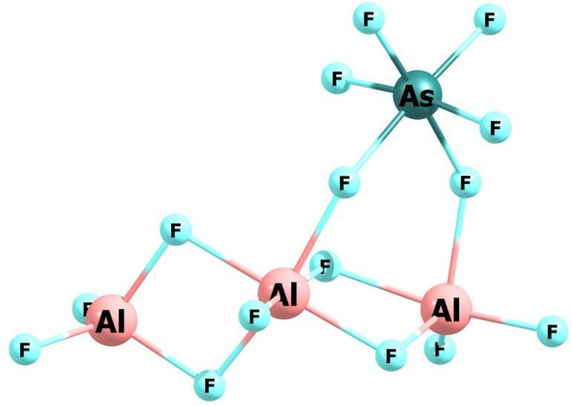

6.0

Al	-3.599638000	-0.594507000	-0.418385000
Al	0.571304000	2.225031000	-0.122210000
Al	-1.146813000	0.077784000	0.819669000
As	2.149071000	-0.931805000	-0.164785000
F	0.005951000	3.260664000	-1.341780000
F	-4.808253000	-1.389355000	0.438947000
F	1.740577000	3.119431000	0.739509000
F	-2.906590000	0.794808000	0.468436000
F	1.903430000	0.868321000	-0.534024000
F	2.293810000	-2.602617000	0.248126000
F	2.942089000	-0.420348000	1.281369000
F	-1.981729000	-1.323527000	-0.202882000
F	-0.351725000	1.660054000	1.377583000
F	-3.956892000	-0.243994000	-2.022747000
F	-0.569487000	0.804268000	-0.754904000
F	3.607264000	-0.870310000	-1.088600000
F	0.583518000	-0.858326000	0.808210000
F	1.186410000	-1.274558000	-1.556069000
F	-1.537533000	-0.575447000	2.339929000

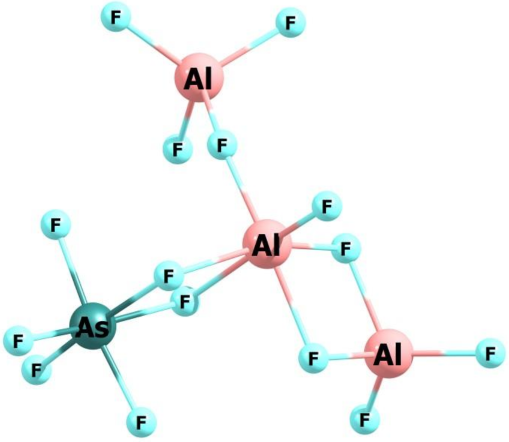

6.2

Al	0.175596000	-3.159398000	0.310845000
Al	-0.757933000	-0.033864000	-0.927865000
Al	-2.937390000	1.162929000	0.443270000
As	1.830664000	1.167807000	0.086480000
F	-1.221242000	-4.078235000	0.017871000
F	2.469867000	1.405124000	1.665488000
F	1.604235000	-3.877653000	-0.250964000
F	0.881212000	0.799754000	-1.484781000
F	-0.040920000	-1.687112000	-0.765900000

	F	-2.825337000	1.733453000	2.017524000	
	F	0.253379000	-2.498252000	1.869987000	
	F	-1.578438000	1.687470000	-0.588286000	
	F	-2.193506000	-0.459926000	0.229790000	
	F	2.465115000	-0.432891000	-0.023285000	
	F	3.146863000	1.816115000	-0.820243000	
	F	0.270544000	0.427102000	0.729594000	
	F	-1.453395000	-0.187381000	-2.465885000	
	F	-4.435219000	1.301702000	-0.302972000	
	F	1.028456000	2.701475000	0.105939000	
**$Al_3AsF_{15}^-$**	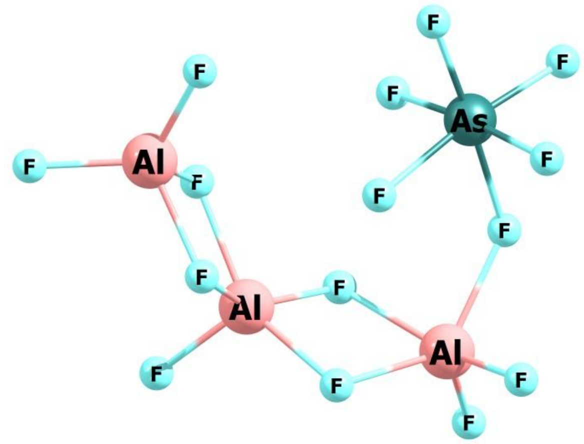				**7.5**
	Al	-2.950813000	-1.148694000	0.209158000	
	Al	-1.579743000	1.294854000	-0.225690000	
	Al	1.168778000	2.079435000	0.215957000	
	As	1.673064000	-1.197333000	-0.117901000	
	F	0.214234000	-0.469457000	0.623496000	
	F	1.940212000	2.058182000	1.731178000	
	F	-2.284678000	-2.588081000	0.740359000	
	F	-2.433060000	0.300412000	1.142967000	
	F	-0.554805000	2.144710000	1.001560000	
	F	1.598911000	3.470675000	-0.651516000	
	F	-2.802572000	2.336810000	-0.742008000	
	F	-0.081542000	1.470956000	-1.171607000	
	F	2.163008000	0.594844000	-0.439535000	
	F	2.470333000	-1.054558000	1.411554000	
	F	1.143868000	-2.809858000	0.209975000	
	F	-2.063723000	-0.313648000	-1.107582000	
	F	-4.608954000	-1.141760000	-0.065185000	
	F	0.862815000	-1.162094000	-1.652870000	
	F	3.157286000	-1.661664000	-0.886540000	

$Al_3AsF_{15}^-$

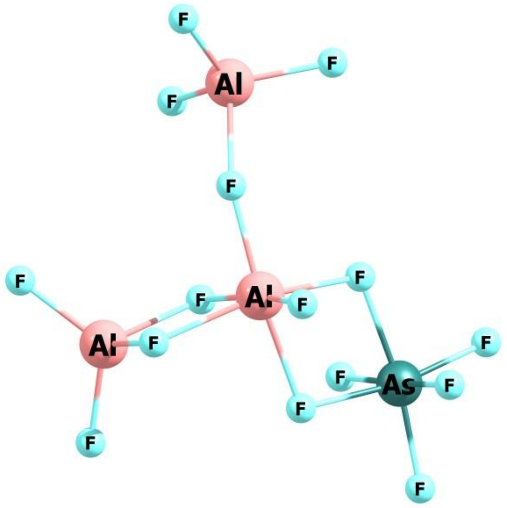

7.7

Al	-1.257421000	2.583498000	-0.212161000
Al	-2.973583000	-1.723887000	-0.317804000
Al	-0.286296000	0.153737000	0.949851000
As	2.420804000	-0.448632000	-0.222594000
F	1.895631000	0.260291000	-1.708775000
F	-0.188384000	3.830742000	-0.564309000
F	2.979956000	-1.863623000	-1.030626000
F	-4.355350000	-1.938199000	0.640929000
F	3.892506000	0.450146000	-0.242893000
F	0.064830000	-0.352626000	2.526206000
F	-1.877410000	-0.692262000	0.737753000
F	-3.207387000	-0.768772000	-1.698852000
F	-0.946592000	1.871998000	1.412714000
F	-0.663264000	0.995499000	-0.758312000
F	-2.887945000	2.883869000	-0.457238000
F	1.527152000	0.915935000	0.654310000
F	2.774982000	-1.119362000	1.327653000
F	0.705178000	-1.161296000	-0.049338000
F	-2.065194000	-3.131080000	-0.579546000

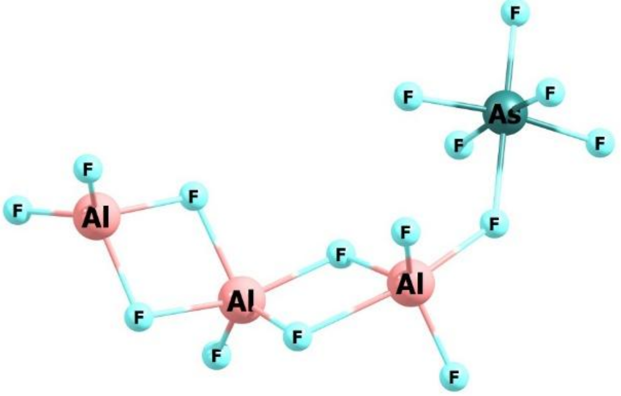

8.2

Al	0.370193000	1.635290000	0.525828000
Al	-2.161651000	0.760504000	-0.656132000
Al	-4.060640000	-1.136980000	0.310549000
As	2.901965000	-0.665135000	-0.101590000
F	-4.153736000	-1.602913000	1.916346000
F	-2.360274000	-1.060859000	-0.304616000
F	2.074667000	1.047462000	0.035438000

	F 3.657362000 -2.222414000 -0.235143000<br>F -5.086451000 -1.878746000 -0.789228000<br>F -1.695251000 1.884545000 0.601207000<br>F 1.405955000 -1.314482000 0.519825000<br>F -0.349625000 0.542761000 -0.774906000<br>F 3.466372000 -0.414137000 1.523990000<br>F -2.642676000 1.183144000 -2.210412000<br>F 0.572454000 3.238583000 0.025645000<br>F 4.288521000 0.194423000 -0.710148000<br>F 0.344037000 1.126476000 2.134003000<br>F -3.955048000 0.629349000 0.094473000<br>F 2.246186000 -0.732651000 -1.714333000	
$B_2AlPF_{15}^-$	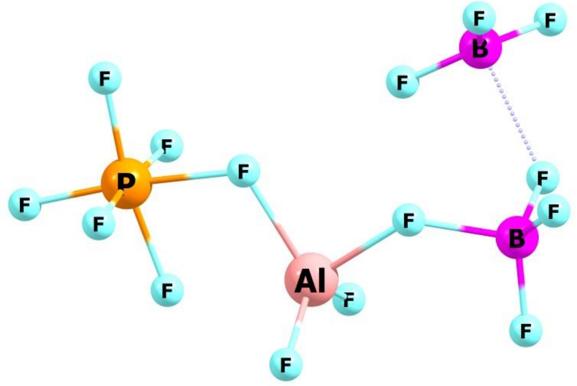	**0.0**
	B 2.814582000 -1.125675000 -0.239204000<br>B 2.502156000 1.991978000 0.046758000<br>Al -0.223124000 -1.203367000 0.313312000<br>P -2.735283000 0.410806000 -0.127647000<br>F 2.776068000 -2.244621000 0.564116000<br>F 3.446946000 -1.273573000 -1.450921000<br>F 1.306435000 -0.825346000 -0.611615000<br>F -0.648004000 -2.715550000 -0.293884000<br>F -2.345428000 -0.897883000 0.865467000<br>F -3.097022000 -0.646069000 -1.309980000<br>F 3.212529000 0.041134000 0.461761000<br>F 1.302472000 1.829326000 0.631644000<br>F 3.497422000 2.586891000 0.736972000<br>F -2.848408000 1.646993000 -1.168527000<br>F -4.261432000 0.475751000 0.398730000<br>F -1.005790000 0.208087000 -0.616350000<br>F 2.602973000 1.893900000 -1.292946000<br>F 0.184038000 -0.912889000 1.916297000<br>F -2.195448000 1.406088000 1.036334000	
	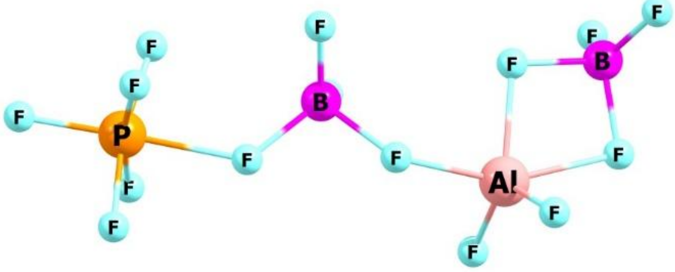	**1.0**
	B -4.100844000 0.891720000 -0.087552000<br>B 0.435154000 0.291050000 0.309807000<br>Al -2.325612000 -1.054812000 -0.041169000	

	P	3.513620000	0.071423000	-0.076654000	
	F	-0.573034000	-0.820353000	0.582168000	
	F	-1.999802000	-1.538917000	-1.620741000	
	F	-4.244320000	-0.511024000	-0.495806000	
	F	1.665587000	-0.549312000	0.158636000	
	F	-2.817770000	-2.117867000	1.173640000	
	F	0.102511000	0.886638000	-0.871708000	
	F	3.225705000	-0.149783000	-1.659183000	
	F	0.512428000	1.065801000	1.427816000	
	F	5.046479000	0.540769000	-0.269206000	
	F	3.520896000	0.204227000	1.542300000	
	F	3.849360000	-1.512242000	0.067736000	
	F	-2.606193000	0.746275000	0.322096000	
	F	-4.185335000	1.754624000	-1.140829000	
	F	2.872778000	1.559656000	-0.181898000	
	F	-4.829612000	1.188994000	1.028728000	
**$B_2AlPF_{15}^-$**	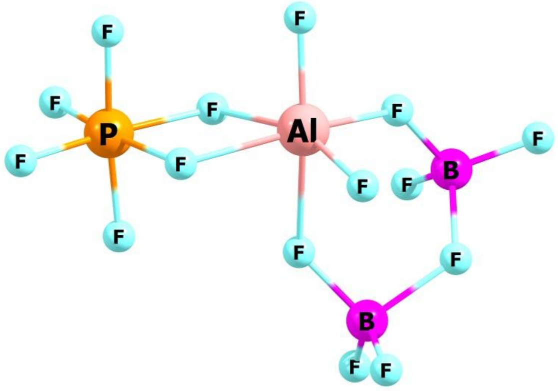				**1.3**
	B	-2.660536000	-0.786848000	0.898063000	
	B	-2.003937000	1.547826000	-0.397649000	
	Al	-0.003971000	-0.919986000	-0.798877000	
	P	2.495942000	0.253310000	0.306462000	
	F	1.044644000	-0.492994000	0.898247000	
	F	-3.717131000	-1.651261000	0.889445000	
	F	1.771354000	1.695157000	0.485573000	
	F	3.105544000	-1.227650000	0.062489000	
	F	0.640689000	-2.492565000	-0.819105000	
	F	-1.463844000	-1.455209000	0.347667000	
	F	-2.978024000	0.282911000	-0.159181000	
	F	-2.281334000	2.427226000	0.606063000	
	F	-0.712741000	0.931307000	-0.216698000	
	F	-0.841908000	-0.654234000	-2.253258000	
	F	3.746810000	0.938957000	-0.462896000	
	F	1.627083000	0.081028000	-1.215107000	
	F	-2.353660000	-0.164450000	2.072956000	
	F	-2.271277000	1.939934000	-1.668636000	
	F	3.121000000	0.325761000	1.797598000	

$B_2AlPF_{15}^-$

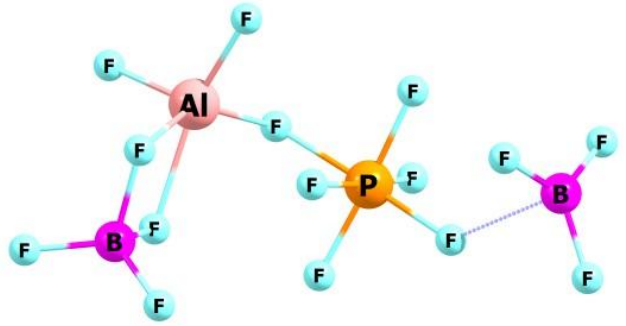

2.2

B	3.769942000	1.007784000	0.593734000
B	-2.930075000	1.401615000	-0.303511000
Al	-1.805730000	-0.734006000	0.737319000
P	0.881136000	-0.753570000	-0.681945000
F	2.885611000	1.401597000	1.516175000
F	2.149951000	0.263946000	-0.914214000
F	4.209211000	1.883986000	-0.327495000
F	4.374694000	-0.184175000	0.700033000
F	1.498785000	-1.275894000	0.723108000
F	0.179197000	-0.245061000	-2.055157000
F	1.552466000	-2.004120000	-1.468388000
F	0.015778000	0.423588000	0.165755000
F	-2.229374000	2.533767000	-0.622581000
F	-2.343542000	0.196217000	-0.943674000
F	-3.114143000	-1.812995000	0.613513000
F	-0.554187000	-1.723070000	-0.364498000
F	-1.061169000	-0.907288000	2.252435000
F	-2.601811000	0.972917000	1.113956000
F	-4.288344000	1.454210000	-0.478645000

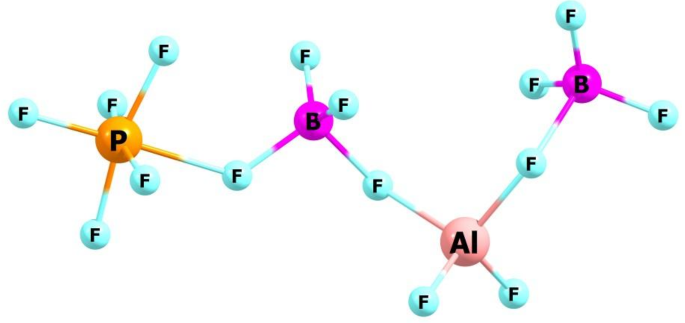

3.8

Al	2.095406000	1.410473000	0.017288000
B	3.889352000	-1.110308000	0.096466000
B	-0.298814000	-0.455861000	-0.252999000
P	-3.383980000	-0.085369000	0.060758000
F	3.244551000	-1.134045000	-1.121119000
F	-1.480920000	0.424276000	-0.356937000
F	-0.376743000	-1.508374000	-1.102893000
F	5.176237000	-0.636435000	0.091489000
F	2.980120000	2.166234000	-1.198508000
F	-4.917463000	-0.458200000	0.382420000
F	3.622009000	-2.140016000	0.954958000
F	0.064350000	-0.665049000	1.044118000
F	-2.742508000	-1.473411000	0.603178000
F	3.046440000	0.188481000	0.851947000
F	-3.379769000	-0.692994000	-1.443307000

$B_2AlPF_{15}^-$	F 0.776902000 0.516609000 -0.848408000<br>F 1.363574000 2.403828000 1.161678000<br>F -3.074415000 0.598421000 1.497151000<br>F -3.683837000 1.385701000 -0.555040000	
	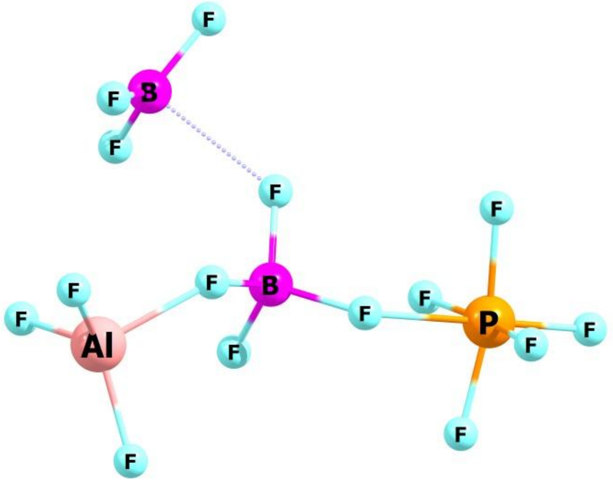	**8.8**
	B -0.129660000 -0.034441000 0.179311000<br>B -1.980692000 2.711154000 -0.045142000<br>Al -2.704129000 -1.718393000 -0.062039000<br>P 2.986161000 -0.192090000 -0.056428000<br>F -2.081061000 2.627421000 1.287574000<br>F -0.268209000 -0.340198000 1.496502000<br>F -3.503173000 -1.925529000 -1.542237000<br>F -2.035262000 -3.137968000 0.572299000<br>F -0.169850000 1.306172000 -0.168066000<br>F 4. 537343000 0.159150000 0.202241000<br>F 3.247928000 -1.283410000 -1.228887000<br>F -3.501342000 -0.722933000 1.047810000<br>F 2.908190000 -1.397827000 1.024554000<br>F 2.750099000 0.943228000 -1.191531000<br>F -2.734401000 1.928487000 -0.829917000<br>F -1.218640000 -0.725413000 -0.609755000<br>F -1.285987000 3.723578000 -0.594036000<br>F 1.069710000 -0.662692000 -0.414975000<br>F 2.386101000 0.823142000 1.057545000	
	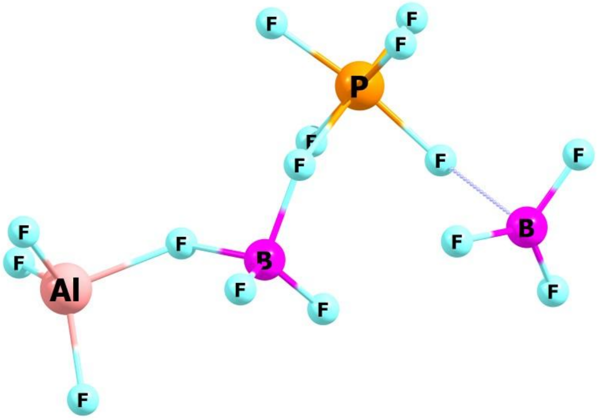	**9.0**
	B -0.747960000 -0.830548000 -0.050014000<br>B 3.146237000 -1.765941000 -0.034992000<br>Al -3.727752000 -0.149896000 0.020224000<br>P 1.377846000 1.468632000 0.025106000	

	F	-4.016322000	-1.725328000	0.568520000	
	F	-4.367066000	1.068908000	1.008210000	
	F	-3.950967000	0.103704000	-1.637875000	
	F	-1.896066000	0.083382000	0.296337000	
	F	2.384099000	2.581846000	0.619469000	
	F	3.148616000	-2.265573000	1.209551000	
	F	2.153950000	0.214403000	0.757148000	
	F	4.241404000	-1.136412000	-0.489987000	
	F	0.222812000	0.137866000	-0.712149000	
	F	-0.213474000	-1.334986000	1.091457000	
	F	0.346489000	1.519919000	1.273690000	
	F	-1.149826000	-1.693174000	-1.026008000	
	F	2.145844000	-2.053686000	-0.875087000	
	F	0.456029000	2.523214000	-0.791749000	
	F	2.250220000	1.187208000	-1.315359000	
**$B_2AlAsF_{15}^-$**	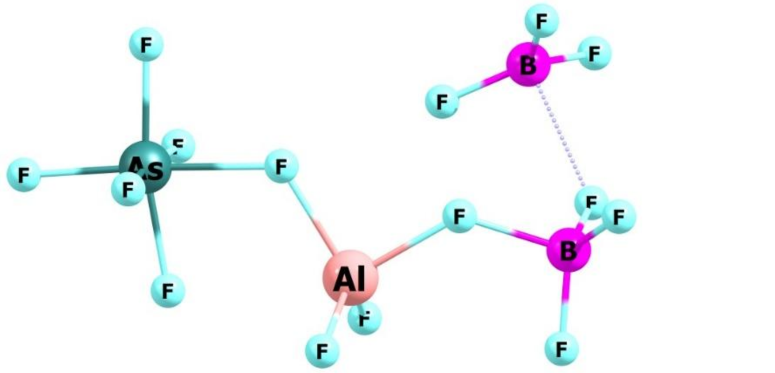				**0.0**
	B	3.235946000	-1.091739000	-0.236244000	
	B	2.764616000	2.083684000	0.039344000	
	Al	0.228579000	-1.245011000	0.298011000	
	As	-2.611441000	0.287914000	-0.092010000	
	F	3.154966000	-2.197916000	0.582553000	
	F	3.865248000	-1.271911000	-1.442088000	
	F	1.707004000	-0.793026000	-0.634048000	
	F	-0.221762000	-2.765322000	-0.257111000	
	F	-2.273495000	-1.060506000	0.983754000	
	F	-2.927745000	-0.842077000	-1.372119000	
	F	3.616295000	0.082462000	0.439959000	
	F	1.586852000	1.826472000	0.627809000	
	F	3.736514000	2.699548000	0.738406000	
	F	-2.624475000	1.587297000	-1.240256000	
	F	-4.276576000	0.420131000	0.351364000	
	F	-0.743279000	0.061072000	-0.525684000	
	F	2.877570000	1.984196000	-1.296405000	
	F	0.510892000	-0.920862000	1.917430000	
	F	-2.076540000	1.382026000	1.142736000	

$B_2AlAsF_{15}^-$

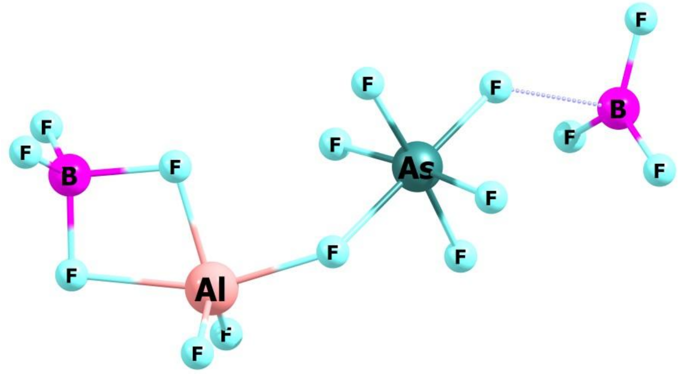

0.1

B	4.450975000	0.486091000	0.532385000
B	-3.871066000	1.129528000	-0.030377000
Al	-2.288710000	-0.924668000	0.445878000
As	1.000155000	-0.175824000	-0.444668000
F	3.964461000	0.794699000	1.743343000
F	2.495563000	0.746460000	-0.545032000
F	5.011644000	1.452840000	-0.219030000
F	4.617084000	-0.798307000	0.182152000
F	1.557796000	-1.081838000	0.925949000
F	0.321819000	0.654598000	-1.812475000
F	1.588393000	-1.421735000	-1.502541000
F	0.251152000	0.978397000	0.618187000
F	-3.763309000	2.302874000	0.660189000
F	-2.451998000	0.676959000	-0.472973000
F	-3.030523000	-2.240857000	-0.308674000
F	-0.607740000	-1.163058000	-0.354695000
F	-1.850399000	-0.920009000	2.071177000
F	-4.097132000	-0.021611000	0.847372000
F	-4.690304000	1.123342000	-1.125439000

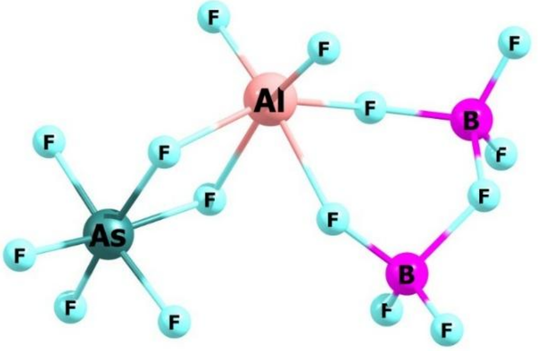

0.7

B	3.262952000	-0.440760000	-0.593670000
B	1.773497000	1.678435000	0.357989000
Al	0.371007000	-1.198696000	0.477623000
As	-2.289787000	0.165668000	-0.156521000
F	-0.669792000	-0.128520000	-0.945904000
F	4.048705000	-1.328924000	0.075952000
F	-1.677793000	1.676928000	0.431578000
F	-2.796384000	-1.397701000	-0.693387000
F	-0.105744000	-2.639179000	-0.287516000
F	1.871528000	-0.929611000	-0.710924000
F	3.114781000	0.789274000	0.326198000
F	1.495191000	1.978813000	-0.939737000
F	0.842771000	0.748503000	0.939114000

$B_2AlAsF_{15}^-$	F	1.152908000	-1.584505000	1.936903000	
	F	-3.696450000	0.318047000	0.840706000	
	F	-1.363268000	-0.671547000	1.218540000	
	F	3.692944000	-0.009223000	-1.814516000	
	F	2.083415000	2.693108000	1.210673000	
	F	-2.930850000	0.920942000	-1.572736000	
	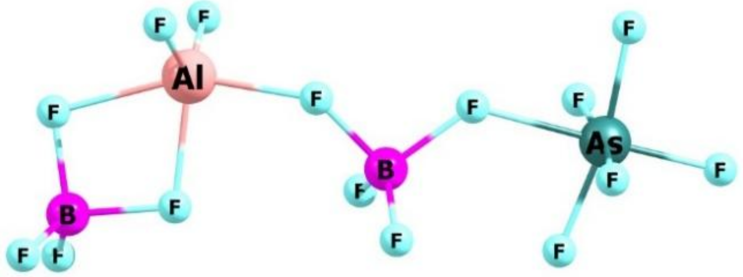				**0.8**
	B	-4.448224000	-0.902670000	0.079918000	
	B	0.065252000	-0.286137000	-0.284857000	
	Al	-2.698711000	1.061785000	0.039227000	
	As	3.206150000	-0.055307000	0.052876000	
	F	-0.925215000	0.829866000	-0.550389000	
	F	-2.392793000	1.562319000	1.617057000	
	F	-4.610754000	0.507936000	0.461662000	
	F	1.318489000	0.556940000	-0.157959000	
	F	-3.164618000	2.107541000	-1.199457000	
	F	-0.237034000	-0.864915000	0.909872000	
	F	2.938599000	0.181859000	1.750063000	
	F	0.137874000	-1.068312000	-1.394885000	
	F	4.853169000	-0.545098000	0.234147000	
	F	3.202127000	-0.206225000	-1.675614000	
	F	3.559100000	1.636295000	-0.111833000	
	F	-2.947164000	-0.747436000	-0.305489000	
	F	-4.542658000	-1.746057000	1.146920000	
	F	2.542005000	-1.651626000	0.182705000	
	F	-5.153890000	-1.223537000	-1.043483000	
	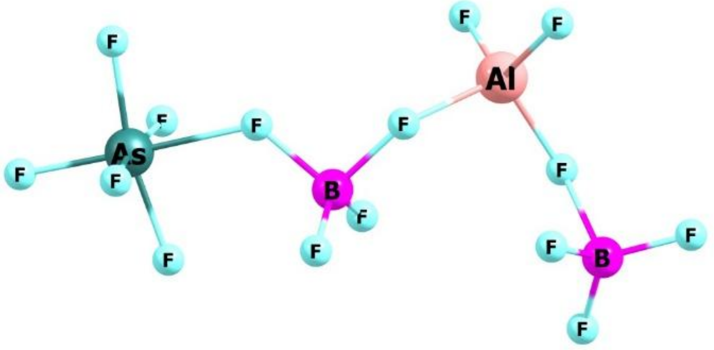				**3.9**
	Al	2.449097000	-1.436371000	-0.022365000	
	B	4.161184000	1.150188000	-0.100468000	
	B	0.068056000	0.444926000	0.269339000	
	As	-3.055949000	0.066010000	-0.048294000	
	F	3.488308000	1.160526000	1.101318000	
	F	-1.140734000	-0.441340000	0.340327000	
	F	-0.027869000	1.467357000	1.148095000	
	F	5.463028000	0.725339000	-0.064341000	
	F	3.331518000	-2.174502000	1.204633000	

$B_2AlAsF_{15}^-$	F	-4.703104000	0.466626000	-0.369488000	
	F	3.865881000	2.149708000	-0.982534000	
	F	0.421543000	0.681558000	-1.021894000	
	F	-2.395718000	1.577035000	-0.582977000	
	F	3.381831000	-0.203610000	-0.854826000	
	F	-3.068258000	0.664158000	1.579105000	
	F	1.110039000	-0.555704000	0.840684000	
	F	1.720409000	-2.437157000	-1.161063000	
	F	-2.750656000	-0.605617000	-1.615879000	
	F	-3.378232000	-1.527829000	0.554409000	
	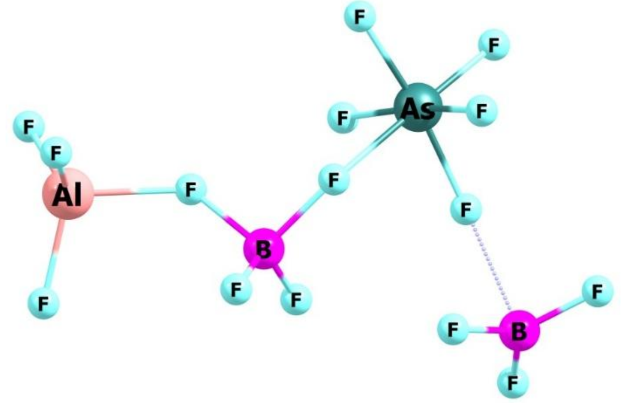				**8.4**
	B	0.953019000	0.930077000	-0.067914000	
	B	-2.892550000	2.104319000	-0.029525000	
	Al	3.897133000	0.123351000	0.026027000	
	As	-1.338026000	-1.300066000	0.020453000	
	F	4.228772000	1.691081000	0.570357000	
	F	4.475211000	-1.113603000	1.027292000	
	F	4.123199000	-0.145703000	-1.628574000	
	F	2.049490000	-0.035912000	0.280930000	
	F	-2.485608000	-2.421398000	0.656876000	
	F	-2.848037000	2.639426000	1.199828000	
	F	-2.098537000	0.081488000	0.791524000	
	F	-4.043774000	1.571937000	-0.471132000	
	F	-0.065757000	-0.008399000	-0.733254000	
	F	0.425971000	1.454054000	1.066069000	
	F	-0.252661000	-1.411390000	1.362809000	
	F	1.385980000	1.767020000	-1.049727000	
	F	-1.872161000	2.280243000	-0.878184000	
	F	-0.421782000	-2.492328000	-0.838626000	
	F	-2.245885000	-0.953555000	-1.414641000	

$B_2AlAsF_{15}^-$	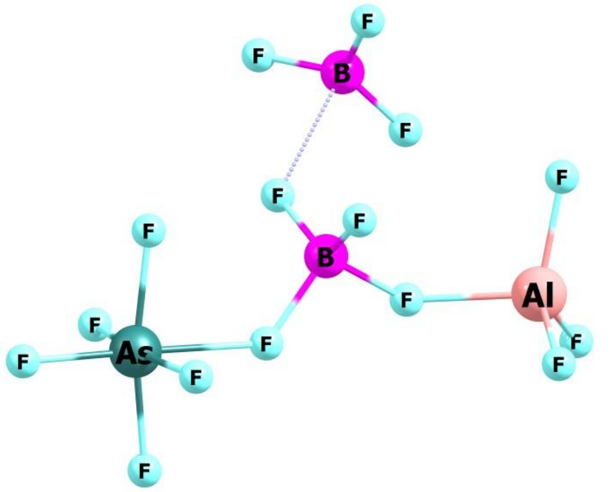	8.9

B	-0.426630000	-0.036402000	0.217365000
B	-2.366868000	2.670849000	-0.053946000
Al	-2.986948000	-1.731809000	-0.073115000
As	2.729557000	-0.126723000	-0.049930000
F	-2.499114000	2.588803000	1.275449000
F	-0.542186000	-0.377107000	1.524783000
F	-3.774273000	-1.896541000	-1.563284000
F	-2.338821000	-3.168981000	0.540125000
F	-0.485827000	1.302923000	-0.109983000
F	4.385585000	0.280779000	0.214531000
F	3.034899000	-1.265065000	-1.322352000
F	-3.762768000	-0.735253000	1.049554000
F	2.707052000	-1.431487000	1.090278000
F	2.457515000	1.104805000	-1.240111000
F	-3.062806000	1.852152000	-0.854519000
F	-1.475005000	-0.749224000	-0.590868000
F	-1.686311000	3.698388000	-0.589939000
F	0.808694000	-0.631449000	-0.392555000
F	2.091413000	0.929830000	1.166790000

$BAl_2PF_{15}^-$	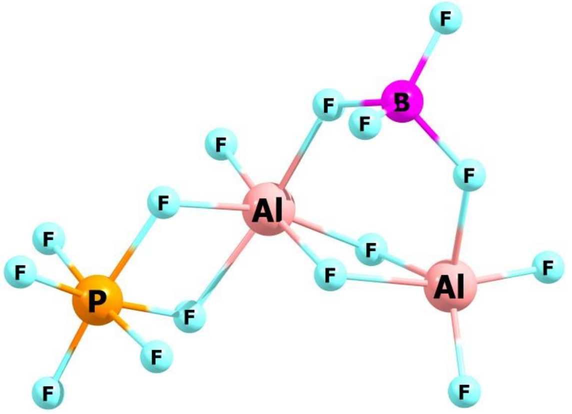	0.0

Al	2.207804000	-1.385083000	-0.106681000
B	2.318313000	1.701438000	-0.470772000
Al	-0.050859000	0.170061000	0.834829000
P	-2.762778000	-0.065852000	-0.249419000
F	1.235720000	1.571187000	0.567248000
F	-1.593358000	-1.030933000	0.641483000
F	-0.514553000	0.777482000	2.348737000
F	3.251060000	2.579928000	0.009085000

Species	Atom	X	Y	Z	
$BAl_2PF_{15}^-$	F	3.611592000	-1.843978000	0.752681000	
	F	-3.676329000	0.985096000	-1.069380000	
	F	1.765170000	1.981601000	-1.687685000	
	F	0.611081000	-0.483086000	-0.732480000	
	F	-1.437115000	1.091173000	-0.188875000	
	F	2.950148000	0.332024000	-0.499488000	
	F	-3.258224000	0.528103000	1.173124000	
	F	1.186147000	-1.082894000	1.414667000	
	F	2.018343000	-2.573161000	-1.304768000	
	F	-2.106718000	-0.645638000	-1.610655000	
	F	-3.841872000	-1.267363000	-0.188227000	
	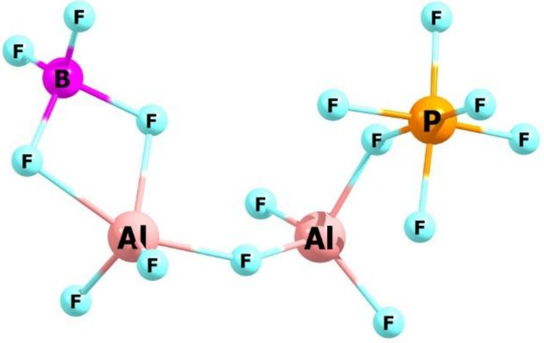				**3.8**
	Al	-2.068256000	0.530262000	0.921805000	
	Al	0.794695000	1.847375000	-0.539437000	
	B	-3.071275000	-1.280247000	-0.702047000	
	P	2.506801000	-0.913785000	0.099198000	
	F	1.686919000	0.370404000	-1.032299000	
	F	-3.651756000	-0.669446000	0.505681000	
	F	3.902767000	-0.173100000	-0.293550000	
	F	-3.722224000	-0.900303000	-1.839267000	
	F	-2.808306000	-2.607383000	-0.537034000	
	F	2.266555000	0.202242000	1.273888000	
	F	0.995375000	-1.464751000	0.342033000	
	F	2.619575000	-1.821765000	-1.243626000	
	F	-2.952661000	1.949977000	0.691817000	
	F	1.808672000	3.003910000	0.136719000	
	F	-0.070968000	2.292140000	-1.913643000	
	F	3.203909000	-1.994458000	1.074493000	
	F	-0.362730000	1.259484000	0.672052000	
	F	-1.736022000	-0.486145000	-0.600927000	
	F	-1.811253000	-0.160947000	2.436052000	
	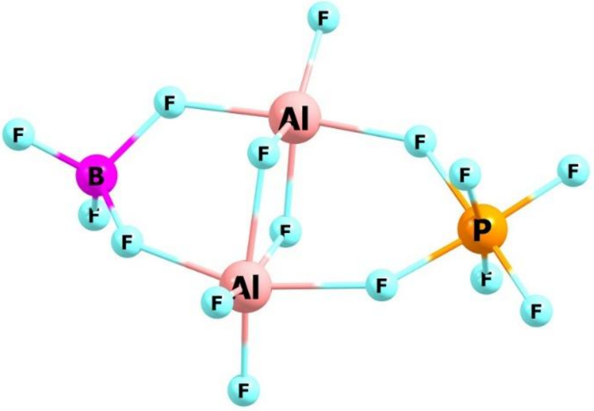				**5.5**

$BAl_2PF_{15}^-$

Al	0.552954000	1.413273000	0.122281000
Al	0.636934000	-1.447745000	0.094527000
B	3.361175000	0.184963000	-0.133226000
P	-2.617725000	0.062783000	-0.064401000
F	-1.378205000	1.330124000	0.024335000
F	0.617848000	0.152563000	-1.104924000
F	0.586909000	-2.462129000	1.460586000
F	0.674626000	-2.448752000	-1.280986000
F	3.649857000	0.219383000	-1.471021000
F	-2.392308000	0.084301000	-1.669584000
F	-2.627384000	0.092130000	1.557030000
F	4.421015000	0.252771000	0.733244000
F	-3.719603000	1.251355000	-0.147073000
F	0.514152000	0.142079000	1.334084000
F	2.458920000	1.376405000	0.179248000
F	0.501944000	3.094428000	0.167680000
F	-3.710823000	-1.125894000	-0.140527000
F	-1.365517000	-1.111237000	0.031815000
F	2.545399000	-1.005131000	0.194276000

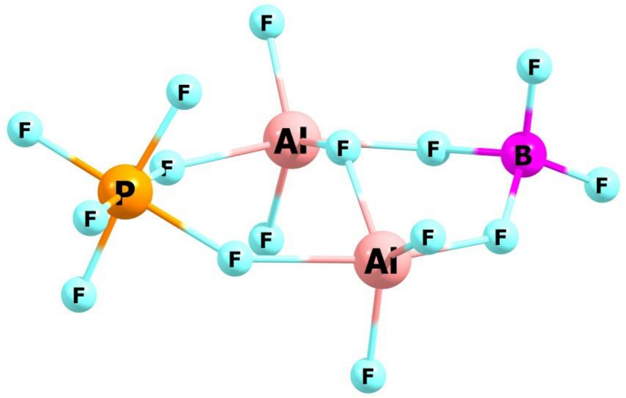

6.9

Al	-0.603527000	-1.613440000	0.211055000
Al	-0.605075000	1.613479000	0.211607000
B	-3.261307000	0.000039000	-0.302780000
P	2.560406000	0.000324000	-0.137989000
F	1.945140000	0.002843000	-1.641168000
F	-0.590027000	1.820148000	1.883197000
F	1.381115000	1.217759000	0.277379000
F	-0.567167000	2.888051000	-0.882914000
F	-4.510989000	0.001382000	0.257659000
F	-0.571908000	-2.884837000	-0.887402000
F	-0.585879000	-1.827190000	1.881745000
F	1.381202000	-1.219637000	0.273011000
F	2.981971000	-0.002606000	1.426814000
F	-0.558414000	0.000388000	-0.601321000
F	3.605825000	-1.186538000	-0.495730000
F	3.605675000	1.188533000	-0.491456000
F	-2.518370000	1.196729000	0.226329000
F	-3.190994000	-0.004073000	-1.672629000
F	-2.516926000	-1.191569000	0.234164000

$BAl_2PF_{15}^-$

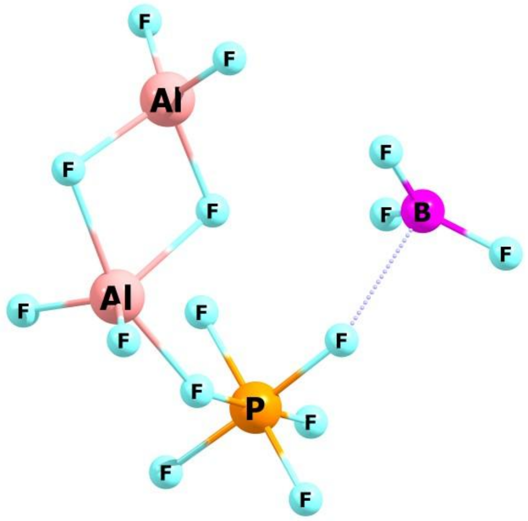

9.2

Al	-2.881902000	0.847601000	0.171537000
Al	-1.334355000	-1.555595000	-0.198811000
B	1.502189000	2.723140000	-0.121911000
P	2.001756000	-0.890507000	0.077187000
F	1.625607000	0.512088000	-0.734447000
F	2.228143000	-2.329084000	0.802508000
F	1.070181000	-0.386558000	1.321005000
F	-3.018026000	1.615071000	1.660943000
F	0.506668000	-1.573398000	-0.645634000
F	3.352815000	-0.241825000	0.701587000
F	0.170823000	2.785583000	-0.289915000
F	-3.203373000	-0.886422000	0.239148000
F	2.014548000	2.554320000	1.102930000
F	-1.834340000	-2.192746000	-1.683682000
F	-1.253310000	-2.431293000	1.239645000
F	2.306798000	3.070793000	-1.140685000
F	-3.600128000	1.622001000	-1.137695000
F	-1.205551000	0.300523000	-0.193980000
F	2.758485000	-1.425072000	-1.263249000

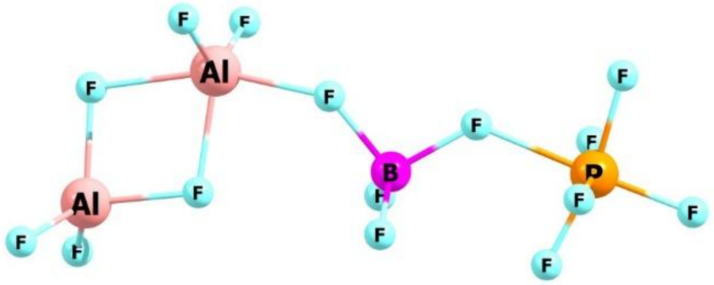

10.0

Al	4.070808000	-0.867786000	-0.021300000
Al	2.106589000	1.237654000	-0.072205000
B	-0.642213000	-0.182241000	0.210083000
P	-3.742293000	-0.159176000	-0.039144000
F	0.296174000	0.993327000	0.409574000
F	-3.018575000	-1.609507000	-0.139825000
F	-0.611912000	-0.948370000	1.337912000
F	2.462473000	2.266237000	1.219533000
F	4.056608000	0.851501000	-0.412976000
F	-4.172014000	1.403588000	0.087515000
F	1.849663000	1.715478000	-1.668603000

	F	-0.345915000	-0.777220000	-0.982258000	
	F	-1.936236000	0.574544000	0.120257000	
	F	2.310957000	-0.590184000	0.280850000	
	F	-3.689146000	-0.260008000	1.581644000	
	F	4.893762000	-1.240219000	1.395980000	
	F	4.349714000	-1.868660000	-1.340900000	
	F	-3.524566000	0.045767000	-1.635648000	
	F	-5.249955000	-0.723990000	-0.169466000	
**$BAl_2AsF_{15}^-$**	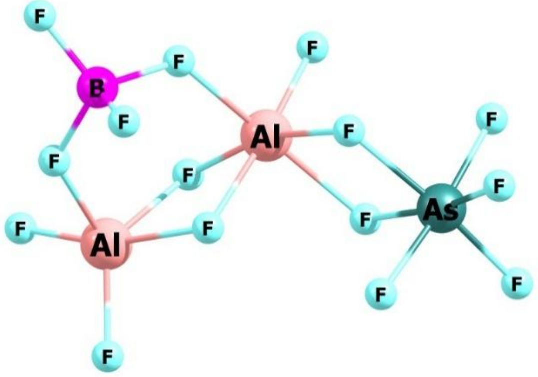				**0.0**
	B	-2.597733000	1.690276000	-0.498748000	
	Al	-2.473634000	-1.391369000	-0.107439000	
	Al	-0.245396000	0.188115000	0.865860000	
	As	2.556525000	-0.051993000	-0.196976000	
	F	-1.490420000	-1.061742000	1.433232000	
	F	1.134624000	1.138028000	-0.148867000	
	F	0.201355000	0.811986000	2.378140000	
	F	-3.892762000	-1.853059000	0.724774000	
	F	-3.549540000	2.563732000	-0.048496000	
	F	3.705076000	-1.337005000	-0.094994000	
	F	-2.246596000	-2.589827000	-1.288159000	
	F	-0.866189000	-0.484920000	-0.708504000	
	F	1.290872000	-1.037449000	0.730674000	
	F	-3.217475000	0.315179000	-0.535413000	
	F	3.066018000	0.598682000	1.320294000	
	F	-1.541878000	1.573344000	0.568010000	
	F	-2.015113000	1.967303000	-1.702266000	
	F	1.886202000	-0.688409000	-1.655659000	
	F	3.532577000	1.073788000	-1.068935000	
	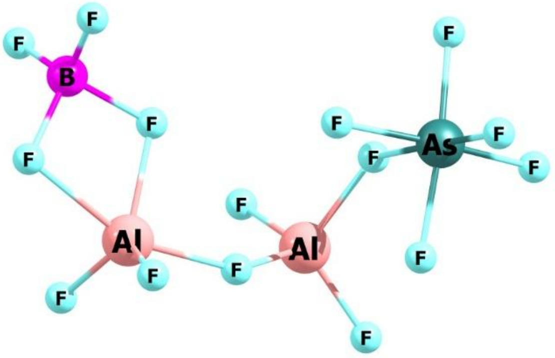				**4.1**
	Al	-2.331081000	0.454885000	0.937282000	
	Al	0.400232000	2.007481000	-0.537916000	
	B	-3.236164000	-1.380692000	-0.712567000	
	As	2.354880000	-0.707611000	0.070947000	
	F	1.417645000	0.612896000	-1.043970000	

<table>
<tr><td rowspan="3">$BAl_2AsF_{15}^-$</td><td>F -3.835271000 -0.834751000 0.518199000
F 3.795240000 0.170717000 -0.344177000
F -3.928511000 -1.017467000 -1.830427000
F -2.886887000 -2.691185000 -0.581563000
F 2.049242000 0.434175000 1.352679000
F 0.779194000 -1.387075000 0.329368000
F 2.506633000 -1.623866000 -1.393878000
F -3.302718000 1.821773000 0.740804000
F 1.331744000 3.229444000 0.139563000
F -0.509912000 2.386063000 -1.901567000
F 3.175327000 -1.851423000 1.068944000
F -0.677151000 1.300027000 0.679705000
F -1.953429000 -0.504435000 -0.611543000
F -2.008833000 -0.240022000 2.436732000</td><td></td></tr>
<tr><td>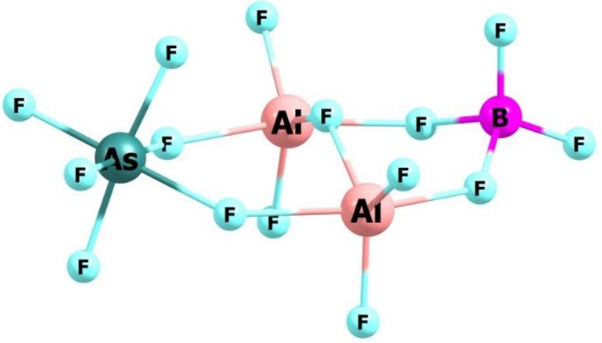
</td><td>5.1</td></tr>
<tr><td>Al -0.869715000 -1.626045000 0.237486000
Al -0.870962000 1.626114000 0.237955000
B -3.502668000 0.000038000 -0.340069000
As 2.365437000 0.000205000 -0.116889000
F 1.719311000 0.002950000 -1.723546000
F -0.878377000 1.824524000 1.910731000
F 1.121875000 1.291951000 0.304563000
F -0.844145000 2.898896000 -0.859607000
F -4.771798000 0.001367000 0.174184000
F -0.849129000 -2.895850000 -0.863680000
F -0.874117000 -1.830817000 1.909510000
F 1.121998000 -1.293881000 0.299791000
F 2.786880000 -0.003034000 1.559724000
F -0.782549000 0.000352000 -0.550361000
F 3.486549000 -1.266052000 -0.485042000
F 3.486607000 1.267721000 -0.480434000
F -2.778141000 1.194854000 0.216621000
F -3.381017000 -0.004142000 -1.706244000
F -2.776977000 -1.189713000 0.224562000</td><td></td></tr>
</table>

$BAl_2AsF_{15}^-$

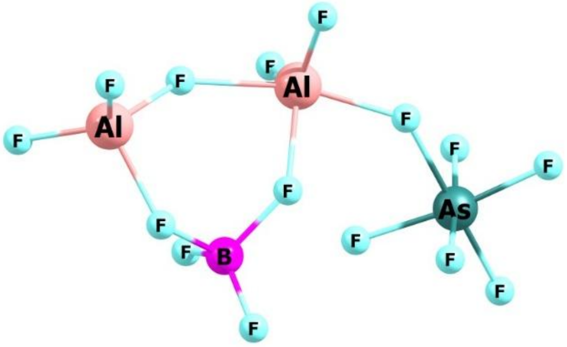

7.6

Al	3.780683000	0.098075000	0.023282000
Al	0.476958000	1.354473000	-0.052329000
B	1.347414000	-1.621374000	0.043699000
As	-2.686970000	-0.111367000	0.017632000
F	0.678175000	-0.416168000	0.604817000
F	-1.505786000	-0.857457000	-1.031789000
F	0.929593000	-2.737752000	0.675800000
F	0.846897000	2.437772000	1.191797000
F	4.569876000	0.633617000	1.406727000
F	-3.686871000	0.785539000	1.124449000
F	0.225660000	1.729123000	-1.674313000
F	1.384952000	-1.560954000	-1.312520000
F	-1.319493000	1.145108000	0.445020000
F	2.418716000	1.092161000	-0.432456000
F	-2.076227000	-1.058270000	1.347004000
F	2.820918000	-1.368157000	0.532213000
F	4.739736000	-0.316253000	-1.292507000
F	-3.145665000	0.966090000	-1.266708000
F	-3.926746000	-1.263415000	-0.364505000

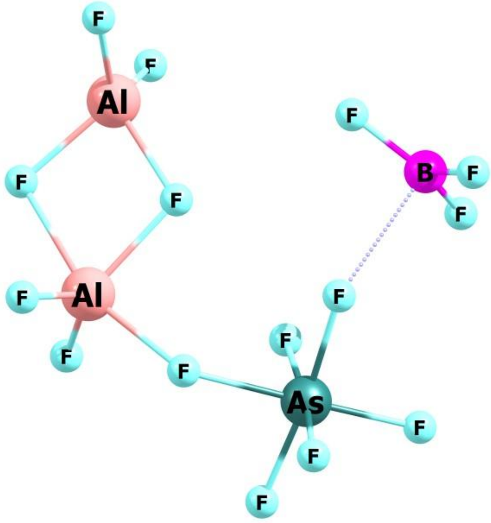

8.7

Al	3.112487000	0.664966000	-0.195861000
Al	1.426541000	-1.631011000	0.225719000
B	-0.989192000	2.946619000	0.138657000
As	-1.925777000	-0.654105000	-0.067934000
F	-1.374255000	0.796247000	0.782114000
F	-2.325021000	-2.162702000	-0.827055000
F	-0.904548000	-0.216757000	-1.406448000
F	3.292893000	1.384857000	-1.703481000
F	-0.417805000	-1.510596000	0.665485000

	Atom	X	Y	Z	
	F	-3.309332000	0.167877000	-0.710630000	
	F	0.348554000	2.839257000	0.210574000	
	F	3.321545000	-1.090273000	-0.227130000	
	F	-1.605688000	2.837054000	-1.044952000	
	F	1.878599000	-2.282319000	1.719241000	
	F	1.268167000	-2.518671000	-1.199075000	
	F	-1.665299000	3.411467000	1.203391000	
	F	3.889015000	1.413125000	1.094663000	
	F	1.408644000	0.228795000	0.190786000	
	F	-2.751107000	-1.140588000	1.381450000	
**$B_2P_2F_{17}^-$**	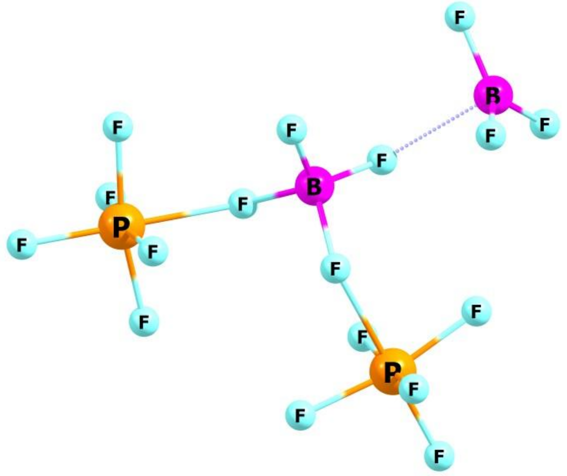				**0.0**
	B	2.928364000	2.342112000	0.070130000	
	B	0.131909000	0.917228000	-0.197000000	
	P	-2.942005000	0.285243000	0.061940000	
	P	1.347783000	-2.000408000	0.010794000	
	F	0.733483000	-1.453288000	1.408896000	
	F	-0.078664000	-2.686031000	-0.333740000	
	F	3.642450000	1.890570000	1.115675000	
	F	1.107331000	1.327093000	0.717257000	
	F	2.020525000	-3.330905000	0.628248000	
	F	2.645708000	-1.051771000	0.227517000	
	F	0.519889000	-0.376434000	-0.801021000	
	F	-0.128711000	1.801412000	-1.200492000	
	F	-4.475803000	-0.001667000	-0.353849000	
	F	2.394603000	3.576913000	0.131056000	
	F	1.824602000	-2.267827000	-1.519677000	
	F	-1.078707000	0.640584000	0.622000000	
	F	-2.956267000	1.846690000	-0.384559000	
	F	-3.271495000	0.741339000	1.588000000	
	F	-2.301805000	-0.106866000	-1.376080000	
	F	-2.640715000	-1.215039000	0.593874000	
	F	3.000462000	1.713090000	-1.113846000	

$B_2P_2F_{17}^-$

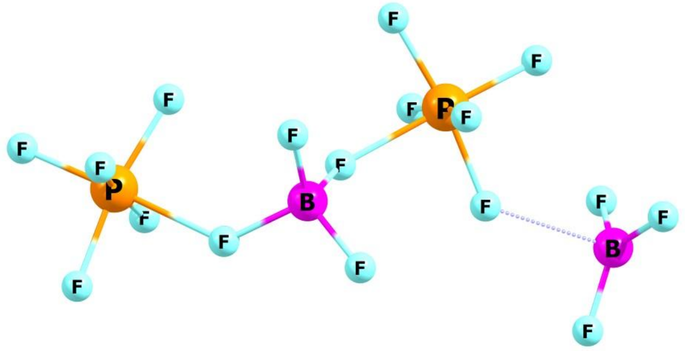

1.0

B	4.425637000	-1.252929000	0.208392000
P	-3.496634000	-0.318947000	0.377757000
B	-0.821528000	-0.089985000	-1.285038000
P	1.642057000	0.999417000	0.203001000
F	4.507687000	-0.845424000	1.483144000
F	-4.770538000	0.088517000	1.283756000
F	-4.113965000	-1.740033000	-0.120635000
F	2.282601000	-0.493565000	-0.108231000
F	3.090304000	1.689257000	0.405346000
F	-1.974464000	-0.873528000	-0.725686000
F	4.029558000	-2.507139000	-0.063699000
F	-4.052322000	0.377455000	-0.980843000
F	-2.711286000	-1.100103000	1.566855000
F	-0.123278000	-0.938208000	-2.092085000
F	-2.634461000	1.014839000	0.699920000
F	4.950675000	-0.499530000	-0.769190000
F	1.587353000	1.270713000	-1.396016000
F	1.480415000	0.572771000	1.759606000
F	-1.233561000	1.099273000	-1.791500000
F	0.796605000	2.355654000	0.472519000
F	-0.022643000	0.141000000	0.006946000

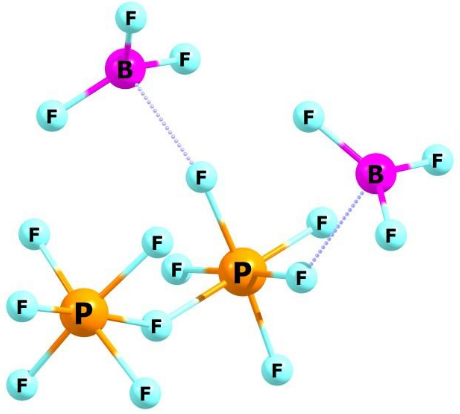

1.0

B	-1.478462000	-2.636890000	-1.054284000
B	-1.729268000	2.545663000	-1.042179000
P	2.418848000	0.092832000	-0.250452000
P	-0.746164000	-0.044412000	1.325433000
F	-1.459397000	-2.134092000	-2.300260000
F	-0.926327000	3.610782000	-0.878458000
F	-1.449400000	1.633516000	-1.978870000
F	-0.678425000	-1.569914000	1.891147000
F	-2.360215000	0.042325000	1.488911000
F	1.898357000	-1.341507000	-0.796696000

F	-0.417767000	-3.312760000	-0.594090000
F	3.616298000	0.250345000	-1.324629000
F	1.296542000	0.834483000	-1.147076000
F	3.321003000	-0.677163000	0.863203000
F	-2.925232000	2.528425000	-0.429945000
F	-2.640765000	-2.681220000	-0.381378000
F	-0.468681000	0.552710000	2.814888000
F	-0.872272000	-0.621625000	-0.218269000
F	2.713721000	1.495679000	0.516253000
F	-0.666839000	1.486187000	0.705990000
F	1.013664000	-0.126190000	1.142346000

$B_2P_2F_{17}^-$

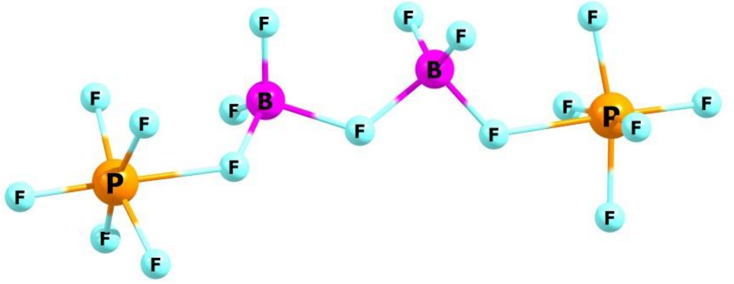

1.1

B	1.343505000	0.678731000	-0.305806000
B	-1.342608000	0.676457000	0.306896000
P	-4.254825000	-0.380727000	-0.053235000
P	4.254423000	-0.381167000	0.052414000
F	-4.080448000	1.132693000	0.506362000
F	1.406353000	1.746996000	0.530019000
F	-1.407976000	1.751058000	-0.520680000
F	-2.258425000	-0.407009000	-0.124724000
F	4.136844000	0.173686000	1.572449000
F	-1.391616000	0.866037000	1.649747000
F	4.081127000	1.136295000	-0.496580000
F	-4.140127000	0.185117000	-1.569435000
F	-4.049773000	-0.943438000	1.454776000
F	-5.865792000	-0.397476000	-0.011141000
F	5.865476000	-0.397578000	0.013326000
F	4.052405000	-0.933001000	-1.460044000
F	-4.081460000	-1.891178000	-0.621878000
F	4.080053000	-1.895689000	0.609796000
F	1.395253000	0.878202000	-1.647124000
F	0.000010000	-0.079891000	-0.003871000
F	2.258266000	-0.407881000	0.119767000

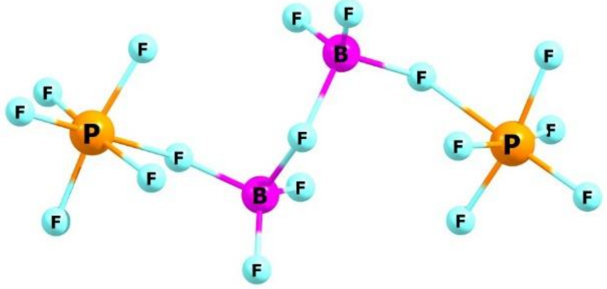

1.1

B	-0.661683000	1.212484000	-0.493410000
B	0.660809000	-1.210381000	-0.495018000
P	3.445566000	0.120203000	0.258433000
P	-3.444725000	-0.121370000	0.258352000

	F	4.133883000	0.355136000	-1.193374000	
	F	4.036296000	-1.393141000	0.269719000	
	F	4.647612000	0.767233000	1.115824000	
	F	2.610635000	1.496235000	0.070061000	
	F	-0.749423000	2.196252000	0.438521000	
	F	0.000199000	0.001409000	0.231632000	
	F	-4.646052000	-0.770463000	1.115320000	
	F	-4.037362000	1.391254000	0.271035000	
	F	-2.509610000	0.252866000	1.528062000	
	F	-1.992236000	0.676585000	-0.860424000	
	F	0.746904000	-2.194797000	0.436302000	
	F	-4.133108000	-0.356022000	-1.193503000	
	F	0.033266000	1.458635000	-1.637531000	
	F	-0.034952000	-1.454393000	-1.639086000	
	F	1.991524000	-0.675499000	-0.861334000	
	F	-2.608397000	-1.496445000	0.068934000	
	F	2.509904000	-0.254066000	1.527660000	
**$B_2P_2F_{17}^-$**	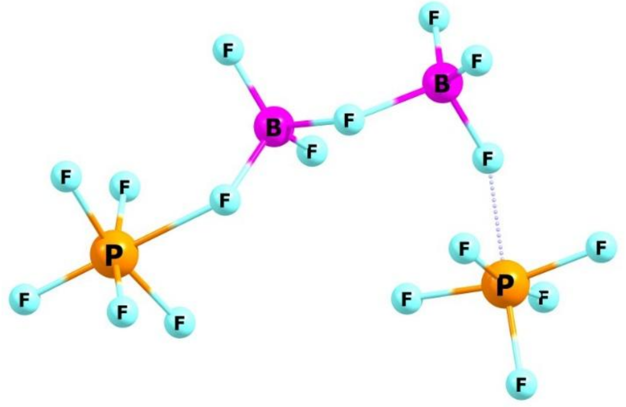				**1.4**
	B	0.820937000	1.317928000	-0.224206000	
	B	-1.846257000	1.913222000	0.165645000	
	P	3.265337000	-0.576512000	0.069942000	
	P	-2.734998000	-1.155138000	-0.022734000	
	F	3.293333000	0.488065000	-1.156441000	
	F	1.476955000	0.197854000	0.484539000	
	F	-0.480972000	1.365760000	0.668994000	
	F	-4.254270000	-0.633081000	0.218486000	
	F	-3.067713000	-1.311006000	-1.602589000	
	F	-1.594421000	2.942193000	-0.697034000	
	F	3.821295000	0.583704000	1.061547000	
	F	4.710516000	-1.224230000	-0.234711000	
	F	-2.351042000	-0.648914000	1.468278000	
	F	-2.407375000	0.782694000	-0.580422000	
	F	-1.162322000	-1.332662000	-0.348606000	
	F	0.446668000	0.982624000	-1.482605000	
	F	1.471904000	2.492285000	-0.012847000	
	F	2.934584000	-1.497655000	1.364081000	
	F	-3.019071000	-2.689069000	0.371305000	
	F	-2.559836000	2.196853000	1.292521000	
	F	2.427490000	-1.604417000	-0.860641000	

$B_2P_2F_{17}^-$	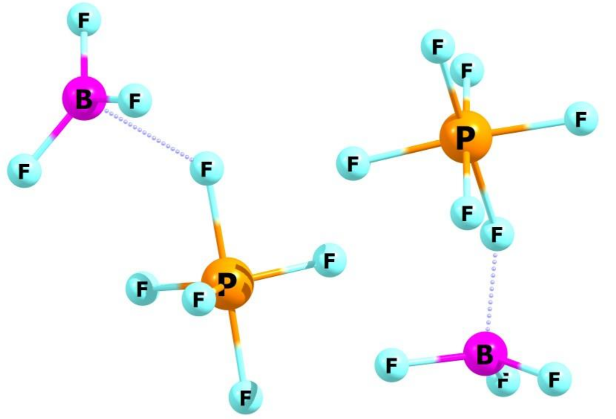	**8.0**

B	-2.308212000	1.703888000	-0.368914000
B	3.423111000	-1.643768000	-0.394655000
P	1.605877000	1.413739000	0.263836000
P	-1.948731000	-1.406736000	0.157579000
F	-0.403947000	-0.979739000	-0.193231000
F	-3.513994000	-1.683226000	0.548586000
F	3.008399000	1.952534000	0.809940000
F	-2.387963000	-1.061234000	-1.375703000
F	4.194675000	-0.633619000	-0.837668000
F	2.456349000	-2.131184000	-1.170741000
F	3.741881000	-2.247342000	0.759551000
F	1.588601000	0.875856000	-1.222663000
F	-3.499755000	1.605357000	-1.040606000
F	-1.167802000	1.620158000	-1.139490000
F	-2.270744000	2.651181000	0.633765000
F	-2.247449000	0.310965000	0.574836000
F	-1.671068000	-2.978741000	-0.181212000
F	0.406934000	1.428829000	1.286827000
F	1.230700000	2.902893000	-0.247765000
F	-1.528993000	-1.588207000	1.731364000
F	2.016212000	-0.089553000	0.786057000

$B_2As_2F_{17}^-$	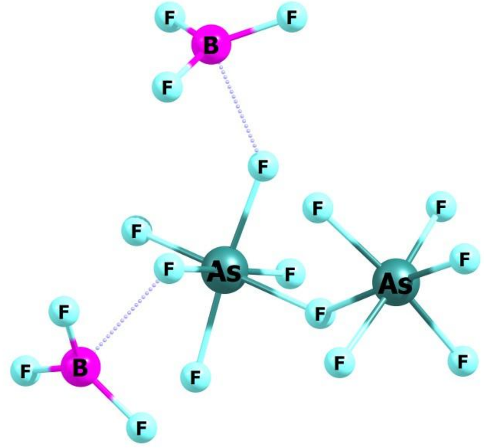	**0.0**

B	-2.167866000	2.333153000	1.371966000
B	-0.916152000	-2.998553000	1.224388000
As	2.306845000	0.346194000	0.135216000
As	-1.072303000	-0.077109000	-1.180027000
F	-2.086087000	1.812808000	2.608137000
F	0.310963000	-3.532729000	1.147421000
F	-1.177458000	-2.022569000	2.100146000

$B_2As_2F_{17}^-$	F	-1.488359000	1.517500000	-1.719303000	
	F	-2.688915000	-0.677569000	-1.329446000	
	F	1.858165000	1.877571000	0.816958000	
	F	-1.189240000	3.124625000	0.915463000	
	F	3.748932000	0.181661000	1.077388000	
	F	1.283613000	-0.451924000	1.279893000	
	F	3.092873000	1.179685000	-1.172832000	
	F	-1.931610000	-3.594677000	0.573537000	
	F	-3.331924000	2.255896000	0.704610000	
	F	-0.624350000	-0.536631000	-2.791167000	
	F	-1.324720000	0.422982000	0.483445000	
	F	2.516353000	-1.151285000	-0.715667000	
	F	-0.469235000	-1.610839000	-0.587000000	
	F	0.687688000	0.588515000	-1.003026000	
	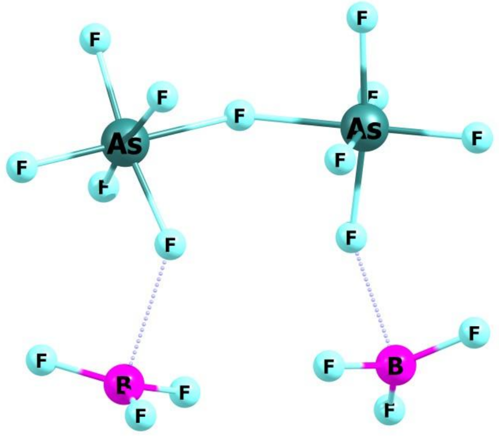				**0.02**
	B	2.106345000	2.581250000	0.237828000	
	B	-2.008051000	2.635061000	-0.251679000	
	As	-1.851699000	-1.025695000	0.103221000	
	As	1.812670000	-1.064598000	-0.093384000	
	F	1.967650000	3.133659000	-0.978021000	
	F	-3.207819000	2.261281000	-0.726579000	
	F	-1.938714000	3.237376000	0.947360000	
	F	2.093729000	-0.514390000	1.528580000	
	F	3.440605000	-0.782325000	-0.606003000	
	F	-1.555101000	0.589406000	0.733889000	
	F	1.137838000	2.719143000	1.151746000	
	F	-3.527834000	-0.720245000	-0.194933000	
	F	-1.337902000	-0.477705000	-1.454511000	
	F	-2.123564000	-1.571666000	1.728867000	
	F	-0.939567000	2.612326000	-1.054589000	
	F	3.304404000	2.096072000	0.601572000	
	F	2.111213000	-2.700960000	0.406689000	
	F	1.280481000	0.553452000	-0.518292000	
	F	-1.911604000	-2.663659000	-0.467788000	
	F	1.290332000	-1.623811000	-1.646348000	
	F	0.004351000	-1.381494000	0.519987000	

$B_2As_2F_{17}^-$

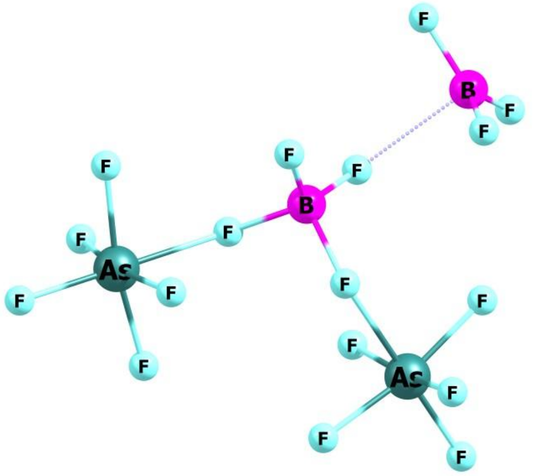

0.8

B	-2.755879000	2.885423000	-0.058685000
B	-0.124430000	1.083213000	0.182750000
As	2.904385000	0.074217000	-0.045042000
As	-1.747646000	-1.678744000	-0.008281000
F	-1.015779000	-1.218924000	-1.510445000
F	-0.355083000	-2.634492000	0.367473000
F	-3.522350000	2.521834000	-1.097895000
F	-1.026878000	1.597612000	-0.737239000
F	-2.669131000	-2.987666000	-0.655660000
F	-2.978742000	-0.484938000	-0.258888000
F	-0.674264000	-0.164277000	0.791061000
F	0.246795000	1.915890000	1.189458000
F	4.501385000	-0.400700000	0.409868000
F	-2.049459000	4.027252000	-0.134387000
F	-2.300246000	-1.875138000	1.625182000
F	1.054451000	0.630472000	-0.620553000
F	3.108238000	1.734324000	0.414503000
F	3.315604000	0.500900000	-1.677101000
F	2.179121000	-0.252011000	1.494176000
F	2.423252000	-1.493905000	-0.595056000
F	-2.878119000	2.262233000	1.122096000

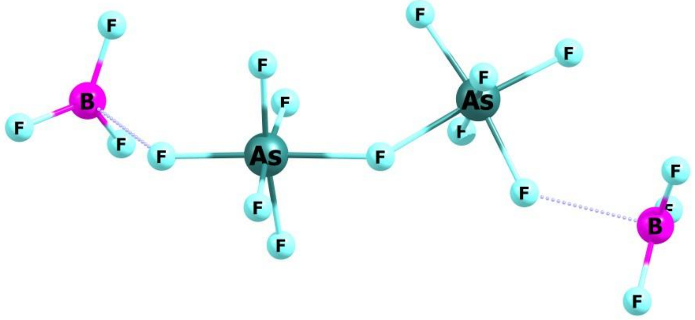

0.9

B	-4.852326000	0.873217000	0.538684000
B	4.968091000	-0.390236000	0.746899000
As	1.647979000	0.610781000	-0.383206000
As	-1.697492000	-0.800935000	-0.135892000
F	-4.833831000	0.201484000	1.700323000
F	5.939037000	0.370457000	0.206002000
F	4.512184000	-0.097588000	1.973653000
F	-2.655553000	0.656679000	0.143586000
F	-3.042147000	-1.851033000	0.147101000

$B_2As_2F_{17}^-$

F	1.400041000	1.732979000	0.917644000
F	-4.738504000	2.213515000	0.549136000
F	3.394749000	0.804723000	-0.343585000
F	1.664199000	-0.695161000	0.750722000
F	1.450558000	1.909066000	-1.520146000
F	4.651881000	-1.559647000	0.172530000
F	-5.242843000	0.252984000	-0.585670000
F	-2.025088000	-0.636031000	-1.831371000
F	-1.216972000	-0.756328000	1.526280000
F	1.706403000	-0.518265000	-1.696613000
F	-0.573000000	-2.072926000	-0.459326000
F	-0.273881000	0.473999000	-0.461117000

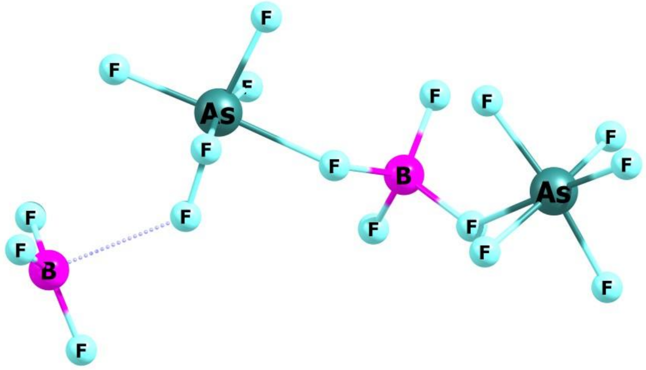

1.0

B	4.597989000	-1.480522000	0.271386000
As	-3.433600000	-0.254601000	0.309222000
B	-0.667331000	-0.241562000	-1.323430000
As	1.862536000	0.937673000	0.128066000
F	4.709996000	-0.980240000	1.510990000
F	-4.811229000	0.293350000	1.196153000
F	-4.116572000	-1.798894000	-0.099696000
F	2.487260000	-0.696881000	-0.064637000
F	3.433347000	1.630203000	0.309119000
F	-1.861816000	-0.947260000	-0.716674000
F	4.174941000	-2.744156000	0.101273000
F	-3.981218000	0.368800000	-1.214386000
F	-2.654838000	-0.985371000	1.676860000
F	0.022903000	-1.181462000	-2.023404000
F	-2.493810000	1.178425000	0.553231000
F	5.125998000	-0.814939000	-0.767031000
F	1.831198000	1.084875000	-1.596807000
F	1.645213000	0.617988000	1.814588000
F	-1.046460000	0.900181000	-1.944459000
F	1.010488000	2.434859000	0.284370000
F	0.101466000	0.092637000	-0.038409000

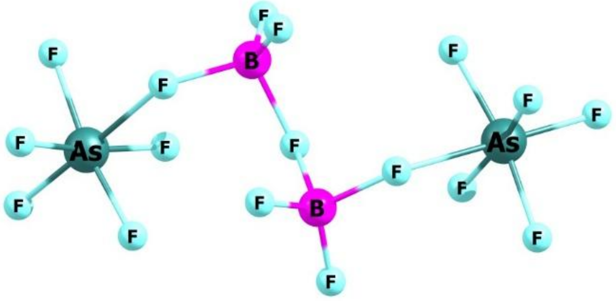

2.1

B	-0.620004000	1.229142000	-0.535894000

$B_2As_2F_{17}^-$

B	0.619935000	-1.229024000	-0.536159000
As	3.457968000	0.087985000	0.213273000
As	-3.457801000	-0.088063000	0.213262000
F	4.179950000	0.368046000	-1.338734000
F	4.112454000	-1.519273000	0.204735000
F	4.752881000	0.779025000	1.120367000
F	2.561005000	1.560815000	0.050847000
F	-0.697266000	2.215211000	0.386992000
F	-0.000294000	0.000214000	0.190189000
F	-4.752349000	-0.780069000	1.120199000
F	-4.114485000	1.518298000	0.203079000
F	-2.489726000	0.331401000	1.586549000
F	-1.979222000	0.714151000	-0.901100000
F	0.697704000	-2.214674000	0.387047000
F	-4.178686000	-0.370498000	-1.338844000
F	0.062882000	1.445329000	-1.688562000
F	-0.063460000	-1.445479000	-1.688392000
F	1.978541000	-0.713107000	-0.901558000
F	-2.558892000	-1.559949000	0.052649000
F	2.488389000	-0.329222000	1.586161000

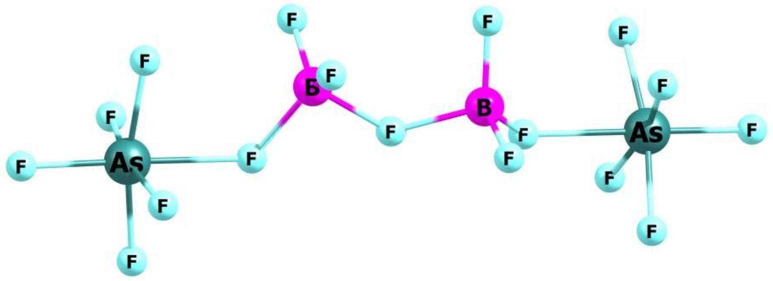

2.2

B	1.333853000	-0.765553000	0.319116000
B	-1.333482000	-0.765845000	-0.319074000
As	-4.279532000	0.329198000	0.041804000
As	4.279432000	0.329190000	-0.041820000
F	-4.114588000	-1.287340000	-0.562283000
F	1.407332000	-1.830719000	-0.514403000
F	-1.407905000	-1.831213000	0.514062000
F	-2.258707000	0.328936000	0.112523000
F	4.178690000	-0.282213000	-1.660524000
F	-1.382763000	-0.947316000	-1.659140000
F	4.114085000	-1.287393000	0.562089000
F	-4.178594000	-0.282379000	1.660436000
F	-4.068615000	0.935613000	-1.568327000
F	-6.003306000	0.363479000	-0.004180000
F	6.003194000	0.363213000	0.004549000
F	4.068184000	0.935499000	1.568296000
F	-4.086734000	1.939164000	0.655420000
F	4.087018000	1.939239000	-0.655344000
F	1.384386000	-0.947441000	1.659103000
F	-0.000096000	-0.001709000	0.000803000
F	2.258576000	0.329267000	-0.113045000

$B_2PAsF_{17}^-$

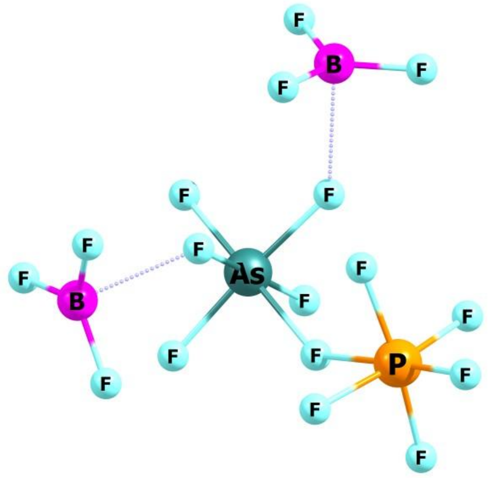

0.0

B	2.090277000	-2.217197000	1.343195000
B	0.568973000	2.990296000	1.213871000
P	-2.455206000	-0.544800000	0.193251000
As	0.825974000	0.109088000	-1.174535000
F	1.989040000	-1.714806000	2.586584000
F	-0.641846000	3.555720000	1.083277000
F	0.767942000	2.027099000	2.120501000
F	1.353446000	-1.446056000	-1.736739000
F	2.402657000	0.813774000	-1.329322000
F	-1.894144000	-1.946844000	0.788580000
F	1.159633000	-3.069840000	0.893385000
F	-3.783104000	-0.501848000	1.112550000
F	-1.510046000	0.259736000	1.231005000
F	-3.146665000	-1.364768000	-1.030295000
F	1.625267000	3.559375000	0.605790000
F	3.246901000	-2.075379000	0.672767000
F	0.349626000	0.567753000	-2.780000000
F	1.142189000	-0.383703000	0.485202000
F	-2.760728000	0.843370000	-0.590638000
F	0.148392000	1.607420000	-0.557018000
F	-0.862483000	-0.652492000	-0.991675000

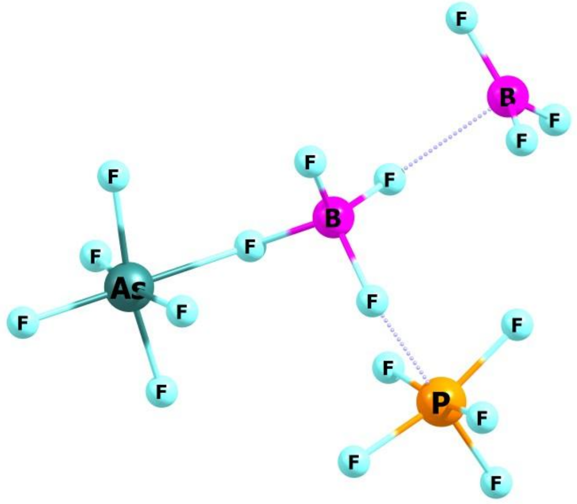

0.2

B	3.143006000	-2.408186000	-0.080204000
B	0.375350000	-0.907826000	0.221694000
As	-2.724900000	-0.183350000	-0.052440000

	Atom	X	Y	Z	
$B_2PAsF_{17}^-$	P	1.700599000	1.985136000	-0.013662000	
	F	1.044556000	1.456787000	-1.398087000	
	F	0.304855000	2.715679000	0.352056000	
	F	3.857785000	-1.952355000	-1.122186000	
	F	1.312041000	-1.344946000	-0.715032000	
	F	2.421751000	3.284824000	-0.634178000	
	F	2.946474000	0.973495000	-0.240069000	
	F	0.792762000	0.360776000	0.819282000	
	F	0.068901000	-1.798417000	1.203758000	
	F	-4.362425000	0.159539000	0.385063000	
	F	2.584434000	-3.629887000	-0.155973000	
	F	2.197212000	2.215349000	1.514680000	
	F	-0.850656000	-0.580907000	-0.604196000	
	F	-2.810247000	-1.857423000	0.396630000	
	F	-3.091351000	-0.626604000	-1.692106000	
	F	-2.057024000	0.191967000	1.502482000	
	F	-2.375083000	1.427502000	-0.584168000	
	F	3.218343000	-1.789425000	1.108488000	
	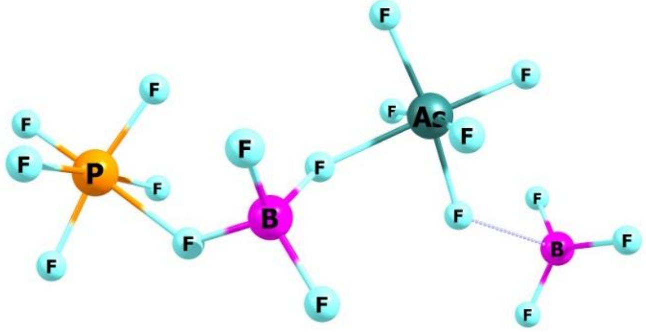				**0.6**
	B	-4.314599000	1.420850000	0.289453000	
	P	3.692154000	0.301441000	0.402701000	
	B	1.007397000	0.327024000	-1.277622000	
	As	-1.534739000	-0.927780000	0.103366000	
	F	-4.430995000	0.882746000	1.513080000	
	F	4.957986000	-0.232430000	1.248966000	
	F	4.331770000	1.757187000	0.060713000	
	F	-2.199021000	0.697769000	-0.047891000	
	F	-3.094666000	-1.657669000	0.243824000	
	F	2.161682000	1.017403000	-0.633652000	
	F	-3.920362000	2.699402000	0.162272000	
	F	4.221445000	-0.245471000	-1.031521000	
	F	2.922100000	0.961792000	1.670798000	
	F	0.305120000	1.264540000	-1.972907000	
	F	2.796408000	-1.037251000	0.572366000	
	F	-4.822936000	0.778201000	-0.773336000	
	F	-1.487552000	-1.026803000	-1.624737000	
	F	-1.355114000	-0.658108000	1.804514000	
	F	1.389780000	-0.808526000	-1.912657000	
	F	-0.659868000	-2.416160000	0.228596000	
	F	0.195345000	-0.048206000	-0.009620000	

$B_2PAsF_{17}^-$	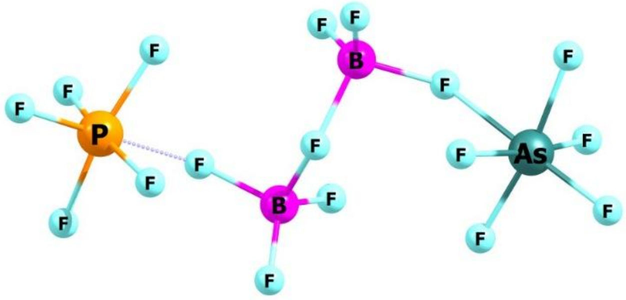	**1.5**
	B -0.979799000 1.261687000 -0.489350000<br>B 0.304234000 -1.180595000 -0.545222000<br>As 3.164179000 0.073684000 0.206200000<br>P -3.747700000 -0.140977000 0.270788000<br>F 3.892076000 0.373050000 -1.340501000<br>F 3.786697000 -1.546580000 0.163078000<br>F 4.480173000 0.714384000 1.122875000<br>F 2.306469000 1.573732000 0.083443000<br>F -1.051420000 2.206708000 0.480523000<br>F -0.300518000 0.013915000 0.208156000<br>F -4.926404000 -0.835906000 1.118644000<br>F -4.366744000 1.358968000 0.314717000<br>F -2.807203000 0.230790000 1.535494000<br>F -2.299137000 0.728230000 -0.854925000<br>F 0.395171000 -2.202194000 0.340806000<br>F -4.430486000 -0.354932000 -1.185294000<br>F -0.276825000 1.531805000 -1.621113000<br>F -0.375463000 -1.373835000 -1.706272000<br>F 1.671923000 -0.663754000 -0.912232000<br>F -2.874402000 -1.485532000 0.040989000<br>F 2.195586000 -0.349110000 1.578997000	
	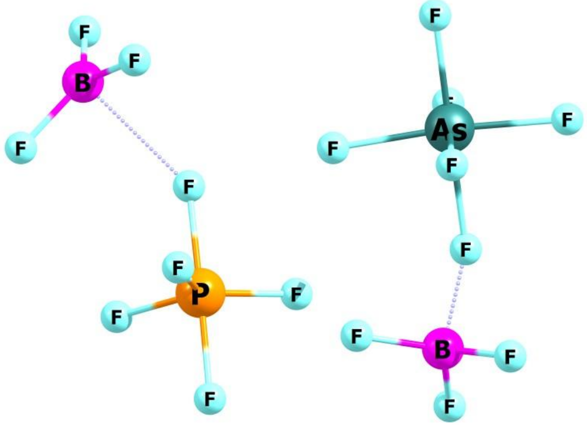	**7.1**
	B -1.943591000 2.075975000 -0.395558000<br>B 3.508842000 -1.812679000 -0.422441000<br>P 1.918717000 1.357145000 0.289262000<br>As -1.940055000 -1.145124000 0.126509000<br>F -0.258676000 -0.864733000 -0.256320000<br>F -3.627110000 -1.267802000 0.554940000<br>F 3.349646000 1.775736000 0.865841000<br>F -2.377610000 -0.739512000 -1.508020000<br>F 4.359142000 -0.853800000 -0.833859000<br>F 2.515965000 -2.206500000 -1.218652000<br>F 3.767690000 -2.465071000 0.719987000	

	F 1.878224000 0.855393000 -1.209365000<br>F -3.135769000 2.116852000 -1.069849000<br>F -0.820691000 1.834853000 -1.156177000<br>F -1.791622000 3.011369000 0.604885000<br>F -2.067492000 0.677395000 0.555251000<br>F -1.819591000 -2.845820000 -0.226449000<br>F 0.706665000 1.438747000 1.293865000<br>F 1.664816000 2.881587000 -0.191442000<br>F -1.505177000 -1.374193000 1.802407000<br>F 2.207678000 -0.183900000 0.781432000	
**$Al_2P_2F_{17}^-$**	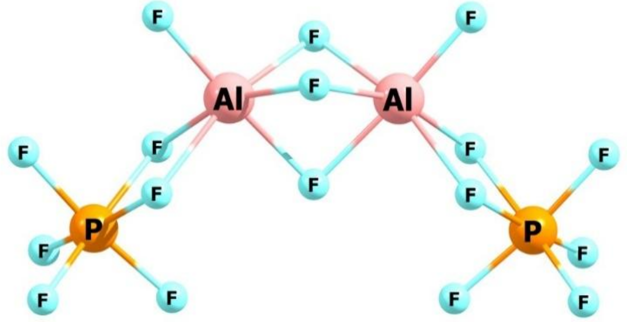	**0.0**
	Al 1.288659000 -1.233535000 -0.001113000<br>Al -1.288870000 -1.233759000 0.001126000<br>P -3.338036000 0.858261000 -0.000117000<br>P 3.338168000 0.858126000 0.000116000<br>F -2.380806000 -0.077233000 1.143567000<br>F 0.001032000 -1.834578000 1.222970000<br>F -0.000031000 0.138184000 0.000014000<br>F -2.381555000 -0.079300000 -1.142679000<br>F 4.408307000 -0.356850000 -0.001370000<br>F -2.376886000 -2.533620000 0.003136000<br>F 2.381602000 -0.079343000 1.142691000<br>F -2.147592000 1.954005000 -0.001482000<br>F 2.147844000 1.954016000 0.001468000<br>F -4.102355000 1.606212000 1.212199000<br>F 4.103396000 1.603798000 1.213259000<br>F -4.103150000 1.604020000 -1.213282000<br>F 4.102569000 1.605967000 -1.212200000<br>F -4.408296000 -0.356626000 0.001348000<br>F 2.380843000 -0.077280000 -1.143569000<br>F -0.001309000 -1.834561000 -1.222964000<br>F 2.376470000 -2.533585000 -0.003121000	
	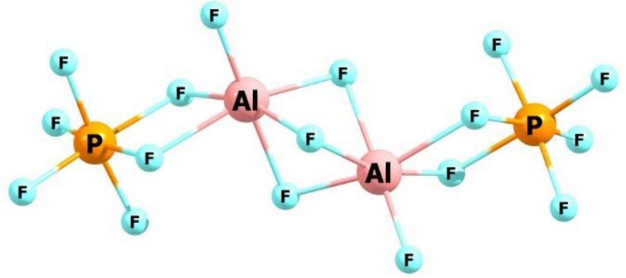	**0.3**
	Al -1.135550000 0.618440000 -0.607688000<br>Al 1.135789000 -0.621136000 -0.604975000<br>P 3.766648000 0.157711000 0.422335000<br>P -3.766851000 -0.155905000 0.422864000	

$Al_2P_2F_{17}^-$	F	1.663480000	-2.092355000	-1.262908000	
	F	-0.494327000	-1.052104000	0.121259000	
	F	0.000166000	-0.003079000	-1.958850000	
	F	2.780335000	0.360085000	-1.021460000	
	F	-3.105004000	-1.518406000	0.994119000	
	F	2.316911000	-0.686894000	0.955169000	
	F	-2.780180000	-0.362810000	-1.020379000	
	F	4.279666000	-1.244919000	-0.200570000	
	F	3.103389000	1.520916000	0.990096000	
	F	4.491651000	-0.141666000	1.835932000	
	F	-4.984325000	-0.970079000	-0.261209000	
	F	-4.278378000	1.245497000	-0.204026000	
	F	4.983690000	0.971263000	-0.263231000	
	F	-4.492379000	0.147559000	1.835293000	
	F	-1.661793000	2.088565000	-1.269245000	
	F	0.493966000	1.050456000	0.119216000	
	F	-2.316877000	0.688855000	0.953754000	
	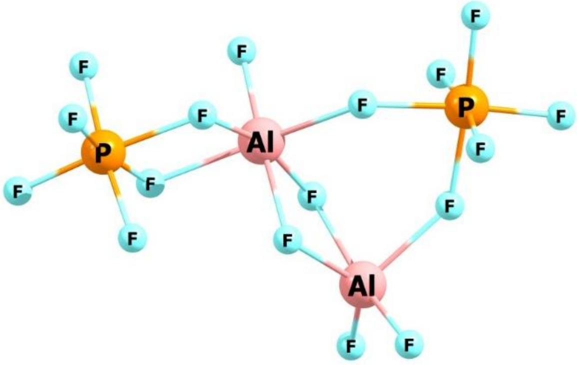				**3.4**
	Al	-0.487322000	-0.224632000	-0.631204000	
	Al	1.212674000	2.057668000	-0.039799000	
	P	-3.280833000	-0.247395000	0.255005000	
	P	2.758809000	-1.024102000	0.199940000	
	F	1.755789000	2. 689231000	1.448040000	
	F	-3.476516000	-1.427324000	-0.833390000	
	F	-2.254294000	0.570891000	-0.920224000	
	F	2.610330000	-1.176583000	-1.401059000	
	F	2.606135000	0.716228000	-0.045921000	
	F	2.723246000	-0.712543000	1.785629000	
	F	-4.025351000	-1.103199000	1.404630000	
	F	-2.918256000	0.939688000	1.295458000	
	F	4.362865000	-0.851190000	0.117564000	
	F	1.268612000	3.368881000	-1.117215000	
	F	-1.733574000	-0.983718000	0.670445000	
	F	-4.579106000	0.547406000	-0.285357000	
	F	-0.673496000	-1.463506000	-1.769804000	
	F	0.570077000	0.931472000	-1.509197000	
	F	-0.207758000	1.125165000	0.650941000	
	F	1.001829000	-1.078037000	0.286398000	
	F	2.791778000	-2.621419000	0.434044000	

$Al_2P_2F_{17}^-$

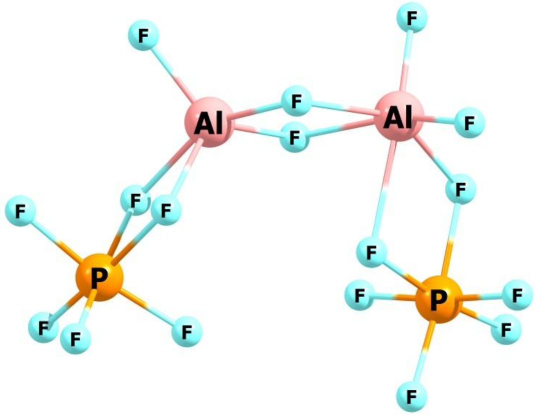

4.5

Al	-1.157122000	-1.510098000	-0.204972000
Al	1.724419000	-1.603502000	0.181523000
P	-2.903807000	0.814231000	0.107700000
P	2.371139000	1.328646000	-0.109479000
F	-2.199302000	-0.137233000	-1.177169000
F	-1.973916000	-0.392362000	1.066190000
F	0.051570000	-2.034905000	1.014456000
F	3.517060000	1.998537000	-1.043225000
F	1.280676000	1.456805000	-1.307806000
F	2.045158000	-2.954963000	-0.807029000
F	-4.120961000	-0.257481000	0.126502000
F	-3.658640000	1.774201000	-0.938080000
F	3.444835000	1.063443000	1.076599000
F	2.784599000	-0.268151000	-0.749898000
F	1.228364000	0.397696000	0.773633000
F	0.261863000	-1.072115000	-1.129994000
F	-2.132161000	-2.817973000	-0.600073000
F	-3.423416000	1.506374000	1.465569000
F	1.863979000	2.705842000	0.574045000
F	2.652819000	-1.739242000	1.599807000
F	-1.554177000	1.697487000	0.093308000

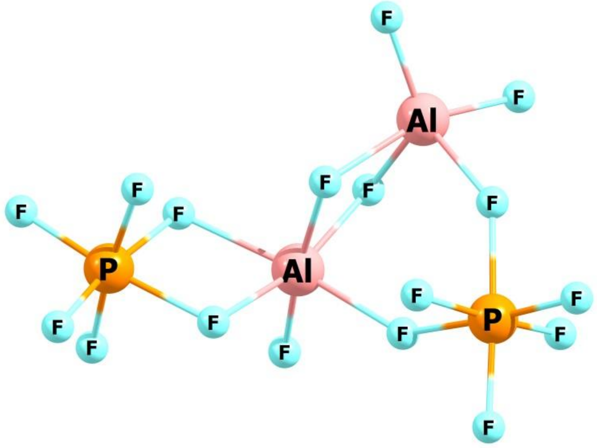

5.3

Al	1.405157000	2.050982000	-0.165115000
Al	-0.512933000	0.154168000	0.869281000
P	-3.163013000	-0.289625000	-0.281251000
P	2.517534000	-1.236687000	-0.210288000
F	1.542094000	-1.425800000	-1.486346000
F	2.712969000	2.772355000	0.656642000
F	-0.869822000	-0.440291000	2.414224000

$Al_2P_2F_{17}^-$	F	-4.474985000	0.650941000	-0.332847000	
	F	3.852086000	-1.363867000	-1.112875000	
	F	-3.565206000	-0.871537000	1.174354000	
	F	1.074959000	-0.975180000	0.758556000	
	F	-2.596683000	0.321944000	-1.668091000	
	F	3.356391000	-0.873411000	1.124282000	
	F	2.447597000	-2.806131000	0.170009000	
	F	0.053979000	0.807492000	-0.730135000	
	F	-2.258604000	0.971977000	0.556980000	
	F	-3.796565000	-1.569755000	-1.034049000	
	F	-1.619036000	-1.120630000	-0.104576000	
	F	0.983269000	3.130414000	-1.403311000	
	F	2.506613000	0.488015000	-0.550915000	
	F	0.437976000	1.662100000	1.370203000	
	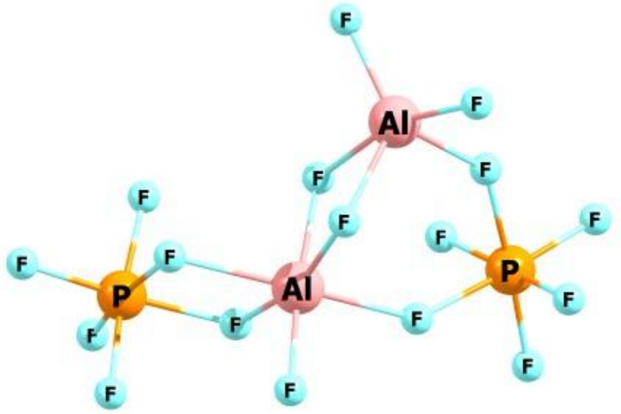				**5.3**
	Al	1.403214000	2.052326000	0.163602000	
	Al	-0.512554000	0.150677000	-0.867192000	
	P	2.519989000	-1.235598000	0.210841000	
	P	-3.164251000	-0.289704000	0.280659000	
	F	2.451429000	-2.806444000	-0.163661000	
	F	-2.599100000	0.322473000	1.667735000	
	F	0.053942000	0.806775000	0.730687000	
	F	-3.564892000	-0.872766000	-1.174887000	
	F	0.437703000	1.658919000	-1.370766000	
	F	0.978697000	3.131480000	1.401225000	
	F	3.355152000	-0.875958000	-1.127019000	
	F	-4.476225000	0.650879000	0.330516000	
	F	-3.798406000	-1.569418000	1.033732000	
	F	1.548095000	-1.421620000	1.490088000	
	F	-0.868990000	-0.444679000	-2.412007000	
	F	2.507025000	0.490461000	0.544719000	
	F	2.709553000	2.777274000	-0.657519000	
	F	1.074774000	-0.979344000	-0.755309000	
	F	-2.258683000	0.970761000	-0.557238000	
	F	3.856937000	-1.357261000	1.110569000	
	F	-1.619751000	-1.121479000	0.106267000	

$Al_2P_2F_{17}^-$	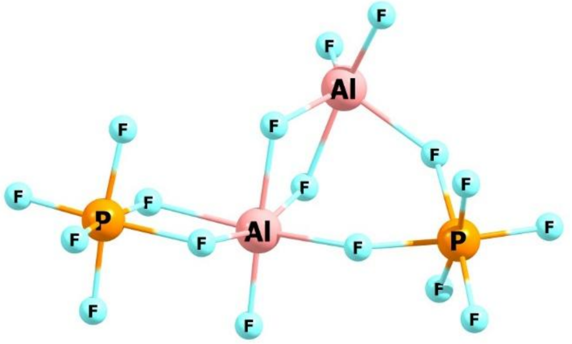	**6.2**

Al	-1.212511000	2.057590000	-0.039900000
Al	0.487547000	-0.225064000	-0.631124000
P	3.280861000	-0.247311000	0.255005000
P	-2.758947000	-1.023892000	0.199978000
F	4.579012000	0.547510000	-0.285604000
F	3.476464000	-1.427476000	-0.833163000
F	4.025480000	-1.102863000	1.404725000
F	2.918291000	0.939837000	1.295330000
F	-1.755660000	2.689094000	1.447932000
F	0.207714000	1.124899000	0.651106000
F	-2.792133000	-2.621130000	0.434690000
F	-4.362990000	-0.850714000	0.117975000
F	-2.723004000	-0.711746000	1.785538000
F	-2.606053000	0.716281000	-0.046562000
F	0.673469000	-1.463955000	-1.769723000
F	-2.610952000	-1.176930000	-1.401021000
F	-1.268509000	3.368619000	-1.117472000
F	-0.569530000	0.931308000	-1.509279000
F	2.254118000	0.570652000	-0.920229000
F	-1.002013000	-1.077996000	0.286010000
F	1.733608000	-0.983700000	0.670698000

$Al_2As_2F_{17}^-$	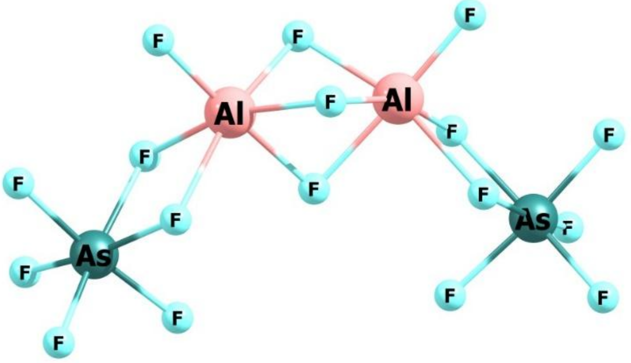	**0.0**

Al	-1.288296000	1.404990000	-0.001279000
Al	1.288589000	1.405321000	0.001268000
As	3.374374000	-0.755018000	-0.000203000
As	-3.374536000	-0.754866000	0.000206000
F	2.359934000	0.257245000	1.177525000
F	-0.001126000	2.011518000	1.219600000
F	0.000049000	0.032333000	-0.000034000
F	2.360867000	0.259743000	-1.176544000
F	-4.534741000	0.525149000	-0.001590000
F	2.392192000	2.691963000	0.003591000
F	-2.360899000	0.259778000	1.176545000

	F	2.095510000	-1.914541000	-0.001933000	
	F	-2.095764000	-1.914497000	0.001919000	
	F	4.175781000	-1.555850000	1.302848000	
	F	-4.177045000	-1.552876000	1.304304000	
	F	4.176823000	-1.553092000	-1.304302000	
	F	-4.176030000	-1.555615000	-1.302839000	
	F	4.534725000	0.524874000	0.001623000	
	F	-2.360001000	0.257297000	-1.177536000	
	F	0.001503000	2.011563000	-1.219590000	
	F	-2.391608000	2.691908000	-0.003582000	
$Al_2As_2F_{17}^-$	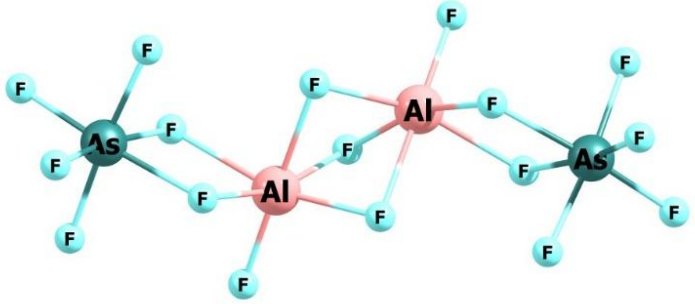				**0.3**
	Al	1.125405000	0.631056000	0.697772000	
	Al	-1.125558000	-0.641451000	0.688897000	
	As	-3.830002000	0.125479000	-0.371930000	
	As	3.830120000	-0.119933000	-0.373599000	
	F	-1.643008000	-2.122690000	1.331887000	
	F	0.514771000	-1.043576000	-0.037391000	
	F	0.000110000	-0.014394000	2.046549000	
	F	-2.775369000	0.321753000	1.141090000	
	F	3.142729000	-1.581093000	-0.987379000	
	F	-2.274392000	-0.735592000	-0.899329000	
	F	2.775219000	-0.337699000	1.136414000	
	F	-4.370991000	-1.380733000	0.276581000	
	F	-3.141369000	1.594369000	-0.965560000	
	F	-4.581830000	-0.197862000	-1.892464000	
	F	5.137491000	-0.979513000	0.356748000	
	F	4.369661000	1.377707000	0.295630000	
	F	-5.137054000	0.975968000	0.369558000	
	F	4.582308000	0.224519000	-1.889302000	
	F	1.642275000	2.103756000	1.360479000	
	F	-0.514892000	1.042651000	-0.023361000	
	F	2.274130000	0.747111000	-0.889508000	
	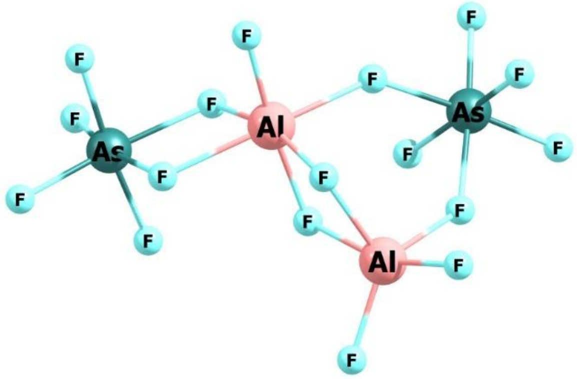				**4.2**
	Al	1.337672000	2.243703000	-0.197954000	

$Al_2As_2F_{17}^-$

Al	-0.465484000	0.275139000	0.934739000
As	-3.163841000	-0.320812000	-0.231701000
As	2.676435000	-1.029626000	-0.164604000
F	1.628388000	-1.345575000	-1.498180000
F	2.604045000	3.080516000	0.578065000
F	-0.815956000	-0.264813000	2.501216000
F	-4.591149000	0.643655000	-0.330157000
F	4.094148000	-1.111836000	-1.145821000
F	-3.587033000	-0.890997000	1.342556000
F	1.156932000	-0.805481000	0.870274000
F	-2.576043000	0.286885000	-1.736862000
F	3.559504000	-0.527648000	1.231020000
F	2.710460000	-2.689356000	0.308985000
F	0.073646000	0.878159000	-0.694496000
F	-2.230680000	1.053283000	0.600548000
F	-3.780392000	-1.748311000	-0.978638000
F	-1.498052000	-1.103471000	0.014296000
F	0.802578000	3.251958000	-1.451253000
F	2.548561000	0.769414000	-0.590185000
F	0.428371000	1.836901000	1.367506000

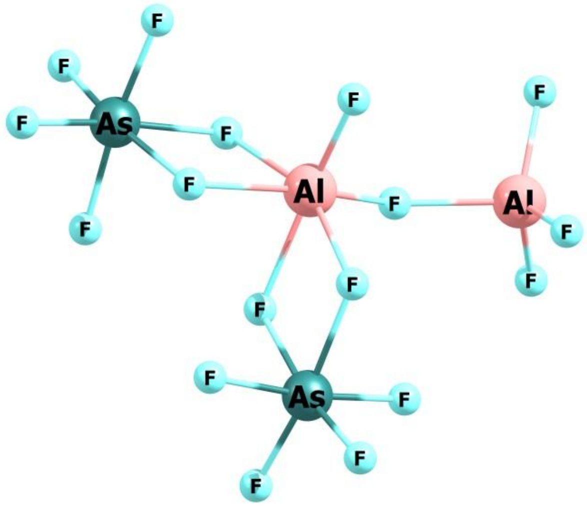

4.3

Al	2.842450000	2.427127000	0.217951000
Al	-0.005961000	0.606714000	-0.522742000
As	-2.926833000	0.354731000	0.151151000
As	1.359904000	-2.027445000	0.024356000
F	0.247170000	-0.801580000	0.850331000
F	-0.068044000	-2.802343000	-0.570194000
F	3.642277000	2.101509000	1.676830000
F	1.249759000	1.513116000	0.397854000
F	1.407499000	-3.032492000	1.420620000
F	2.686595000	-1.068963000	0.566284000
F	1.094302000	-0.742131000	-1.308967000
F	-0.156168000	1.572172000	-1.897175000
F	-4.170191000	-0.661144000	-0.471802000
F	2.370788000	4.047133000	0.051422000
F	2.366637000	-2.967271000	-1.008207000
F	-1.378141000	1.293580000	0.629682000
F	-3.153482000	1.524808000	-1.096267000
F	-3.873819000	1.270438000	1.262497000
F	-1.643438000	-0.431825000	-0.919279000

$Al_2As_2F_{17}^-$	F	-2.507116000	-0.791620000	1.374478000	
	F	3.533622000	1.727685000	-1.161380000	
	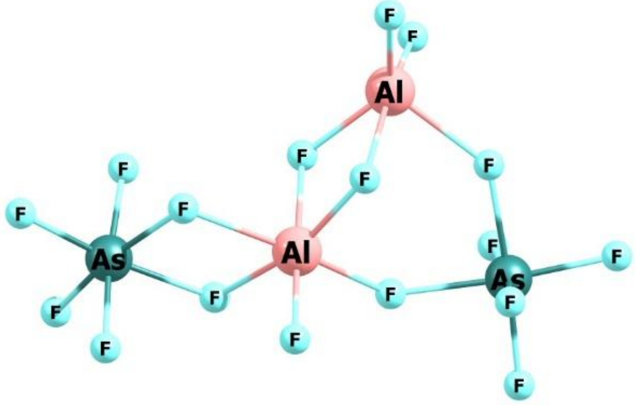				**5.1**
	Al	-0.437633000	-0.162610000	-0.673909000	
	Al	1.141667000	2.207475000	-0.046500000	
	As	-3.308529000	-0.269696000	0.211599000	
	As	2.917235000	-0.829242000	0.167337000	
	F	1.632976000	2.859602000	1.450006000	
	F	-3.489441000	-1.515150000	-0.969445000	
	F	-2.211580000	0.618665000	-0.992732000	
	F	2.797849000	-0.975731000	-1.545774000	
	F	2.645734000	0.992724000	-0.072857000	
	F	2.818769000	-0.518897000	1.861178000	
	F	-4.077842000	-1.226297000	1.424039000	
	F	-2.960007000	0.986541000	1.345797000	
	F	4.616696000	-0.534473000	0.116357000	
	F	1.088292000	3.527514000	-1.112368000	
	F	-1.648110000	-0.996310000	0.620302000	
	F	-4.704251000	0.563745000	-0.367048000	
	F	-0.595552000	-1.382957000	-1.837355000	
	F	0.592570000	1.040145000	-1.522921000	
	F	-0.211698000	1.169180000	0.634117000	
	F	1.071643000	-0.999377000	0.220536000	
	F	3.051759000	-2.533178000	0.399327000	
	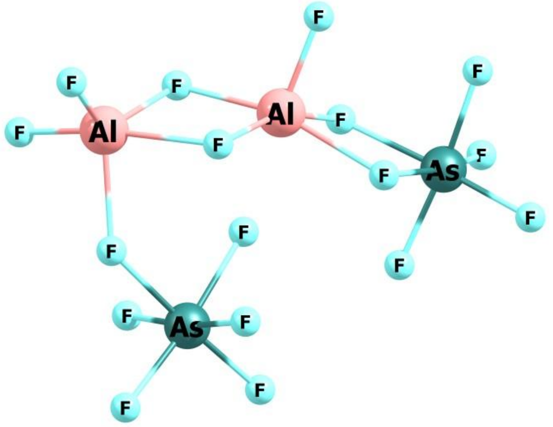				**6.5**
	Al	0.400792000	1.426650000	-0.132618000	
	Al	-2.443122000	1.833649000	0.124448000	
	As	2.910378000	-0.199865000	0.070470000	
	As	-1.781600000	-1.547541000	-0.070901000	
	F	1.571848000	0.258070000	-1.146271000	
	F	1.714355000	0.762428000	1.135737000	

$Al_2As_2F_{17}^-$	F	-0.850229000	2.003313000	1.079816000	
	F	-3.176829000	-2.457465000	-0.532885000	
	F	-1.303758000	-1.320230000	-1.716501000	
	F	-3.115468000	2.982856000	-0.923941000	
	F	3.670199000	1.301424000	-0.307075000	
	F	3.809721000	-1.025909000	-1.146120000	
	F	-2.248744000	-1.540268000	1.592790000	
	F	-2.819220000	-0.002430000	-0.251220000	
	F	-0.429239000	-0.422710000	0.410975000	
	F	-1.027680000	1.458700000	-1.204333000	
	F	1.217939000	2.869387000	-0.481687000	
	F	3.966826000	-0.462095000	1.407851000	
	F	-0.750525000	-2.914147000	0.132599000	
	F	-3.416152000	1.821006000	1.524148000	
	F	1.998135000	-1.614094000	0.439496000	
	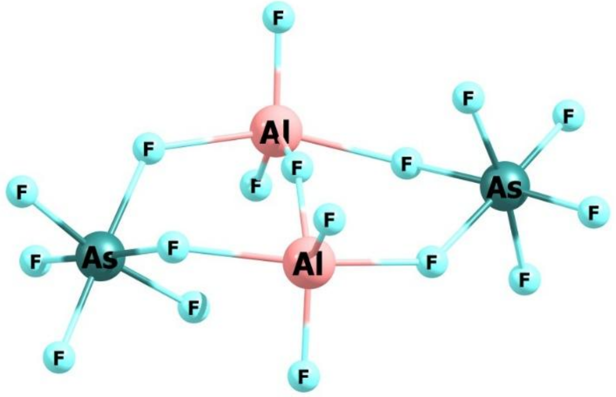				**9.4**
	Al	-0.070291000	-1.727849000	0.312293000	
	Al	-0.070856000	1.728253000	0.312280000	
	As	-3.005479000	-0.000149000	-0.146307000	
	As	3.061795000	-0.000012000	-0.167139000	
	F	2.703869000	0.000061000	1.524373000	
	F	-0.429124000	-2.497781000	-1.141022000	
	F	0.056404000	0.000353000	0.821057000	
	F	-0.428521000	2.497563000	-1.141635000	
	F	-3.893158000	1.258832000	-0.923386000	
	F	4.225695000	1.268239000	-0.009825000	
	F	-1.744602000	-0.000281000	-1.346656000	
	F	1.754560000	1.292394000	-0.366961000	
	F	3.166239000	-0.000330000	-1.891840000	
	F	4.225038000	-1.268854000	-0.009623000	
	F	1.753952000	-1.292294000	-0.366794000	
	F	0.256702000	-2.653303000	1.678392000	
	F	0.256202000	2.654261000	1.677971000	
	F	-1.998303000	1.292662000	0.705790000	
	F	-4.016637000	0.000218000	1.253429000	
	F	-3.892915000	-1.259718000	-0.922676000	
	F	-1.998016000	-1.292015000	0.706548000	

$Al_2PAsF_{17}^-$

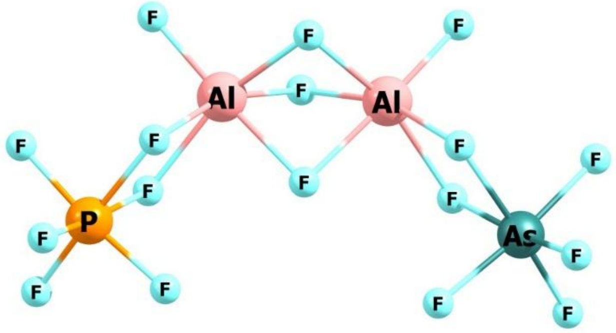

0.0

Al	0.948143000	1.367402000	0.001108000
Al	-1.626720000	1.256989000	-0.001093000
P	-3.577496000	-0.925952000	0.000101000
As	3.131938000	-0.694818000	-0.000093000
F	-2.663500000	0.052730000	-1.143601000
F	-0.364359000	1.915275000	-1.221234000
F	-0.275751000	-0.058756000	-0.000068000
F	-2.664292000	0.054666000	1.142720000
F	4.232545000	0.636826000	0.001478000
F	-2.771572000	2.507062000	-0.003024000
F	2.073066000	0.271382000	-1.176684000
F	-2.338251000	-1.965829000	0.001403000
F	1.908199000	-1.912615000	-0.001576000
F	-4.306851000	-1.707470000	-1.212290000
F	3.970432000	-1.455583000	-1.303976000
F	-4.307681000	-1.705408000	1.213316000
F	3.969598000	-1.457944000	1.302946000
F	-4.701447000	0.239236000	-0.001282000
F	2.072316000	0.269246000	1.177627000
F	-0.366636000	1.915063000	1.221300000
F	1.993075000	2.702252000	0.003095000

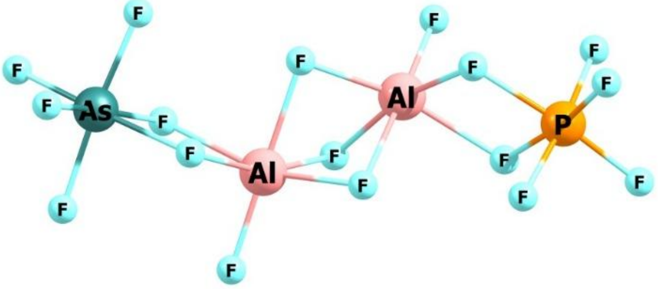

0.3

Al	1.462675000	0.434910000	0.756977000
Al	-0.797451000	-0.793916000	0.487955000
As	-3.514571000	0.214503000	-0.305937000
P	4.081712000	-0.028729000	-0.471299000
F	-1.318124000	-2.382330000	0.773648000
F	0.824663000	-1.018817000	-0.342395000
F	0.357321000	-0.510916000	1.934008000
F	-2.429686000	0.043263000	1.188375000
F	3.422891000	-1.222363000	-1.344234000
F	-1.977911000	-0.505142000	-1.053714000
F	3.124816000	-0.592540000	0.894773000
F	-4.057923000	-1.399471000	-0.019486000
F	-2.823887000	1.778857000	-0.550902000
F	-4.298444000	0.262367000	-1.843935000

$Al_2PAsF_{17}^-$

F	5.320346000	-0.966081000	-0.024066000
F	4.590327000	1.187225000	0.467466000
F	-4.799341000	0.872496000	0.641826000
F	4.776591000	0.617538000	-1.779960000
F	1.984298000	1.709175000	1.746613000
F	-0.185678000	1.008707000	0.179851000
F	2.612770000	0.897967000	-0.758837000

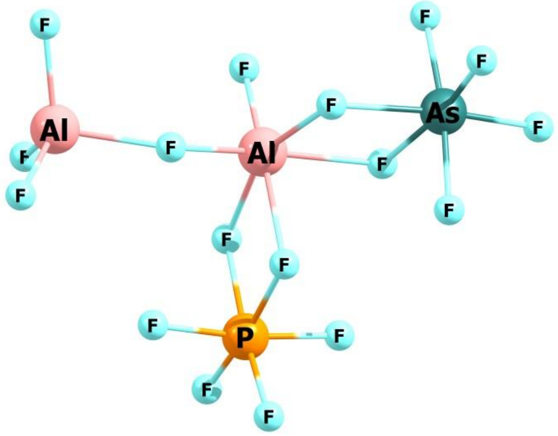

3.8

Al	-3.162686000	-1.951640000	0.220381000
Al	-0.157075000	-0.402301000	-0.530921000
As	2.773352000	-0.461312000	0.150449000
P	-1.215825000	2.278074000	0.035168000
F	-0.278379000	1.026147000	0.831233000
F	0.187716000	2.872267000	-0.519645000
F	-3.914664000	-1.548767000	1.685529000
F	-1.487078000	-1.199350000	0.386177000
F	-1.168413000	3.210012000	1.347081000
F	-2.542386000	1.503890000	0.538335000
F	-1.113290000	1.073891000	-1.268834000
F	-0.113933000	-1.348977000	-1.925410000
F	4.120369000	0.420411000	-0.461177000
F	-2.853485000	-3.610243000	0.051088000
F	-2.069540000	3.265409000	-0.908213000
F	1.132015000	-1.234868000	0.617673000
F	2.882562000	-1.644800000	-1.100512000
F	3.612963000	-1.475826000	1.262382000
F	1.585415000	0.457937000	-0.921161000
F	2.468531000	0.717176000	1.377345000
F	-3.795775000	-1.189484000	-1.153594000

$Al_2PAsF_{17}^-$

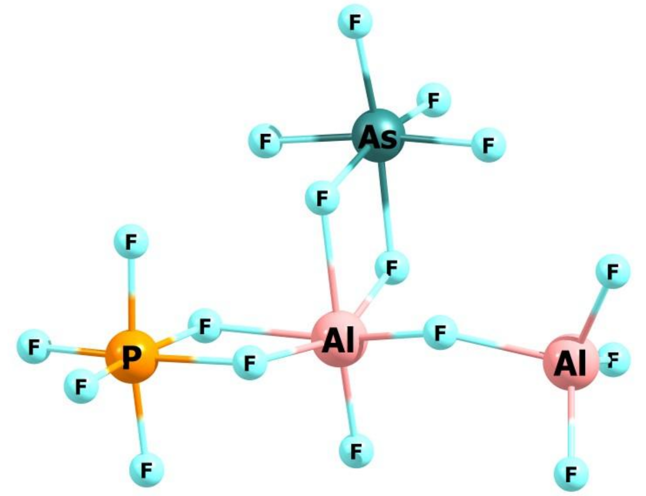

4.0

Al	2.704966000	2.331149000	0.206384000
Al	-0.228853000	0.639076000	-0.506770000
P	-3.072971000	0.553236000	0.191412000
As	1.003436000	-2.064625000	0.025809000
F	-0.017556000	-0.776989000	0.870885000
F	-0.479450000	-2.760846000	-0.530914000
F	3.499914000	1.973903000	1.660534000
F	1.075449000	1.487203000	0.401015000
F	1.030626000	-3.067132000	1.424532000
F	2.394939000	-1.179528000	0.529503000
F	0.776998000	-0.771850000	-1.307870000
F	-0.335265000	1.600440000	-1.887945000
F	-4.302858000	-0.325727000	-0.366389000
F	2.302897000	3.969777000	0.039369000
F	1.930861000	-3.062474000	-1.026780000
F	-1.564088000	1.381939000	0.647697000
F	-3.228418000	1.651364000	-0.985018000
F	-3.907044000	1.463035000	1.229622000
F	-1.938165000	-0.290264000	-0.843407000
F	-2.728596000	-0.534517000	1.341350000
F	3.355499000	1.599572000	-1.175947000

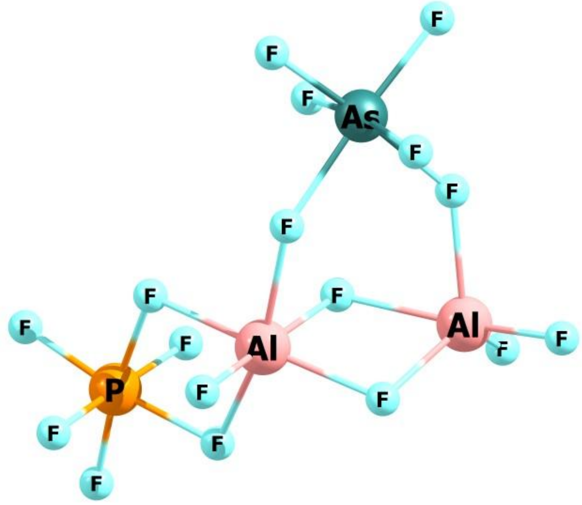

4.2

Al	1.015860000	2.244717000	-0.185885000
Al	-0.758560000	0.220613000	0.893376000
P	-3.353261000	-0.420713000	-0.290697000

$Al_2PAsF_{17}^-$

As	2.436693000	-0.996467000	-0.156675000
F	1.430494000	-1.317735000	-1.520903000
F	2.241990000	3.106688000	0.626531000
F	-1.109034000	-0.338034000	2.453340000
F	-4.717159000	0.437003000	-0.400852000
F	3.880171000	-1.030113000	-1.102778000
F	-3.750444000	-0.974737000	1.177214000
F	0.887023000	-0.825581000	0.843162000
F	-2.794583000	0.174610000	-1.687530000
F	3.272504000	-0.494940000	1.268036000
F	2.499672000	-2.662014000	0.293350000
F	-0.196934000	0.851342000	-0.719033000
F	-2.542612000	0.920672000	0.518667000
F	-3.893478000	-1.762285000	-1.008357000
F	-1.766276000	-1.151146000	-0.054386000
F	0.487070000	3.248962000	-1.445309000
F	2.276126000	0.804846000	-0.558696000
F	0.078041000	1.806328000	1.354581000

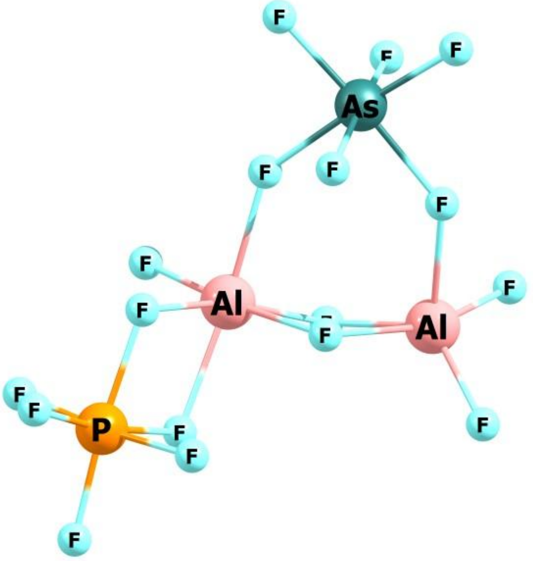

4.2

Al	1.012597000	2.245384000	0.184271000
Al	-0.758239000	0.216644000	-0.893239000
As	2.438738000	-0.994842000	0.157317000
P	-3.354050000	-0.420965000	0.290685000
F	2.503272000	-2.661801000	-0.287277000
F	-2.795237000	0.175603000	1.686840000
F	-0.197050000	0.849536000	0.718841000
F	-3.750904000	-0.976437000	-1.176757000
F	0.076814000	1.803426000	-1.355681000
F	0.481547000	3.247640000	1.444406000
F	3.271263000	-0.496851000	-1.270580000
F	-4.717232000	0.437992000	0.399298000
F	-3.895472000	-1.761236000	1.009778000
F	1.435536000	-1.313052000	1.524415000
F	-1.106763000	-0.343404000	-2.453085000
F	2.276406000	0.807492000	0.553517000
F	2.236647000	3.111255000	-0.627049000
F	0.887012000	-0.829695000	-0.840474000
F	-2.541985000	0.918951000	-0.520083000

$Al_2PAsF_{17}^-$

F	3.884012000	-1.023375000	1.100850000
F	-1.767227000	-1.152941000	0.055798000

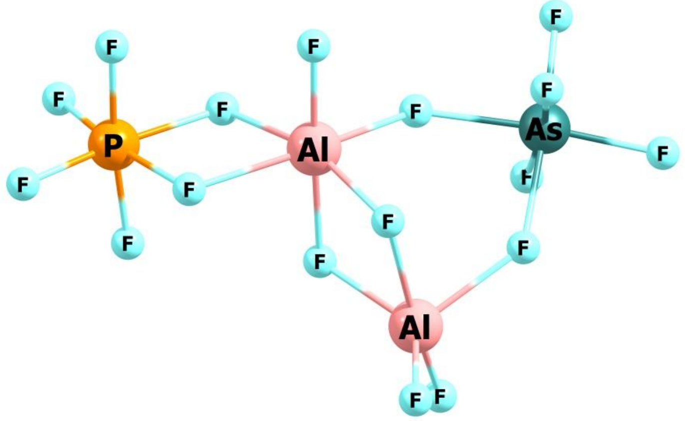

5.1

Al	-0.827669000	2.202794000	-0.044434000
Al	0.726522000	-0.193665000	-0.640239000
P	3.509609000	-0.349864000	0.264598000
As	-2.647148000	-0.808928000	0.155678000
F	4.844760000	0.397488000	-0.252557000
F	3.662611000	-1.518591000	-0.842680000
F	4.207371000	-1.256630000	1.404377000
F	3.189542000	0.833269000	1.323286000
F	-1.337364000	2.866793000	1.441012000
F	0.493675000	1.144240000	0.662663000
F	-2.810598000	-2.508739000	0.399257000
F	-4.341204000	-0.490006000	0.076291000
F	-2.570036000	-0.485747000	1.848174000
F	-2.345600000	1.006883000	-0.095872000
F	0.866237000	-1.417134000	-1.802697000
F	-2.503715000	-0.970736000	-1.554129000
F	-0.739525000	3.520124000	-1.111687000
F	-0.264248000	1.027543000	-1.510001000
F	2.527951000	0.531185000	-0.902983000
F	-0.805318000	-1.006167000	0.238618000
F	1.928425000	-1.026675000	0.656083000

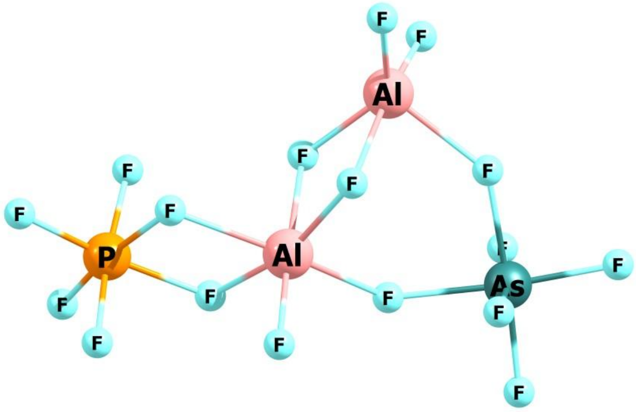

5.1

Al	-0.726513000	-0.193828000	-0.640046000
Al	0.827551000	2.202813000	-0.044446000
P	-3.509665000	-0.349875000	0.264536000
As	2.647271000	-0.808853000	0.155686000
F	1.336892000	2.866991000	1.440985000
F	-3.662449000	-1.518910000	-0.842460000

$Al_2PAsF_{17}^-$	F	-2.527952000	0.530940000	-0.903115000
	F	2.503872000	-0.970620000	-1.554114000
	F	2.345564000	1.006958000	-0.095801000
	F	2.570173000	-0.485722000	1.848201000
	F	-4.207476000	-1.256424000	1.404449000
	F	-3.189808000	0.833477000	1.323029000
	F	4.341296000	-0.489840000	0.076305000
	F	0.739452000	3.519958000	-1.111967000
	F	-1.928472000	-1.026513000	0.656334000
	F	-4.844816000	0.397267000	-0.252933000
	F	-0.866040000	-1.417349000	-1.802516000
	F	0.264180000	1.027413000	-1.509964000
	F	-0.493801000	1.144096000	0.662697000
	F	0.805456000	-1.006012000	0.238618000
	F	2.810764000	-2.508659000	0.399220000
	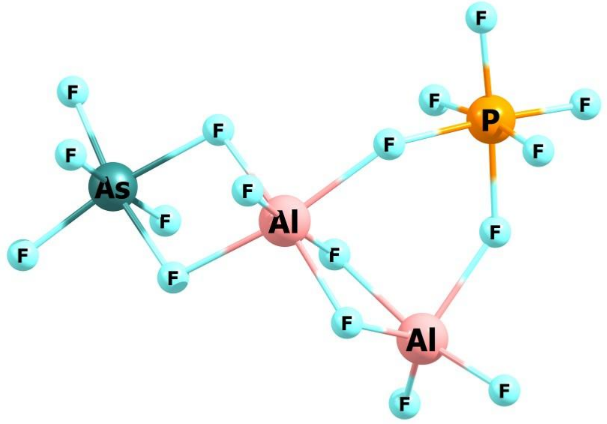			**5.4**
	Al	1.715134000	2.043292000	-0.175095000
	Al	-0.216907000	0.188362000	0.908016000
	As	-2.963928000	-0.219046000	-0.223987000
	P	2.765447000	-1.261790000	-0.220809000
	F	1.753711000	-1.447033000	-1.468849000
	F	3.053449000	2.745095000	0.613861000
	F	-0.570991000	-0.392509000	2.458831000
	F	-4.339409000	0.822371000	-0.258144000
	F	4.073445000	-1.421543000	-1.156231000
	F	-3.388557000	-0.825259000	1.336269000
	F	1.353300000	-0.963514000	0.781811000
	F	-2.372795000	0.413405000	-1.717798000
	F	3.644673000	-0.898448000	1.087313000
	F	2.678133000	-2.825338000	0.179018000
	F	0.324158000	0.823595000	-0.705979000
	F	-1.944080000	1.071962000	0.638600000
	F	-3.669674000	-1.582648000	-1.010486000
	F	-1.338358000	-1.097272000	-0.041489000
	F	1.284558000	3.124258000	-1.408709000
	F	2.775024000	0.459883000	-0.580641000
	F	0.777966000	1.675646000	1.383262000

$Al_2PAsF_{17}^-$

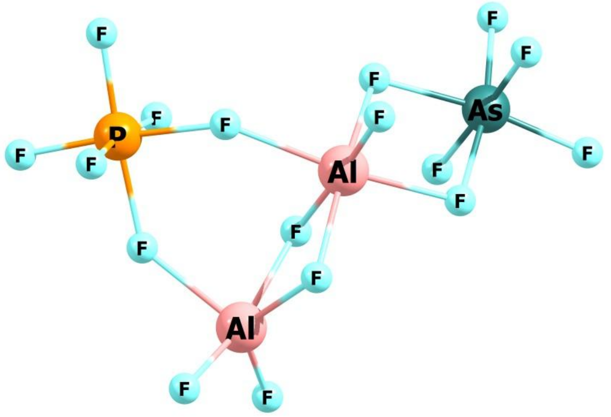

5.4

Al	1.708618000	2.046075000	0.171528000
Al	-0.216509000	0.180194000	-0.906343000
P	2.770953000	-1.258004000	0.222634000
As	-2.964945000	-0.219572000	0.223727000
F	2.687885000	-2.824374000	-0.166849000
F	-2.375118000	0.418001000	1.715795000
F	0.323629000	0.820722000	0.705829000
F	-3.387963000	-0.831307000	-1.334803000
F	0.776092000	1.668220000	-1.386019000
F	1.270759000	3.127054000	1.402777000
F	3.643173000	-0.899668000	-1.091579000
F	-4.339362000	0.823356000	0.251972000
F	-3.672816000	-1.579609000	1.014410000
F	1.765597000	-1.439216000	1.476399000
F	-0.568285000	-0.406470000	-2.455536000
F	2.775414000	0.465830000	0.571265000
F	3.044077000	2.755376000	-0.615604000
F	1.353398000	-0.971511000	-0.775351000
F	-1.942336000	1.066849000	-0.642597000
F	4.083657000	-1.406929000	1.153221000
F	-1.339860000	-1.100275000	0.046680000

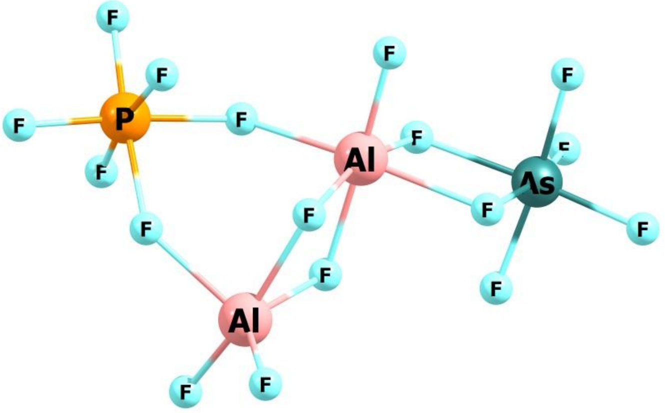

6.2

Al	-0.194151000	-0.208140000	-0.662339000
Al	1.517551000	2.056384000	-0.042633000
As	-3.072697000	-0.189527000	0.203538000
P	3.035047000	-1.037569000	0.214593000
F	2.039200000	2.681892000	1.455023000
F	-3.293917000	-1.448955000	-0.955572000
F	-1.933869000	0.633449000	-1.008886000
F	2.911871000	-1.181257000	-1.389509000

Species	Atom	x	y	z	
	F	2.901850000	0.705249000	-0.024414000	
	F	2.976044000	-0.733631000	1.801085000	
	F	-3.887834000	-1.092235000	1.427363000	
	F	-2.684557000	1.074113000	1.316552000	
	F	4.641609000	-0.878567000	0.159507000	
	F	1.600276000	3.366585000	-1.119131000	
	F	-1.445218000	-0.970982000	0.639034000	
	F	-4.430808000	0.686333000	-0.401896000	
	F	-0.392659000	-1.446616000	-1.799210000	
	F	0.894454000	0.932387000	-1.521657000	
	F	0.073592000	1.138785000	0.623243000	
	F	1.276679000	-1.075800000	0.271638000	
	F	3.049855000	-2.636223000	0.441160000	
$Al_2PAsF_{17}^-$	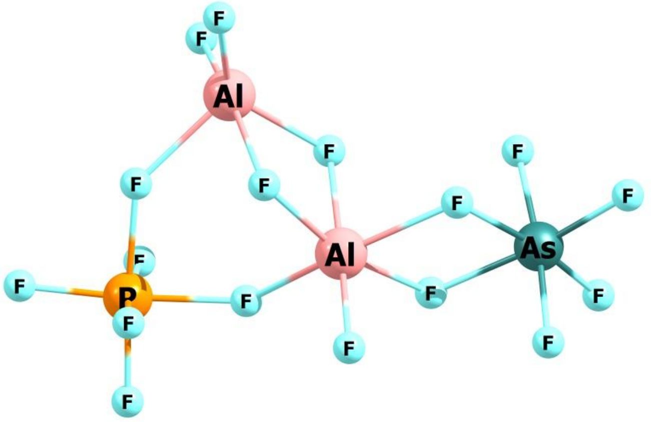				**6.2**
	Al	-1.517505000	2.056394000	-0.042650000	
	Al	0.194133000	-0.208201000	-0.662300000	
	As	3.072693000	-0.189518000	0.203524000	
	P	-3.035077000	-1.037532000	0.214593000	
	F	4.430813000	0.686272000	-0.401995000	
	F	3.293899000	-1.449056000	-0.955471000	
	F	3.887851000	-1.092110000	1.427424000	
	F	2.684572000	1.074228000	1.316422000	
	F	-2.039050000	2.682041000	1.454987000	
	F	-0.073540000	1.138818000	0.623206000	
	F	-3.049898000	-2.636199000	0.441025000	
	F	-4.641636000	-0.878534000	0.159435000	
	F	-2.976175000	-0.733766000	1.801122000	
	F	-2.901846000	0.705313000	-0.024241000	
	F	0.392696000	-1.446792000	-1.799037000	
	F	-2.911805000	-1.181102000	-1.389513000	
	F	-1.600309000	3.366484000	-1.119276000	
	F	-0.894481000	0.932271000	-1.521647000	
	F	1.933855000	0.633345000	-1.008958000	
	F	-1.276706000	-1.075771000	0.271751000	
	F	1.445222000	-0.970933000	0.639119000	

$Al_2PAsF_{17}^{-}$

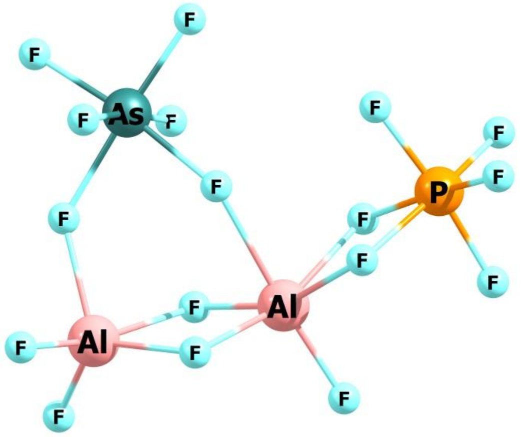

6.6

Al	-0.194151000	-0.208140000	-0.662339000
Al	1.517551000	2.056384000	-0.042633000
As	-3.072697000	-0.189527000	0.203538000
P	3.035047000	-1.037569000	0.214593000
F	2.039200000	2.681892000	1.455023000
F	-3.293917000	-1.448955000	-0.955572000
F	-1.933869000	0.633449000	-1.008886000
F	2.911871000	-1.181257000	-1.389509000
F	2.901850000	0.705249000	-0.024414000
F	2.976044000	-0.733631000	1.801085000
F	-3.887834000	-1.092235000	1.427363000
F	-2.684557000	1.074113000	1.316552000
F	4.641609000	-0.878567000	0.159507000
F	1.600276000	3.366585000	-1.119131000
F	-1.445218000	-0.970982000	0.639034000
F	-4.430808000	0.686333000	-0.401896000
F	-0.392659000	-1.446616000	-1.799210000
F	0.894454000	0.932387000	-1.521657000
F	0.073592000	1.138785000	0.623243000
F	1.276679000	-1.075800000	0.271638000
F	3.049855000	-2.636223000	0.441160000

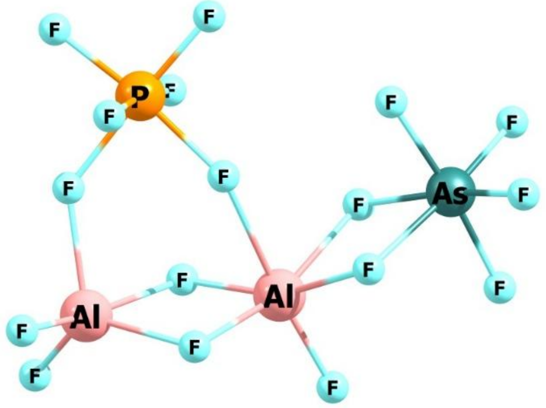

7.3

Al	-0.158084000	-1.348085000	-0.141834000
Al	2.706670000	-1.450617000	0.132463000
As	-2.756522000	0.125218000	0.072579000
P	1.746511000	1.769652000	-0.095494000
F	-1.398433000	-0.257739000	-1.150731000
F	-1.484690000	-0.741684000	1.134425000
F	1.133207000	-1.790997000	1.078318000
F	2.958640000	2.738860000	-0.559117000

Species	Atom	X	Y	Z	Energy
	F	1.299295000	1.504624000	-1.631200000	
	F	3.507570000	-2.536238000	-0.893910000	
	F	-3.413143000	-1.429980000	-0.280475000	
	F	-3.720516000	0.870894000	-1.146082000	
	F	2.217609000	1.823601000	1.453911000	
	F	2.873431000	0.398447000	-0.280741000	
	F	0.581133000	0.602526000	0.387652000	
	F	1.260951000	-1.271718000	-1.214557000	
	F	-0.876340000	-2.846907000	-0.465247000	
	F	-3.816235000	0.327546000	1.417487000	
	F	0.673909000	2.959861000	0.100348000	
	F	3.659421000	-1.318333000	1.539597000	
	F	-1.940703000	1.601255000	0.416897000	
$BAlP_2F_{17}^-$	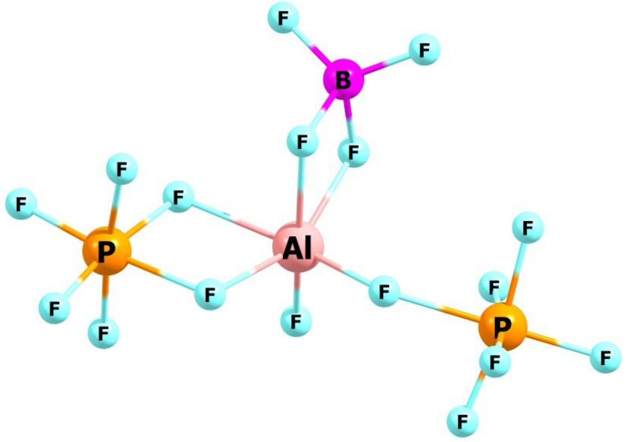				**0.0**
	B	0.029157000	2.571434000	0.047072000	
	Al	0.218476000	0.024738000	-0.484931000	
	P	2.991557000	-0.704687000	0.187977000	
	P	-3.070369000	-0.659527000	0.141884000	
	F	4.426771000	-0.179139000	-0.330697000	
	F	2.855710000	-1.753717000	-1.036931000	
	F	3.539231000	-1.856151000	1.180302000	
	F	2.957788000	0.380504000	1.392203000	
	F	-1.096120000	3.183657000	0.499371000	
	F	0.404469000	1.396490000	0.902293000	
	F	-3.124232000	-1.414626000	1.585954000	
	F	-4.665621000	-0.771072000	-0.122618000	
	F	-3.175283000	0.778251000	0.900801000	
	F	-2.760760000	0.119041000	-1.259410000	
	F	0.091329000	-0.879929000	-1.901117000	
	F	-2.729875000	-2.078606000	-0.577940000	
	F	1.117605000	3.354367000	-0.212199000	
	F	-0.280610000	1.707442000	-1.176175000	
	F	2.151644000	0.460243000	-0.798932000	
	F	-1.230009000	-0.527919000	0.463519000	
	F	1.317539000	-1.109454000	0.616114000	

$BAlP_2F_{17}^-$

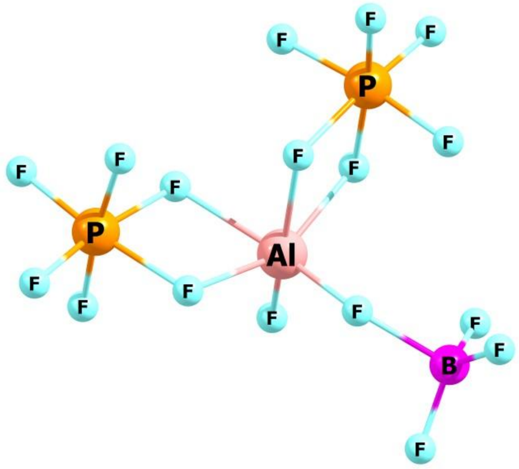

0.9

B	2.589669000	2.325272000	0.233675000
Al	0.026357000	0.604352000	-0.411980000
P	-2.840402000	0.243648000	0.158074000
P	1.358958000	-1.988056000	0.016302000
F	0.281133000	-0.914827000	0.868911000
F	0.044069000	-2.707781000	-0.606618000
F	3.354217000	2.089921000	1.352012000
F	1.175073000	1.485454000	0.620415000
F	1.405721000	-3.011654000	1.261946000
F	2.587643000	-1.111086000	0.594795000
F	1.140407000	-0.726246000	-1.211491000
F	-0.135737000	1.583079000	-1.775718000
F	-3.982461000	-0.693912000	-0.490381000
F	2.132426000	3.613966000	0.088074000
F	2.334633000	-2.811213000	-0.971441000
F	-1.427738000	1.159830000	0.718878000
F	-3.051229000	1.393715000	-0.961444000
F	-3.785225000	1.025337000	1.210333000
F	-1.616258000	-0.446448000	-0.869409000
F	-2.458427000	-0.877263000	1.265881000
F	2.994049000	1.691703000	-0.920108000

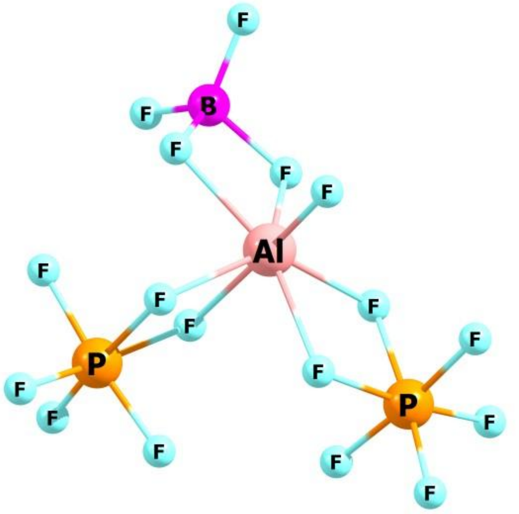

3.6

Al	-0.177127000	0.792658000	-0.453313000
B	-2.197605000	2.330831000	0.332957000
P	2.700970000	0.088437000	0.165253000

	P	-1.416122000	-1.909894000	-0.014356000	
	F	1.459357000	-0.457792000	-0.881765000	
	F	1.415766000	1.176333000	0.659878000	
	F	-0.754181000	2.188721000	0.769630000	
	F	-2.026399000	-3.065405000	-0.964066000	
	F	-0.999487000	-0.912795000	-1.359274000	
	F	-2.408427000	3.568076000	-0.216120000	
	F	3.116655000	1.182648000	-0.961250000	
	F	3.752072000	-0.996999000	-0.414270000	
	F	-1.691276000	-2.624441000	1.410106000	
	F	-2.837457000	-1.140339000	0.068214000	
	F	-0.693731000	-0.494512000	0.840332000	
	F	-2.117667000	1.275289000	-0.699607000	
	F	0.218162000	1.662630000	-1.845667000	
	F	3.709187000	0.764896000	1.240983000	
	F	0.067412000	-2.547152000	-0.079246000	
	F	-3.028326000	1.971120000	1.362410000	
	F	2.153667000	-0.954372000	1.288026000	
**$BAlP_2F_{17}^-$**	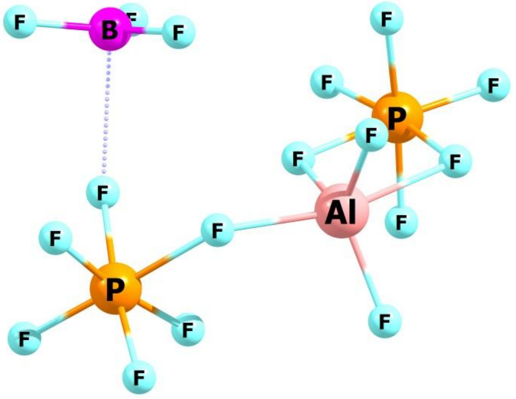				**7.3**
	B	0.997860000	2.674783000	0.501810000	
	Al	-0.518575000	-1.289511000	0.994002000	
	P	-2.696515000	0.128247000	-0.457934000	
	P	2.479364000	-0.619883000	-0.468174000	
	F	1.269847000	-0.812067000	0.877313000	
	F	3.552784000	-0.416953000	-1.665828000	
	F	-2.497202000	-0.966881000	-1.639970000	
	F	3.435353000	0.065182000	0.658013000	
	F	-2.612480000	-1.151910000	0.666287000	
	F	-0.742027000	-0.919896000	2.622310000	
	F	-0.513554000	-2.874768000	0.432591000	
	F	-2.725217000	1.159463000	0.796581000	
	F	2.202993000	3.264511000	0.570461000	
	F	0.568473000	1.924220000	1.523962000	
	F	0.164277000	2.997697000	-0.495797000	
	F	1.782863000	0.857178000	-0.740961000	
	F	3.035595000	-2.094121000	-0.064308000	
	F	-2.502252000	1.344587000	-1.500648000	
	F	-4.307250000	0.052286000	-0.542064000	
	F	1.355470000	-1.291717000	-1.440453000	
	F	-0.911066000	0.059218000	-0.228542000	

$BAlP_2F_{17}^-$

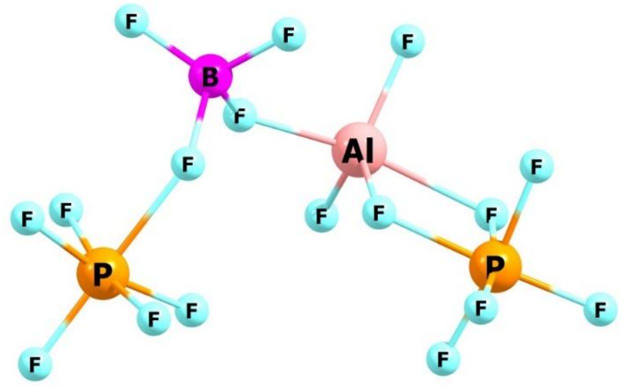

8.6

Al	1.205067000	1.345722000	0.857189000
B	-1.298709000	1.665417000	-0.909164000
P	-2.816922000	-0.896676000	0.178764000
P	2.729300000	-0.916466000	-0.281641000
F	-4.072105000	-0.484406000	-0.768182000
F	1.895185000	2.765282000	0.271339000
F	-0.368572000	1.883232000	-1.891094000
F	-3.054576000	0.452156000	1.048225000
F	2.182272000	-1.791790000	0.968449000
F	-2.347747000	2.531525000	-0.827363000
F	3.027985000	0.362543000	0.825660000
F	-1.768090000	0.257738000	-1.050265000
F	3.143495000	0.102001000	-1.475267000
F	-2.410673000	-2.045149000	-0.893684000
F	4.230440000	-1.488589000	-0.136247000
F	-1.394467000	-1.112272000	0.929015000
F	2.194582000	-2.008405000	-1.340385000
F	-3.663065000	-1.868997000	1.146651000
F	1.116127000	-0.118347000	-0.298736000
F	-0.584882000	1.663648000	0.429354000
F	1.000980000	1.052680000	2.500921000

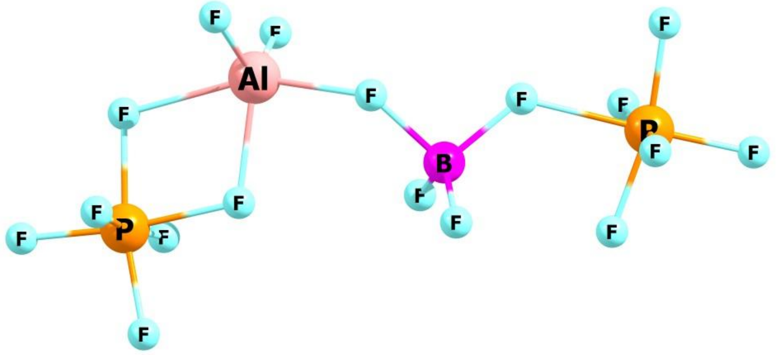

8.9

B	-1.080629000	0.189454000	-0.398747000
Al	1.547751000	-1.366410000	0.041405000
P	3.744549000	0.627341000	0.059233000
P	-4.153925000	0.185786000	0.109657000
F	1.998424000	-2.529750000	-1.090331000
F	1.166205000	-1.731299000	1.636776000
F	-0.135600000	-0.995309000	-0.654921000
F	2.014109000	0.390682000	-0.395350000
F	-3.815481000	0.019306000	1.687690000
F	3.542264000	-1.001701000	0.545665000
F	-2.339685000	-0.568092000	-0.160403000

F	4.110051000	0.079672000	-1.425049000
F	3.204575000	1.073044000	1.521360000
F	5.288581000	0.655172000	0.521979000
F	-4.584871000	-1.379288000	0.053908000
F	-4.206511000	0.240160000	-1.512110000
F	3.665574000	2.146548000	-0.479620000
F	-5.644853000	0.760021000	0.330587000
F	-1.148117000	0.907518000	-1.553517000
F	-0.655513000	0.817039000	0.735330000
F	-3.412149000	1.629516000	0.118241000

$BAlAs_2F_{17}^-$

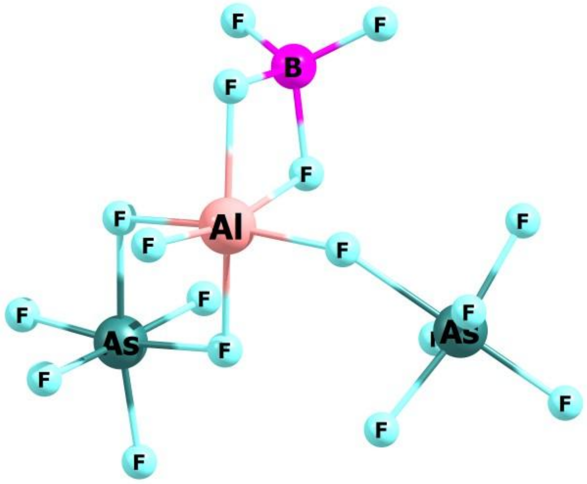

0.0

Al	0.306401000	0.675455000	0.927448000
B	0.109868000	2.919831000	-0.378699000
As	-2.790319000	-0.705066000	-0.133726000
As	2.646663000	-0.831490000	-0.199925000
F	-3.386447000	0.818221000	-0.716764000
F	1.244008000	3.576655000	-0.759039000
F	0.139999000	1.488052000	-0.828742000
F	-1.485976000	0.348052000	0.846744000
F	2.275911000	-0.150829000	-1.743820000
F	-1.086013000	3.506993000	-0.650595000
F	0.805297000	-0.961690000	0.090606000
F	-1.625766000	-0.733239000	-1.424922000
F	-3.774666000	-0.536428000	1.288862000
F	-3.964007000	-1.645717000	-0.991940000
F	2.701324000	-2.424962000	-0.853189000
F	2.863403000	-1.422100000	1.408451000
F	-2.010502000	-2.087148000	0.578296000
F	4.319342000	-0.440785000	-0.354210000
F	0.553197000	0.323337000	2.562534000
F	0.197211000	2.562244000	1.105576000
F	2.256808000	0.815600000	0.53627900

$BAlAs_2F_{17}^-$

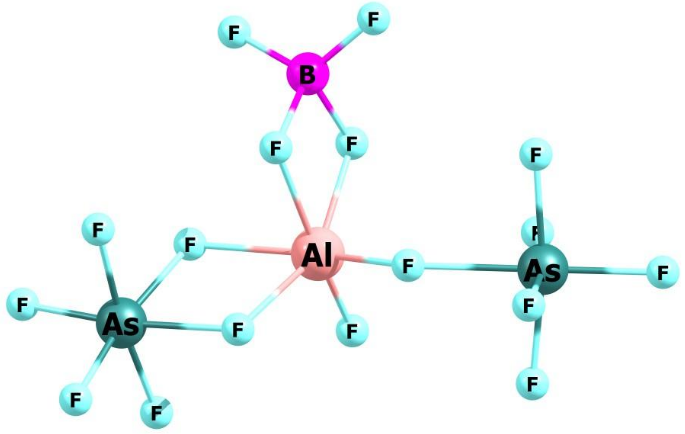

0.6

B	0.050299000	2.706361000	0.054592000
Al	0.233784000	0.168541000	-0.504170000
As	3.074591000	-0.616944000	0.149470000
As	-3.140196000	-0.570706000	0.112878000
F	4.601312000	-0.059509000	-0.425958000
F	2.910850000	-1.735805000	-1.154928000
F	3.645569000	-1.853986000	1.208090000
F	3.062571000	0.540190000	1.434546000
F	-1.068071000	3.297115000	0.548302000
F	0.462923000	1.522698000	0.885689000
F	-3.158101000	-1.364841000	1.660341000
F	-4.837435000	-0.771678000	-0.166671000
F	-3.324262000	0.960994000	0.912102000
F	-2.845553000	0.263117000	-1.384934000
F	0.078610000	-0.741486000	-1.913272000
F	-2.709465000	-2.074185000	-0.640215000
F	1.123656000	3.502134000	-0.223742000
F	-0.285674000	1.853870000	-1.170103000
F	2.155843000	0.608541000	-0.871348000
F	-1.237911000	-0.339366000	0.443878000
F	1.300060000	-1.000065000	0.594197000

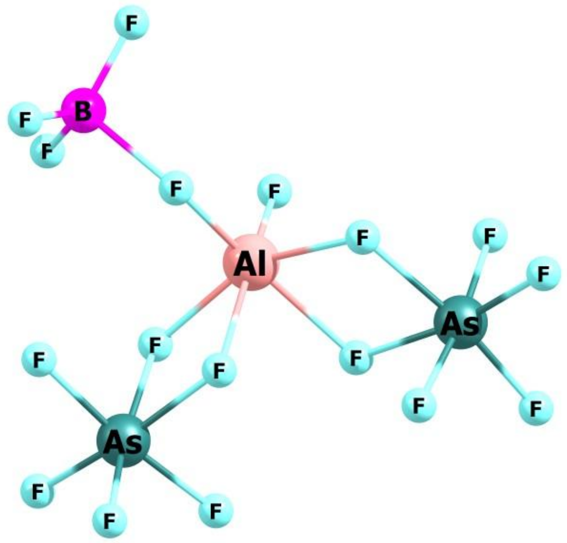

2.1

B	2.357829000	2.835206000	0.241713000
Al	0.047117000	0.784840000	-0.419673000
As	-2.820054000	-0.003398000	0.124914000
As	1.766681000	-1.666991000	0.010657000

	F	0.468101000	-0.691246000	0.876422000	
	F	0.494445000	-2.612966000	-0.684284000	
	F	3.150051000	2.702706000	1.357253000	
	F	1.083059000	1.788005000	0.620296000	
	F	1.940565000	-2.757843000	1.333723000	
	F	2.928032000	-0.570670000	0.660434000	
	F	1.345823000	-0.350511000	-1.246035000	
	F	-0.262545000	1.762616000	-1.758841000	
	F	-3.865527000	-1.168287000	-0.598393000	
	F	1.712983000	4.042247000	0.110840000	
	F	2.930964000	-2.370577000	-1.049265000	
	F	-1.471306000	1.143827000	0.716649000	
	F	-3.213439000	1.188694000	-1.060777000	
	F	-3.942239000	0.672339000	1.248906000	
	F	-1.402368000	-0.501604000	-0.934684000	
	F	-2.260835000	-1.141185000	1.301584000	
	F	2.848640000	2.280442000	-0.919015000	
**$BAlAs_2F_{17}^-$**	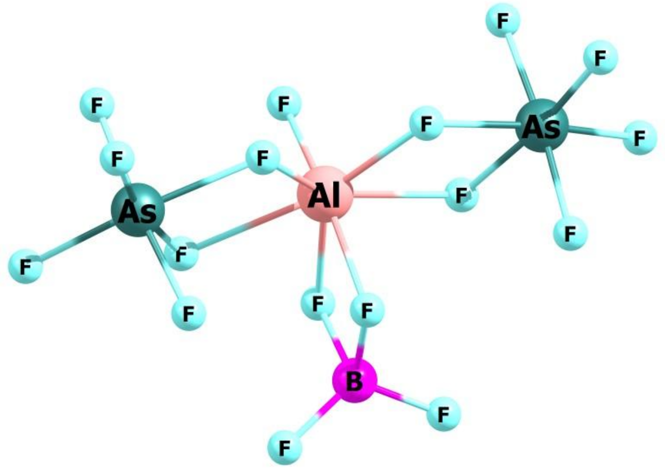				**2.8**
	B	-0.000855000	2.636342000	-0.016757000	
	Al	0.000490000	0.087073000	0.583746000	
	As	2.955157000	-0.563849000	-0.158271000	
	As	-2.954830000	-0.564109000	-0.158189000	
	F	1.197343000	-1.075550000	-0.461787000	
	F	-0.000307000	1.307308000	-0.822750000	
	F	1.163747000	3.302275000	-0.251097000	
	F	2.052945000	0.630241000	0.857276000	
	F	-1.196487000	-1.074817000	-0.461477000	
	F	-0.000130000	-0.742313000	2.048752000	
	F	-2.789685000	0.524572000	-1.498907000	
	F	2.789866000	0.524435000	-1.499244000	
	F	2.971485000	-1.645319000	1.195039000	
	F	3.529616000	-1.815209000	-1.206665000	
	F	-3.527964000	-1.815497000	-1.207405000	
	F	-4.497217000	0.090519000	0.264863000	
	F	4.496778000	0.092207000	0.265338000	
	F	-2.971009000	-1.646498000	1.194421000	
	F	-1.166147000	3.301045000	-0.250811000	
	F	-0.000235000	1.958183000	1.302950000	
	F	-2.054029000	0.629857000	0.857975000	

$BAlAs_2F_{17}^-$

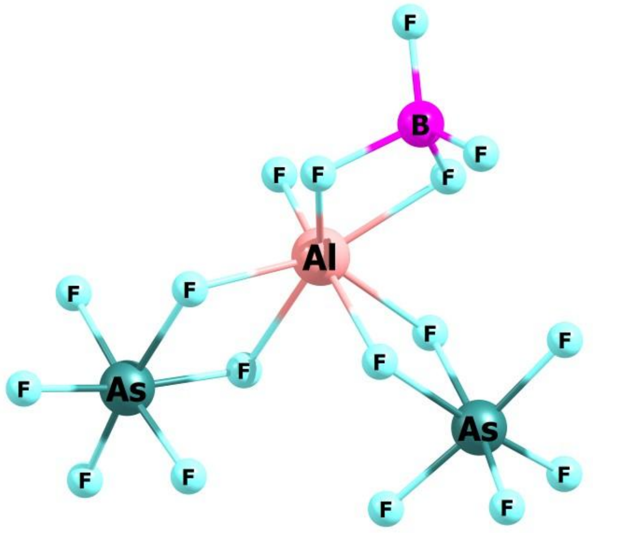

4.8

Al	0.145886000	1.006915000	0.467051000
B	1.936638000	2.791761000	-0.351425000
As	-2.673425000	-0.150783000	-0.131371000
As	1.800890000	-1.563806000	0.015326000
F	-1.245752000	-0.487684000	0.937906000
F	-1.500722000	1.182892000	-0.641176000
F	0.515356000	2.474478000	-0.759407000
F	2.647233000	-2.681103000	1.022870000
F	1.230123000	-0.557209000	1.418770000
F	2.007019000	4.041798000	0.203670000
F	-3.281531000	0.923004000	1.084222000
F	-3.575635000	-1.479578000	0.506156000
F	2.157028000	-2.273138000	-1.519098000
F	3.186045000	-0.540365000	-0.116173000
F	0.806934000	-0.192962000	-0.841162000
F	2.006694000	1.728459000	0.678625000
F	-0.361054000	1.827983000	1.852597000
F	-3.862108000	0.404249000	-1.260953000
F	0.340250000	-2.476292000	0.120885000
F	2.786148000	2.538364000	-1.396459000
F	-1.943370000	-1.151479000	-1.345171000

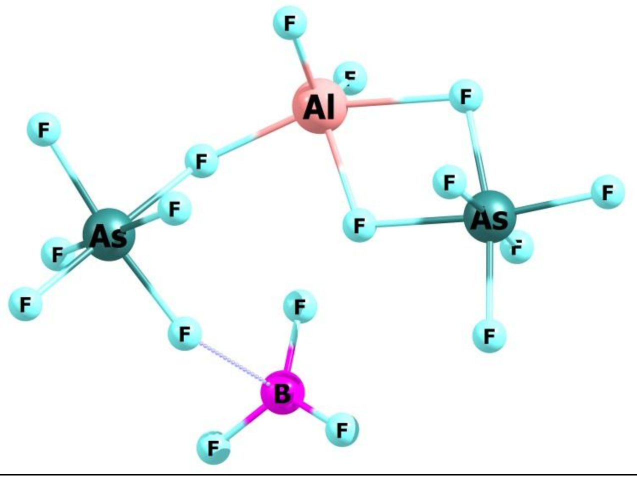

8.1

B	-0.782310000	2.777844000	-0.548879000
Al	0.431890000	-1.277848000	-1.127890000
As	-2.598489000	-0.448765000	0.403636000

As	2.734743000	0.015599000	0.404110000
F	2.816973000	1.157340000	-0.895160000
F	-3.281014000	-1.986242000	-0.022402000
F	0.105603000	2.991658000	0.431253000
F	-1.440882000	-1.222988000	1.442342000
F	2.589709000	1.283321000	1.561981000
F	2.468478000	-1.183192000	1.623965000
F	-3.734242000	-0.159967000	1.677912000
F	-1.340775000	-0.728360000	-0.994560000
F	-0.469667000	1.983694000	-1.581261000
F	-1.772946000	1.081421000	0.688370000
F	0.861372000	0.018372000	0.142870000
F	-1.903830000	3.516554000	-0.595281000
F	-3.566692000	0.333914000	-0.806726000
F	0.664141000	-0.870546000	-2.745398000
F	2.533165000	-1.287436000	-0.832990000
F	0.333014000	-2.869139000	-0.592993000
F	4.448770000	-0.167594000	0.470448000

$BAlPAsF_{17}^-$

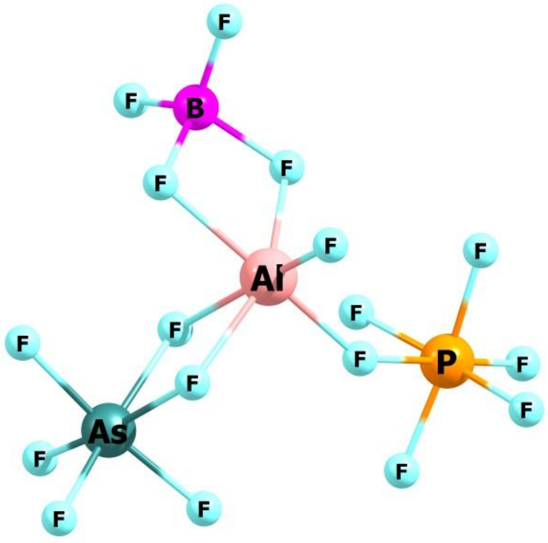

0.0

As	-2.095459000	1.108423000	-0.090172000
P	2.833859000	0.801394000	-0.171926000
Al	-0.011129000	-0.887685000	0.843205000
B	-0.400850000	-3.044476000	-0.529871000
F	4.205142000	1.143533000	-0.965634000
F	-2.543227000	1.697675000	-1.645220000
F	0.783413000	-2.094569000	-0.357413000
F	-0.854418000	-2.991893000	-1.814346000
F	0.349326000	-1.528723000	2.367457000
F	-3.201967000	-0.218179000	-0.199178000
F	-1.408307000	0.286974000	1.444734000
F	-1.303475000	-2.274968000	0.391958000
F	-3.218445000	2.017850000	0.854663000
F	-0.099896000	-4.270396000	-0.015554000
F	1.254139000	0.408500000	0.755125000
F	2.176088000	2.269044000	-0.415065000
F	-0.874532000	2.310448000	0.105054000
F	-0.805027000	-0.001915000	-0.781103000
F	3.397297000	1.301232000	1.272513000
F	3.283006000	-0.724818000	0.191319000

$BAlPAsF_{17}^{-}$	F	2.059906000	0.243916000	-1.495727000	
	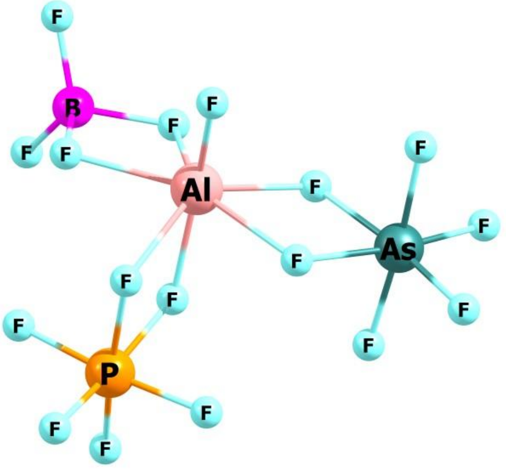				**4.8**
	As	-2.534352000	-0.066976000	0.128149000	
	P	1.667928000	1.910407000	-0.018156000	
	Al	0.430807000	-0.800802000	-0.457333000	
	B	2.456301000	-2.325558000	0.343807000	
	F	-1.165300000	-1.193998000	0.656283000	
	F	0.187908000	2.552185000	-0.117069000	
	F	0.040903000	-1.685233000	-1.841996000	
	F	2.673482000	-3.564350000	-0.199155000	
	F	-2.974090000	-1.247666000	-1.060874000	
	F	0.918993000	0.498259000	0.828622000	
	F	1.279737000	0.911292000	-1.365773000	
	F	1.012090000	-2.186767000	0.776626000	
	F	1.911452000	2.627305000	1.410936000	
	F	-1.184750000	0.467097000	-0.955355000	
	F	-3.639041000	1.091125000	-0.524437000	
	F	2.304416000	3.061643000	-0.955327000	
	F	2.373215000	-1.275293000	-0.694948000	
	F	3.284150000	-1.957034000	1.372227000	
	F	3.083376000	1.135220000	0.102587000	
	F	-1.965205000	1.056877000	1.320200000	
	F	-3.615478000	-0.780404000	1.277423000	
	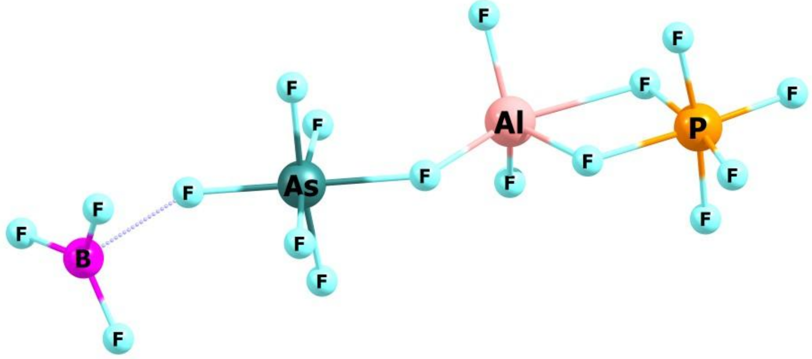				**8.8**
	Al	1.516947000	-0.804544000	0.002652000	
	P	4.229846000	0.492903000	-0.000864000	
	As	-1.847661000	-0.201543000	-0.001153000	
	B	-5.404480000	0.661063000	0.000811000	
	F	4.133138000	0.475277000	-1.622299000	
	F	4.483745000	2.091630000	-0.007391000	
	F	3.677533000	-1.107114000	0.005716000	

**$BAlPAsF_{17}^{-}$**	F	4.131232000	0.489076000	1.620573000	
	F	-0.001385000	0.264487000	-0.005036000	
	F	-1.311877000	-1.856928000	0.006877000	
	F	1.438823000	-1.548927000	1.505052000	
	F	-1.767524000	-0.170649000	1.731410000	
	F	-5.116789000	1.273410000	1.158535000	
	F	-6.187403000	-0.433070000	0.007651000	
	F	-5.122328000	1.262417000	-1.164180000	
	F	-1.770137000	-0.188247000	-1.734046000	
	F	-3.545740000	-0.647216000	0.002311000	
	F	5.782690000	0.054340000	0.002046000	
	F	-2.175518000	1.502838000	-0.009522000	
	F	2.445935000	0.813626000	-0.003157000	
	F	1.441960000	-1.562603000	-1.493154000	
	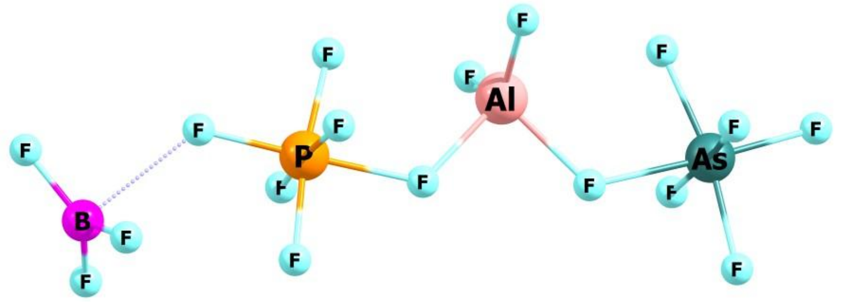				**9.6**
	Al	-0.770717000	0.808682000	-0.001090000	
	As	-3.831252000	-0.339105000	0.000158000	
	P	2.388539000	0.158718000	0.001012000	
	B	5.926364000	-0.576764000	-0.000763000	
	F	-3.715524000	-0.354731000	-1.731412000	
	F	-3.977260000	-2.067408000	0.002849000	
	F	-3.348683000	1.348680000	-0.002431000	
	F	-3.715941000	-0.349304000	1.731809000	
	F	0.634643000	-0.372643000	0.000820000	
	F	1.782400000	1.684477000	-0.001403000	
	F	-0.810242000	1.580171000	1.484887000	
	F	2.302656000	0.140892000	1.623403000	
	F	5.664086000	-1.190580000	1.160568000	
	F	6.634943000	0.565007000	-0.002085000	
	F	5.661527000	-1.191706000	-1.160923000	
	F	2.304425000	0.136414000	-1.621387000	
	F	3.945181000	0.674098000	0.001170000	
	F	-5.527815000	-0.013174000	-0.000542000	
	F	2.776529000	-1.418173000	0.003400000	
	F	-1.913814000	-0.615748000	0.000854000	
	F	-0.809253000	1.574914000	-1.489847000	

BAlPAsF$_{17}^{-}$

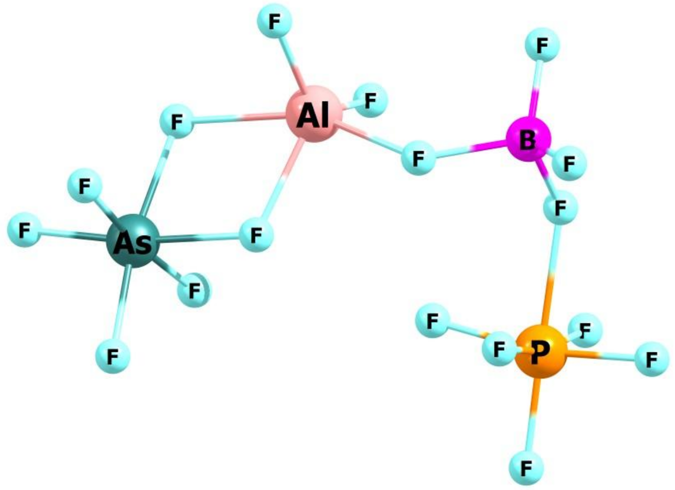

9.8

Al	-0.663209000	1.499023000	0.333448000
As	-2.864636000	-0.560270000	-0.098307000
B	2.341247000	1.826622000	-0.295904000
P	2.843364000	-1.280165000	0.043693000
F	-3.503499000	0.527008000	-1.286153000
F	-2.838933000	-1.850003000	-1.243359000
F	-2.567658000	0.875262000	0.965167000
F	-2.054037000	-1.529933000	1.080282000
F	0.915984000	1.440217000	-0.657641000
F	2.295219000	2.907770000	0.542776000
F	-0.058781000	1.289198000	1.885060000
F	2.827159000	0.658793000	0.486564000
F	1.284956000	-1.233422000	0.493336000
F	3.278060000	-1.415323000	1.600784000
F	2.875813000	-2.860069000	-0.272861000
F	3.035814000	1.968580000	-1.461217000
F	4.397162000	-1.008500000	-0.347881000
F	-4.420049000	-0.872705000	0.576083000
F	2.406256000	-0.819751000	-1.450124000
F	-1.149654000	-0.004707000	-0.669102000
F	-1.301808000	2.935472000	-0.271332000

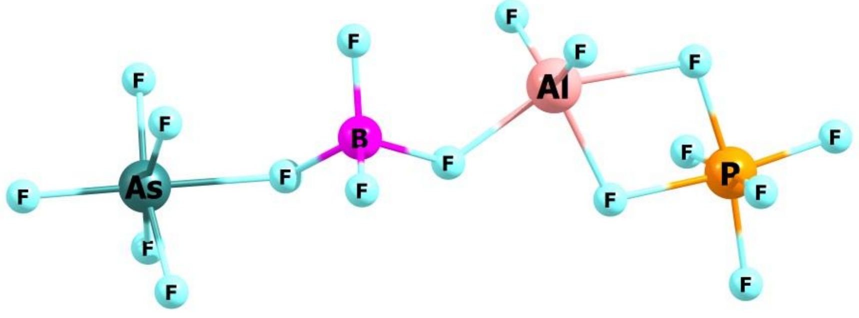

9.9

Al	-1.878848000	0.815093000	-0.429221000
P	-4.455687000	-0.557081000	0.129916000
B	1.050073000	0.854431000	0.419942000
As	3.928589000	-0.377217000	-0.039788000
F	-4.153528000	-1.454003000	-1.187944000
F	-4.654536000	-1.882924000	1.031146000
F	-3.953000000	0.815027000	-0.755848000
F	-4.561255000	0.393623000	1.440976000
F	-0.302483000	0.150057000	0.318860000

F	1.078304000	1.848254000	-0.509415000
F	-1.969216000	2.297781000	0.355677000
F	1.273486000	1.153780000	1.724648000
F	5.651705000	-0.484729000	-0.074325000
F	3.771877000	0.040257000	-1.715244000
F	3.650310000	-2.041489000	-0.444621000
F	1.925806000	-0.296937000	-0.013232000
F	3.869949000	1.306189000	0.371567000
F	-6.004160000	-0.343001000	-0.260370000
F	3.789962000	-0.780171000	1.641618000
F	-2.679201000	-0.627066000	0.455688000
F	-1.582182000	0.564906000	-2.063133000

$BP_3F_{19}^-$

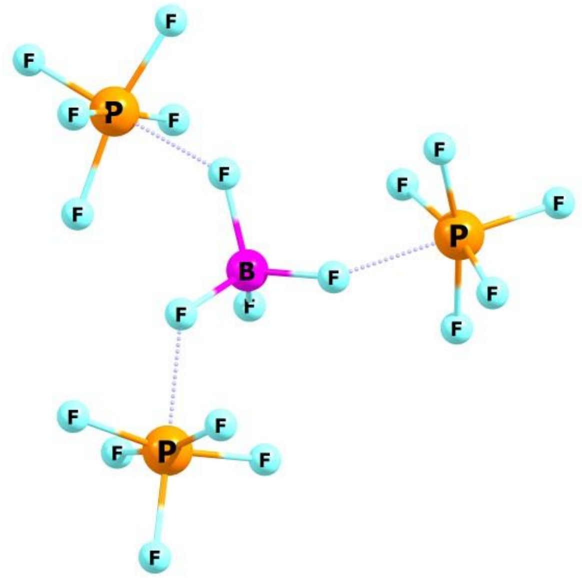

0.0

B	0.000162000	0.000094000	0.146893000
F	1.277682000	-0.385976000	-0.442721000
F	-0.304275000	1.299435000	-0.442726000
F	-0.972892000	-0.913301000	-0.442622000
F	0.000217000	0.000122000	1.497056000
P	-1.214487000	-2.906629000	-0.034390000
F	-2.573477000	-2.682558000	-0.889027000
F	-1.979558000	-2.370698000	1.287714000
F	0.197673000	-2.764169000	0.745381000
F	-0.401078000	-3.096695000	-1.424840000
F	-1.434198000	-4.474060000	0.254575000
P	3.124625000	0.401640000	-0.034396000
F	2.882662000	1.200990000	-1.424954000
F	3.610176000	-0.887378000	-0.888869000
F	3.043087000	-0.528721000	1.287846000
F	2.294976000	1.553383000	0.745139000
F	4.591849000	0.995231000	0.254613000
P	-1.910268000	2.504978000	-0.034379000
F	-1.037041000	3.569977000	-0.889185000
F	-1.063662000	2.899966000	1.287634000
F	-2.492389000	1.210598000	0.745491000
F	-2.481474000	1.895253000	-1.424740000
F	-3.158154000	3.478568000	0.254571000

$BP_3F_{19}^-$

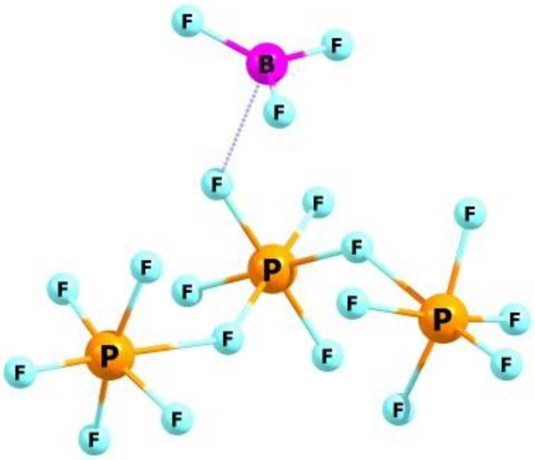

2.1

P	0.115355000	-0.827596000	1.500376000
P	2.517448000	0.999084000	-0.450959000
P	-2.421696000	1.281272000	-0.286042000
B	-0.478206000	-2.727357000	-1.514009000
F	0.576966000	-3.527611000	-1.753213000
F	0.851528000	0.528879000	0.717258000
F	1.489094000	-1.096073000	2.322172000
F	3.780744000	1.431903000	-1.351090000
F	-1.213101000	1.270774000	-1.346551000
F	-0.425775000	0.208001000	2.630248000
F	0.651587000	-1.772389000	0.256012000
F	-0.612412000	-1.587209000	-2.198532000
F	-1.489418000	-3.187413000	-0.761862000
F	-1.254475000	-0.498407000	0.563630000
F	1.635137000	2.329725000	-0.721796000
F	-3.381325000	0.923430000	0.966610000
F	3.113296000	-0.421914000	0.036419000
F	-1.503789000	2.147754000	0.714230000
F	1.697015000	0.238927000	-1.616352000
F	-0.652360000	-2.088518000	2.180672000
F	-3.289631000	2.473796000	-0.907950000
F	3.041008000	1.663731000	0.933364000
F	-3.100265000	0.056545000	-1.094447000

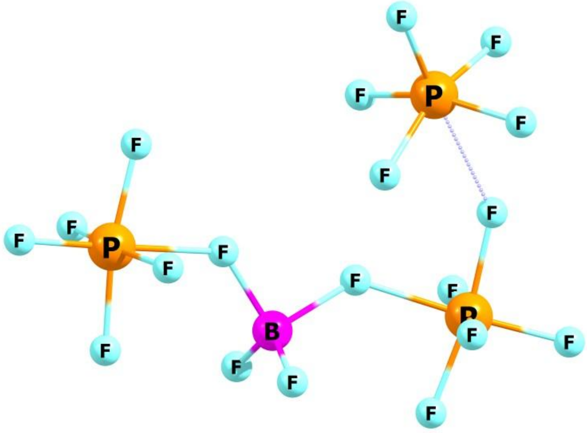

2.5

P	-1.685131000	2.244414000	-0.007633000
P	-2.261997000	-1.600837000	0.000543000
P	3.422059000	0.233928000	0.005495000
B	0.853993000	-1.509966000	-0.027062000

	F	4.955941000	0.653619000	0.287638000	
	F	-1.606140000	-3.062309000	0.259915000	
	F	-1.924868000	-1.156506000	1.521641000	
	F	-2.918819000	2.540741000	-1.011246000	
	F	-2.703220000	-0.030855000	-0.326371000	
	F	3.661321000	-0.140626000	-1.558548000	
	F	1.559557000	-0.234511000	-0.360876000	
	F	3.611432000	-1.333934000	0.391686000	
	F	-0.668706000	1.441441000	0.940445000	
	F	0.867878000	-1.753949000	1.303441000	
	F	-0.839282000	1.741556000	-1.280155000	
	F	-0.566799000	-1.003276000	-0.445254000	
	F	1.200268000	-2.497027000	-0.892700000	
	F	-2.755669000	2.226260000	1.202048000	
	F	2.906280000	0.530356000	1.515242000	
	F	-1.118845000	3.721779000	0.151235000	
	F	-3.776773000	-2.051485000	0.344978000	
	F	2.929282000	1.715759000	-0.441593000	
	F	-2.412165000	-1.930673000	-1.583832000	
**$BAs_3F_{19}^-$**	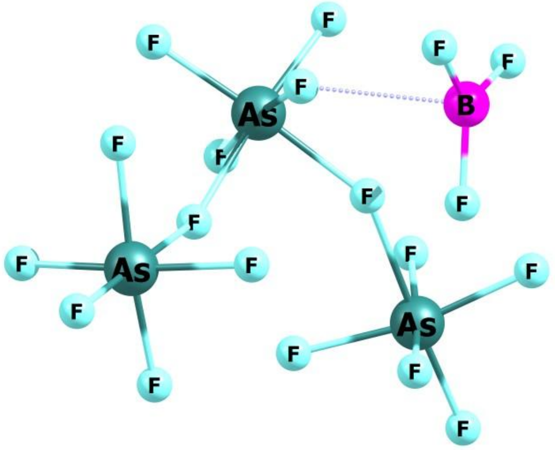				**0.0**
	As	-0.044431000	0.890154000	1.422227000	
	As	-2.690251000	-0.889778000	-0.390249000	
	As	2.561011000	-1.095270000	-0.291436000	
	B	0.444318000	2.759307000	-1.812256000	
	F	-0.592181000	3.589948000	-2.015455000	
	F	-0.986725000	-0.526265000	0.712490000	
	F	-1.365258000	1.266217000	2.462660000	
	F	-4.109498000	-1.248843000	-1.301418000	
	F	1.579063000	-1.216652000	-1.708593000	
	F	0.627997000	-0.251962000	2.520341000	
	F	-0.681082000	1.878572000	0.139757000	
	F	0.498101000	1.591297000	-2.453939000	
	F	1.487308000	3.180616000	-1.083218000	
	F	1.258397000	0.431437000	0.197883000	
	F	-1.844020000	-2.343563000	-0.794140000	
	F	3.319212000	-0.708602000	1.218596000	
	F	-3.251807000	0.632077000	0.216036000	
	F	1.459384000	-2.133750000	0.542304000	
	F	-1.857880000	-0.063956000	-1.659662000	
	F	0.968180000	2.168018000	1.987077000	
	F	3.681237000	-2.328917000	-0.733557000	

$BAs_3F_{19}^-$	F	-3.233018000	-1.642083000	1.076078000	
	F	3.432541000	0.208071000	-1.031754000	
	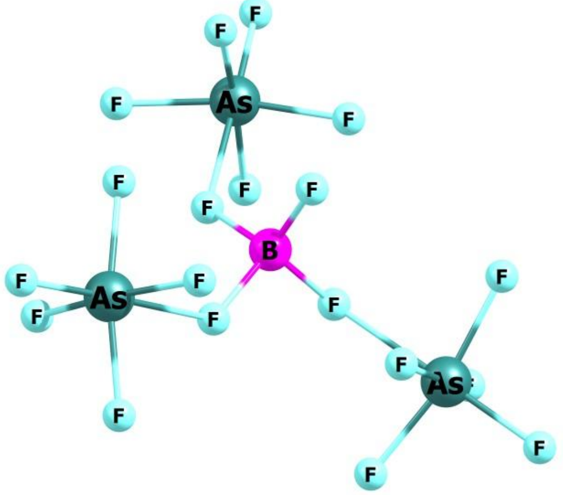				**0.2**
	B	-0.000182000	0.000090000	0.187024000	
	F	1.016417000	0.864629000	-0.414082000	
	F	-1.257099000	0.448030000	-0.414143000	
	F	0.240466000	-1.312557000	-0.413674000	
	F	-0.000263000	0.000395000	1.529549000	
	As	1.800319000	-2.617912000	-0.032236000	
	F	0.814953000	-3.701535000	-0.954506000	
	F	0.893438000	-3.023462000	1.383283000	
	F	2.492608000	-1.276885000	0.814748000	
	F	2.440245000	-1.981464000	-1.511313000	
	F	3.088244000	-3.723275000	0.255372000	
	As	1.367291000	2.867797000	-0.032242000	
	F	0.497678000	3.104262000	-1.512159000	
	F	2.799225000	2.555868000	-0.953132000	
	F	2.170376000	2.284994000	1.384097000	
	F	-0.141086000	2.797370000	0.813261000	
	F	1.680874000	4.535772000	0.255721000	
	As	-3.167565000	-0.249987000	-0.032236000	
	F	-3.613500000	1.145795000	-0.953484000	
	F	-3.064641000	0.737290000	1.383837000	
	F	-2.352163000	-1.520672000	0.813607000	
	F	-2.937099000	-1.121518000	-1.511954000	
	F	-4.768736000	-0.812715000	0.255689000	
	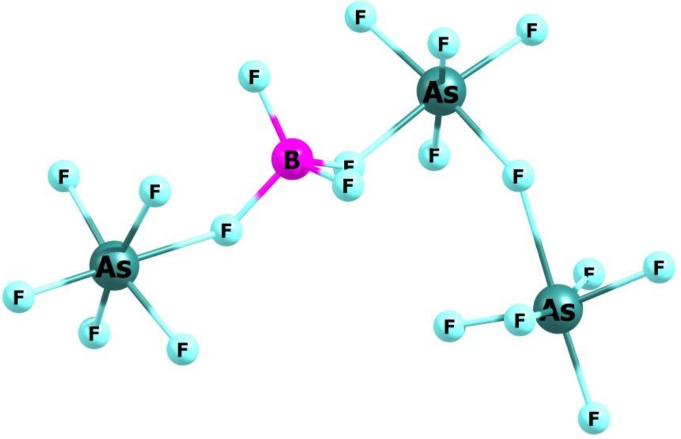				**1.8**
	As	1.646165000	-1.857306000	-0.131275000	

	As	-4.009132000	0.522113000	-0.130566000	
	As	2.898891000	1.623202000	-0.000350000	
	B	-1.223965000	-0.756929000	0.609305000	
	F	2.361715000	-0.309873000	0.559064000	
	F	3.967345000	0.833933000	-1.111219000	
	F	-0.862159000	-0.027210000	1.682417000	
	F	0.813250000	-3.232098000	-0.754115000	
	F	-2.044120000	0.026153000	-0.347729000	
	F	-3.393182000	2.018582000	0.484655000	
	F	1.620311000	1.493374000	-1.156117000	
	F	-3.845692000	-0.173838000	1.449411000	
	F	-3.812723000	1.121378000	-1.743702000	
	F	2.046964000	-1.288456000	-1.703511000	
	F	-1.678704000	-2.020670000	0.747023000	
	F	3.370513000	3.227638000	-0.408185000	
	F	-4.313107000	-1.058269000	-0.773164000	
	F	-5.670669000	0.952610000	0.026744000	
	F	0.072280000	-0.834824000	-0.356623000	
	F	4.078408000	1.395877000	1.250232000	
	F	1.740728000	2.063384000	1.205057000	
	F	1.108521000	-2.166440000	1.477126000	
	F	3.155249000	-2.656764000	0.095499000	
**$AlP_3F_{19}^-$**	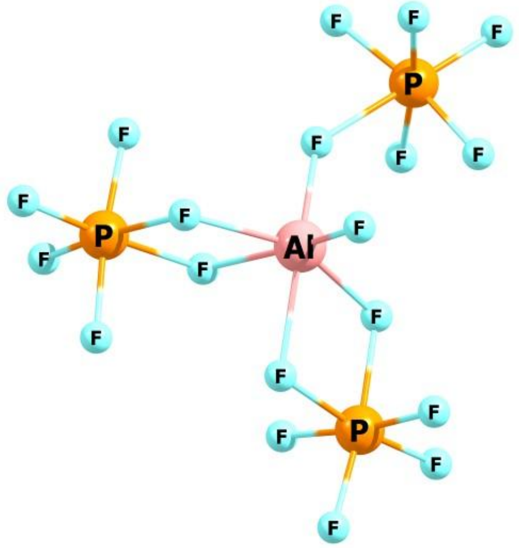				**0.0**
	Al	-0.144024000	0.031790000	0.776068000	
	F	1.469011000	-0.570665000	0.228750000	
	F	-0.128561000	1.566841000	-0.341978000	
	F	-0.829291000	-0.970035000	-0.809625000	
	F	0.191320000	0.584379000	2.332589000	
	P	-1.382198000	-2.485312000	-0.145584000	
	F	-1.814912000	-3.024066000	-1.600850000	
	F	-2.822849000	-1.779516000	0.095613000	
	F	-0.835764000	-1.672098000	1.334485000	
	F	0.129619000	-3.039189000	-0.308523000	
	F	-1.823722000	-3.784308000	0.705352000	
	P	3.141421000	0.181187000	-0.179359000	
	F	2.444879000	0.646449000	-1.579727000	
	F	3.471741000	-1.270684000	-0.837044000	
	F	3.612278000	-0.378843000	1.274656000	

$AlP_3F_{19}^-$	F	2.574824000	1.534405000	0.533658000	
	F	4.584525000	0.821198000	-0.541298000	
	P	-1.763283000	2.255625000	-0.211000000	
	F	-1.334385000	3.502160000	-1.138653000	
	F	-1.255949000	3.004604000	1.130237000	
	F	-1.963041000	0.811889000	0.757093000	
	F	-2.159162000	1.362317000	-1.503407000	
	F	-3.295760000	2.690074000	0.040927000	
	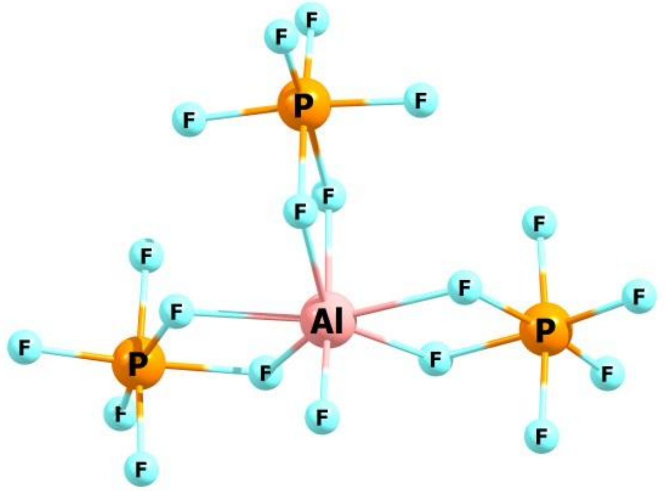				**2.9**
	Al	0.001566000	-0.430940000	-0.544274000	
	F	-0.001220000	0.883854000	0.811281000	
	F	-2.010624000	-0.014154000	-0.858677000	
	F	2.006802000	-0.012796000	-0.857087000	
	F	-1.184860000	-1.525951000	0.583212000	
	P	2.845240000	-1.089861000	0.188352000	
	F	4.284271000	-0.543776000	-0.305927000	
	F	2.792205000	-2.205041000	-0.990290000	
	F	1.189295000	-1.526992000	0.586072000	
	F	2.747587000	0.040902000	1.352684000	
	F	3.407170000	-2.180574000	1.246022000	
	P	-0.002110000	2.507353000	0.015497000	
	F	-1.615644000	2.429621000	0.017427000	
	F	-0.001448000	1.464805000	-1.369311000	
	F	1.611676000	2.431126000	0.017516000	
	F	-0.002402000	3.217993000	1.465857000	
	F	-0.002607000	3.839607000	-0.895561000	
	P	-2.843692000	-1.092124000	0.188072000	
	F	-4.285806000	-0.550461000	-0.302123000	
	F	-2.745890000	0.038825000	1.352361000	
	F	0.000166000	-1.314544000	-1.978652000	
	F	-2.790581000	-2.208111000	-0.989717000	
	F	-3.399415000	-2.184145000	1.247888000	

**$AlAs_3F_{19}^-$**	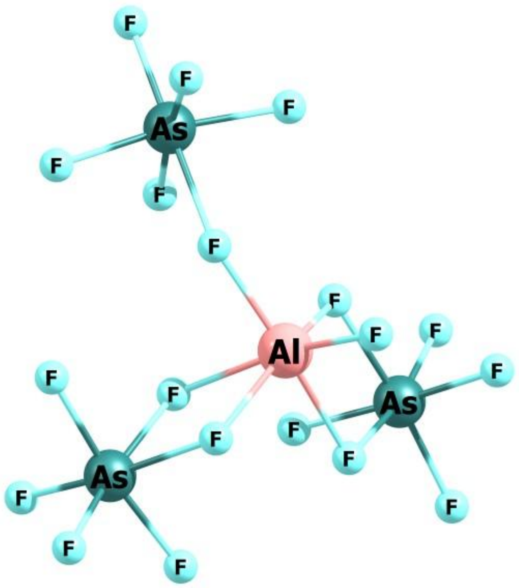	**0.0**

Al	-0.291784000	0.019440000	1.027405000
F	1.510151000	0.174384000	0.859263000
F	-0.689188000	1.562856000	-0.008423000
F	-0.227460000	-0.991069000	-0.670127000
F	-0.460462000	0.568714000	2.615684000
As	-0.215045000	-2.761894000	-0.148101000
F	-0.137659000	-3.323797000	-1.772138000
F	-1.943863000	-2.705276000	-0.175829000
F	-0.308360000	-1.848240000	1.482225000
F	1.499506000	-2.642584000	-0.017755000
F	-0.231727000	-4.288268000	0.654586000
As	2.845589000	1.162867000	-0.160598000
F	1.731143000	1.039707000	-1.490602000
F	3.492969000	-0.393615000	-0.570708000
F	3.767576000	1.148167000	1.311822000
F	2.009557000	2.586142000	0.387259000
F	4.033701000	2.046331000	-1.056862000
As	-2.546214000	1.540897000	-0.231182000
F	-2.509804000	3.036173000	-1.084314000
F	-2.657582000	2.344182000	1.292840000
F	-2.240828000	-0.025036000	0.703648000
F	-2.280363000	0.640264000	-1.680877000
F	-4.245054000	1.256029000	-0.284159000

$AlAs_3F_{19}^-$

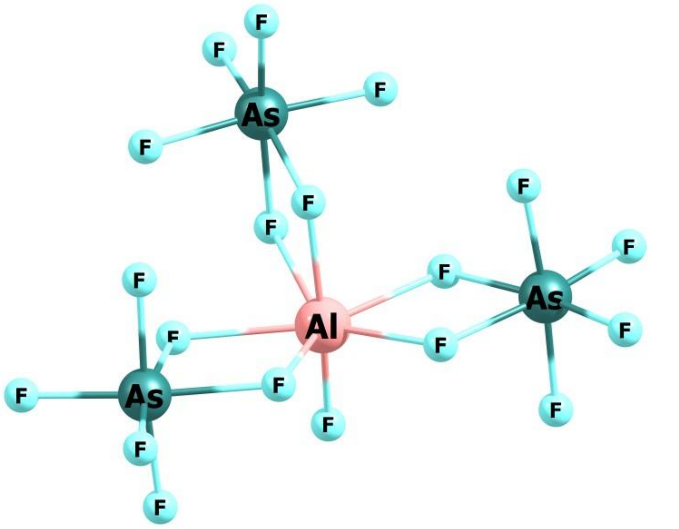

4.1

Al	0.000453000	-0.480580000	-0.581197000
F	-0.000427000	0.807303000	0.792350000
F	-1.990743000	-0.009276000	-0.919732000
F	1.988097000	-0.010856000	-0.919931000
F	-1.184515000	-1.599382000	0.548457000
As	2.932557000	-1.133307000	0.157369000
F	4.450417000	-0.506019000	-0.375264000
F	2.899418000	-2.308034000	-1.113783000
F	1.185720000	-1.600807000	0.550015000
F	2.811971000	0.053428000	1.414922000
F	3.545215000	-2.304219000	1.272812000
As	0.000136000	2.541632000	0.007838000
F	-1.723169000	2.470358000	0.014046000
F	-0.000485000	1.435062000	-1.443495000
F	1.723339000	2.468911000	0.013665000
F	0.000596000	3.260633000	1.576486000
F	0.000631000	3.975690000	-0.950334000
As	-2.932560000	-1.133060000	0.157315000
F	-4.452087000	-0.507606000	-0.372860000
F	-2.811711000	0.053017000	1.415601000
F	-0.001572000	-1.380530000	-2.005197000
F	-2.899754000	-2.307541000	-1.114067000
F	-3.542083000	-2.305264000	1.273237000

$BP_2AsF_{19}^-$

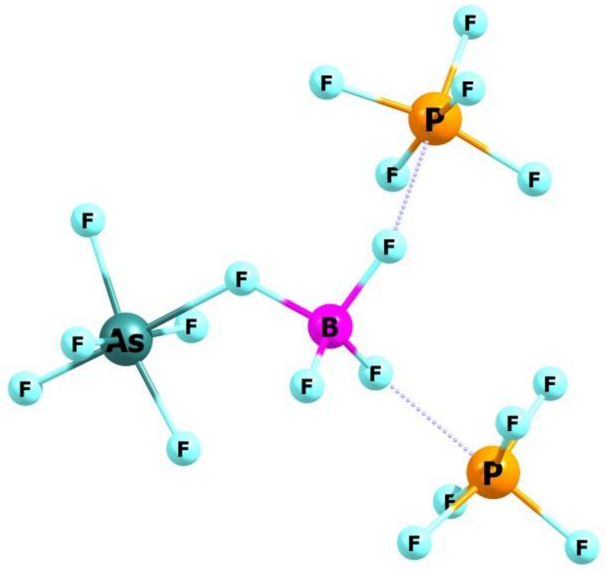

0.0

P	-1.683818000	2.587020000	-0.141374000
P	-2.038068000	-2.370349000	-0.162118000
As	2.886758000	-0.124140000	-0.141995000
B	-0.104025000	-0.066709000	0.926651000
F	-2.541609000	-1.580590000	1.160155000
F	-0.286279000	3.252312000	0.329826000
F	4.415943000	-0.742654000	-0.652790000
F	1.123577000	0.644683000	0.447075000
F	-2.825599000	-1.291896000	-1.072078000
F	2.168138000	-0.160476000	-1.718221000
F	-1.051509000	0.994573000	1.148098000
F	-0.529790000	-0.870144000	-0.182665000
F	-2.355020000	3.096085000	1.243095000
F	-1.236326000	-2.857236000	-1.479593000
F	-2.956847000	1.619244000	-0.367710000
F	-3.174403000	-3.504463000	-0.173832000
F	2.180347000	-1.667772000	0.201801000
F	3.294728000	1.544474000	-0.380106000
F	-0.972029000	-3.169028000	0.752862000
F	-2.178703000	3.797316000	-1.070071000
F	0.144767000	-0.755707000	2.067092000
F	-0.878963000	1.756586000	-1.269079000
F	3.335733000	0.025816000	1.527802000

$BP_2AsF_{19}^-$

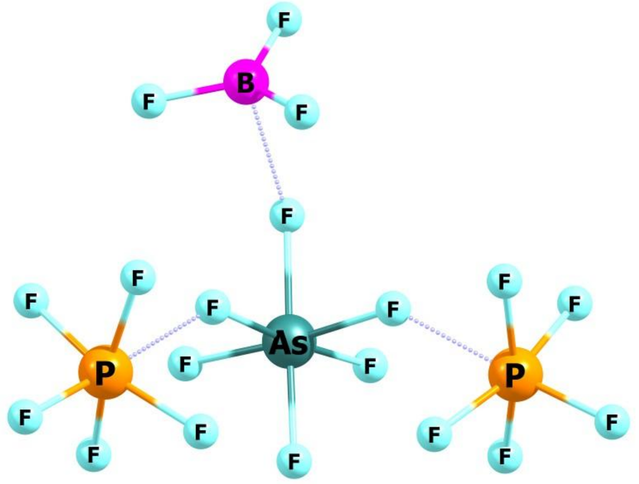

0.3

P	-2.576642000	-1.448857000	-0.389145000
P	2.968397000	-0.759262000	-0.590161000
As	0.098106000	0.533178000	1.273194000
B	-1.028955000	2.907497000	-1.305016000
F	-1.262368000	-2.355095000	-0.621940000
F	-2.401465000	-0.853970000	-1.884021000
F	3.096537000	-1.036408000	0.998395000
F	-3.610387000	-0.244991000	-0.068310000
F	3.744975000	0.650515000	-0.380202000
F	-2.476290000	-1.743767000	1.196280000
F	-1.126811000	0.089932000	0.015993000
F	4.261360000	-1.610688000	-1.032188000
F	1.314334000	0.379393000	-0.084598000
F	-0.338319000	2.039899000	-2.052530000
F	-2.288925000	2.637561000	-0.938502000
F	-0.536245000	4.142471000	-1.092255000
F	0.312477000	-1.135752000	1.646167000
F	1.898032000	-1.964341000	-0.702880000
F	-3.672139000	-2.569017000	-0.740514000
F	-1.154253000	0.738314000	2.449952000
F	-0.108495000	2.186003000	0.708566000
F	1.356760000	1.032751000	2.349854000
F	2.550218000	-0.272876000	-2.078461000

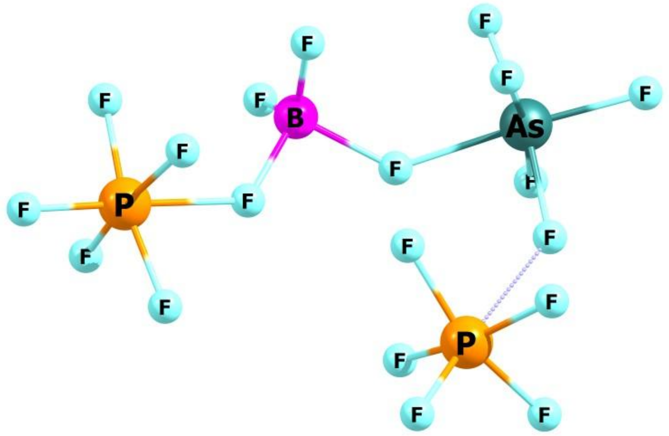

1.0

P	-3.526795000	-0.138176000	0.046400000
P	0.905342000	2.529832000	-0.029692000
As	2.390452000	-1.124402000	0.035412000

$BP_2AsF_{19}^-$	B	-0.792481000	-1.623463000	-0.151840000	
	F	0.113874000	1.483950000	0.895366000	
	F	-1.643737000	-0.444371000	-0.450312000	
	F	0.260781000	1.821867000	-1.323392000	
	F	-0.040997000	3.801811000	0.091880000	
	F	1.895031000	2.814691000	1.216491000	
	F	2.467767000	0.619115000	-0.265530000	
	F	-3.164117000	1.399066000	-0.328069000	
	F	-5.069335000	0.140765000	0.428909000	
	F	-3.570737000	-1.737546000	0.335036000	
	F	-1.003369000	-2.619836000	-1.050722000	
	F	-3.814983000	-0.434282000	-1.525685000	
	F	4.051319000	-1.210939000	0.507665000	
	F	1.828664000	-0.785641000	1.634058000	
	F	2.072630000	-2.813920000	0.249757000	
	F	0.571860000	-0.940937000	-0.558594000	
	F	2.747013000	-1.380661000	-1.642378000	
	F	-0.758890000	-1.915085000	1.167740000	
	F	-2.948829000	0.102210000	1.541582000	
	F	2.050417000	3.138377000	-0.997138000	
	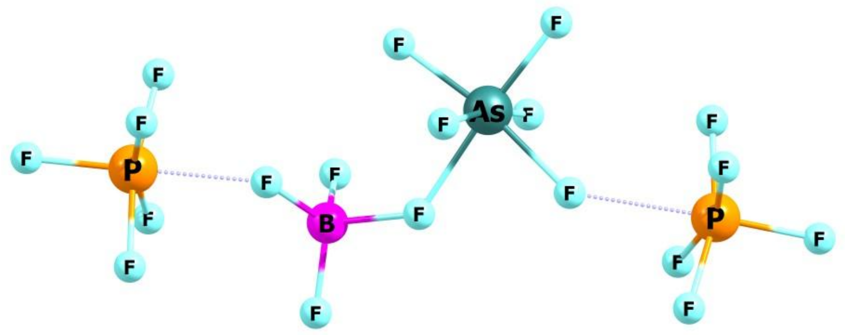				**1.4**
	P	4.457940000	0.071655000	-0.246032000	
	P	-4.300239000	0.541825000	-0.191979000	
	As	-0.845161000	-0.922143000	0.127644000	
	B	1.606615000	1.096822000	0.475482000	
	F	-4.521186000	-0.662553000	-1.247628000	
	F	0.170253000	0.689798000	-0.085224000	
	F	0.558062000	-1.678007000	0.789614000	
	F	1.741818000	2.386917000	0.068027000	
	F	1.623415000	0.818073000	1.800800000	
	F	6.064782000	-0.037986000	-0.221133000	
	F	-4.019455000	1.531610000	-1.439233000	
	F	-2.147080000	0.070485000	-0.609716000	
	F	-4.135361000	-0.556122000	0.976552000	
	F	-1.300418000	-0.293960000	1.670577000	
	F	-1.860184000	-2.311131000	0.249371000	
	F	-5.837909000	0.913178000	0.050153000	
	F	4.196165000	-1.007652000	0.932908000	
	F	4.342810000	1.266113000	0.847878000	
	F	4.386930000	1.179902000	-1.429151000	
	F	-3.640073000	1.642697000	0.786243000	
	F	4.213773000	-1.092152000	-1.346963000	
	F	2.442445000	0.181119000	-0.310913000	
	F	-0.335264000	-1.290950000	-1.484331000	

$BP_2AsF_{19}^-$

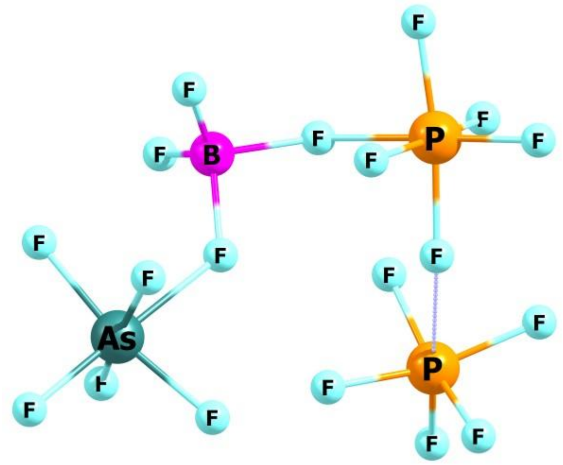

1.6

P	-2.260600000	2.023901000	0.127321000
P	-1.886225000	-1.817598000	-0.431555000
As	2.854841000	0.413548000	-0.278834000
B	0.693842000	-1.299528000	1.260896000
F	-3.767166000	1.495526000	-0.112490000
F	1.225786000	-2.512878000	0.975428000
F	-2.184889000	2.350800000	-1.452487000
F	1.047173000	-0.282040000	0.217476000
F	-0.840375000	-1.352575000	1.059805000
F	-1.762531000	-0.211470000	-0.835689000
F	3.440586000	-0.559980000	1.034138000
F	2.943083000	-0.968267000	-1.321808000
F	2.507156000	1.687991000	0.845711000
F	-0.656400000	2.033346000	0.162225000
F	0.861226000	-0.783412000	2.507878000
F	-2.827058000	-2.160202000	-1.698860000
F	-3.124547000	-1.447656000	0.549023000
F	-2.245281000	1.200088000	1.511602000
F	1.971588000	1.267582000	-1.499673000
F	4.404750000	1.033919000	-0.725893000
F	-2.573123000	3.482973000	0.682922000
F	-1.863604000	-3.332489000	0.149442000
F	-0.498216000	-2.079476000	-1.219801000

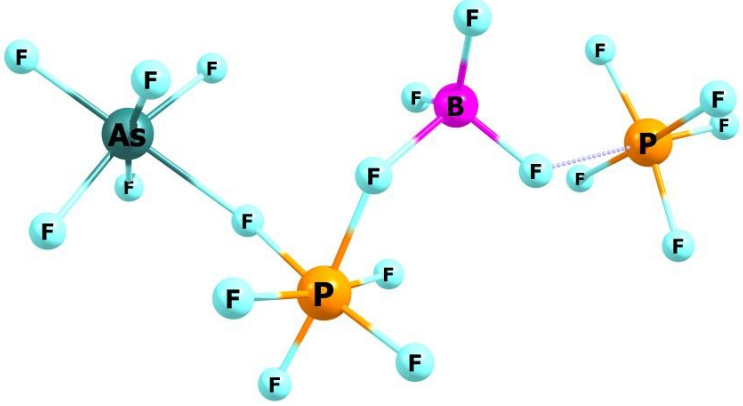

3.6

P	4.306138000	-0.344671000	-0.181143000
P	-0.650346000	1.694460000	0.037891000
As	-3.335908000	-0.711822000	-0.096707000
B	1.271337000	-0.754984000	0.516420000
F	0.060396000	0.329759000	0.918093000

	F	2.354420000	0.184182000	0.337266000	
	F	-3.822490000	-0.392158000	-1.730559000	
	F	4.499570000	1.261255000	-0.149353000	
	F	0.310254000	1.320077000	-1.205147000	
	F	1.360107000	-1.459803000	1.668532000	
	F	-2.576419000	-0.820522000	1.452832000	
	F	3.729687000	-1.858930000	-0.107729000	
	F	-2.293960000	-1.965505000	-0.675232000	
	F	-1.756810000	0.503458000	-0.628311000	
	F	4.520281000	-0.378903000	1.424338000	
	F	-4.664938000	-1.744258000	0.279479000	
	F	-1.632902000	1.833733000	1.308645000	
	F	3.739518000	-0.228010000	-1.691311000	
	F	-1.414295000	2.852040000	-0.789176000	
	F	5.815977000	-0.723995000	-0.575524000	
	F	-4.117073000	0.753158000	0.395341000	
	F	0.863908000	-1.359103000	-0.616305000	
	F	0.457146000	2.673327000	0.690566000	
**$BPAs_2F_{19}^-$**	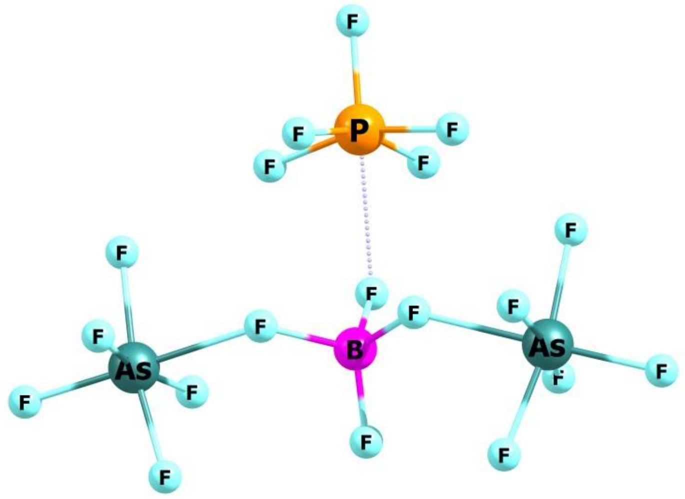				**0.0**
	As	2.985925000	-1.155147000	0.090819000	
	As	-3.182096000	-0.873987000	0.091989000	
	P	0.296106000	2.954236000	0.043765000	
	B	-0.068812000	-0.489818000	-0.493886000	
	F	-0.143010000	-1.467719000	-1.422076000	
	F	4.647658000	-1.515540000	-0.205349000	
	F	-1.220099000	-0.533199000	0.463869000	
	F	2.970761000	-1.726904000	1.728628000	
	F	-0.184702000	3.393814000	-1.435146000	
	F	2.400478000	-2.698823000	-0.434058000	
	F	-4.859495000	-1.164243000	-0.192626000	
	F	-3.391873000	0.773040000	0.582947000	
	F	1.780756000	2.759085000	-0.548012000	
	F	-2.677258000	-2.473197000	-0.340547000	
	F	-1.245774000	2.677976000	0.413508000	
	F	-3.200306000	-1.350225000	1.760043000	
	F	3.276261000	0.448517000	0.674867000	
	F	0.039030000	0.810114000	-0.991809000	
	F	1.045409000	-0.734447000	0.475516000	
	F	2.658078000	-0.507979000	-1.483679000	
	F	0.717429000	2.033481000	1.298629000	

$BPAs_2F_{19}^-$	F	-2.817384000	-0.332389000	-1.513831000	
	F	0.468054000	4.397187000	0.700270000	
	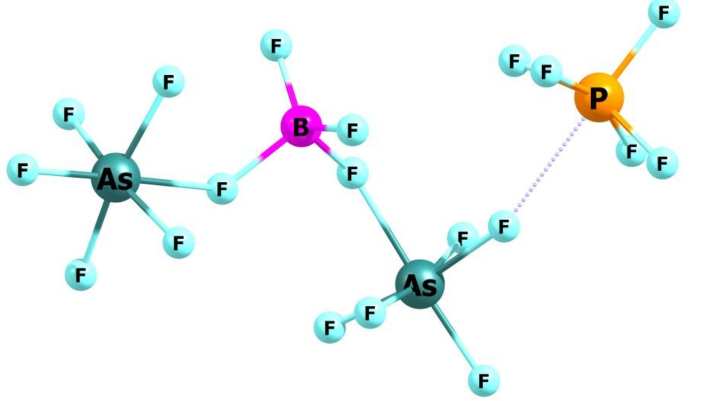				**0.5**
	As	3.760021000	-0.362851000	-0.124439000	
	As	-1.271071000	1.713419000	-0.002051000	
	P	-3.697294000	-1.355600000	-0.104900000	
	B	0.702320000	-0.747813000	0.525813000	
	F	-0.357830000	0.311535000	0.974454000	
	F	1.886237000	0.161132000	0.372696000	
	F	-4.274497000	-0.978271000	-1.566470000	
	F	4.063054000	1.335096000	0.048654000	
	F	-0.084105000	1.498264000	-1.237015000	
	F	0.853021000	-1.552939000	1.607555000	
	F	-2.787396000	-1.327871000	1.223740000	
	F	3.138931000	-1.980466000	-0.208188000	
	F	-2.546002000	-2.214619000	-0.830781000	
	F	-2.206930000	0.402701000	-0.748084000	
	F	3.995192000	-0.561509000	1.583378000	
	F	-4.697721000	-2.538343000	0.274531000	
	F	-2.360543000	1.682361000	1.341637000	
	F	3.244644000	-0.094302000	-1.756327000	
	F	-2.148058000	2.929805000	-0.857265000	
	F	5.382087000	-0.781314000	-0.544549000	
	F	-4.540193000	-0.116555000	0.492777000	
	F	0.325239000	-1.275423000	-0.659242000	
	F	-0.239301000	2.823420000	0.835010000	
	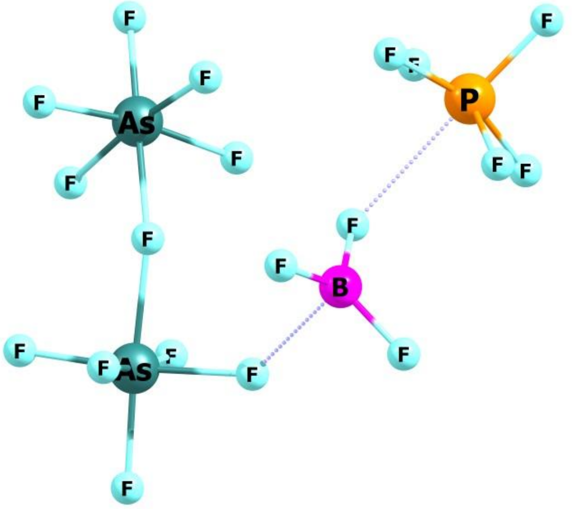				**1.5**
	As	2.410501000	-1.197834000	-0.163988000	

$BPAs_2F_{19}^-$

As	0.804749000	2.084459000	0.088789000
P	-3.756454000	-0.118708000	-0.165957000
B	-1.021480000	-2.015499000	0.501216000
F	-0.595757000	1.701937000	1.038881000
F	-1.356586000	-3.226207000	0.030784000
F	0.896772000	-2.113455000	-0.379437000
F	3.781153000	-0.177701000	0.112157000
F	0.343423000	3.728624000	-0.193125000
F	-3.750775000	-0.875712000	-1.592154000
F	-3.053600000	1.134758000	-0.873267000
F	-5.277001000	0.346735000	-0.134681000
F	-3.887221000	-1.576570000	0.532390000
F	3.352965000	-2.543680000	-0.715924000
F	2.437420000	-1.729448000	1.487944000
F	2.195171000	-0.559837000	-1.757027000
F	-0.633820000	-1.858202000	1.769195000
F	1.289842000	0.190824000	0.473847000
F	1.678424000	2.432611000	1.550543000
F	-1.399607000	-0.915546000	-0.213580000
F	2.286022000	2.192210000	-0.802923000
F	-3.273850000	0.452117000	1.258666000
F	0.006021000	1.463153000	-1.318416000

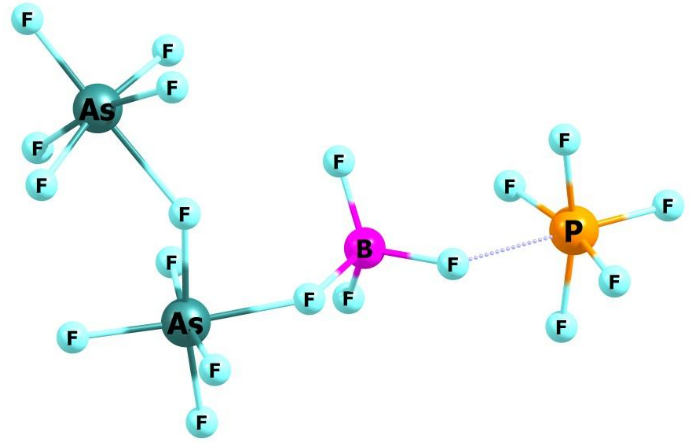

1.6

As	-1.060530000	1.697151000	0.108585000
As	-3.231317000	-1.248549000	0.013650000
P	4.651383000	-0.684370000	0.151587000
B	1.720536000	0.250277000	-0.525430000
F	-4.194924000	-0.142863000	0.937616000
F	-2.764529000	-2.035009000	1.486438000
F	6.133779000	-1.255135000	-0.081958000
F	0.615672000	0.954410000	0.499248000
F	-0.841710000	1.146908000	-1.507188000
F	3.971228000	-2.093409000	0.564744000
F	2.039273000	1.211849000	-1.420550000
F	2.734832000	0.063129000	0.496836000
F	-0.289587000	3.208705000	-0.205860000
F	-2.660038000	2.265414000	-0.165534000
F	-4.522606000	-2.328911000	-0.357771000
F	4.815897000	-0.328454000	1.722834000
F	4.110765000	-0.894428000	-1.361905000
F	1.145598000	-0.901285000	-0.928590000

$BPAs_2F_{19}^-$	F	-3.435890000	-0.238696000	-1.375896000	
	F	-1.674313000	0.000333000	0.516874000	
	F	4.987571000	0.857923000	-0.209257000	
	F	-1.136081000	1.999766000	1.807337000	
	F	-2.006322000	-2.133546000	-0.826350000	
	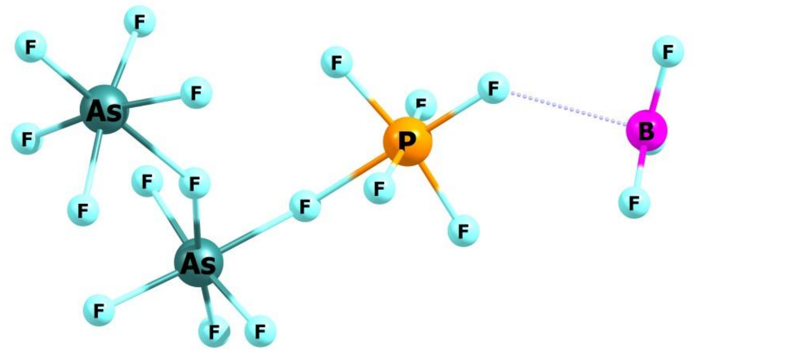				**1.6**
	As	-1.022924000	1.818072000	0.108032000	
	As	-2.755218000	-1.408771000	0.036551000	
	P	2.056090000	-0.032476000	-0.412443000	
	B	5.470938000	-0.903776000	0.430164000	
	F	-0.595090000	3.482421000	-0.083167000	
	F	-3.698207000	-0.146438000	-0.680254000	
	F	3.239442000	-1.152564000	-0.350448000	
	F	5.750171000	0.110159000	-0.398945000	
	F	-2.010936000	-1.702576000	-1.498302000	
	F	-1.285974000	-0.007137000	0.260157000	
	F	5.729429000	-2.164638000	0.046761000	
	F	-3.963563000	-2.631451000	-0.121769000	
	F	1.364421000	-0.462488000	0.981190000	
	F	-0.337865000	1.737462000	1.690133000	
	F	2.545244000	0.611858000	-1.818472000	
	F	2.896217000	1.110603000	0.374606000	
	F	-1.567452000	1.687656000	-1.524150000	
	F	5.104428000	-0.657892000	1.693793000	
	F	0.654397000	1.375210000	-0.548985000	
	F	-3.267005000	-0.874841000	1.605373000	
	F	-1.577085000	-2.430679000	0.788905000	
	F	1.009965000	-0.963041000	-1.215539000	
	F	-2.603577000	2.133827000	0.717401000	

$BPAs_2F_{19}^-$

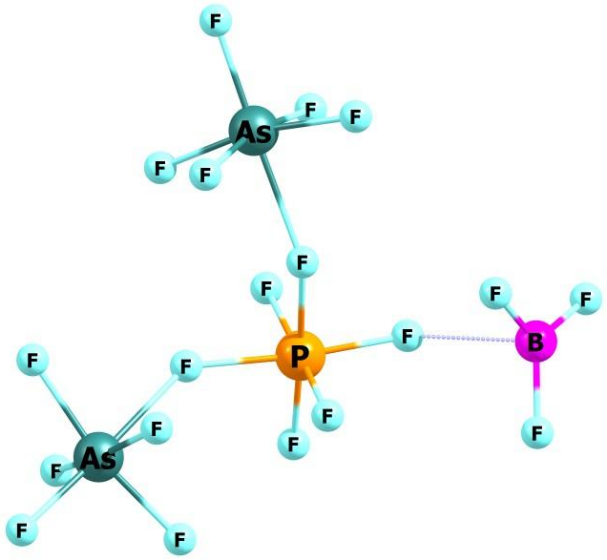

2.8

As	-1.870651000	1.972965000	0.071775000
As	3.079294000	0.083266000	0.251854000
P	-0.145560000	-1.073875000	-0.812047000
B	-2.741012000	-3.079624000	0.744373000
F	-3.055246000	1.251408000	1.110878000
F	2.802022000	1.787785000	0.152462000
F	-1.067077000	0.070109000	0.141074000
F	0.780965000	-2.095087000	-1.648605000
F	0.227910000	-1.762556000	0.597859000
F	-0.519769000	-0.216920000	-2.125204000
F	1.191638000	0.022324000	-0.600398000
F	-1.500272000	-1.984643000	-0.961141000
F	-2.759358000	1.393489000	-1.297454000
F	3.600221000	0.096969000	-1.402324000
F	3.034248000	-1.644798000	0.195129000
F	-0.543588000	2.363311000	-0.963078000
F	-1.827652000	-4.045194000	0.921177000
F	-3.744161000	-3.281088000	-0.126528000
F	-2.570019000	3.549173000	0.075939000
F	-2.755007000	-2.010386000	1.544936000
F	2.230879000	0.046188000	1.757672000
F	-0.851161000	2.224007000	1.447624000
F	4.659119000	0.197088000	0.933213000

$BPAs_2F_{19}^-$

3.3

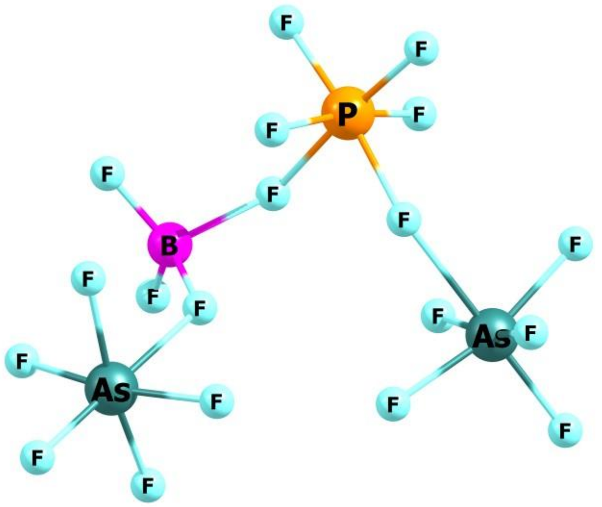

As	2.423075000	-1.457623000	0.103374000
As	-3.069598000	-0.625490000	-0.263887000
P	1.459883000	2.004987000	-0.439295000
B	-1.098440000	1.340796000	1.217836000
F	-2.713546000	-1.829375000	0.929077000
F	1.579379000	0.295412000	-0.710580000
F	-4.564406000	-1.326206000	-0.761813000
F	-2.072003000	-1.467761000	-1.395879000
F	3.098632000	-2.920533000	0.707581000
F	3.931834000	-0.644977000	-0.143771000
F	0.092193000	1.933438000	-1.290527000
F	-3.149102000	0.719993000	-1.351392000
F	2.722277000	1.895591000	0.556780000
F	2.433270000	-1.918693000	-1.565853000
F	-3.745634000	0.374531000	0.984915000
F	0.768542000	-1.934862000	0.207175000
F	-1.186757000	0.988704000	2.521982000
F	-1.294221000	0.191037000	0.320167000
F	2.267618000	-0.666411000	1.632443000
F	-1.702725000	2.468290000	0.784904000
F	2.375541000	2.292389000	-1.734669000
F	0.489159000	1.543361000	1.016520000
F	1.217639000	3.557620000	-0.062930000

3.4

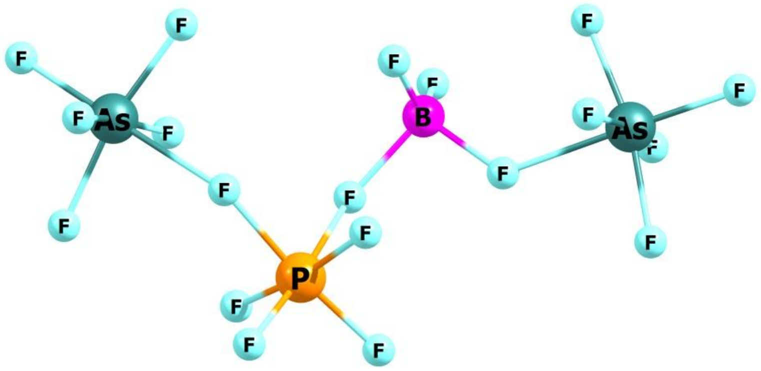

As	-3.613172000	-0.731772000	0.103948000
As	3.951618000	-0.278362000	0.147079000
P	-0.953514000	1.726666000	-0.039166000
B	0.915481000	-0.723616000	-0.558774000
F	5.572709000	-0.668406000	0.586467000

F	-0.213540000	0.353565000	-0.957488000
F	-4.077461000	-0.401202000	1.740454000
F	-4.935330000	-1.773453000	-0.258468000
F	4.231070000	-0.347849000	-1.562872000
F	0.537958000	-1.312809000	0.590921000
F	0.133023000	2.710271000	-0.715401000
F	1.044850000	-1.447441000	-1.693675000
F	-2.012497000	0.514170000	0.623957000
F	-4.398186000	0.731268000	-0.383832000
F	3.375780000	-1.913562000	0.097439000
F	-2.854960000	-0.830598000	-1.446042000
F	2.052372000	0.212946000	-0.377573000
F	-2.548890000	-1.965364000	0.679750000
F	-1.721188000	2.871858000	0.794702000
F	4.182660000	1.437379000	0.096107000
F	-1.941812000	1.837966000	-1.306043000
F	0.041924000	1.359290000	1.174724000
F	3.371136000	-0.139975000	1.772145000

$BPAs_2F_{19}^-$

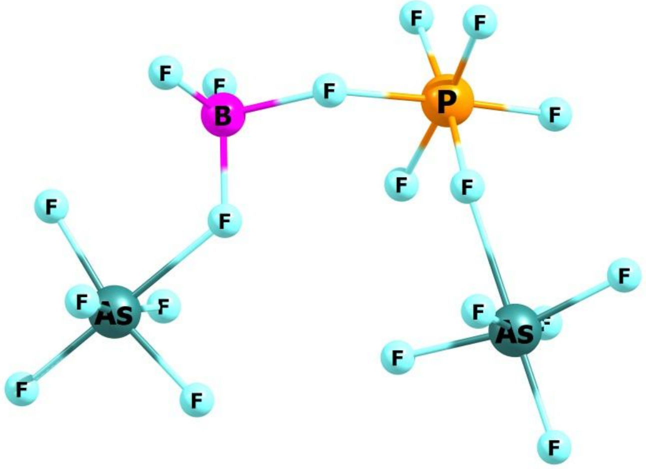

3.5

As	3.156385000	-0.776410000	-0.135729000
As	-2.572480000	-1.460647000	0.088213000
P	-1.561751000	1.988991000	-0.235457000
B	1.440343000	1.753104000	0.593185000
F	1.990701000	2.074579000	1.789472000
F	-1.000069000	3.381931000	-0.825399000
F	4.562462000	-1.694337000	-0.526620000
F	-2.008119000	0.513964000	0.561042000
F	-2.293846000	2.740856000	0.996403000
F	-0.874578000	-1.701077000	-0.069595000
F	-0.681978000	1.113638000	-1.260340000
F	1.479507000	0.303337000	0.337558000
F	-0.135117000	1.923940000	0.889359000
F	-2.422183000	-1.630464000	1.806113000
F	-4.171211000	-0.833607000	0.328158000
F	-2.888682000	1.951773000	-1.143495000
F	-3.050334000	-3.084871000	-0.217583000
F	2.026168000	-2.070707000	-0.296730000
F	-2.644067000	-0.925756000	-1.553500000
F	3.236169000	-1.073823000	1.570280000
F	2.816338000	-0.287217000	-1.761156000
F	3.988648000	0.726242000	0.115895000

<table>
<tr><td></td><td colspan="2">F 1.731934000 2.485212000 -0.502753000</td></tr>
<tr><td rowspan="2">$AlP_2AsF_{19}^-$</td><td>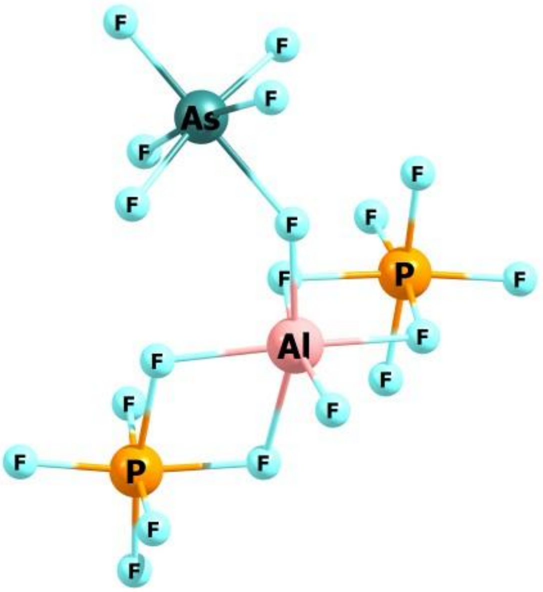
</td><td>0.0</td></tr>
<tr><td colspan="2">P -0.916549000 2.666973000 -0.190905000
P -2.382798000 -1.876913000 -0.289062000
As 2.768481000 -0.578172000 -0.144777000
Al -0.543480000 -0.002265000 0.984261000
F -2.442257000 -0.355328000 0.577990000
F 0.678629000 2.845634000 -0.005135000
F 4.108991000 -1.214591000 -1.035787000
F 1.263814000 0.131818000 0.870547000
F -2.247749000 -1.014788000 -1.652740000
F 1.665552000 -0.641906000 -1.488078000
F -0.917268000 1.837497000 1.381171000
F -0.644453000 -1.594310000 -0.040087000
F -1.218339000 4.079655000 0.529239000
F -2.069600000 -3.255219000 -1.062326000
F -2.498823000 2.319894000 -0.279474000
F -3.990805000 -1.926135000 -0.398458000
F 2.202850000 -2.139165000 0.373204000
F 3.124113000 1.076650000 -0.525714000
F -2.388358000 -2.617598000 1.149876000
F -0.878940000 3.198134000 -1.709392000
F -0.666694000 -0.528966000 2.583991000
F -0.603794000 1.022333000 -0.700046000
F 3.655972000 -0.417142000 1.340303000</td></tr>
</table>

$AlP_2AsF_{19}^-$

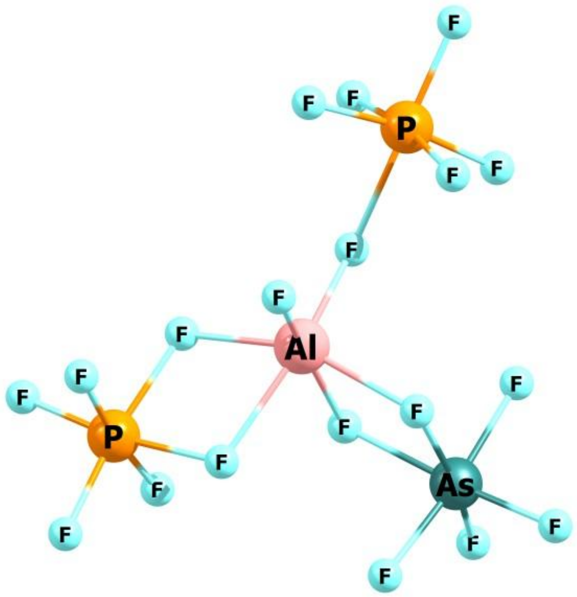

1.4

P	-2.756262000	-1.673804000	-0.179942000
P	3.151934000	-1.154943000	-0.141001000
As	-0.295406000	2.487452000	-0.040929000
Al	-0.166411000	-0.483702000	0.518175000
F	0.122333000	-1.354312000	1.928737000
F	-4.255552000	-1.419502000	0.356287000
F	1.353521000	-0.651831000	-0.439987000
F	-3.094236000	-2.879330000	-1.198245000
F	0.076914000	3.805988000	1.005377000
F	-2.425402000	-2.706428000	1.019902000
F	4.695406000	-1.583134000	0.099427000
F	3.520812000	0.282050000	-0.809043000
F	-1.928506000	2.434155000	0.528901000
F	2.541215000	-2.527677000	0.482450000
F	1.343462000	2.347082000	-0.553950000
F	3.061689000	-1.807494000	-1.632641000
F	-2.919724000	-0.570144000	-1.355762000
F	0.107845000	1.244253000	1.296067000
F	-1.035548000	-1.759777000	-0.618346000
F	-2.124902000	-0.397217000	0.834176000
F	-0.618009000	0.874375000	-0.874563000
F	2.985270000	-0.426517000	1.307089000
F	-0.742513000	3.388062000	-1.439375000

**$AlPAs_2F_{19}^-$**	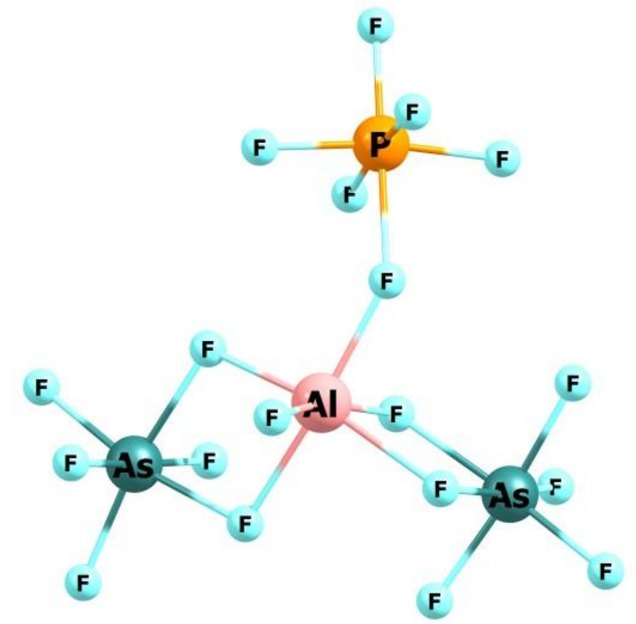	**0.0**

As	1.014387000	2.474418000	-0.140649000
As	-2.790330000	-0.594822000	-0.224118000
P	2.305651000	-2.279268000	-0.210190000
Al	-0.098212000	-0.093106000	1.001576000
F	-1.889238000	0.711734000	0.718945000
F	2.556389000	1.709270000	-0.050250000
F	2.999834000	-3.461133000	-1.072958000
F	1.503169000	-0.910481000	0.803353000
F	-2.220660000	0.157899000	-1.671195000
F	1.369098000	-1.756192000	-1.439645000
F	0.604469000	1.634776000	1.478816000
F	-1.084317000	-1.339097000	-0.042955000
F	1.594542000	3.876123000	0.680752000
F	-3.349011000	-1.972300000	-1.094748000
F	-0.604508000	3.084851000	-0.132983000
F	-4.248268000	0.324580000	-0.238727000
F	1.073781000	-3.213151000	0.310456000
F	3.421409000	-1.161776000	-0.597008000
F	-3.182201000	-1.322120000	1.292077000
F	1.276422000	2.997169000	-1.759126000
F	-0.454656000	-0.557337000	2.586652000
F	0.318470000	0.854515000	-0.685604000
F	3.126176000	-2.615914000	1.155226000

$AlPAs_2F_{19}^-$

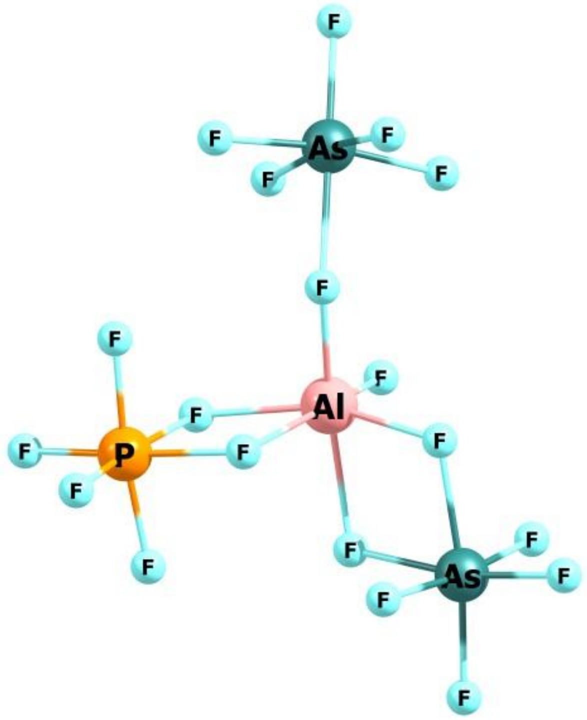

0.2

As	2.952833000	-1.147917000	0.143347000
As	-3.120260000	-0.950810000	0.114866000
P	0.173603000	2.810319000	0.057897000
Al	0.228532000	-0.067624000	-0.546110000
F	-0.030518000	-0.933463000	-1.964540000
F	4.529946000	-0.773801000	-0.440522000
F	-1.286371000	-0.345478000	0.410006000
F	3.378603000	-2.412510000	1.234781000
F	-0.273964000	4.047645000	-0.873772000
F	2.663913000	-2.271181000	-1.134281000
F	-4.748117000	-1.485872000	-0.131857000
F	-3.586458000	0.546943000	0.859358000
F	1.695805000	2.875789000	-0.498779000
F	-2.408863000	-2.373148000	-0.580087000
F	-1.339910000	2.555843000	0.558465000
F	-2.972670000	-1.666953000	1.693423000
F	3.060705000	0.037354000	1.397385000
F	-0.159669000	1.662345000	-1.257673000
F	1.141410000	-1.315751000	0.586051000
F	2.170997000	0.146886000	-0.910617000
F	0.607257000	1.299964000	0.836800000
F	-2.999148000	-0.138209000	-1.416408000
F	0.551511000	3.652746000	1.377815000

$AlPAs_2F_{19}^-$

3.2

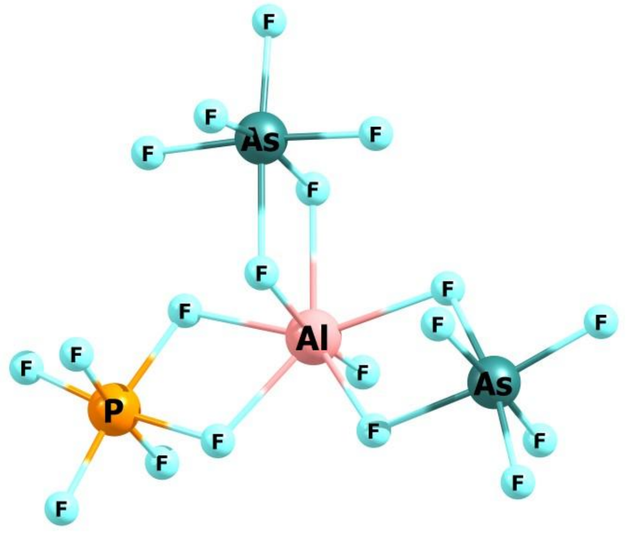

As	-2.861034000	-0.803781000	0.150914000
As	0.579563000	2.402550000	0.009313000
P	2.851062000	-1.636346000	0.200495000
Al	0.138339000	-0.584044000	-0.567397000
F	-1.759200000	0.168643000	-0.921723000
F	-4.269815000	0.033259000	-0.393086000
F	0.300434000	0.700975000	0.805890000
F	-3.642421000	-1.870842000	1.265183000
F	4.356742000	-1.278245000	-0.265193000
F	-2.578947000	0.356948000	1.407019000
F	0.815594000	3.806981000	-0.964041000
F	2.271428000	2.071156000	0.050756000
F	2.669788000	-2.715853000	-0.998213000
F	-1.134458000	2.593642000	-0.023440000
F	2.885959000	-0.518028000	1.380325000
F	0.657569000	3.135134000	1.569712000
F	-2.989213000	-1.975423000	-1.116970000
F	2.179100000	-0.440489000	-0.840981000
F	-1.201616000	-1.517172000	0.556438000
F	0.018009000	-1.472766000	-1.993406000
F	1.147379000	-1.852148000	0.565946000
F	0.441487000	1.289763000	-1.433992000
F	3.245982000	-2.806828000	1.247691000

4.1

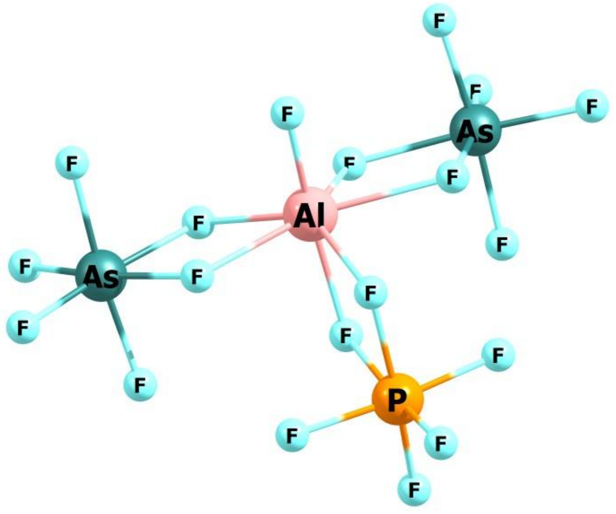

	As	-2.936401000	-0.947052000	0.153075000
	P	-0.001478000	2.664481000	0.011184000
	As	2.937432000	-0.945743000	0.153161000
	Al	0.001078000	-0.281193000	-0.566693000
	F	-2.002010000	0.168523000	-0.933610000
	F	-4.461086000	-0.351706000	-0.397537000
	F	-0.001244000	1.022564000	0.788278000
	F	-3.539102000	-2.109021000	1.283869000
	F	4.462018000	-0.348576000	-0.395380000
	F	-2.840985000	0.259167000	1.394183000
	F	-0.001384000	4.012254000	-0.875893000
	F	-0.002832000	3.348832000	1.474131000
	F	2.881361000	-2.140323000	-1.098685000
	F	-0.000030000	1.645009000	-1.384080000
	F	2.840230000	0.258673000	1.395974000
	F	-1.615038000	2.584992000	0.015022000
	F	-2.878652000	-2.143853000	-1.096754000
	F	2.002431000	0.171312000	-0.931861000
	F	-1.184605000	-1.386261000	0.567180000
	F	0.001548000	-1.176516000	-1.993668000
	F	1.185286000	-1.386560000	0.565665000
	F	1.612168000	2.586439000	0.017429000
	F	3.539053000	-2.109336000	1.282784000

Table S2. The vertical electron detachment energies (VDE in eV) calculated with OVGF(full) method using the aug-cc-pVDZ basis set characterizing the most stable $(X_nF_{(3n+1)})^-$, $(Y_nF_{(5n+1)})^-$ and $(X_nY_{n'}F_{\{(3n+5n')+1\}})^-$ ($n+n'$=1-4 and X=B and/or Al, Y=P and/or As) anions.

Species	VDE (PS)
BF_4^-	9.09 (0.925)
AlF_4^-	9.90 (0.932)
PF_6^-	9.65 (0.916)
AsF_6^-	10.11 (0.915)
$B_2F_7^-$	10.70 (0.923)
$B_3F_{10}^-$	11.68 (0.921)
$B_4F_{13}^-$	12.07 (0.919)
$Al_2F_7^-$	11.46 (0.930)
$Al_3F_{10}^-$	10.87 (0.926)
$Al_4F_{13}^-$	12.37 (0.927)
$P_2F_{11}^-$	11.05 (0.913)
$P_3F_{16}^-$	11.83 (0.911)
$P_4F_{21}^-$	11.85 (0.910)
$As_2F_{11}^-$	11.49 (0.911)
$As_3F_{16}^-$	12.25 (0.911)
$As_4F_{21}^-$	12.41 (0.909)
BPF_9^-	10.69 (0.914)
$AlPF_9^-$	11.21 (0.929)
$BAsF_9^-$	11.10 (0.914)
$AlAsF_9^-$	11.30 (0.929)
$Al_2AsF_{12}^-$	11.07 (0.925)
$Al_2PF_{12}^-$	11.20 (0.923)
$B_2AsF_{12}^-$	11.76 (0.913)
$B_2PF_{12}^-$	11.48 (0.914)
$AlAs_2F_{14}^-$	12.37 (0.925)
$BAs_2F_{14}^-$	12.13 (0.912)
$AlP_2F_{14}^-$	12.23 (0.926)
$BP_2F_{14}^-$	11.78 (0.913)
$BAlAsF_{12}^-$	12.06 (0.926)
$BAlPF_{12}^-$	12.11 (0.927)
$AlPAsF_{14}^-$	12.28 (0.925)
$BPAsF_{14}^-$	11.90 (0.913)
$B_3PF_{15}^-$	11.49 (0.913)
$B_3AsF_{15}^-$	11.42 (0.911)
$Al_3PF_{15}^-$	11.64 (0.928)
$Al_3AsF_{15}^-$	11.65 (0.928)
$B_2AlPF_{15}^-$	12.14 (0.926)
$B_2AlAsF_{15}^-$	12.28 (0.926)
$BAl_2PF_{15}^-$	11.49 (0.928)
$BAl_2AsF_{15}^-$	11.50 (0.928)
$BP_3F_{19}^-$	12.20 (0.912)

$AlP_3F_{19}^-$	12.08 (0.922)
$BAs_3F_{19}^-$	12.21 (0.910)
$AlAs_3F_{19}^-$	12.18 (0.922)
$BP_2AsF_{19}^-$	12.17 (0.911)
$AlP_2AsF_{19}^-$	12.18 (0.922)
$BPAs_2F_{19}^-$	12.28 (0.911)
$AlPAs_2F_{19}^-$	12.10 (0.922)
$B_2P_2F_{17}^-$	11.82 (0.912)
$B_2As_2F_{17}^-$	11.85 (0.910)
$Al_2P_2F_{17}^-$	11.91 (0.925)
$Al_2As_2F_{17}^-$	11.95 (0.925)
$B_2PAsF_{17}^-$	11.70 (0.911)
$Al_2PAsF_{17}^-$	11.93 (0.925)
$BAlP_2F_{17}^-$	11.93 (0.923)
$BAlAs_2F_{17}^-$	12.10 (0.923)
$BAlPAsF_{17}^-$	12.04 (0.924)

Table S3. NBO atomic charges, Wiberg bond indices, and bonding orbital compositions for the studied $(X_nY_{n'}F_{\{(3n+5n')+1\}})^-$ (n+n'=1-4 and X=B and/or Al, Y=P and/or As) anions calculated at the MP2/aug-cc-pvdz level.

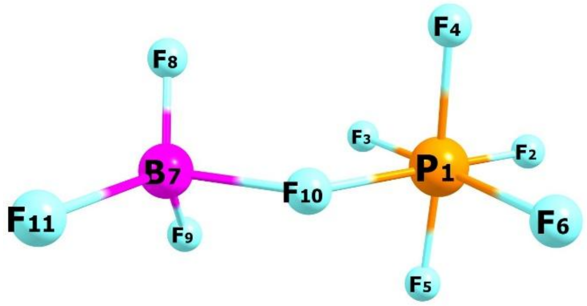

NBO charges	Wiberg indices
**P_1** 2.735	**P_1-F_2** 0.529
**F_2** -0.606	**P_1-F_3** 0.538
**F_3** -0.594	**P_1-F_4** 0.533
**F_4** -0.600	**P_1-F_5** 0.533
**F_5** -0.600	**P_1-F_6** 0.530
**F_6** -0.602	**P_1-F_{10}** 0.355
**B_7** 1.550	**B_7-F_{10}** 0.262
**F_8** -0.572	**B_7-F_8** 0.640
**F_9** -0.573	**B_7-F_{11}** 0.625
**F_{10}** -0.557	**B_7-F_9** 0.640
**F_{11}** -0.582	

Natural bond orbital (BD, 2c-2e): composition (coefficients), hybridization, [occupancy (e)]
**P_1-F_2** 0.3559$sp^{2.71}d^{1.71}$(**P**)+0.9345$sp^{2.81}$(**F**) [1.902]
**P_1-F_3** 0.3552$sp^{2.89}d^{1.91}$(**P**)+0.9348$sp^{2.97}$(**F**) [1.894]
**P_1-F_4** 0.3558$sp^{2.90}d^{1.88}$(**P**)+0.9346$sp^{2.99}$(**F**) [1.896]
**P_1-F_5** 0.3558$sp^{2.90}d^{1.88}$(**P**)+0.9346$sp^{2.99}$(**F**) [1.896]
**P_1-F_6** 0.3563$sp^{2.91}d^{1.85}$(**P**)+0.9344$sp^{2.99}$(**F**) [1.898]
**P_1-F_{10}** 0.2992$sp^{3.93}d^{2.78}$(**P**)+0.9542$sp^{2.47}$(**F**) [1.906]
**B_7-F_{10}** 0.2798$sp^{5.30}$(**B**)+0.9600$sp^{2.01}$(**F**) [1.962]
**B_7-F_8** 0.3847$sp^{2.53}$(**B**)+0.9230$sp^{1.66}$(**F**) [1.970]
**B_7-F_{11}** 0.3836$sp^{2.55}$(**B**)+0.9235$sp^{1.68}$(**F**) [1.971]
**B_7-F_9** 0.3847$sp^{2.53}$(**B**)+0.9230$sp^{1.66}$(**F**) [1.970]

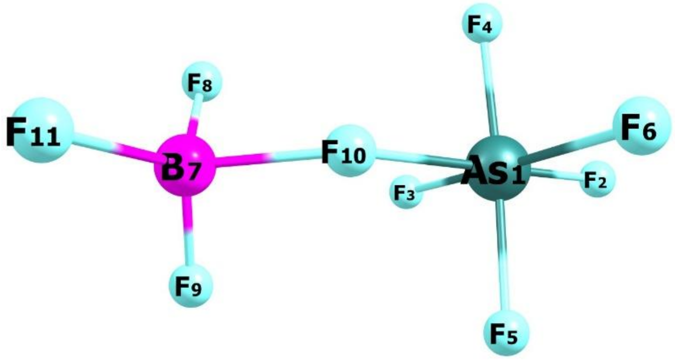

NBO charges	Wiberg indices
**As_1** 2.858	**As_1-F_2** 0.488
**F_2** -0.629	**As_1-F_3** 0.499

**F_3** -0.615	**As_1-F_4** 0.494
**F_4** -0.621	**As_1-F_5** 0.494
**F_5** -0.621	**As_1-F_6** 0.491
**F_6** -0.623	**As_1-F_{10}** 0.327
**B_7** 1.548	**B_7-F_{10}** 0.256
**F_8** -0.571	**B_7-F_8** 0.642
**F_9** -0.571	**B_7-F_{11}** 0.628
**F_{10}** -0.576	**B_7-F_9** 0.642
**F_{11}** -0.580	

Natural bond orbital (BD, 2c-2e): composition (coefficients), hybridization, [occupancy (e)]

As_1-F_2 0.3364sp$^{2.82}$d$^{1.70}$(**As**)+0.9417sp$^{3.79}$(**F**) [1.887]
As_1-F_3 0.3363sp$^{2.86}$d$^{1.89}$(**As**)+0.9418sp$^{4.08}$(**F**) [1.877]
As_1-F_4 0.3368sp$^{2.87}$d$^{1.84}$(**As**)+0.9416sp$^{4.08}$(**F**) [1.880]
As_1-F_5 0.3368sp$^{2.87}$d$^{1.84}$(**As**)+0.9416sp$^{4.08}$(**F**) [1.880]
As_1-F_6 0.3375sp$^{2.89}$d$^{1.80}$(**As**)+0.9413sp$^{4.06}$(**F**) [1.882]
As_1-F_{10} 0.2775sp$^{3.92}$d$^{2.83}$(**As**)+0.9607sp$^{2.82}$(**F**) [1.901]
B_7-F_{10} 0.2765sp$^{5.50}$(**B**)+0.9610sp$^{1.97}$(**F**) [1.962]
B_7-F_8 0.3851sp$^{2.52}$(**B**)+0.9229sp$^{1.67}$(**F**) [1.970]
B_7-F_{11} 0.3842sp$^{2.51}$(**B**)+0.9233sp$^{1.69}$(**F**) [1.971]
B_7-F_9 0.3851sp$^{2.52}$(**B**)+0.9229sp$^{1.67}$(**F**) [1.970]

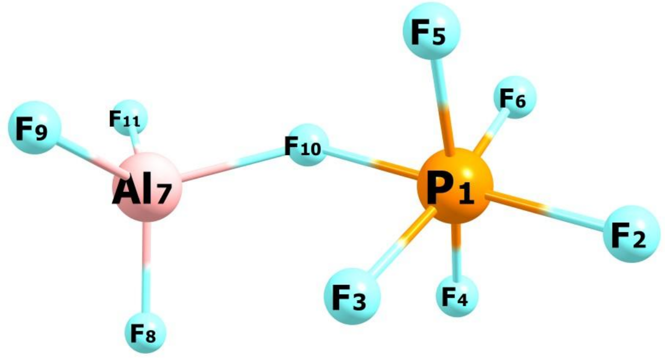

NBO charges	Wiberg indices
**P_1** 2.734	**P_1-F_2** 0.539
**F_2** -0.599	**P_1-F_3** 0.539
**F_3** -0.593	**P_1-F_4** 0.543
**F_4** -0.593	**P_1-F_5** 0.543
**F_5** -0.593	**P_1-F_6** 0.540
**F_6** -0.596	**P_1-F_{10}** 0.321
**Al_7** 2.215	**Al_7-F_{10}** 0.186
**F_8** -0.777	**Al_7-F_8** 0.351
**F_9** -0.777	**Al_7-F_{11}** 0.341
**F_{10}** -0.639	**Al_7-F_9** 0.351
**F_{11}** -0.782	

Natural bond orbital (BD, 2c-2e): composition (coefficients), hybridization, [occupancy (e)]

P_1-F_2 0.3587sp$^{2.66}$d$^{1.61}$(**P**)+0.9334sp$^{2.78}$(**F**) [1.903]
P_1-F_3 0.3555sp$^{2.89}$d$^{1.91}$(**P**)+0.9347sp$^{3.05}$(**F**) [1.894]
P_1-F_4 0.3581sp$^{2.86}$d$^{1.83}$(**P**)+0.9337sp$^{3.02}$(**F**) [1.896]
P_1-F_5 0.3581sp$^{2.86}$d$^{1.83}$(**P**)+0.9337sp$^{3.02}$(**F**) [1.896]
P_1-F_6 0.3587sp$^{2.86}$d$^{1.78}$(**P**)+0.9334sp$^{3.01}$(**F**) [1.898]

P_1-F_{10} 0.2834sp$^{4.33}$d$^{3.23}$(**P**)+0.9590sp$^{2.35}$(**F**) [1.913]
Al_7-F_8 0.2668sp$^{2.62}$(**Al**)+0.9637sp$^{2.07}$(**F**) [1.969]
Al_7-F_9 0.2668sp$^{2.62}$(**Al**)+0.9638sp$^{2.07}$(**F**) [1.969]
Al_7-F_{10} 0.2124sp$^{4.22}$(**Al**)+0.9772sp$^{1.96}$(**F**) [1.964]
Al_7-F_{11} 0.2647sp$^{2.67}$(**Al**)+0.9643sp$^{2.07}$(**F**) [1.969]

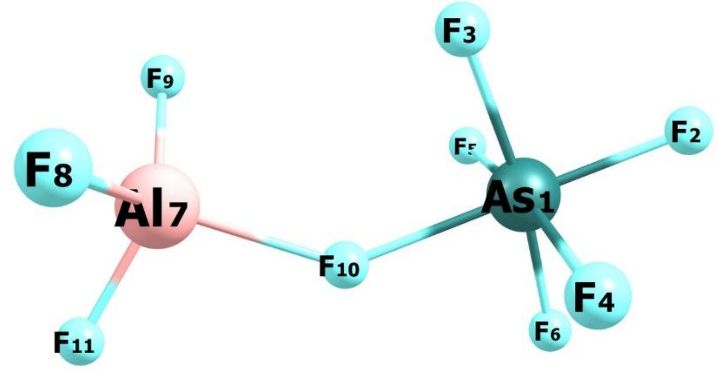

NBO charges	Wiberg indices
**As_1** 2.859	**As_1-F_2** 0.498
**F_2** -0.622	**As_1-F_3** 0.502
**F_3** -0.612	**As_1-F_4** 0.504
**F_4** -0.613	**As_1-F_5** 0.504
**F_5** -0.613	**As_1-F_6** 0.500
**F_6** -0.616	**As_1-F_{10}** 0.288
**Al_7** 2.216	**Al_7-F_{10}** 0.183
**F_8** -0.776	**Al_7-F_8** 0.352
**F_9** -0.776	**Al_7-F_{11}** 0.343
**F_{10}** -0.665	**Al_7-F_9** 0.352
**F_{11}** -0.781	

Natural bond orbital (BD, 2c-2e): composition (coefficients), hybridization, [occupancy (e)]

As_1-F_2 0.3398sp$^{2.73}$d$^{1.59}$(**As**)+0.9405sp$^{3.77}$(**F**) [1.887]
As_1-F_3 0.3372sp$^{2.86}$d$^{1.87}$(**As**)+0.9414sp$^{4.18}$(**F**) [1.876]
As_1-F_4 0.3398sp$^{2.84}$d$^{1.79}$(**As**)+0.9405sp$^{4.16}$(**F**) [1.879]
As_1-F_5 0.3398sp$^{2.84}$d$^{1.79}$(**As**)+0.9405sp$^{4.16}$(**F**) [1.879]
As_1-F_6 0.3407sp$^{2.86}$d$^{1.74}$(**As**)+0.9402sp$^{4.12}$(**F**) [1.881]
As_1-F_{10} 0.2578sp$^{4.37}$d$^{3.12}$(**As**)+0.9662sp$^{2.52}$(**F**) [1.911]
Al_7-F_8 0.2674sp$^{2.62}$(**Al**)+0.9636sp$^{2.10}$(**F**) [1.969]
Al_7-F_9 0.2673sp$^{2.62}$(**Al**)+0.9636sp$^{2.10}$(**F**) [1.969]
Al_7-F_{10} 0.2103sp$^{4.35}$(**Al**)+0.9776sp$^{1.85}$(**F**) [1.964]
Al_7-F_{11} 0.2657sp$^{2.62}$(**Al**)+0.9641sp$^{2.10}$(**F**) [1.970]

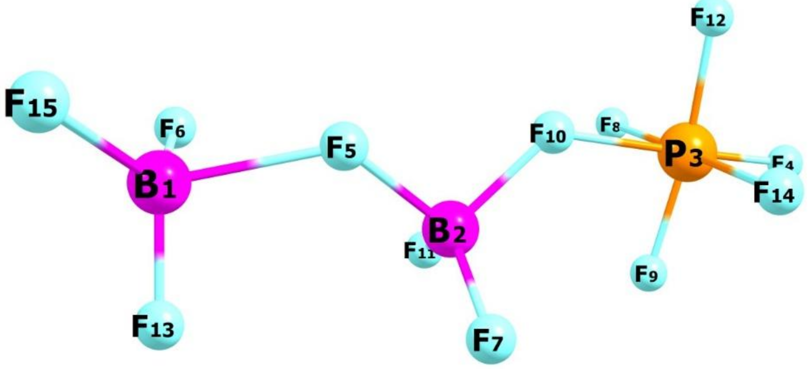

NBO charges	Wiberg indices
**B_1** 1.555	**B_1-F_5** 0.214
**B_2** 1.547	**B_1-F_6** 0.653
**P_3** 2.739	**B_1-F_{13}** 0.651
**F_4** -0.596	**B_1-F_{15}** 0.647
**F_5** -0.537	**B_2-F_5** 0.469
**F_6** -0.564	**B_2-F_7** 0.653
**F_7** -0.563	**B_2-F_{10}** 0.372
**F_8** -0.588	**B_2-F_{11}** 0.654
**F_9** -0.582	**P_3-F_4** 0.544
**F_{10}** -0.537	**P_3-F_8** 0.548
**F_{11}** -0.561	**P_3-F_9** 0.549
**F_{12}** -0.591	**P_3-F_{10}** 0.283
**F_{13}** -0.562	**P_3-F_{12}** 0.544
**F_{14}** -0.588	**P_3-F_{14}** 0.547
**F_{15}** -0.569	

Natural bond orbital (BD, 2c-2e): composition (coefficients), hybridization, [occupancy (e)]
**B_1-F_5** $0.2544sp^{6.00}$(**B**)+$0.9671sp^{3.57}$(**F**) [1.954]
**B_1-F_6** $0.3862sp^{2.45}$(**B**)+$0.9224sp^{1.67}$(**F**) [1.970]
**B_1-F_{13}** $0.3858sp^{2.49}$(**B**)+$0.9226sp^{1.68}$(**F**) [1.968]
**B_1-F_{15}** $0.3857sp^{2.45}$(**B**)+$0.9226sp^{1.68}$(**F**) [1.970]
**B_2-F_5** $0.3554sp^{3.20}$(**B**)+$0.9347sp^{1.69}$(**F**) [1.968]
**B_2-F_7** $0.3861sp^{2.53}$(**B**)+$0.9224sp^{1.55}$(**F**) [1.971]
**B_2-F_{10}** $0.3260sp^{3.92}$(**B**)+$0.9454sp^{1.70}$(**F**) [1.966]
**B_2-F_{11}** $0.3864sp^{2.55}$(**B**)+$0.9223sp^{1.56}$(**F**) [1.971]
**P_3-F_4** $0.3607sp^{2.60}d^{1.53}$(**P**)+$0.9327sp^{2.75}$(**F**) [1.905]
**P_3-F_8** $0.3585sp^{2.86}d^{1.82}$(**P**)+$0.9335sp^{3.03}$(**F**) [1.894]
**P_3-F_9** $0.3573sp^{2.86}d^{1.86}$(**P**)+$0.9340sp^{3.02}$(**F**) [1.892]
**P_3-F_{10}** $0.2677sp^{4.38}d^{3.25}$(**P**)+$0.9635sp^{3.15}$(**F**) [1.909]
**P_3-F_{12}** $0.3586sp^{2.88}d^{1.80}$(**P**)+$0.9335sp^{3.02}$(**F**) [1.896]
**P_3-F_{14}** $0.3583sp^{2.86}d^{1.83}$(**P**)+$0.9336sp^{3.03}$(**F**) [1.894]

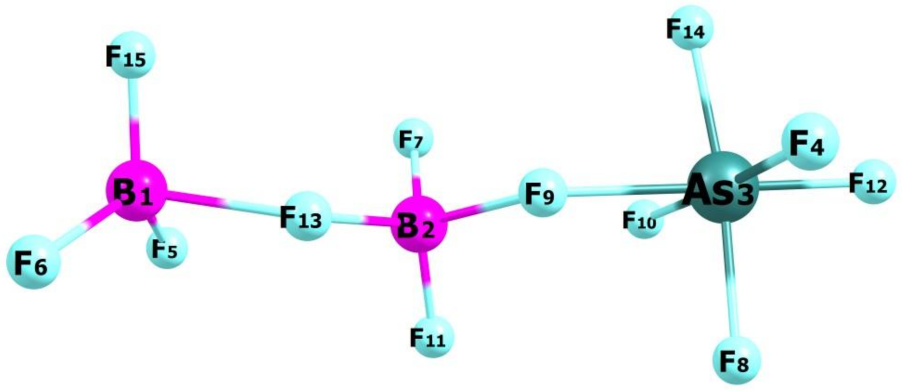

NBO charges	Wiberg indices
**B_1** 1.559	**B_1-F_5** 0.657
**B_2** 1.543	**B_1-F_6** 0.653
**As_3** 2.858	**B_1-F_{13}** 0.190
**F_4** -0.613	**B_1-F_{15}** 0.660
**F_5** -0.559	**B_2-F_7** 0.656

**F_6** -0.564	**B_2-F_9** 0.358
**F_7** -0.561	**B_2-F_{11}** 0.655
**F_8** -0.611	**B_2-F_{13}** 0.486
**F_9** -0.552	**As_3-F_4** 0.502
**F_{10}** -0.605	**As_3-F_8** 0.506
**F_{11}** -0.562	**As_3-F_9** 0.268
**F_{12}** -0.621	**As_3-F_{10}** 0.508
**F_{13}** -0.542	**As_3-F_{12}** 0.500
**F_{14}** -0.610	**As_3-F_{14}** 0.506
**F_{15}** -0.560	
Natural bond orbital (BD, 2c-2e): composition (coefficients), hybridization, [occupancy (e)]	
**B_1-F_5** $0.3859sp^{2.46}$(**B**)+$0.9225sp^{1.69}$(**F**) [1.968]	
**B_1-F_6** $0.3860sp^{2.41}$(**B**)+$0.9225sp^{1.68}$(**F**) [1.970]	
**B_1-F_{13}** $0.2403sp^{6.46}$(**B**)+$0.9707sp^{4.10}$(**F**) [1.951]	
**B_1-F_{15}** $0.3864sp^{2.41}$(**B**)+$0.9223sp^{1.68}$(**F**) [1.970]	
**B_2-F_7** $0.3868sp^{2.55}$(**B**)+$0.9222sp^{1.57}$(**F**) [1.971]	
**B_2-F_9** $0.3205sp^{4.08}$(**B**)+$0.9472sp^{1.69}$(**F**) [1.966]	
**B_2-F_{11}** $0.3866sp^{2.52}$(**B**)+$0.9223sp^{1.57}$(**F**) [1.971]	
**B_2-F_{13}** $0.3595sp^{3.08}$(**B**)+$0.9331sp^{1.66}$(**F**) [1.969]	
**As_3-F_4** $0.3403sp^{2.86}d^{1.76}$(**As**)+$0.9403sp^{4.14}$(**F**) [1.880]	
**As_3-F_8** $0.3398sp^{2.84}d^{1.79}$(**As**)+$0.9405sp^{4.16}$(**F**) [1.877]	
**As_3-F_9** $0.2511sp^{4.22}d^{3.12}$(**As**)+$0.9680sp^{3.38}$(**F**) [1.905]	
**As_3-F_{10}** $0.3389sp^{2.84}d^{1.82}$(**As**)+$0.9408sp^{4.16}$(**F**) [1.874]	
**As_3-F_{12}** $0.3417sp^{2.76}d^{1.52}$(**As**)+$0.9398sp^{3.74}$(**F**) [1.889]	
**As_3-F_{14}** $0.3400sp^{2.84}d^{1.79}$(**As**)+$0.9404sp^{4.16}$(**F**) [1.877]	

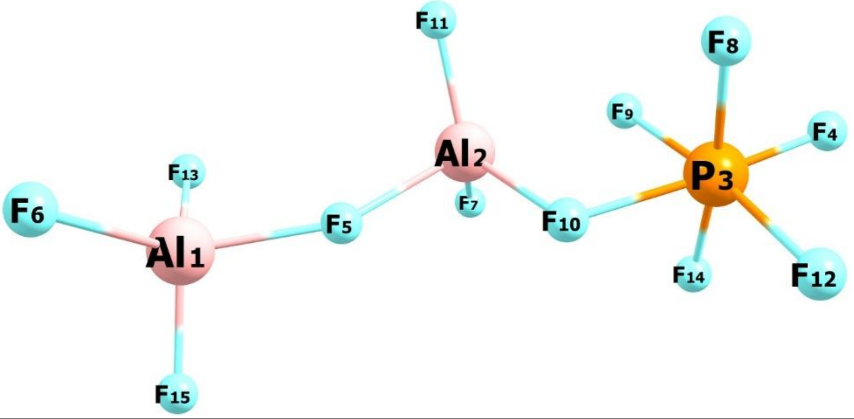

NBO charges	Wiberg indices
**Al_1** 2.21297	**Al_1-F_5** 0.185
**Al_2** 2.21223	**Al_1-F_6** 0.352
**P_3** 2.72710	**Al_1-F_{13}** 0.353
**F_4** -0.58486	**Al_1-F_{15}** 0.352
**F_5** -0.75305	**Al_2-F_5** 0.202
**F_6** -0.77642	**Al_2-F_7** 0.375
**F_7** -0.75911	**Al_2-F_{10}** 0.191
**F_8** -0.58340	**Al_2-F_{11}** 0.375
**F_9** -0.59362	**P_3-F_4** 0.558
**F_{10}** -0.62442	**P_3-F_8** 0.554
**F_{11}** -0.75910	**P_3-F_9** 0.469

**F_{12}** -0.58343	**P_3-F_{10}** 0.336
**F_{13}** -0.77506	**P_3-F_{12}** 0.557
**F_{14}** -0.58341	**P_3-F_{14}** 0.554
**F_{15}** -0.77642	

Natural bond orbital (BD, 2c-2e): composition (coefficients), hybridization, [occupancy (e)]
**Al_1-F_5** 0.2098sp$^{4.38}$(**Al**)+0.9777sp$^{1.76}$(**F**) [1.970]
**Al_1-F_6** 0.2689sp$^{2.57}$(**Al**)+0.9632sp$^{2.17}$(**F**) [1.970]
**Al_1-F_{13}** 0.2683sp$^{2.70}$(**Al**)+0.9633sp$^{2.18}$(**F**) [1.968]
**Al_1-F_{15}** 0.2689sp$^{2.57}$(**Al**)+0.9632sp$^{2.17}$(**F**) [1.970]
**Al_2-F_5** 0.2148sp$^{5.24}$(**Al**)+0.9767sp$^{1.35}$(**F**) [1.970]
**Al_2-F_7** 0.2722sp$^{2.19}$(**Al**)+0.9622sp$^{1.87}$(**F**) [1.970]
**Al_2-F_{10}** 0.2172sp$^{3.39}$(**Al**)+0.9761sp$^{2.39}$(**F**) [1.963]
**Al_2-F_{11}** 0.2722sp$^{2.19}$(**Al**)+0.9622sp$^{1.87}$(**F**) [1.970]
**P_3-F_4** 0.3641sp$^{2.62}$d$^{1.55}$(**P**)+0.9313sp$^{2.74}$(**F**) [1.904]
**P_3-F_8** 0.3607sp$^{2.84}$d$^{1.82}$(**P**)+0.9327sp$^{3.03}$(**F**) [1.894]
**P_3-F_9** 0.3377sp$^{3.22}$d$^{2.30}$(**P**)+0.9413sp$^{3.32}$(**F**) [1.894]
**P_3-F_{10}** 0.2901sp$^{4.08}$d$^{3.19}$(**P**)+0.9570sp$^{3.30}$(**F**) [1.906]
**P_3-F_{12}** 0.3629sp$^{2.76}$d$^{1.67}$(**P**)+0.9318sp$^{2.89}$(**F**) [1.900]
**P_3-F_{14}** 0.3607sp$^{2.84}$d$^{1.82}$(**P**)+0.9327sp$^{3.03}$(**F**) [1.894]

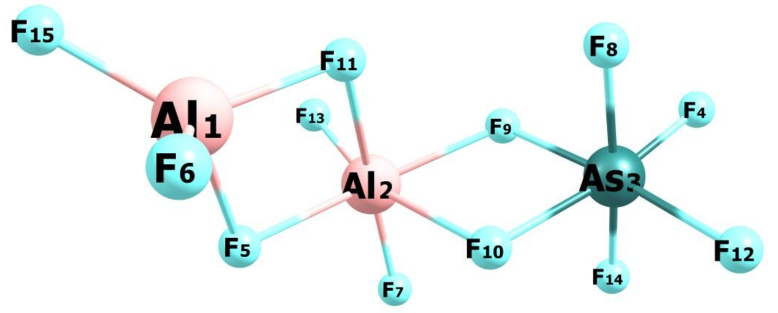

NBO charges	Wiberg indices
**Al_1** 2.213	**Al_1-F_5** 0.240
**Al_2** 2.160	**Al_1-F_6** 0.371
**As_3** 2.852	**Al_1-F_{11}** 0.255
**F_4** -0.611	**Al_1-F_{15}** 0.370
**F_5** -0.732	**Al_2-F_5** 0.175
**F_6** -0.764	**Al_2-F_7** 0.350
**F_7** -0.773	**Al_2-F_9** 0.157
**F_8** -0.606	**Al_2-F_{10}** 0.103
**F_9** -0.633	**Al_2-F_{11}** 0.157
**F_{10}** -0.624	**Al_2-F_{13}** 0.346
**F_{11}** -0.732	**As_3-F_4** 0.509
**F_{12}** -0.611	**As_3-F_8** 0.511
**F_{13}** -0.776	**As_3-F_9** 0.346
**F_{14}** -0.600	**As_3-F_{10}** 0.401
**F_{15}** -0.764	**As_3-F_{12}** 0.511
	**As_3-F_{14}** 0.519

Natural bond orbital (BD, 2c-2e): composition (coefficients), hybridization, [occupancy (e)]
**Al_1-F_5** 0.2333sp$^{3.57}$(**Al**)+0.9724sp$^{2.92}$(**F**) [1.964]
**Al_1-F_6** 0.2729sp$^{2.52}$(**Al**)+0.9621sp$^{2.12}$(**F**) [1.969]

**Al_1-F_{11}** $0.2395sp^{3.36}$(**Al**)+$0.9709sp^{2.78}$(**F**) [1.964]
**Al_1-F_{15}** $0.2727sp^{2.51}$(**Al**)+$0.9621sp^{2.12}$(**F**) [1.969]
**Al_2-F_5** $0.1837sp^{2.77}d^{1.35}$(**Al**)+$0.9830sp^{2.61}$(**F**) [1.947]
**Al_2-F_7** $0.2482sp^{1.98}d^{0.72}$(**Al**)+$0.9687sp^{1.52}$(**F**) [1.954]
**Al_2-F_9** $0.1837sp^{2.93}d^{1.07}$(**Al**)+$0.9830sp^{2.18}$(**F**) [1.950]
**Al_2-F_{11}** $0.1714sp^{3.64}d^{2.41}$(**Al**)+$0.9852sp^{3.14}$(**F**) [1.943]
**Al_2-F_{13}** $0.2581sp^{3.76}$(**Al**)+$0.9661sp^{1.51}$(**F**) [1.966]
**As_3-F_4** $0.3432sp^{2.81}d^{1.69}$(**As**)+$0.9393sp^{3.91}$(**F**) [1.884]
**As_3-F_8** $0.3415sp^{2.82}d^{1.81}$(**As**)+$0.9399sp^{4.15}$(**F**) [1.876]
**As_3-F_9** $0.2835sp^{3.69}d^{2.83}$(**As**)+$0.9590sp^{4.02}$(**F**) [1.893]
**As_3-F_{10}** $0.3050sp^{3.35}d^{2.47}$(**As**)+$0.9524sp^{4.25}$(**F**) [1.885]
**As_3-F_{12}** $0.3442sp^{2.76}d^{1.64}$(**As**)+$0.9389sp^{3.83}$(**F**) [1.886]
**As_3-F_{14}** $0.3438sp^{2.78}d^{1.77}$(**As**)+$0.9390sp^{4.17}$(**F**) [1.876]

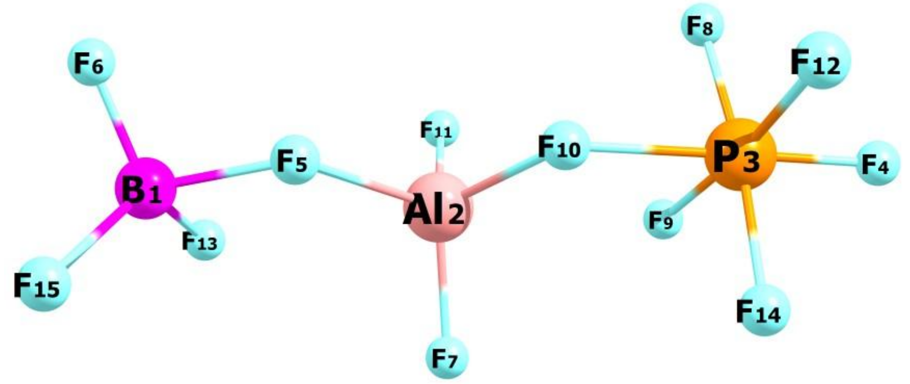

NBO charges	Wiberg indices
**B_1** 1.532	**B_1-F_6** 0.638
**Al_2** 2.224	**B_1-F_5** 0.304
**P_3** 2.731	**B_1-F_{13}** 0.613
**F_4** -0.593	**B_1-F_{15}** 0.638
**F_5** -0.650	**Al_2-F_5** 0.226
**F_6** -0.574	**Al_2-F_7** 0.372
**F_7** -0.761	**Al_2-F_{10}** 0.201
**F_8** -0.588	**Al_2-F_{11}** 0.372
**F_9** -0.591	**P_3-F_{10}** 0.306
**F_{10}** -0.638	**P_3-F_9** 0.526
**F_{11}** -0.761	**P_3-F_8** 0.549
**F_{12}** -0.590	**P_3-F_4** 0.548
**F_{13}** -0.579	**P_3-F_{14}** 0.549
**F_{14}** -0.588	**P_3-F_{12}** 0.548
**F_{15}** -0.574	

Natural bond orbital (BD, 2c-2e): composition (coefficients), hybridization, [occupancy (e)]
**B_1-F_5** $0.2980sp^{4.91}$(**B**)+$0.9546sp^{2.29}$(**F**) [1.966]
**B_1-F_6** $0.3870sp^{2.53}$(**B**)+$0.9221sp^{1.69}$(**F**) [1.971]
**B_1-F_{13}** $0.3839sp^{2.69}$(**B**)+$0.9234sp^{1.77}$(**F**) [1.969]
**B_1-F_{15}** $0.3870sp^{2.53}$(**B**)+$0.9221sp^{1.69}$(**F**) [1.971]
**Al_2-F_5** $0.2284sp^{3.57}$(**Al**)+$0.9736sp^{2.26}$(**F**) [1.965]
**Al_2-F_7** $0.2691sp^{2.34}$(**Al**)+$0.9631sp^{1.88}$(**F**) [1.969]
**Al_2-F_{10}** $0.2186sp^{3.99}$(**Al**)+$0.9758sp^{1.96}$(**F**) [1.964]

**Al_2-F_{11}** 0.2691sp$^{2.34}$(**Al**)+0.9631sp$^{1.88}$(**F**) [1.969]
**P_3-F_4** 0.3618sp$^{2.64}$d$^{1.54}$(**P**)+0.9323sp$^{2.75}$(**F**) [1.904]
**P_3-F_8** 0.3592sp$^{2.84}$d$^{1.81}$(**P**)+0.9333sp$^{3.04}$(**F**) [1.895]
**P_3-F_9** 0.3527sp$^{2.95}$d$^{1.97}$(**P**)+0.9357sp$^{3.13}$(**F**) [1.893]
**P_3-F_{10}** 0.2773sp$^{4.37}$d$^{3.41}$(**P**)+0.9608sp$^{2.88}$(**F**) [1.912]
**P_3-F_{12}** 0.3602sp$^{2.83}$d$^{1.75}$(**P**)+0.9329sp$^{2.98}$(**F**) [1.898]
**P_3-F_{14}** 0.3592sp$^{2.84}$d$^{1.81}$(**P**)+0.9333sp$^{3.04}$(**F**) [1.895]

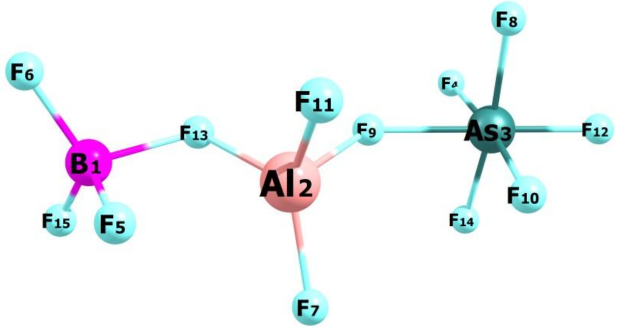

NBO charges	Wiberg indices
**B_1** 1.518	**B_1-F_5** 0.525
**Al_2** 2.207	**B_1-F_6** 0.650
**As_3** 2.859	**B_1-F_{13}** 0.380
**F_4** -0.615	**B_1-F_{15}** 0.650
**F_5** -0.586	**Al_2-F_7** 0.371
**F_6** -0.565	**Al_2-F_9** 0.186
**F_7** -0.761	**Al_2-F_{11}** 0.371
**F_8** -0.612	**Al_2-F_{13}** 0.199
**F_9** -0.663	**As_3-F_4** 0.502
**F_{10}** -0.612	**As_3-F_8** 0.506
**F_{11}** -0.761	**As_3-F_9** 0.284
**F_{12}** -0.620	**As_3-F_{10}** 0.498
**F_{13}** -0.613	**As_3-F_{12}** 0.501
**F_{14}** -0.612	**As_3-F_{14}** 0.506
**F_{15}** -0.565	

Natural bond orbital (BD, 2c-2e): composition (coefficients), hybridization, [occupancy (e)]
**B_1-F_5** 0.3698sp$^{3.09}$(**B**)+0.9291sp$^{1.98}$(**F**) [1.967]
**B_1-F_6** 0.3778sp$^{2.60}$d$^{0.17}$(**B**)+0.9259sp$^{1.67}$(**F**) [1.951]
**B_1-F_{13}** 0.2735sp$^{2.65}$d$^{0.82}$(**B**)+0.9619sp$^{2.44}$ (**F**) [1.902]
**B_1-F_{15}** 0.3778sp$^{2.60}$d$^{0.17}$(**B**)+0.9259sp$^{1.67}$ (**F**) [1.951]
**Al_2-F_7** 0.2706sp$^{2.14}$(**Al**)+0.9627sp$^{1.79}$ (**F**) [1.970]
**Al_2-F_9** 0.2092sp$^{5.91}$d$^{0.11}$(**Al**)+0.9779sp$^{1.67}$ (**F**) [1.963]
**Al_2-F_{11}** 0.2706sp$^{2.14}$ (**Al**)+0.9627sp$^{1.79}$ (**F**) [1.970]
**Al_2-F_{13}** 0.2182sp$^{3.24}$ (**Al**)+0.9759sp$^{3.04}$ (**F**) [1.960]
**As_3-F_4** 0.3407sp$^{2.86}$d$^{1.75}$(**As**)+0.9402sp$^{4.12}$ (**F**) [1.881]
**As_3-F_8** 0.3402sp$^{2.83}$d$^{1.79}$(**As**)+0.9403sp$^{4.16}$ (**F**) [1.878]
**As_3-F_9** 0.2568sp$^{4.30}$d$^{3.18}$(**As**)+0.9665sp$^{2.84}$ (**F**) [1.909]
**As_3-F_{10}** 0.3361sp$^{2.88}$d$^{1.89}$(**As**)+0.9418sp$^{4.18}$ (**F**) [1.876]
**As_3-F_{12}** 0.3412sp$^{2.74}$d$^{1.58}$(**As**)+0.9400sp$^{3.76}$ (**F**) [1.888]
**As_3-F_{14}** 0.3402sp$^{2.83}$d$^{1.79}$(**As**)+0.9403sp$^{4.16}$ (**F**) [1.878]

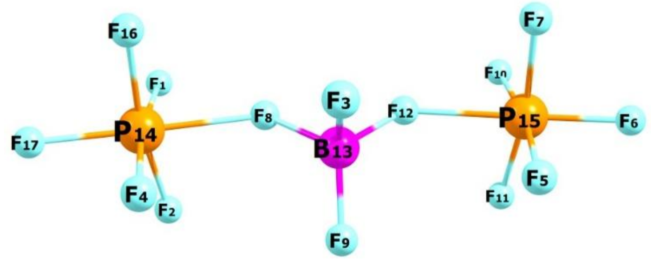

NBO charges	Wiberg indices
**F_1** -0.587	**F_1-P_{14}** 0.549
**F_2** -0.5847	**F_2-P_{14}** 0.553
**F_3** -0.556	**F_3-B_{13}** 0.663
**F_4** -0.580	**F_4-P_{14}** 0.552
**F_5** -0.579	**F_5-P_{15}** 0.552
**F_6** -0.593	**F_6-P_{15}** 0.550
**F_7** -0.584	**F_7-P_{15}** 0.553
**F_8** -0.535	**F_8-B_{13}** 0.404
**F_9** -0.556	**F_8-P_{14}** 0.257
**F_{10}** -0.587	**F_9-B_{13}** 0.663
**F_{11}** -0.584	**F_{10}-P_{15}** 0.550
**F_{12}** -0.535	**F_{11}-P_{15}** 0.553
**B_{13}** 1.554	**F_{12}-B_{13}** 0.405
**P_{14}** 2.741	**F_{12}-P_{15}** 0.256
**P_{15}** 2.741	**P_{14}-F_{16}** 0.553
**F_{16}** -0.584	**P_{14}-F_{17}** 0.549
**F_{17}** -0.593	

Natural bond orbital (BD, 2c-2e): composition (coefficients), hybridization, [occupancy (e)]
**F_1-P_{14}** 0.9331sp$^{3.04}$ (**F**)+0.3595sp$^{2.87}$d$^{1.78}$(**P**) [1.895]
**F_2-P_{14}** 0.9333sp$^{3.05}$ (**F**)+0.3592sp$^{2.85}$d$^{1.81}$(**P**) [1.894]
**F_3-B_{13}** 0.9226sp$^{1.51}$ (**F**)+0.3859sp$^{2.49}$ (**B**) [1.971]
**F_4-P_{14}** 0.9338sp$^{3.05}$ (**F**)+0.3577sp$^{2.86}$d$^{1.84}$(**P**) [1.892]
**F_5-P_{15}** 0.9338sp$^{3.05}$ (**F**)+0.3577sp$^{2.86}$d$^{1.84}$(**P**) [1.892]
**F_6-P_{15}** 0.9321sp$^{2.74}$ (**F**)+0.3623sp$^{2.55}$d$^{1.46}$(**P**) [1.906]
**F_7-P_{15}** 0.9333sp$^{3.05}$ (**F**)+0.3592sp$^{2.85}$d$^{1.81}$(**P**) [1.894]
**F_8-B_{13}** 0.9418sp$^{1.63}$ (**F**)+0.3362sp$^{3.62}$ (**B**) [1.967]
**F_8-P_{14}** 0.9670sp$^{3.53}$ (**F**)+0.2548sp$^{4.57}$d$^{3.42}$(**P**) [1.910]
**F_9-B_{13}** 0.9226sp$^{1.51}$ (**F**)+0.3859sp$^{2.49}$ (**B**) [1.971]
**F_{10}-P_{15}** 0.9331sp$^{3.04}$ (**F**)+0.3596sp$^{2.87}$d$^{1.78}$(**P**) [1.895]
**F_{11}-P_{15}** 0.9333sp$^{3.05}$ (**F**)+0.3592sp$^{2.85}$d$^{1.81}$(**P**) [1.894]
**F_{12}-B_{13}** 0.9417sp$^{1.63}$ (**F**)+0.3365sp$^{3.61}$ (**B**) [1.967]
**F_{12}-P_{15}** 0.9671sp$^{3.54}$ (**F**)+0.2544sp$^{4.57}$d$^{3.42}$(**P**) [1.910]
**P_{14}-F_{16}** 0.3592sp$^{2.85}$d$^{1.81}$(**P**)+0.9333sp$^{3.05}$ (**F**) [1.894]
**P_{14}-F_{17}** 0.3622sp$^{2.56}$d$^{1.46}$(**P**)+0.9321sp$^{2.74}$ (**F**) [1.906]

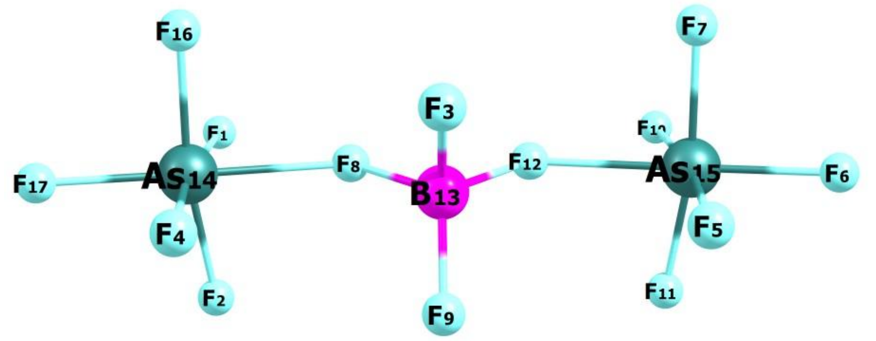

NBO charges	Wiberg indices
F_1 -0.608	F_1-As_{14} 0.509
F_2 -0.605	F_2-As_{14} 0.512
F_3 -0.552	F_3-B_{13} 0.670
F_4 -0.600	F_4-As_{14} 0.511
F_5 -0.601	F_5-As_{15} 0.511
F_6 -0.616	F_6-As_{15} 0.508
F_7 -0.6056	F_7-As_{15} 0.512
F_8 -0.545	F_{12}-As_{15} 0.239
F_9 -0.5525	F_8-B_{13} 0.402
F_{10} -0.608	F_8-As_{14} 0.238
F_{11} -0.605	F_9-B_{13} 0.670
F_{12} -0.545	F_{10}-As_{15} 0.509
B_{13} 1.546	F_{11}-As_{15} 0.512
As_{14} 2.858	F_{12}-B_{13} 0.401
As_{15} 2.858	As_{14}-F_{16} 0.512
F_{16} -0.605	As_{14}-F_{17} 0.508
F_{17} -0.615	

Natural bond orbital (BD, 2c-2e): composition (coefficients), hybridization, [occupancy (e)]
**F_1-As_{14}** $0.9397sp^{4.19}$ (**F**)+$0.3421sp^{2.85}d^{1.73}$(**As**) [1.878]
**F_2-As_{14}** $0.9399sp^{4.22}$ (**F**)+$0.3414sp^{2.83}d^{1.76}$(**As**) [1.876]
**F_3-B_{13}** $0.9220sp^{1.52}$(**F**)+$0.3872sp^{2.47}$ (**B**) [1.971]
**F_4-As_{14}** $0.9406sp^{4.22}$ (**F**)+$0.3396sp^{2.85}d^{1.81}$(**As**) [1.873]
**F_5-As_{15}** $0.9341sp^{4.22}$ (**F**)+$0.3571sp^{2.51}d^{1.17}$(**As**) [1.893]
**F_6-As_{15}** $0.9249sp^{3.73}$ (**F**)+$0.3802sp^{5.67}d^{0.11}$(**As**) [1.935]
**F_7-As_{15}** $0.9344sp^{4.22}$(**F**)+$0.3563sp^{2.50}d^{1.19}$(**As**) [1.893]
**F_8-B_{13}** $0.9421sp^{1.62}$(**F**)+$0.3354sp^{3.64}$(**B**) [1.967]
**F_8-As_{14}** $0.9718sp^{3.82}$ (**F**)+$0.2359sp^{4.41}d^{3.27}$(**As**) [1.907]
**F_9-B_{13}** $0.9220sp^{1.52}$ (**F**)+$0.3872sp^{2.47}$ (**B**) [1.971]
**F_{10}-As_{15}** $0.9346sp^{4.19}$(**F**)+$0.3556sp^{2.53}d^{1.19}$(**As**) [1.894]
**F_{11}-As_{15}** $0.9344sp^{4.22}$ (**F**)+$0.3563sp^{2.50}d^{1.19}$(**As**) [1.893]
**F_{12}-B_{13}** $0.9429sp^{1.35}$ (**F**)+$0.3330sp^{3.65}$ (**B**) [1.968]
**As_{14}-F_{16}** $0.3414sp^{2.83}d^{1.76}$(**As**)+$0.9399sp^{4.22}$ (**F**) [1.876]
**As_{14}-F_{17}** $0.3446sp^{2.72}d^{1.43}$(**As**)+$0.9387sp^{3.73}$ (**F**) [1.890]

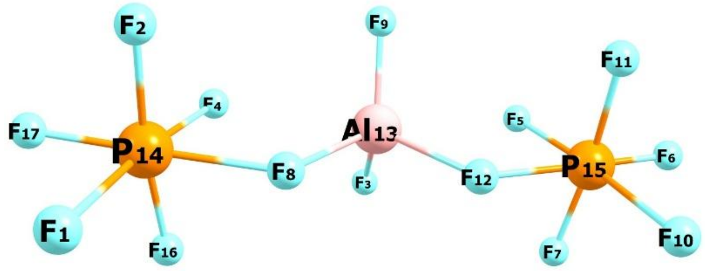

NBO charges	Wiberg indices
**F_1** -0.589	**F_1-P_{14}** 0.549
**F_2** -0.5879	**F_2-P_{14}** 0.550
**F_3** -0.7609	**F_3-Al_{13}** 0.372
**F_4** -0.5909	**F_4-P_{14}** 0.521
**F_5** -0.5909	**F_5-P_{15}** 0.520
**F_6** -0.5919	**F_6-P_{15}** 0.550
**F_7** -0.587	**F_7-P_{15}** 0.550
**F_8** -0.638	**F_8-Al_{13}** 0.201
**F_9** -0.760	**F_8-P_{14}** 0.306
**F_{10}** -0.589	**F_9-Al_{13}** 0.372
**F_{11}** -0.587	**F_{10}-P_{15}** 0.549
**F_{12}** -0.638	**F_{11}-P_{15}** 0.550
**Al_{13}** 2.222	**F_{12}-Al_{13}** 0.201
**P_{14}** 2.731	**F_{12}-P_{15}** 0.306
**P_{15}** 2.731	**P_{14}-F_{16}** 0.550
**F_{16}** -0.587	**P_{14}-F_{17}** 0.550
**F_{17}** -0.591	

Natural bond orbital (BD, 2c-2e): composition (coefficients), hybridization, [occupancy (e)]

F_1-P_{14} $0.9328sp^{2.97}$ (**F**)+$0.3605sp^{2.83}d^{1.74}$(**P**) [1.898]
F_2-P_{14} $0.9331sp^{3.04}$ (**F**)+$0.3595sp^{2.84}d^{1.81}$(**P**) [1.895]
F_3-Al_{13} $0.9635sp^{1.78}$(**F**)+$0.2676sp^{2.26}$(**Al**) [1.969]
F_4-P_{14} $0.9362sp^{3.15}$(**F**)+$0.3516sp^{2.97}d^{1.99}$(**P**) [1.893]
F_5-P_{15} $0.9362sp^{3.15}$(**F**)+$0.3514sp^{2.97}d^{1.99}$(**P**) [1.893]
F_6-P_{15} $0.9320sp^{2.74}$(**F**)+$0.3624sp^{2.64}d^{1.53}$(**P**) [1.904]
F_7-P_{15} $0.9331sp^{3.04}$(**F**)+$0.3595sp^{2.84}d^{1.81}$(**P**) [1.895]
F_8-Al_{13} $0.9760sp^{1.90}$(**F**)+$0.2178sp^{3.97}$(**Al**) [1.964]
F_8-P_{14} $0.9609sp^{3.03}$(**F**)+$0.2771sp^{4.34}d^{3.43}$(**P**) [1.912]
F_9-Al_{13} $0.9635sp^{1.78}$(**F**)+$0.2676sp^{2.26}$(**Al**) [1.969]
F_{10}-P_{15} $0.9327sp^{2.97}$(**F**)+$0.3605sp^{2.82}d^{1.74}$(**P**) [1.898]
F_{11}-P_{15} $0.9331sp^{3.04}$(**F**)+$0.3595sp^{2.84}d^{1.81}$(**P**) [1.895]
F_{12}-Al_{13} $0.9760sp^{1.90}$(**F**)+$0.2178sp^{3.96}$(**Al**) [1.964]
F_{12}-P_{15} $0.9608sp^{3.04}$(**F**)+$0.2772sp^{4.34}d^{3.43}$(**P**) [1.911]
P_{14}-F_{16} $0.3595sp^{2.84}d^{1.81}$(**P**)+$0.9331sp^{3.04}$(**F**) [1.895]
P_{14}-F_{17} $0.3624sp^{2.64}d^{1.53}$(**P**)+$0.9320sp^{2.74}$(**F**) [1.904]

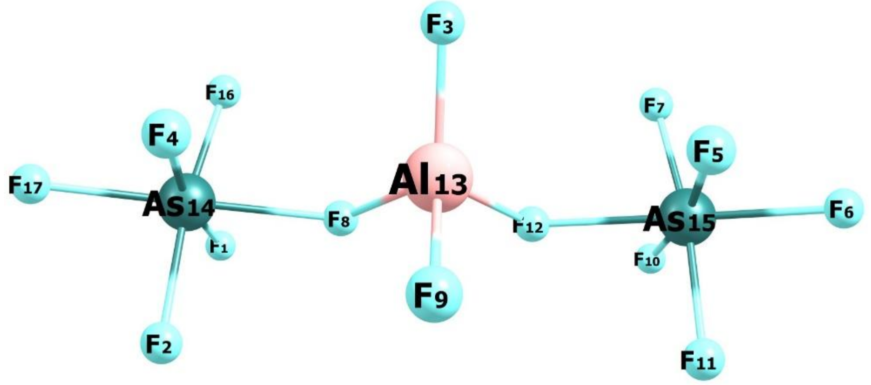

NBO charges	Wiberg indices
F_1 -0.608	F_1-As_{14} 0.510
F_2 -0.607	F_2-As_{14} 0.512
F_3 -0.759	F_3-Al_{13} 0.375
F_4 -0.612	F_4-As_{14} 0.486
F_5 -0.612	F_5-As_{15} 0.486
F_6 -0.613	F_6-As_{15} 0.510
F_7 -0.607	F_7-As_{15} 0.512
F_8 -0.663	F_8-Al_{13} 0.201
F_9 -0.759	F_8-As_{14} 0.270
F_{10} -0.608	F_9-Al_{13} 0.375
F_{11} -0.607	F_{10}-As_{15} 0.510
F_{12} -0.663	F_{11}-As_{15} 0.512
Al_{13} 2.228	F_{12}-Al_{13} 0.201
As_{14} 2.854	F_{12}-As_{15} 0.271
As_{15} 2.854	As_{14}-F_{16} 0.512
F_{16} -0.607	As_{14}-F_{17} 0.511
F_{17} -0.613	

Natural bond orbital (BD, 2c-2e): composition (coefficients), hybridization, [occupancy (e)]

F_1-As_{14} 0.9393$sp^{4.12}$ (**F**)+0.3432$sp^{2.83}d^{1.71}$(**As**) [1.881]
F_2-As_{14} 0.9398$sp^{4.21}$ (**F**)+0.3418$sp^{2.82}d^{1.78}$(**As**) [1.877]
F_3-Al_{13} 0.9632$sp^{1.84}$ (**F**)+0.2686$sp^{2.26}$(**Al**) [1.969]
F_4-As_{14} 0.9431$sp^{4.25}$(**F**)+0.3325$sp^{2.95}d^{1.96}$(**As**) [1.876]
F_5-As_{15} 0.9431$sp^{4.25}$(**F**)+0.3325$sp^{2.95}d^{1.96}$(**As**) [1.876]
F_6-As_{15} 0.9386$sp^{3.76}$(**F**)+0.3451$sp^{2.72}d^{1.51}$(**As**) [1.889]
F_7-As_{15} 0.9398$sp^{4.21}$(**F**)+0.3418$sp^{2.82}d^{1.78}$(**As**) [1.877]
F_8-Al_{13} 0.9760$sp^{1.86}$(**F**)+0.2178$sp^{3.95}$(**Al**) [1.965]
F_8-As_{14} 0.9681$sp^{3.37}$(**F**)+0.2505$sp^{4.34}d^{3.37}$(**As**) [1.909]
F_9-Al_{13} 0.9632$sp^{1.84}$(**F**)+0.2686$sp^{2.26}$(**Al**) [1.969]
F_{10}-As_{15} 0.9393$sp^{4.12}$(**F**)+0.3432$sp^{2.83}d^{1.71}$(**As**) [1.881]
F_{11}-As_{15} 0.9398$sp^{4.21}$(**F**)+0.3418$sp^{2.82}d^{1.78}$(**As**) [1.877]
F_{12}-Al_{13} 0.9760$sp^{1.86}$(**F**)+0.2177$sp^{3.95}$(**Al**) [1.965]
F_{12}-As_{15} 0.9681$sp^{3.37}$(**F**)+0.2507$sp^{4.33}d^{3.37}$(**As**) [1.909]
As_{14}-F_{16} 0.3418$sp^{2.82}d^{1.78}$(**As**)+0.9398$sp^{4.21}$(**F**) [1.877]
As_{14}-F_{17} 0.3451$sp^{2.72}d^{1.51}$(**As**)+0.9386$sp^{3.76}$(**F**) [1.889]

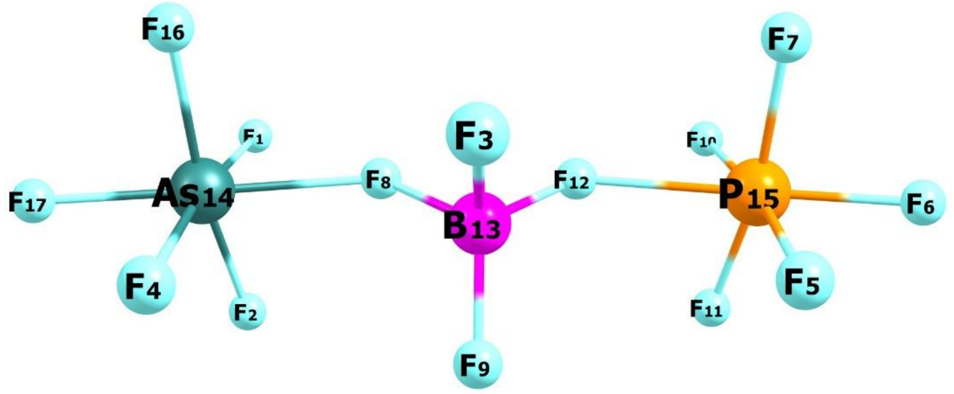

NBO charges	Wiberg indices
F_1 -0.609	F_1-As_{14} 0.507
F_2 -0.607	F_2-As_{14} 0.510
F_3 -0.554	F_3-B_{13} 0.666
F_4 -0.601	F_4-As_{14} 0.510
F_5 -0.578	F_5-P_{15} 0.553
F_6 -0.591	F_6-P_{15} 0.552
F_7 -0.583	F_7-P_{15} 0.555
F_8 -0.546	F_8-B_{13} 0.390
F_9 -0.554	F_8-As_{14} 0.247
F_{10} -0.585	F_9-B_{13} 0.666
F_{11} -0.583	F_{10}-P_{15} 0.552
F_{12} -0.535	F_{11}-P_{15} 0.555
B_{13} 1.550	F_{12}-B_{13} 0.416
As_{14} 2.858	F_{12}-P_{15} 0.245
P_{15} 2.742	As_{14}-F_{16} 0.510
F_{16} -0.607	As_{14}-F_{17} 0.506
F_{17} -0.617	

Natural bond orbital (BD, 2c-2e): composition (coefficients), hybridization, [occupancy (e)]
**F_1-As_{14}** $0.9398sp^{4.17}$ (**F**)+$0.3416sp^{2.85}d^{1.74}$(**As**) [1.879]
**F_2-As_{14}** $0.9401sp^{4.20}$ (**F**)+$0.3410sp^{2.83}d^{1.77}$(**As**) [1.876]
**F_3-B_{13}** $0.9223sp^{1.52}$ (**F**)+$0.3865sp^{2.49}$ (**B**) [1.971]
**F_4-As_{14}** $0.9406sp^{4.20}$ (**F**)+$0.3394sp^{2.85}d^{1.81}$(**As**) [1.874]
**F_5-P_{15}** $0.9338sp^{3.07}$ (**F**)+$0.3578sp^{2.86}d^{1.84}$(**P**) [1.891]
**F_6-P_{15}** $0.9319sp^{2.73}$ (**F**)+$0.3628sp^{2.53}d^{1.43}$(**P**) [1.906]
**F_7-P_{15}** $0.9332sp^{3.06}$(**F**)+$0.3595sp^{2.85}d^{1.80}$(**P**) [1.893]
**F_8-B_{13}** $0.9434sp^{1.62}$(**F**)+$0.3316sp^{3.77}$(**B**) [1.967]
**F_8-As_{14}** $0.9707sp^{3.70}$ (**F**)+$0.2404sp^{4.35}d^{3.23}$(**As**) [1.906]
**F_9-B_{13}** $0.9223sp^{1.52}$ (**F**)+$0.3865sp^{2.49}$ (**B**) [1.971]
**F_{10}-P_{15}** $0.9330sp^{3.05}$(**F**)+$0.3599sp^{2.86}d^{1.77}$(**P**) [1.895]
**F_{11}-P_{15}** $0.9332sp^{3.06}$(**F**)+$0.3595sp^{2.85}d^{1.80}$(**P**) [1.893]
**F_{12}-B_{13}** $0.9403sp^{1.62}$(**F**)+$0.3403sp^{3.49}$(**B**) [1.967]
**F_{12}-P_{15}** $0.9686sp^{3.71}$(**F**)+$0.2487sp^{4.67}d^{3.50}$(**P**) [1.910]
**As_{14}-F_{16}** $0.3410sp^{2.83}d^{1.77}$(**As**)+$0.9401sp^{4.20}$(**F**) [1.876]
**As_{14}-F_{17}** $0.3438sp^{2.73}d^{1.46}$(**As**)+$0.9390sp^{3.73}$(**F**) [1.890]

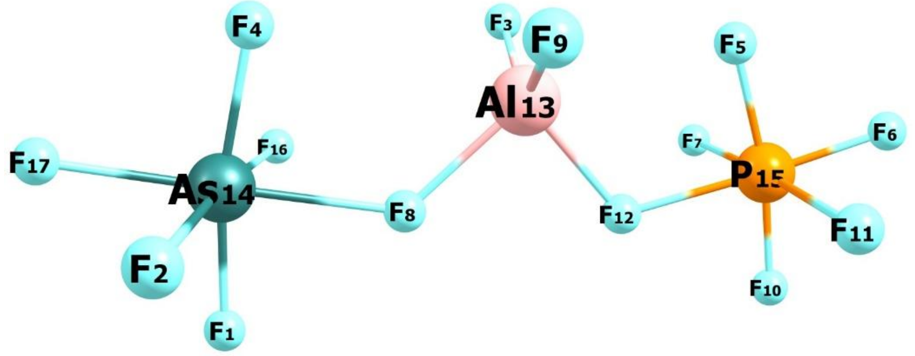

NBO charges	Wiberg indices
F_1 -0.6107	F_1-As_{14} 0.507
F_2 -0.608	F_2-As_{14} 0.510
F_3 -0.759	F_3-Al_{13} 0.373
F_4 -0.611	F_4-As_{14} 0.493
F_5 -0.591	F_5-P_{15} 0.502
F_6 -0.588	F_6-P_{15} 0.554
F_7 -0.585	F_7-P_{15} 0.552
F_8 -0.665	F_8-Al_{13} 0.196
F_9 -0.759	F_8-As_{14} 0.272
F_{10} -0.586	F_9-Al_{13} 0.373
F_{11} -0.585	F_{10}-P_{15} 0.552
F_{12} -0.637	F_{11}-P_{15} 0.552
Al_{13} 2.223	F_{12}-Al_{13} 0.196
As_{14} 2.856	F_{12}-P_{15} 0.314
P_{15} 2.730	As_{14}-F_{16} 0.510
F_{16} -0.608	As_{14}-F_{17} 0.507
F_{17} -0.615	

Natural bond orbital (BD, 2c-2e): composition (coefficients), hybridization, [occupancy (e)]
**F_1-As_{14}** 0.9396$sp^{4.13}$ (**F**)+0.3422$sp^{2.84}d^{1.73}$(**As**) [1.880]
**F_2-As_{14}** 0.9400$sp^{4.19}$(**F**)+0.3413$sp^{2.82}d^{1.78}$(**As**) [1.877]
**F_3-Al_{13}** 0.9634$sp^{1.78}$(**F**)+0.2682$sp^{2.23}$ (**Al**) [1.969]
**F_4-As_{14}** 0.9423$sp^{4.22}$ (**F**)+0.3347$sp^{2.91}d^{1.92}$(**As**) [1.876]
**F_5-P_{15}** 0.9380$sp^{3.21}$(**F**)+0.3468$sp^{3.06}d^{2.11}$(**P**) [1.893]
**F_6-P_{15}** 0.9317$sp^{2.74}$ (**F**)+0.3632$sp^{2.61}d^{1.54}$(**P**) [1.904]
**F_7-P_{15}** 0.9330$sp^{3.03}$(**F**)+0.3600$sp^{2.84}d^{1.82}$(**P**) [1.895]
**F_8-Al_{13}** 0.9767$sp^{1.72}$(**F**)+0.2147$sp^{4.49}$(**Al**) [1.964]
**F_8-As_{14}** 0.9679$sp^{3.17}$(**F**)+0.2512$sp^{4.35}d^{3.31}$(**As**) [1.910]
**F_9-Al_{13}** 0.9634$sp^{1.78}$(**F**)+0.2682$sp^{2.23}$(**Al**) [1.969]
**F_{10}-P_{15}** 0.9324$sp^{2.94}$(**F**)+0.3614$sp^{2.81}d^{1.72}$(**P**) [1.899]
**F_{11}-P_{15}** 0.9330$sp^{3.03}$(**F**)+0.3600$sp^{2.84}d^{1.82}$(**P**) [1.895]
**F_{12}-Al_{13}** 0.9762$sp^{2.04}$(**F**)+0.2170$sp^{3.68}$(**Al**) [1.964]
**F_{12}-P_{15}** 0.9597$sp^{3.21}$(**F**)+0.2809$sp^{4.24}d^{3.28}$(**P**) [1.909]
**As_{14}-F_{16}** 0.3413$sp^{2.82}d^{1.78}$(**As**)+0.9400$sp^{4.19}$(**F**) [1.877]
**As_{14}-F_{17}** 0.3437$sp^{2.73}d^{1.53}$(**As**)+0.9391$sp^{3.76}$(**F**) [1.888]

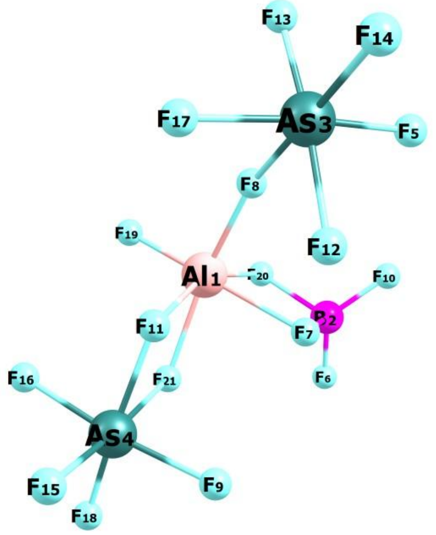

NBO charges	Wiberg indices
**Al$_1$** 2.184	**Al$_1$-F$_7$** 0.165
**B$_2$** 1.526	**Al$_1$-F$_8$** 0.206
**As$_3$** 2.860	**Al$_1$-F$_{11}$** 0.176
**As$_4$** 2.852	**Al$_1$-F$_{19}$** 0.360
**F$_5$** -0.607	**Al$_1$-F$_{20}$** 0.177
**F$_6$** -0.557	**Al$_1$-F$_{21}$** 0.148
**F$_7$** -0.590	**B$_2$-F$_6$** 0.659
**F$_8$** -0.668	**B$_2$-F$_7$** 0.446
**F$_9$** -0.593	**B$_2$-F$_{10}$** 0.669
**F$_{10}$** -0.551	**B$_2$-F$_{20}$** 0.418
**F$_{11}$** -0.627	**As$_3$-F$_5$** 0.510
**F$_{12}$** -0.609	**As$_3$-F$_8$** 0.258
**F$_{13}$** -0.610	**As$_3$-F$_{12}$** 0.504
**F$_{14}$** -0.616	**As$_3$-F$_{13}$** 0.507
**F$_{15}$** -0.598	**As$_3$-F$_{14}$** 0.506
**F$_{16}$** -0.592	**As$_3$-F$_{17}$** 0.506
**F$_{17}$** -0.607	**As$_4$-F$_9$** 0.525
**F$_{18}$** -0.602	**As$_4$-F$_{11}$** 0.336
**F$_{19}$** -0.764	**As$_4$-F$_{15}$** 0.526
**F$_{20}$** -0.603	**As$_4$-F$_{16}$** 0.526
**F$_{21}$** -0.625	**As$_4$-F$_{18}$** 0.522
	**As$_4$-F$_{21}$** 0.360

Natural bond orbital (BD, 2c-2e): composition (coefficients), hybridization, [occupancy (e)]
**Al$_1$-F$_7$** 0.1739sp$^{3.11}$d$^{1.86}$(**Al**)+0.9848sp$^{3.16}$(**F**) [1.934]
**Al$_1$-F$_8$** 0.2144sp$^{7.06}$d$^{0.15}$(**Al**)+0.9768sp$^{1.09}$ (**F**) [1.962]
**Al$_1$-F$_{11}$** 0.1916sp$^{2.51}$d$^{0.95}$(**Al**)+0.9815sp$^{1.85}$ (**F**) [1.948]
**Al$_1$-F$_{19}$** 0.2469sp$^{1.76}$d$^{0.69}$(**Al**)+0.9690sp$^{1.35}$(**F**) [1.952]
**Al$_1$-F$_{20}$** 0.1843sp$^{2.34}$d$^{1.09}$(**Al**)+0.9829sp$^{2.66}$ (**F**) [1.941]
**B$_2$-F$_6$** 0.3910sp$^{2.54}$(**B**)+0.9204sp$^{1.63}$(**F**) [1.971]
**B$_2$-F$_7$** 0.3506sp$^{3.42}$ (**B**)+0.9365sp$^{2.38}$(**F**) [1.964]
**B$_2$-F$_{10}$** 0.3925sp$^{2.50}$ (**B**)+0.9197sp$^{1.62}$(**F**) [1.971]
**B$_2$-F$_{20}$** 0.3416sp$^{3.69}$(**B**)+0.9399sp$^{2.54}$(**F**) [1.964]

As_3-F_5 0.3420$sp^{2.82}d^{1.74}$(**As**)+0.9397$sp^{4.19}$(**F**) [1.878]
As_3-F_8 0.2442$sp^{4.58}d^{3.27}$(**As**)+0.9697$sp^{2.71}$(**F**) [1.913]
As_3-F_{12} 0.3383$sp^{2.86}d^{1.83}$(**As**)+0.9411$sp^{4.20}$(**F**) [1.875]
As_3-F_{13} 0.3419$sp^{2.83}d^{1.74}$(**As**)+0.9398$sp^{4.17}$(**F**) [1.879]
As_3-F_{14} 0.3424$sp^{2.71}d^{1.52}$(**As**)+0.9396$sp^{3.75}$(**F**) [1.887]
As_3-F_{17} 0.3395$sp^{2.85}d^{1.80}$(**As**)+0.9406$sp^{4.21}$(**F**) [1.876]
As_4-F_9 0.3457$sp^{2.78}d^{1.75}$(**As**)+0.9384$sp^{4.26}$ (**F**) [1.873]
As_4-F_{11} 0.2792$sp^{3.72}d^{2.93}$(**As**)+0.9602$sp^{4.52}$(**F**) [1.892]
As_4-F_{15} 0.3489$sp^{2.72}d^{1.58}$(**As**)+0.9372$sp^{3.92}$(**F**) [1.884]
As_4-F_{16} 0.3461$sp^{2.77}d^{1.75}$(**As**)+0.9382$sp^{4.26}$(**F**) [1.873]
As_4-F_{18} 0.3481$sp^{2.73}d^{1.57}$(**As**)+0.9374$sp^{3.87}$(**F**) [1.886]
As_4-F_{21} 0.2890$sp^{3.59}d^{2.78}$(**As**)+0.9573$sp^{4.44}$(**F**) [1.888]

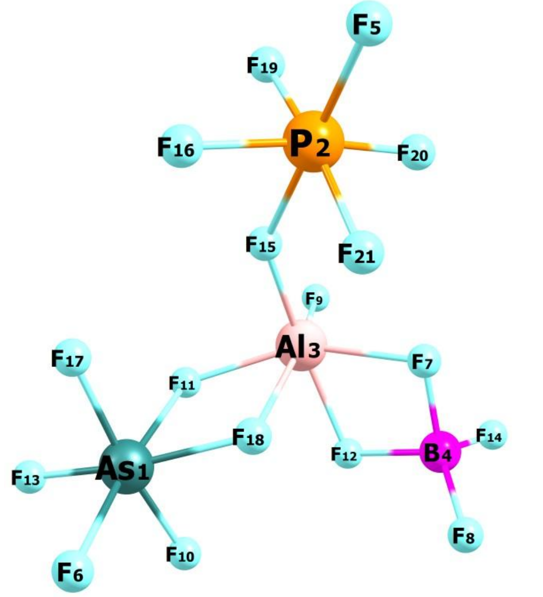

NBO charges	Wiberg indices
**As_1** 2.	**As_1-F_6** 0.526
**P_2** 2.739	**As_1-F_{10}** 0.519
**Al_3** 2.186	**As_1-F_{11}** 0.333
**B_4** 1.527	**As_1-F_{13}** 0.520
**F_5** -0.594	**As_1-F_{17}** 0.530
**F_6** -0.599	**As_1-F_{18}** 0.367
**F_7** -0.598	**P_2-F_5** 0.547
**F_8** -0.555	**P_2-F_{15}** 0.281
**F_9** -0.766	**P_2-F_{16}** 0.552
**F_{10}** -0.598	**P_2-F_{19}** 0.548
**F_{11}** -0.633	**P_2-F_{20}** 0.544
**F_{12}** -0.594	**P_2-F_{21}** 0.544
**F_{13}** -0.602	**Al_3-F_7** 0.183
**F_{14}** -0.555	**Al_3-F_9** 0.356
**F_{15}** -0.648	**Al_3-F_{11}** 0.170
**F_{16}** -0.586	**Al_3-F_{12}** 0.159
**F_{17}** -0.589	**Al_3-F_{15}** 0.213
**F_{18}** -0.620	**Al_3-F_{18}** 0.146
**F_{19}** -0.588	**B_4-F_7** 0.419

**F_{20}** -0.588	**B_4-F_8** 0.662
**F_{21}** -0.588	**B_4-F_{12}** 0.447
	**B_4-F_{14}** 0.663

Natural bond orbital (BD, 2c-2e): composition (coefficients), hybridization, [occupancy (e)]

As_1-F_6 0.3494sp$^{2.70}$d$^{1.55}$(**As**)+0.9370sp$^{3.87}$(**F**) [1.886]
As_1-F_{10} 0.3438sp$^{2.80}$d$^{1.79}$(**As**)+0.9390sp$^{4.24}$(**F**) [1.874]
As_1-F_{11} 0.2776sp$^{3.74}$d$^{2.98}$(**As**)+0.9607sp$^{4.53}$(**F**) [1.893]
As_1-F_{13} 0.3469sp$^{2.75}$d$^{1.62}$(**As**)+0.9379sp$^{3.92}$(**F**) [1.884]
As_1-F_{17} 0.3474sp$^{2.75}$d$^{1.73}$(**As**)+0.9377sp$^{4.27}$(**F**) [1.873]
As_1-F_{18} 0.2922sp$^{3.56}$d$^{2.71}$(**As**)+0.9564sp$^{4.23}$(**F**) [1.887]
P_2-F_5 0.3607sp$^{2.60}$d$^{1.53}$(**P**)+0.9327sp$^{2.75}$(**F**) [1.903]
P_2-F_{15} 0.2653sp$^{4.64}$d$^{3.47}$(**P**)+0.9642sp$^{2.51}$(**F**) [1.916]
P_2-F_{16} 0.3604sp$^{2.83}$d$^{1.76}$(**P**)+0.9328sp$^{3.03}$(**F**) [1.896]
P_2-F_{19} 0.3597sp$^{2.84}$d$^{1.77}$(**P**)+0.9331sp$^{3.03}$(**F**) [1.896]
P_2-F_{20} 0.3572sp$^{2.87}$d$^{1.85}$(**P**)+0.9340sp$^{3.07}$(**F**) [1.893]
P_2-F_{21} 0.3570sp$^{2.87}$d$^{1.85}$(**P**)+0.9341sp$^{3.06}$(**F**) [1.893]
Al_3-F_7 0.1866sp$^{2.62}$d$^{1.26}$(**Al**)+0.9824sp$^{2.59}$(**F**) [1.939]
Al_3-F_9 0.2570sp$^{4.10}$(**Al**)+0.9664sp$^{1.33}$(**F**) [1.964]
Al_3-F_{11} 0.1887sp$^{2.82}$d$^{1.05}$(**Al**)+0.9820sp$^{1.83}$(**F**) [1.950]
Al_3-F_{12} 0.1723sp$^{2.89}$d$^{1.61}$(**Al**)+0.9851sp$^{3.15}$(**F**) [1.936]
Al_3-F_{15} 0.2054sp$^{2.48}$d$^{0.99}$(**Al**)+0.9787sp$^{1.15}$(**F**) [1.952]
B_4-F_7 0.3425sp$^{3.62}$ (**B**)+0.9395sp$^{2.54}$(**F**) [1.963]
B_4-F_8 0.3913sp$^{2.54}$(**B**)+0.9203sp$^{1.62}$(**F**) [1.971]
B_4-F_{12} 0.3506sp$^{3.44}$(**B**)+0.9365sp$^{2.40}$(**F**) [1.964]
B_4-F_{14} 0.3914sp$^{2.53}$(**B**)+0.9202sp$^{1.62}$(**F**) [1.971]

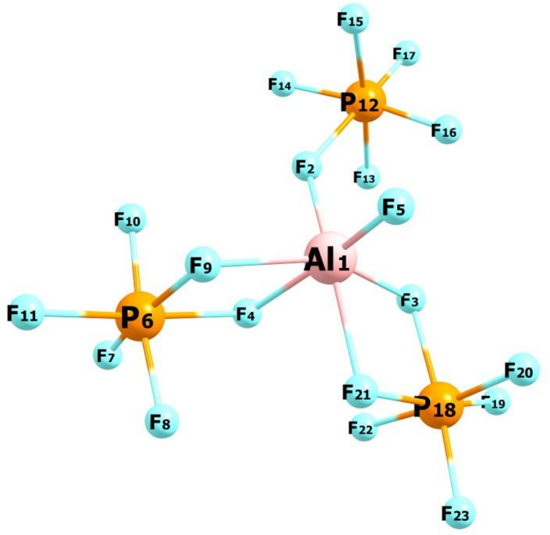

NBO charges	Wiberg indices
**Al_1** 2.187	**Al_1-F_2** 0.211
**F_2** -0.651	**Al_1-F_3** 0.173
**F_3** -0.605	**Al_1-F_5** 0.357
**F_4** -0.598	**Al_1-F_9** 0.166
**F_5** -0.765	**Al_1-F_4** 0.147
**P_6** 2.727	**Al_1-F_{21}** 0.149
**F_7** -0.578	**F_2-P_{12}** 0.277

F_8 -0.578	F_4-P_6 0.401
F_9 -0.608	P_6-F_7 0.565
F_{10} -0.573	P_6-F_8 0.557
F_{11} -0.580	P_6-F_9 0.374
P_{12} 2.740	P_6-F_{10} 0.563
F_{13} -0.589	P_6-F_{11} 0.562
F_{14} -0.587	P_{12}-F_{13} 0.545
F_{15} -0.586	P_{12}-F_{14} 0.550
F_{16} -0.584	P_{12}-F_{15} 0.550
F_{17} -0.592	P_{12}-F_{16} 0.544
P_{18} 2.727	P_{12}-F_{17} 0.548
F_{19} -0.578	P_{18}-F_{19} 0.566
F_{20} -0.572	P_{18}-F_3 0.371
F_{21} -0.600	P_{18}-F_{20} 0.564
F_{22} -0.576	P_{18}-F_{21} 0.398
F_{23} -0.580	P_{18}-F_{22} 0.560
	P_{18}-F_{23} 0.563
Natural bond orbital (BD, 2c-2e): composition (coefficients), hybridization, [occupancy (e)]	
Al_1-F_2 0.2180sp$^{5.38}$(**Al**)+0.9760sp$^{1.10}$(**F**) [1.964]	
Al_1-F_3 0.2058sp$^{2.15}$(**Al**)+0.9786sp$^{1.78}$(**F**) [1.960]	
Al_1-F_5 0.2556sp$^{3.37}$(**Al**)+0.9668sp$^{1.25}$(**F**) [1.966]	
Al_1-F_9 0.2020sp$^{2.18}$(**Al**)+0.9794sp$^{1.77}$(**F**) [1.961]	
F_2-P_{12} 0.9646sp$^{2.68}$(**F**)+0.2635sp$^{4.64}$d$^{3.52}$(**P**) [1.915]	
F_4-P_6 0.9488sp$^{3.52}$(**F**)+0.3158sp$^{3.50}$d$^{2.67}$(**P**) [1.896]	
P_6-F_7 0.3348sp$^{2.86}$d$^{2.01}$(**P**)+0.9423sp$^{2.77}$(**F**) [1.863]	
P_6-F_8 0.3608sp$^{2.91}$d$^{1.88}$(**P**)+0.9327sp$^{3.03}$(**F**) [1.892]	
P_6-F_9 0.2897sp$^{3.30}$d$^{2.44}$(**P**)+0.9571sp$^{3.78}$(**F**) [1.882]	
P_6-F_{10} 0.3624sp$^{2.88}$d$^{1.85}$(**P**)+0.9320sp$^{3.03}$(**F**) [1.892]	
P_6-F_{11} 0.3550sp$^{3.16}$d$^{1.54}$(**P**)+0.9349sp$^{2.80}$(**F**) [1.889]	
P_{12}-F_{13} 0.3579sp$^{2.87}$d$^{1.84}$(**P**)+0.9338sp$^{3.07}$(**F**) [1.894]	
P_{12}-F_{14} 0.3602sp$^{2.84}$d$^{1.77}$(**P**)+0.9329sp$^{3.03}$(**F**) [1.896]	
P_{12}-F_{15} 0.3597sp$^{2.83}$d$^{1.78}$(**P**)+0.9331sp$^{3.04}$(**F**) [1.895]	
P_{12}-F_{16} 0.3570sp$^{2.87}$d$^{1.86}$(**P**)+0.9341sp$^{3.07}$(**F**) [1.893]	
P_{12}-F_{17} 0.3614sp$^{2.60}$d$^{1.53}$(**P**)+0.9324sp$^{2.75}$(**F**) [1.904]	
P_{18}-F_{19} 0.3728sp$^{2.40}$d$^{0.95}$(**P**)+0.9279sp$^{2.80}$(**F**) [1.908]	
P_{18}-F_{20} 0.3758sp$^{2.37}$d$^{1.11}$(**P**)+0.9267sp$^{3.03}$(**F**) [1.908]	
P_{18}-F_{21} 0.3271sp$^{2.76}$d$^{1.67}$(**P**)+0.9450sp$^{3.69}$(**F**) [1.908]	
P_{18}-F_{22} 0.3750sp$^{2.40}$d$^{1.13}$(**P**)+0.9270sp$^{3.03}$(**F**) [1.908]	
P_{18}-F_{23} 0.3481sp$^{5.15}$d$^{1.05}$(**P**)+0.9375sp$^{2.77}$(**F**) [1.878]	

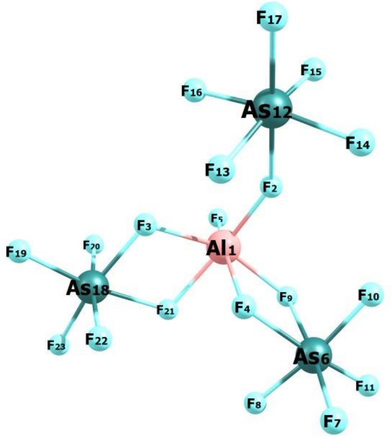

NBO charges	Wiberg indices
**Al$_1$** 2.188	**Al$_1$-F$_2$** 0.207
**F$_2$** -0.674	**Al$_1$-F$_3$** 0.175
**F$_3$** -0.628	**Al$_1$-F$_4$** 0.156
**F$_4$** -0.623	**Al$_1$-F$_5$** 0.356
**F$_5$** -0.766	**Al$_1$-F$_9$** 0.1688
**As$_6$** 2.852	**Al$_1$-F$_{21}$** 0.153
**F$_7$** -0.596	**F$_2$-As$_{12}$** 0.252
**F$_8$** -0.596	**F$_3$-As$_{18}$** 0.333
**F$_9$** -0.633	**F$_4$-As$_6$** 0.356
**F$_{10}$** -0.587	**As$_6$-F$_7$** 0.529
**F$_{11}$** -0.600	**As$_6$-F$_8$** 0.521
**As$_{12}$** 2.862	**As$_6$-F$_9$** 0.333
**F$_{13}$** -0.609	**As$_6$-F$_{10}$** 0.531
**F$_{14}$** -0.605	**As$_6$-F$_{11}$** 0.523
**F$_{15}$** -0.608	**As$_{12}$-F$_{13}$** 0.504
**F$_{16}$** -0.608	**As$_{12}$-F$_{14}$** 0.512
**F$_{17}$** -0.615	**As$_{12}$-F$_{15}$** 0.509
**As$_{18}$** 2.853	**As$_{12}$-F$_{16}$** 0.506
**F$_{19}$** -0.597	**As$_{12}$-F$_{17}$** 0.507
**F$_{20}$** -0.590	**As$_{18}$-F$_{19}$** 0.528
**F$_{21}$** -0.626	**As$_{18}$-F$_{20}$** 0.527
**F$_{22}$** -0.592	**As$_{18}$-F$_{21}$** 0.355
**F$_{23}$** -0.600	**As$_{18}$-F$_{22}$** 0.525
	**As$_{18}$-F$_{23}$** 0.524

Natural bond orbital (BD, 2c-2e): composition (coefficients), hybridization, [occupancy (e)]
**Al$_1$-F$_2$** 0.1938sp$^{2.74}$d$^{1.48}$(**Al**)+0.9810sp$^{1.05}$ (**F**) [1.948]
**Al$_1$-F$_3$** 0.1755sp$^{3.10}$d$^{2.11}$(**Al**)+0.9845sp$^{1.73}$ (**F**) [1.939]
**Al$_1$-F$_4$** 0.1619sp$^{4.23}$d$^{3.26}$(**Al**)+0.9868sp$^{2.01}$ (**F**) [1.936]
**Al$_1$-F$_5$** 0.2372sp$^{1.98}$d$^{0.95}$(**Al**)+0.9715sp$^{1.29}$ (**F**) [1.948]
**Al$_1$-F$_9$** 0.1734sp$^{3.03}$d$^{1.96}$(**Al**)+0.9849sp$^{1.75}$ (**F**) [1.941]
**Al$_1$-F$_{21}$** 0.1624sp$^{3.54}$d$^{2.56}$(**Al**)+0.9867sp$^{1.97}$ (**F**) [1.938]
**F$_2$-As$_{12}$** 0.9704sp$^{2.80}$ (**F**)+0.2415sp$^{4.61}$d$^{3.31}$(**As**) [1.913]
**F$_3$-As$_{18}$** 0.9605sp$^{4.64}$ (**F**)+0.2782sp$^{3.72}$d$^{2.94}$(**As**) [1.891]
**F$_4$-As$_6$** 0.9577sp$^{4.45}$(**F**)+0.2878sp$^{3.61}$d$^{2.78}$(**As**) [1.888]
**As$_6$-F$_7$** 0.3504sp$^{2.70}$d$^{1.54}$(**As**)+0.9366sp$^{3.89}$ (**F**) [1.885]

**As_6-F_8** 0.3443$sp^{2.80}d^{1.78}$(**As**)+0.9389$sp^{4.26}$(**F**) [1.873]
**As_6-F_9** 0.2775$sp^{3.72}d^{2.98}$(**As**)+0.9607$sp^{4.67}$(**F**) [1.892]
**As_6-F_{10}** 0.3478$sp^{2.75}d^{1.73}$(**As**)+0.9376$sp^{4.29}$(**F**) [1.872]
**As_6-F_{11}** 0.3478$sp^{2.74}d^{1.60}$(**As**)+0.9376$sp^{3.91}$(**F**) [1.884]
**As_{12}-F_{13}** 0.3383$sp^{2.86}d^{1.83}$(**As**)+0.9410$sp^{4.21}$(**F**) [1.875]
**As_{12}-F_{14}** 0.3428$sp^{2.82}d^{1.72}$(**As**)+0.9394$sp^{4.20}$(**F**) [1.878]
**As_{12}-F_{15}** 0.3421$sp^{2.83}d^{1.73}$(**As**)+0.9397$sp^{4.18}$(**F**) [1.879]
**As_{12}-F_{16}** 0.3391$sp^{2.85}d^{1.81}$(**As**)+0.9408$sp^{4.22}$(**F**) [1.875]
**As_{12}-F_{17}** 0.3430$sp^{2.71}d^{1.51}$(**As**)+0.9393$sp^{3.75}$(**F**) [1.888]
**As_{18}-F_{19}** 0.3494$sp^{2.72}d^{1.57}$(**As**)+0.9370$sp^{3.92}$(**F**) [1.884]
**As_{18}-F_{20}** 0.3464$sp^{2.76}d^{1.75}$(**As**)+0.9381$sp^{4.27}$(**F**) [1.873]
**As_{18}-F_{21}** 0.2870$sp^{3.60}d^{2.80}$(**As**)+0.9579$sp^{4.55}$(**F**) [1.888]
**As_{18}-F_{22}** 0.3457$sp^{2.78}d^{1.76}$(**As**)+0.9383$sp^{4.27}$(**F**) [1.873]
**As_{18}-F_{23}** 0.3487$sp^{2.73}d^{1.57}$(**As**)+0.9372$sp^{3.87}$(**F**) [1.885]

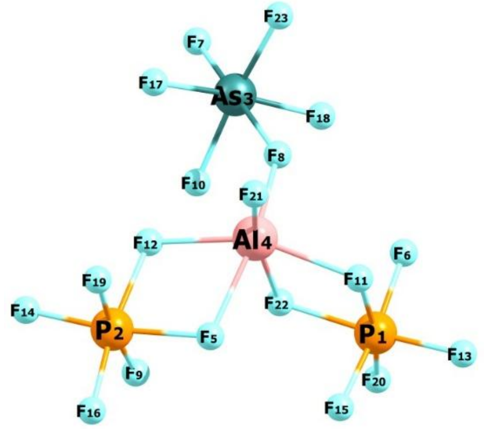

NBO charges	Wiberg indices
**P_1** 2.726	**P_1-F_6** 0.567
**P_2** 2.727	**P_1-F_{11}** 0.372
**As_3** 2.861	**P_1-F_{13}** 0.563
**Al_4** 2.186	**P_1-F_{15}** 0.557
**F_5** -0.600	**P_1-F_{20}** 0.568
**F_6** -0.571	**P_1-F_{22}** 0.396
**F_7** -0.616	**P_2-F_5** 0.396
**F_8** -0.671	**P_2-F_9** 0.562
**F_9** -0.575	**P_2-F_{12}** 0.370
**F_{10}** -0.608	**P_2-F_{14}** 0.567
**F_{11}** -0.608	**P_2-F_{16}** 0.563
**F_{12}** -0.604	**P_2-F_{19}** 0.564
**F_{13}** -0.580	**As_3-F_7** 0.507
**F_{14}** -0.580	**As_3-F_8** 0.252
**F_{15}** -0.580	**As_3-F_{10}** 0.504
**F_{16}** -0.580	**As_3-F_{17}** 0.506
**F_{17}** -0.609	**As_3-F_{18}** 0.512
**F_{18}** -0.606	**As_3-F_{23}** 0.509
**F_{19}** -0.573	**Al_4-F_5** 0.151
**F_{20}** -0.576	**Al_4-F_8** 0.207
**F_{21}** -0.766	**Al_4-F_{11}** 0.168

**F_{22}** -0.597<br>**F_{23}** -0.609	**Al_4-F_{12}** 0.175<br>**Al_4-F_{21}** 0.356<br>**Al_4-F_{22}** 0.156

Natural bond orbital (BD, 2c-2e): composition (coefficients), hybridization, [occupancy (e)]
**P_1-F_6** $0.3639sp^{2.79}d^{1.77}$(**P**)+$0.9314sp^{3.04}$(**F**) [1.893]<br>**P_1-F_{11}** $0.3047sp^{3.74}d^{2.94}$(**P**)+$0.9524sp^{3.80}$(**F**) [1.899]<br>**P_1-F_{13}** $0.3645sp^{2.70}d^{1.61}$(**P**)+$0.9312sp^{2.80}$(**F**) [1.902]<br>**P_1-F_{15}** $0.3615sp^{2.84}d^{1.82}$(**P**)+$0.9324sp^{3.04}$(**F**) [1.893]<br>**P_1-F_{20}** $0.3663sp^{2.65}d^{1.55}$(**P**)+$0.9305sp^{2.77}$(**F**) [1.903]<br>**P_1-F_{22}** $0.3145sp^{3.60}d^{2.74}$(**P**)+$0.9493sp^{3.58}$(**F**) [1.897]<br>**P_2-F_5** $0.3141sp^{3.60}d^{2.76}$(**P**)+$0.9494sp^{3.64}$(**F**) [1.896]<br>**P_2-F_9** $0.3626sp^{2.82}d^{1.80}$(**P**)+$0.9319sp^{3.03}$(**F**) [1.893]<br>**P_2-F_{12}** $0.3046sp^{3.74}d^{2.92}$(**P**)+$0.9525sp^{3.79}$(**F**) [1.898]<br>**P_2-F_{14}** $0.3656sp^{2.68}d^{1.58}$(**P**)+$0.9308sp^{2.80}$(**F**) [1.902]<br>**P_2-F_{16}** $0.3652sp^{2.68}d^{1.57}$(**P**)+$0.9309sp^{2.77}$(**F**) [1.903]<br>**P_2-F_{19}** $0.3630sp^{2.81}d^{1.79}$(**P**)+$0.9318sp^{3.04}$(**F**) [1.893]<br>**As_3-F_7** $0.3429sp^{2.71}d^{1.51}$(**As**)+$0.9394sp^{3.75}$(**F**) [1.888]<br>**As_3-F_8** $0.2417sp^{4.61}d^{3.30}$(**As**)+$0.9704sp^{2.80}$(**F**) [1.913]<br>**As_3-F_{10}** $0.3384sp^{2.86}d^{1.82}$(**As**)+$0.9410sp^{4.21}$(**F**) [1.875]<br>**As_3-F_{17}** $0.3392sp^{2.85}d^{1.81}$(**As**)+$0.9407sp^{4.22}$(**F**) [1.876]<br>**As_3-F_{18}** $0.3428sp^{2.82}d^{1.73}$(**As**)+$0.9394sp^{4.20}$(**F**) [1.878]<br>**As_3-F_{23}** $0.3420sp^{2.83}d^{1.73}$(**As**)+$0.9397sp^{4.18}$(**F**) [1.879]<br>**Al_4-F_5** $0.1614sp^{3.57}d^{2.54}$(**Al**)+$0.9869sp^{2.05}$(**F**) [1.937]<br>**Al_4-F_8** $0.1934sp^{2.74}d^{1.54}$(**Al**)+$0.9811sp^{1.04}$(**F**) [1.948]<br>**Al_4-F_{11}** $0.1726sp^{2.92}d^{1.86}$(**Al**)+$0.9850sp^{1.81}$(**F**) [1.940]<br>**Al_4-F_{12}** $0.1749sp^{3.01}d^{2.04}$(**Al**)+$0.9846sp^{1.79}$(**F**) [1.938]<br>**Al_4-F_{21}** $0.2367sp^{1.96}d^{0.92}$(**Al**)+$0.9716sp^{1.27}$(**F**) [1.948]<br>**Al_4-F_{22}** $0.1612sp^{4.12}d^{3.05}$(**Al**)+$0.9869sp^{2.10}$(**F**) [1.934]

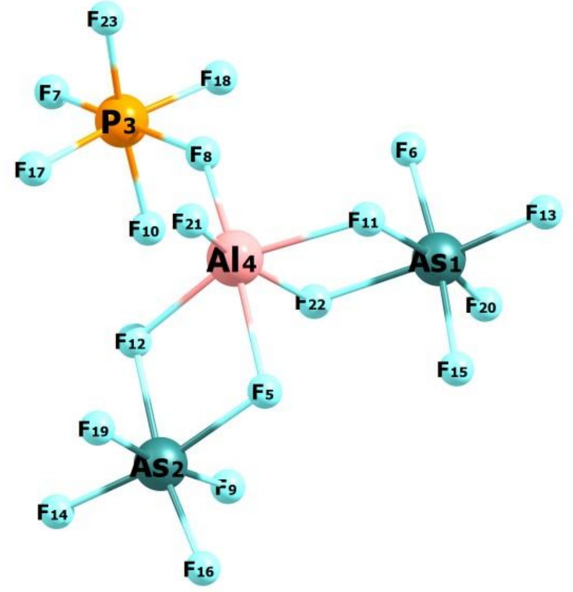

NBO charges	Wiberg indices
**As_1** 2.852<br>**As_2** 2.853<br>**P_3** 2.740	**As_1-F_6** 0.531<br>**As_1-F_{11}** 0.334<br>**As_1-F_{13}** 0.523

**Al$_4$** 2.187	**As$_1$-F$_{15}$** 0.520
**F$_5$** -0.625	**As$_1$-F$_{20}$** 0.528
**F$_6$** -0.587	**As$_1$-F$_{22}$** 0.357
**F$_7$** -0.592	**As$_2$-F$_5$** 0.358
**F$_8$** -0.655	**As$_2$-F$_9$** 0.524
**F$_9$** -0.593	**As$_2$-F$_{12}$** 0.334
**F$_{10}$** -0.587	**As$_2$-F$_{14}$** 0.527
**F$_{11}$** -0.633	**As$_2$-F$_{16}$** 0.524
**F$_{12}$** -0.629	**As$_2$-F$_{19}$** 0.527
**F$_{13}$** -0.600	**P$_3$-F$_7$** 0.549
**F$_{14}$** -0.597	**P$_3$-F$_8$** 0.275
**F$_{15}$** -0.596	**P$_3$-F$_{10}$** 0.543
**F$_{16}$** -0.600	**P$_3$-F$_{17}$** 0.545
**F$_{17}$** -0.587	**P$_3$-F$_{18}$** 0.553
**F$_{18}$** -0.585	**P$_3$-F$_{23}$** 0.549
**F$_{19}$** -0.591	**Al$_4$-F$_5$** 0.150
**F$_{20}$** -0.597	**Al$_4$-F$_8$** 0.212
**F$_{21}$** -0.767	**Al$_4$-F$_{11}$** 0.167
**F$_{22}$** -0.623	**Al$_4$-F$_{12}$** 0.174
**F$_{23}$** -0.587	**Al$_4$-F$_{21}$** 0.354
	**Al$_4$-F$_{22}$** 0.155
Natural bond orbital (BD, 2c-2e): composition (coefficients), hybridization, [occupancy (e)]	
**As$_1$-F$_6$** $0.3477sp^{2.75}d^{1.73}$(**As**)+$0.9376sp^{4.29}$(**F**) [1.872]	
**As$_1$-F$_{11}$** $0.2781sp^{3.71}d^{2.96}$(**As**)+$0.9605sp^{4.67}$(**F**) [1.892]	
**As$_1$-F$_{13}$** $0.3476sp^{2.74}d^{1.61}$(**As**)+$0.9377sp^{3.91}$(**F**) [1.884]	
**As$_1$-F$_{15}$** $0.3442sp^{2.80}d^{1.79}$(**As**)+$0.9389sp^{4.25}$(**F**) [1.873]	
**As$_1$-F$_{20}$** $0.3501sp^{2.70}d^{1.55}$(**As**)+$0.9367sp^{3.88}$(**F**) [1.885]	
**As$_1$-F$_{22}$** $0.2882sp^{3.60}d^{2.77}$(**As**)+$0.9576sp^{4.45}$(**F**) [1.888]	
**As$_2$-F$_5$** $0.2882sp^{3.58}d^{2.78}$(**As**)+$0.9576sp^{4.54}$(**F**) [1.888]	
**As$_2$-F$_9$** $0.3455sp^{2.79}d^{1.76}$(**As**)+$0.9384sp^{4.26}$(**F**) [1.873]	
**As$_2$-F$_{12}$** $0.2783sp^{3.71}d^{2.94}$(**As**)+$0.9605sp^{4.65}$(**F**) [1.891]	
**As$_2$-F$_{14}$** $0.3490sp^{2.73}d^{1.58}$(**As**)+$0.9371sp^{3.92}$(**F**) [1.884]	
**As$_2$-F$_{16}$** $0.3485sp^{2.73}d^{1.57}$(**As**)+$0.9373sp^{3.87}$ (**F**) [1.885]	
**As$_2$-F$_{19}$** $0.3462sp^{2.77}d^{1.75}$(**As**)+$0.9381sp^{4.27}$ (**F**) [1.873]	
**P$_3$-F$_7$** $0.3613sp^{2.59}d^{1.52}$(**P**)+$0.9325sp^{2.75}$(**F**) [1.904]	
**P$_3$-F$_8$** $0.2624sp^{4.68}d^{3.52}$(**P**)+$0.9649sp^{2.58}$(**F**) [1.916]	
**P$_3$-F$_{10}$** $0.3568sp^{2.87}d^{1.85}$(**P**)+$0.9342sp^{3.07}$(**F**) [1.893]	
**P$_3$-F$_{17}$** $0.3575sp^{2.87}d^{1.84}$(**P**)+$0.9339sp^{3.07}$ (**F**) [1.893]	
**P$_3$-F$_{18}$** $0.3604sp^{2.83}d^{1.76}$(**P**)+$0.9328sp^{3.04}$(**F**) [1.895]	
**P$_3$-F$_{23}$** $0.3599sp^{2.84}d^{1.77}$(**P**)+$0.9330sp^{3.03}$(**F**) [1.896]	
**Al$_4$-F$_5$** $0.1609sp^{3.59}d^{2.60}$(**Al**)+$0.9870sp^{1.97}$(**F**) [1.939]	
**Al$_4$-F$_8$** $0.1960sp^{2.66}d^{1.43}$(**Al**)+$0.9806sp^{1.09}$(**F**) [1.948]	
**Al$_4$-F$_{11}$** $0.1723sp^{3.05}d^{1.98}$(**Al**)+$0.9850sp^{1.74}$(**F**) [1.941]	
**Al$_4$-F$_{12}$** $0.1752sp^{3.09}d^{2.09}$(**Al**)+$0.9845sp^{1.71}$(**F**) [1.939]	
**Al$_4$-F$_{21}$** $0.2363sp^{1.98}d^{0.95}$(**Al**)+$0.9717sp^{1.27}$(**F**) [1.948]	
**Al$_4$-F$_{22}$** $0.1612sp^{4.24}d^{3.25}$(**Al**)+$0.9869sp^{2.00}$(**F**) [1.936]	

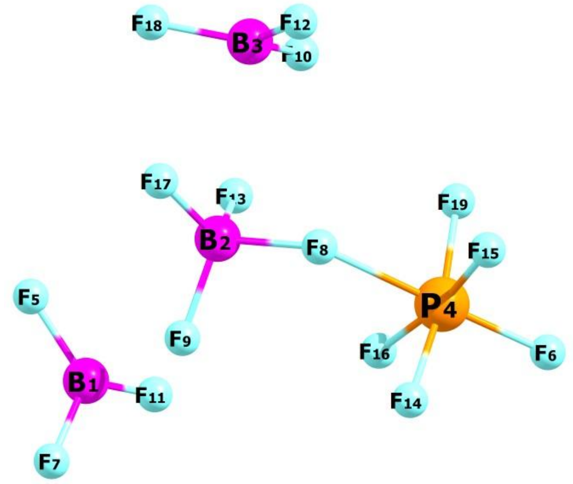

NBO charges	Wiberg indices
**B_1** 1.573	**B_1-F_5** 0.679
**B_2** 1.550	**B_1-F_7** 0.676
**B_3** 1.575	**B_1-F_{11}** 0.690
**P_4** 2.738	**B_1-F_9** 0.103
**F_5** -0.546	**B_2-F_8** 0.393
**F_6** -0.594	**B_2-F_9** 0.548
**F_7** -0.551	**B_2-F_{13}** 0.562
**F_8** -0.535	**B_2-F_{17}** 0.642
**F_9** -0.564	**B_3-F_{10}** 0.685
**F_{10}** -0.545	**B_3-F_{12}** 0.688
**F_{11}** -0.542	**B_3-F_{13}** 0.089
**F_{12}** -0.541	**B_3-F_{18}** 0.683
**F_{13}** -0.565	**P_4-F_6** 0.547
**F_{14}** -0.588	**P_4-F_8** 0.267
**F_{15}** -0.589	**P_4-F_{14}** 0.547
**F_{16}** -0.579	**P_4-F_{15}** 0.546
**F_{17}** -0.568	**P_4-F_{16}** 0.553
**F_{18}** -0.546	**P_4-F_{19}** 0.553
**F_{19}** -0.584	

Natural bond orbital (BD, 2c-2e): composition (coefficients), hybridization, [occupancy (e)]
**B_1-F_5** 0.3863sp$^{2.33}$ (**B**)+0.9224sp$^{1.75}$ (**F**) [1.970]
**B_1-F_7** 0.3859sp$^{2.00}$ (**B**)+0.9226sp$^{1.74}$ (**F**) [1.972]
**B_1-F_{11}** 0.3875sp$^{1.98}$ (**B**)+0.9219sp$^{1.74}$ (**F**) [1.972]
**B_2-F_8** 0.2819sp$^{2.99}$d$^{0.53}$(**B**)+0.9594sp$^{1.60}$ (**F**) [1.909]
**B_2-F_9** 0.3639sp$^{3.04}$ (**B**)+0.9314sp$^{1.36}$(**F**) [1.962]
**B_2-F_{13}** 0.3510sp$^{2.19}$ (**B**)+0.9364sp$^{1.75}$ (**F**) [1.935]
**B_2-F_{17}** 0.3860sp$^{2.56}$ (**B**)+0.9225sp$^{1.55}$ (**F**) [1.971]
**B_3-F_{10}** 0.3863sp$^{2.34}$ (**B**)+0.9224sp$^{1.75}$ (**F**) [1.970]
**B_3-F_{12}** 0.3878sp$^{1.97}$ (**B**)+0.9217sp$^{1.76}$ (**F**) [1.972]
**B_3-F_{18}** 0.3862sp$^{2.00}$ (**B**)+0.9224sp$^{1.76}$ (**F**) [1.972]
**P_4-F_6** 0.3615sp$^{2.57}$d$^{1.49}$(**P**)+0.9324sp$^{2.74}$ (**F**) [1.905]
**P_4-F_8** 0.2600sp$^{4.53}$d$^{3.36}$(**P**)+0.9656sp$^{3.26}$ (**F**) [1.911]
**P_4-F_{14}** 0.3583sp$^{2.87}$d$^{1.82}$(**P**)+0.9336sp$^{3.05}$ (**F**) [1.894]
**P_4-F_{15}** 0.3590sp$^{2.88}$d$^{1.80}$(**P**)+0.9333sp$^{3.04}$ (**F**) [1.896]

P$_4$-F$_{16}$ 0.3582sp$^{2.85}$d$^{1.84}$(**P**)+0.9336sp$^{3.04}$ (**F**) [1.892]
P$_4$-F$_{19}$ 0.3593sp$^{2.85}$d$^{1.80}$(**P**)+0.9332sp$^{3.04}$ (**F**) [1.893]

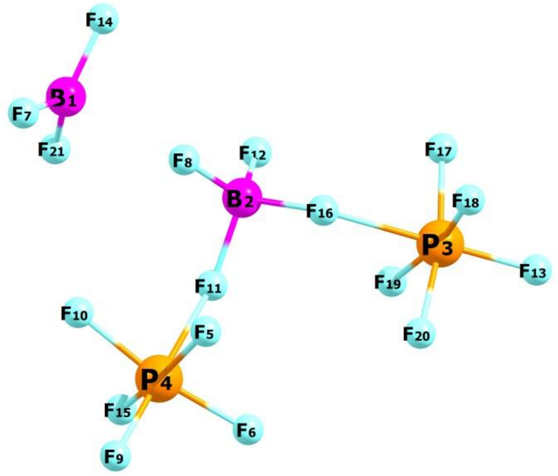

NBO charges	Wiberg indices
**B$_1$** 1.576	**B$_1$-F$_7$** 0.692
**B$_2$** 1.553	**B$_1$-F$_8$** 0.069
**P$_3$** 2.743	**B$_1$-F$_{14}$** 0.688
**P$_4$** 2.744	**B$_1$-F$_{21}$** 0.695
**F$_5$** -0.575	**B$_2$-F$_8$** 0.579
**F$_6$** -0.575	**B$_2$-F$_{11}$** 0.453
**F$_7$** -0.540	**B$_2$-F$_{12}$** 0.657
**F$_8$** -0.564	**B$_2$-F$_{16}$** 0.445
**F$_9$** -0.588	**P$_3$-F$_{13}$** 0.554
**F$_{10}$** -0.578	**P$_3$-F$_{16}$** 0.223
**F$_{11}$** -0.537	**P$_3$-F$_{17}$** 0.557
**F$_{12}$** -0.557	**P$_3$-F$_{18}$** 0.554
**F$_{13}$** -0.589	**P$_3$-F$_{19}$** 0.558
**F$_{14}$** -0.543	**P$_3$-F$_{20}$** 0.563
**F$_{15}$** -0.581	**P$_4$-F$_5$** 0.559
**F$_{16}$** -0.536	**P$_4$-F$_6$** 0.564
**F$_{17}$** -0.580	**P$_4$-F$_9$** 0.556
**F$_{18}$** -0.583	**P$_4$-F$_{10}$** 0.559
**F$_{19}$** -0.575	**P$_4$-F$_{11}$** 0.214
**F$_{20}$** -0.576	**P$_4$-F$_{15}$** 0.556
**F$_{21}$** -0.536	

Natural bond orbital (BD, 2c-2e): composition (coefficients), hybridization, [occupancy (e)]

B$_1$-F$_7$ 0.3890sp$^{2.19}$ (**B**)+0.9213sp$^{1.75}$ (**F**) [1.972]
B$_1$-F$_{14}$ 0.3882sp$^{2.00}$ (**B**)+0.9216sp$^{1.77}$ (**F**) [1.972]
B$_1$-F$_{21}$ 0.3898sp$^{1.96}$ (**B**)+0.9209sp$^{1.77}$ (**F**) [1.972]
B$_2$-F$_8$ 0.3767sp$^{2.74}$ (**B**)+0.9263sp$^{1.65}$ (**F**) [1.970]
B$_2$-F$_{11}$ 0.3479sp$^{3.39}$ (**B**)+0.9375sp$^{1.47}$ (**F**) [1.968]
B$_2$-F$_{12}$ 0.3874sp$^{2.51}$ (**B**)+0.9219sp$^{1.51}$ (**F**) [1.971]
B$_2$-F$_{16}$ 0.3470sp$^{3.45}$ (**B**)+0.9379sp$^{1.50}$ (**F**) [1.968]
P$_3$-F$_{13}$ 0.3634sp$^{2.50}$d$^{1.39}$(**P**)+0.9316sp$^{2.73}$ (**F**) [1.906]
P$_3$-F$_{16}$ 0.2367sp$^{4.90}$d$^{3.64}$(**P**)+0.9716sp$^{3.81}$ (**F**) [1.915]
P$_3$-F$_{17}$ 0.3592sp$^{2.85}$d$^{1.80}$(**P**)+0.9332sp$^{3.09}$ (**F**) [1.892]

**P_3-F_{18}** 0.3601$sp^{2.86}d^{1.76}$(**P**)+0.9329$sp^{3.08}$ (**F**) [1.895]
**P_3-F_{19}** 0.3587$sp^{2.85}d^{1.81}$(**P**)+0.9334$sp^{3.08}$ (**F**) [1.891]
**P_3-F_{20}** 0.3616$sp^{2.82}d^{1.74}$(**P**)+0.9323$sp^{3.08}$ (**F**) [1.893]
**P_4-F_5** 0.3588$sp^{2.85}d^{1.80}$(**P**)+0.9334$sp^{3.10}$ (**F**) [1.891]
**P_4-F_6** 0.3617$sp^{2.82}d^{1.73}$(**P**)+0.9323$sp^{3.09}$ (**F**) [1.893]
**P_4-F_9** 0.3637$sp^{2.48}d^{1.37}$(**P**)+0.9315$sp^{2.74}$ (**F**) [1.906]
**P_4-F_{10}** 0.3596$sp^{2.84}d^{1.78}$(**P**)+0.9331$sp^{3.10}$ (**F**) [1.892]
**P_4-F_{11}** 0.2316$sp^{5.02}d^{3.70}$(**P**)+0.9728$sp^{3.89}$ (**F**) [1.916]
**P_4-F_{15}** 0.3606$sp^{2.85}d^{1.75}$(**P**)+0.9327$sp^{3.09}$ (**F**) [1.895]

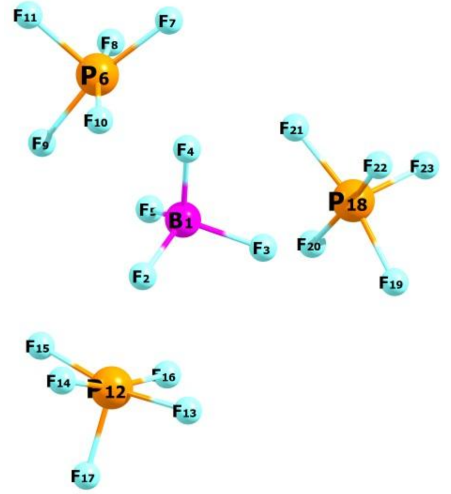

NBO charges	Wiberg indices
**B_1** 1.556	**B_1-F_2** 0.480
**F_2** -0.538	**B_1-F_3** 0.480
**F_3** -0.538	**B_1-F_4** 0.480
**F_4** -0.538	**B_1-F_5** 0.675
**F_5** -0.546	**P_6-F_7** 0.563
**P_6** 2.748	**P_6-F_8** 0.567
**F_7** -0.576	**P_6-F_9** 0.564
**F_8** -0.573	**P_6-F_{10}** 0.562
**F_9** -0.569	**P_6-F_{11}** 0.561
**F_{10}** -0.576	**P_6-F_4** 0.187
**F_{11}** -0.585	**P_{12}-F_{13}** 0.562
**P_{12}** 2.748	**P_{12}-F_{14}** 0.563
**F_{13}** -0.576	**P_{12}-F_{15}** 0.567
**F_{14}** -0.576	**P_{12}-F_{16}** 0.564
**F_{15}** -0.573	**P_{12}-F_{17}** 0.561
**F_{16}** -0.569	**P_{12}-F_2** 0.187
**F_{17}** -0.585	**P_{18}-F_{19}** 0.563
**P_{18}** 2.748	**P_{18}-F_{20}** 0.567
**F_{19}** -0.576	**P_{18}-F_{21}** 0.564
**F_{20}** -0.573	**P_{18}-F_{22}** 0.562
**F_{21}** -0.569	**P_{18}-F_{23}** 0.562
**F_{22}** -0.576	**P_{18}-F_3** 0.187
**F_{23}** -0.585	

Natural bond orbital (BD, 2c-2e): composition (coefficients), hybridization, [occupancy (e)]

B_1-F_2 $0.3538sp^{3.22}$ (**B**)+$0.9353sp^{2.23}$ (**F**) [1.962]

B_1-F_3 $0.3538sp^{3.22}$ (**B**)+$0.9353sp^{2.23}$ (**F**) [1.962]

B_1-F_4 $0.3538sp^{3.22}$ (**B**)+$0.9353sp^{2.23}$ (**F**) [1.962]

B_1-F_5 $0.3860sp^{2.40}$ (**B**)+$0.9225sp^{1.42}$ (**F**) [1.971]

P_6-F_7 $0.3736sp^{2.59}d^{1.22}$(**P**)+$0.9276sp^{3.11}$ (**F**) [1.909]

P_6-F_8 $0.3745sp^{2.57}d^{1.21}$(**P**)+$0.9272sp^{3.12}$ (**F**) [1.908]

P_6-F_9 $0.3745sp^{2.57}d^{1.21}$(**P**)+$0.9272sp^{3.14}$ (**F**) [1.908]

P_6-F_{10} $0.3733sp^{2.59}d^{1.23}$(**P**)+$0.9277sp^{3.13}$ (**F**) [1.908]

P_6-F_{11} $0.3978sp^{4.87}d^{0.17}$(**P**)+$0.9175sp^{2.73}$ (**F**) [1.948]

P_{12}-F_{13} $0.3733sp^{2.59}d^{1.23}$(**P**)+$0.9277sp^{3.13}$ (**F**) [1.908]

P_{12}-F_{14} $0.3736sp^{2.59}d^{1.22}$(**P**)+$0.9276sp^{3.11}$ (**F**) [1.909]

P_{12}-F_{15} $0.3745sp^{2.57}d^{1.21}$(**P**)+$0.9272sp^{3.12}$ (**F**) [1.908]

P_{12}-F_{16} $0.3745sp^{2.57}d^{1.21}$(**P**)+$0.9272sp^{3.14}$ (**F**) [1.908]

P_{12}-F_{17} $0.3978sp^{4.87}d^{0.17}$(**P**)+$0.9175sp^{2.73}$ (**F**) [1.948]

P_{18}-F_{19} $0.3736sp^{2.59}d^{1.22}$(**P**)+$0.9276sp^{3.11}$ (**F**) [1.909]

P_{18}-F_{20} $0.3745sp^{2.57}d^{1.21}$(**P**)+$0.9272sp^{3.12}$ (**F**) [1.908]

P_{18}-F_{21} $0.3745sp^{2.57}d^{1.21}$(**P**)+$0.9272sp^{3.14}$ (**F**) [1.908]

P_{18}-F_{22} $0.3733sp^{2.59}d^{1.23}$(**P**)+$0.9277sp^{3.13}$ (**F**) [1.908]

P_{18}-F_{23} $0.3978sp^{4.87}d^{0.17}$(**P**)+$0.9175sp^{2.73}$ (**F**) [1.948]

Table S4. Gibbs free energies (ΔG_r^{298} in kcal/mol; for T=298.15 K) and electronic energies (ΔE in kcal/mol) predicted for the fragmentation processes of $(X_nF_{(3n+1)})^-$, $(Y_nF_{(5n+1)})^-$ and $(X_nY_{n'}F_{\{(3n+5n')+1\}})^-$ ($n+n'$=1-4 and X=B and/or Al, Y=P and/or As) anions obtained at MP2/aug-cc-pVDZ level.

Formula	Fragmentation path	ΔG_r^{298}	ΔE
$(X_nF_{(3n+1)})^-$ and $(Y_nF_{(5n+1)})^-$ (n=1–4; X=B, Al and Y=P, As)	$BF_4^- \rightarrow BF_3 + F^-$	71.24	80.51
	$AlF_4^- \rightarrow AlF_3 + F^-$	102.36	111.79
	$PF_6^- \rightarrow PF_5 + F^-$	84.32	95.25
	$AsF_6^- \rightarrow AsF_5 + F^-$	94.71	105.61
	$B_2F_7^- \rightarrow BF_4^- + BF_3$	14.04	23.43
	$B_3F_{10}^- \rightarrow B_2F_7^- + BF_3$	2.95	13.46
	$B_4F_{13}^- \rightarrow B_3F_{10}^- + BF_3$	0.50	9.89
	$Al_2F_7^- \rightarrow AlF_4^- + AlF_3$	44.67	51.21
	$Al_3F_{10}^- \rightarrow Al_2F_7^- + AlF_3$	24.38	42.29
	$Al_4F_{13}^- \rightarrow Al_3F_{10}^- + AlF_3$	41.45	53.24
	$P_2F_{11}^- \rightarrow PF_6^- + PF_5$	6.87	17.45
	$P_3F_{16}^- \rightarrow P_2F_{11}^- + PF_5$	-2.04	8.71
	$P_4F_{21}^- \rightarrow P_3F_{16}^- + PF_5$	-2.94	8.05
	$As_2F_{11}^- \rightarrow AsF_6^- + AsF_5$	16.27	26.67
	$As_3F_{16}^- \rightarrow As_2F_{11}^- + AsF_5$	6.19	16.73
	$As_4F_{21}^- \rightarrow As_3F_{16}^- + AsF_5$	-0.04	13.72
XYF_9^- (X=B and/or Al; Y=P and/or As)	$BPF_9^- \rightarrow BF_4^- + PF_5$	18.62	29.11
	$BPF_9^- \rightarrow PF_6^- + BF_3$	5.55	14.37
	$BAsF_9^- \rightarrow BF_4^- + AsF_5$	28.71	39.05
	$BAsF_9^- \rightarrow AsF_6^- + BF_3$	5.24	13.95
	$AlPF_9^- \rightarrow AlF_4^- + PF_5$	16.24	26.74
	$AlPF_9^- \rightarrow PF_6^- + AlF_3$	34.28	43.28
	$AlAsF_9^- \rightarrow AlF_4^- + AsF_5$	25.79	36.11

	$AlAsF_9^- \rightarrow AsF_6^- + AlF_3$	33.43	42.29
**$(X_nY_{n'}F_{\{(3n+5n')+1\}})^-$ ($n+n'$=3 and X=B and/or Al, Y=P and/or As)**	$B_2PF_{12}^- \rightarrow BPF_9^- + BF_3$	0.58	11.18
	$B_2PF_{12}^- \rightarrow B_2F_7^- + PF_5$	5.16	16.87
	$B_2AsF_{12}^- \rightarrow BAsF_9^- + BF_3$	0.13	10.59
	$B_2AsF_{12}^- \rightarrow B_2F_7^- + AsF_5$	14.80	26.21
	$Al_2PF_{12}^- \rightarrow AlPF_9^- + AlF_3$	31.68	42.63
	$Al_2PF_{12}^- \rightarrow Al_2F_7^- + PF_5$	3.25	18.17
	$Al_2AsF_{12}^- \rightarrow AlAsF_9^- + AlF_3$	26.68	41.80
	$Al_2AsF_{12}^- \rightarrow Al_2F_7^- + AsF_5$	7.79	26.71
	$BAlPF_{12}^- \rightarrow AlPF_9^- + BF_3$	4.83	15.81
	$BAlPF_{12}^- \rightarrow BPF_9^- + AlF_3$	33.56	44.72
	$BAlPF_{12}^- \rightarrow AlF_4^- + PF_5 + BF_3$	21.07	42.55
	$BAlAsF_{12}^- \rightarrow AlAsF_9^- + BF_3$	3.24	15.55
	$BAlAsF_{12}^- \rightarrow BAsF_9^- + AlF_3$	31.43	43.89
	$BAlAsF_{12}^- \rightarrow AlF_4^- + AsF_5 + BF_3$	29.03	51.66
	$BP_2F_{14}^- \rightarrow P_2F_{11}^- + BF_3$	0.06	10.71
	$BP_2F_{14}^- \rightarrow BPF_9^- + PF_5$	1.38	13.80
	$AlP_2F_{14}^- \rightarrow P_2F_{11}^- + AlF_3$	33.93	45.27
	$AlP_2F_{14}^- \rightarrow AlPF_9^- + PF_5$	6.52	19.44
	$BAs_2F_{14}^- \rightarrow As_2F_{11}^- + BF_3$	-1.87	9.06
	$BAs_2F_{14}^- \rightarrow BAsF_9^- + AsF_5$	9.16	21.79
	$AlAs_2F_{14}^- \rightarrow As_2F_{11}^- + AlF_3$	31.88	43.30
	$AlAs_2F_{14}^- \rightarrow AlAsF_9^- + AsF_5$	14.72	27.68
	$BPAsF_{14}^- \rightarrow BPF_9^- + AsF_5$	10.43	21.69
	$BPAsF_{14}^- \rightarrow BAsF_9^- + PF_5$	0.34	11.76
	$BPAsF_{14}^- \rightarrow AsF_6^- + PF_5 + BF_3$	5.58	25.71
	$AlPAsF_{14}^- \rightarrow AlPF_9^- + AsF_5$	15.62	27.21
	$AlPAsF_{14}^- \rightarrow AlAsF_9^- + PF_5$	6.07	17.84
	$AlPAsF_{14}^- \rightarrow AsF_6^- + PF_5 + AlF_3$	39.50	60.13
	$AlPAsF_{14}^- \rightarrow AsF_5 + PF_5 + AlF_4^-$	31.86	53.95

$(X_nY_{n'}F_{\{(3n+5n')+1\}})^-$ (n+n'=4 and X=B and/or Al, Y=P and/or As)	$B_3PF_{15}^- \rightarrow BPF_9^- + 2BF_3$	0.78	20.62
	$B_3PF_{15}^- \rightarrow B_2PF_{12}^- + BF_3$	0.21	9.44
	$B_3PF_{15}^- \rightarrow B_3F_{10}^- + PF_5$	2.42	12.85
	$B_3AsF_{15}^- \rightarrow BAsF_9^- + 2BF_3$	-0.04	19.94
	$B_3AsF_{15}^- \rightarrow B_2AsF_{12}^- + BF_3$	-0.16	9.35
	$B_3AsF_{15}^- \rightarrow B_3F_{10}^- + AsF_5$	11.69	22.09
	$Al_3PF_{15}^- \rightarrow AlPF_9^- + 2AlF_3$	64.08	93.07
	$Al_3PF_{15}^- \rightarrow Al_2PF_{12}^- + AlF_3$	32.39	50.43
	$Al_3PF_{15}^- \rightarrow Al_3F_{10}^- + PF_5$	11.26	26.30
	$Al_3AsF_{15}^- \rightarrow AlAsF_9^- + 2AlF_3$	63.60	92.75
	$Al_3AsF_{15}^- \rightarrow Al_2AsF_{12}^- + AlF_3$	36.92	50.94
	$Al_3AsF_{15}^- \rightarrow Al_3F_{10}^- + AsF_5$	20.33	35.35
	$B_2AlPF_{15}^- \rightarrow BAlPF_{12}^- + BF_3$	-2.23	8.99
	$B_2AlPF_{15}^- \rightarrow AlPF_9^- + 2BF_3$	2.60	24.81
	$B_2AlAsF_{15}^- \rightarrow BAlAsF_{12}^- + BF_3$	-0.59	8.44
	$B_2AlAsF_{15}^- \rightarrow AlAsF_9^- + 2BF_3$	2.65	23.98
	$BAl_2PF_{15}^- \rightarrow BAlPF_{12}^- + AlF_3$	26.48	44.43
	$BAl_2PF_{15}^- \rightarrow Al_2PF_{12}^- + BF_3$	-0.38	17.60
	$BAl_2PF_{15}^- \rightarrow Al_2F_7^- + PF_5 + BF_3$	2.87	35.77
	$BAl_2AsF_{15}^- \rightarrow BAlAsF_{12}^- + AlF_3$	27.64	44.42
	$BAl_2AsF_{15}^- \rightarrow Al_2AsF_{12}^- + BF_3$	4.20	18.16
	$BAl_2AsF_{15}^- \rightarrow Al_2F_7^- + AsF_5 + BF_3$	11.99	44.87
	$BP_3F_{19}^- \rightarrow P_3F_{16}^- + BF_3$	0.12	11.18
	$BP_3F_{19}^- \rightarrow BF_4^- + 3PF_5$	18.03	52.10
	$BP_3F_{19}^- \rightarrow BP_2F_{14}^- + PF_5$	-1.98	9.18
	$BP_3F_{19}^- \rightarrow BPF_9^- + 2PF_5$	-0.60	22.98
	$AlP_3F_{19}^- \rightarrow P_3F_{16}^- + AlF_3$	38.87	55.09
	$AlP_3F_{19}^- \rightarrow AlF_4^- + 3PF_5$	25.66	64.73
	$AlP_3F_{19}^- \rightarrow AlP_2F_{14}^- + PF_5$	2.90	18.54
	$AlP_3F_{19}^- \rightarrow AlPF_9^- + 2PF_5$	9.42	37.99

$(X_nY_{n'}F_{\{(3n+5n')+1\}})^-$ (n+n'=4 and X=B and/or Al, Y=P and/or As)	$BAs_3F_{19}^- \rightarrow As_3F_{16}^- + BF_3$	-3.28	7.71
	$BAs_3F_{19}^- \rightarrow BF_4^- + 3AsF_5$	42.65	76.21
	$BAs_3F_{19}^- \rightarrow BAs_2F_{14}^- + AsF_5$	4.78	15.38
	$BAs_3F_{19}^- \rightarrow BAsF_9^- + 2AsF_5$	13.94	37.17
	$AlAs_3F_{19}^- \rightarrow As_3F_{16}^- + AlF_3$	37.00	54.42
	$AlAs_3F_{19}^- \rightarrow AlF_4^- + 3AsF_5$	51.82	91.65
	$AlAs_3F_{19}^- \rightarrow AlAs_2F_{14}^- + AsF_5$	11.31	27.86
	$AlAs_3F_{19}^- \rightarrow AlAsF_9^- + 2AsF_5$	26.03	55.53
	$BP_2AsF_{19}^- \rightarrow BP_2F_{14}^- + AsF_5$	6.31	17.20
	$BP_2AsF_{19}^- \rightarrow BPF_9^- + PF_5 + AsF_5$	7.69	30.99
	$BP_2AsF_{19}^- \rightarrow BAsF_9^- + 2PF_5$	-2.39	21.06
	$BP_2AsF_{19}^- \rightarrow P_2F_{11}^- + BF_3 + AsF_5$	6.37	27.91
	$BP_2AsF_{19}^- \rightarrow BPAsF_{14}^- + PF_5$	-2.74	9.30
	$AlP_2AsF_{19}^- \rightarrow AlP_2F_{14}^- + AsF_5$	11.63	28.00
	$AlP_2AsF_{19}^- \rightarrow AlPF_9^- + PF_5 + AsF_5$	18.15	47.44
	$AlP_2AsF_{19}^- \rightarrow AlAsF_9^- + 2PF_5$	8.60	38.07
	$AlP_2AsF_{19}^- \rightarrow P_2F_{11}^- + AlF_3 + AsF_5$	45.56	73.26
	$AlP_2AsF_{19}^- \rightarrow AlPAsF_{14}^- + PF_5$	2.53	20.24
	$BPAs_2F_{19}^- \rightarrow BAs_2F_{14}^- + PF_5$	-4.30	7.16
	$BPAs_2F_{19}^- \rightarrow BAsF_9^- + PF_5 + AsF_5$	4.87	28.94
	$BPAs_2F_{19}^- \rightarrow BPF_9^- + 2AsF_5$	14.95	38.88
	$BPAs_2F_{19}^- \rightarrow As_2F_{11}^- + BF_3 + PF_5$	-6.17	16.22
	$BPAs_2F_{19}^- \rightarrow BPAsF_{14}^- + AsF_5$	4.52	17.18
	$AlPAs_2F_{19}^- \rightarrow AlAs_2F_{14}^- + PF_5$	2.15	18.64
	$AlPAs_2F_{19}^- \rightarrow AlAsF_9^- + PF_5 + AsF_5$	16.87	46.32
	$AlPAs_2F_{19}^- \rightarrow AlPF_9^- + 2AsF_5$	26.41	55.69
	$AlPAs_2F_{19}^- \rightarrow As_2F_{11}^- + AlF_3 + PF_5$	34.03	61.94
	$AlPAs_2F_{19}^- \rightarrow AlPAsF_{14}^- + AsF_5$	10.79	28.48
	$B_2P_2F_{17}^- \rightarrow B_2PF_{12}^- + PF_5$	-1.33	10.87
	$B_2P_2F_{17}^- \rightarrow BP_2F_{14}^- + BF_3$	-2.14	8.25

$(X_nY_{n'}F_{\{(3n+5n')+1\}})^-$ (n+n'=4 and X=B and/or Al, Y=P and/or As)	$B_2P_2F_{17}^- \rightarrow P_2F_{11}^- + 2BF_3$	-2.07	18.96
	$B_2P_2F_{17}^- \rightarrow BPF_9^- + BF_3 + PF_5$	-0.75	22.05
	$B_2As_2F_{17}^- \rightarrow B_2AsF_{12}^- + AsF_5$	9.69	19.57
	$B_2As_2F_{17}^- \rightarrow BAs_2F_{14}^- + BF_3$	0.65	8.37
	$B_2As_2F_{17}^- \rightarrow As_2F_{11}^- + 2BF_3$	-1.22	17.43
	$B_2As_2F_{17}^- \rightarrow BAsF_9^- + BF_3 + AsF_5$	9.82	30.16
	$Al_2P_2F_{17}^- \rightarrow Al_2PF_{12}^- + PF_5$	2.23	22.00
	$Al_2P_2F_{17}^- \rightarrow AlP_2F_{14}^- + AlF_3$	27.39	45.19
	$Al_2P_2F_{17}^- \rightarrow P_2F_{11}^- + 2AlF_3$	61.32	90.46
	$Al_2P_2F_{17}^- \rightarrow Al_2F_7^- + 2PF_5$	5.48	40.17
	$Al_2P_2F_{17}^- \rightarrow AlPF_9^- + AlF_3 + PF_5$	33.91	64.64
	$Al_2As_2F_{17}^- \rightarrow Al_2AsF_{12}^- + AsF_5$	16.06	31.83
	$Al_2As_2F_{17}^- \rightarrow AlAs_2F_{14}^- + AlF_3$	28.02	45.96
	$Al_2As_2F_{17}^- \rightarrow As_2F_{11}^- + 2AlF_3$	59.90	89.26
	$Al_2As_2F_{17}^- \rightarrow Al_2F_7^- + 2AsF_5$	23.85	58.54
	$Al_2As_2F_{17}^- \rightarrow AlAsF_9^- + AlF_3 + AsF_5$	42.74	73.63
	$B_2PAsF_{17}^- \rightarrow B_2AsF_{12}^- + PF_5$	1.28	10.49
	$B_2PAsF_{17}^- \rightarrow B_2PF_{12}^- + AsF_5$	10.92	19.83
	$B_2PAsF_{17}^- \rightarrow BAsF_9^- + BF_3 + PF_5$	1.41	21.08
	$B_2PAsF_{17}^- \rightarrow BPF_9^- + AsF_5 + BF_3$	11.49	31.01
	$B_2PAsF_{17}^- \rightarrow BPAsF_{14}^- + BF_3$	1.07	9.32
	$Al_2PAsF_{17}^- \rightarrow Al_2AsF_{12}^- + PF_5$	6.88	22.65
	$Al_2PAsF_{17}^- \rightarrow Al_2PF_{12}^- + AsF_5$	11.42	31.19
	$Al_2PAsF_{17}^- \rightarrow AlAsF_9^- + AlF_3 + PF_5$	33.55	64.45
	$Al_2PAsF_{17}^- \rightarrow AlPF_9^- + AsF_5 + AlF_3$	43.10	73.82
	$Al_2PAsF_{17}^- \rightarrow AlPAsF_{14}^- + AlF_3$	27.48	46.61
	$BAlP_2F_{17}^- \rightarrow BAlPF_{12}^- + PF_5$	3.27	19.47
	$BAlP_2F_{17}^- \rightarrow AlPF_9^- + PF_5 + BF_3$	8.10	35.28
	$BAlP_2F_{17}^- \rightarrow AlP_2F_{14}^- + BF_3$	1.58	15.83
	$BAlP_2F_{17}^- \rightarrow BPF_9^- + PF_5 + AlF_3$	36.83	64.19

$(X_nY_{n'}F_{\{(3n+5n')+1\}})^-$ ($n+n'$=4 and X=B and/or Al, Y=P and/or As)	$BAlAs_2F_{17}^- \rightarrow BAlAsF_{12}^- + AsF_5$	13.88	29.07
	$BAlAs_2F_{17}^- \rightarrow AlAsF_9^- + AsF_5 + BF_3$	17.12	44.62
	$BAlAs_2F_{17}^- \rightarrow AlAs_2F_{14}^- + BF_3$	2.40	16.94
	$BAlAs_2F_{17}^- \rightarrow BAsF_9^- + AsF_5 + AlF_3$	45.31	72.96
	$BAlPAsF_{17}^- \rightarrow BAlAsF_{12}^- + PF_5$	4.28	19.83
	$BAlPAsF_{17}^- \rightarrow BAlPF_{12}^- + AsF_5$	12.24	28.93
	$BAlPAsF_{17}^- \rightarrow AlAsF_9^- + PF_5 + BF_3$	7.52	35.37
	$BAlPAsF_{17}^- \rightarrow AlPF_9^- + AsF_5 + BF_3$	17.07	44.74
	$BAlPAsF_{17}^- \rightarrow BAsF_9^- + PF_5 + AlF_3$	35.71	63.72
	$BAlPAsF_{17}^- \rightarrow BPF_9^- + AsF_5 + AlF_3$	45.80	73.65
	$BAlPAsF_{17}^- \rightarrow AlPAsF_{14}^- + BF_3$	1.45	17.54
	$BAlPAsF_{17}^- \rightarrow BPAsF_{14}^- + AlF_3$	35.37	51.96

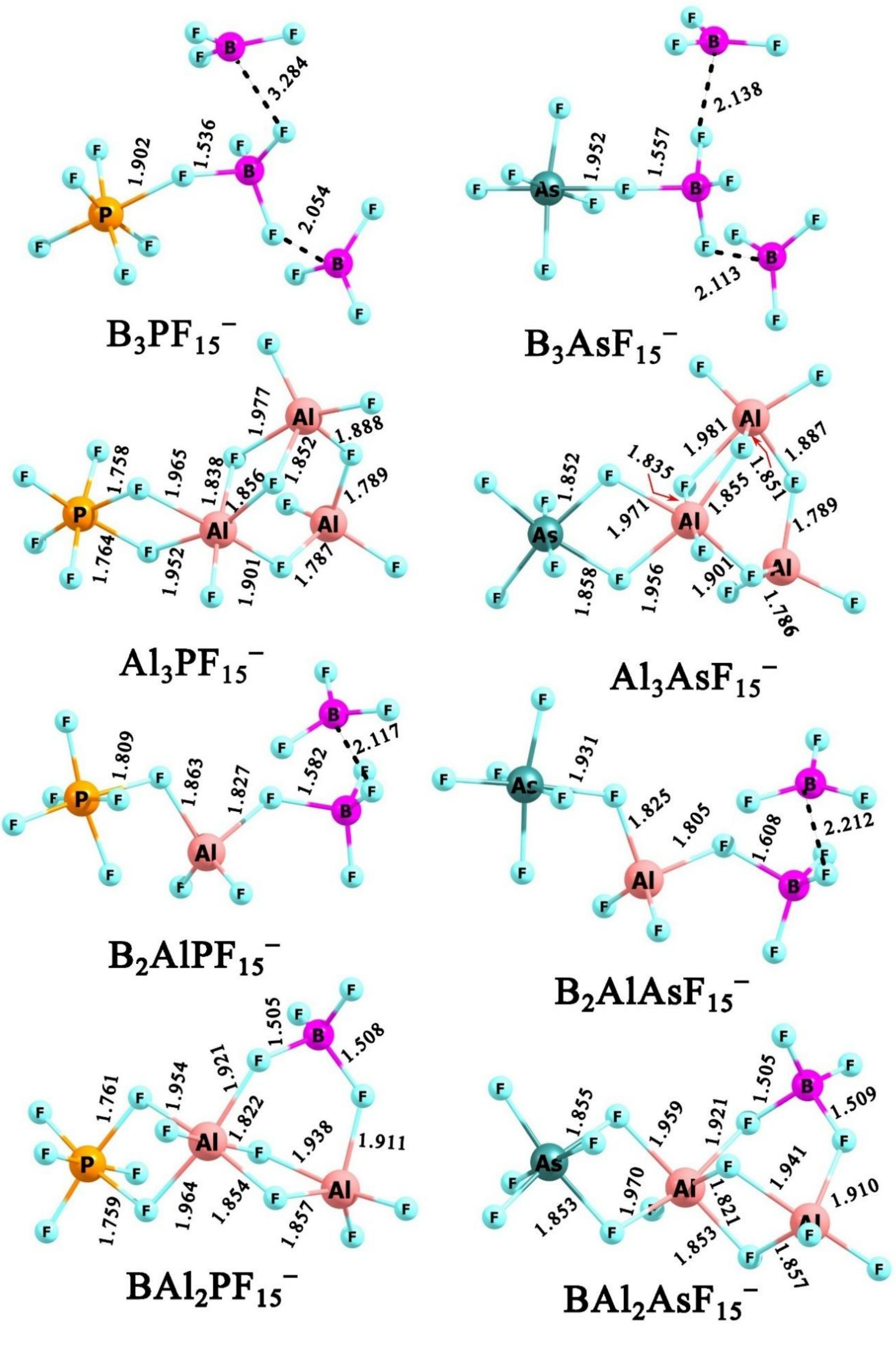

Figure S1. The equilibrium structures of $(\mathbf{X}_3\mathbf{Y}F_{15})^-$ anions (where **X**=B and/or Al, **Y**=P or As) obtained at MP2/aug-cc-pVDZ level.

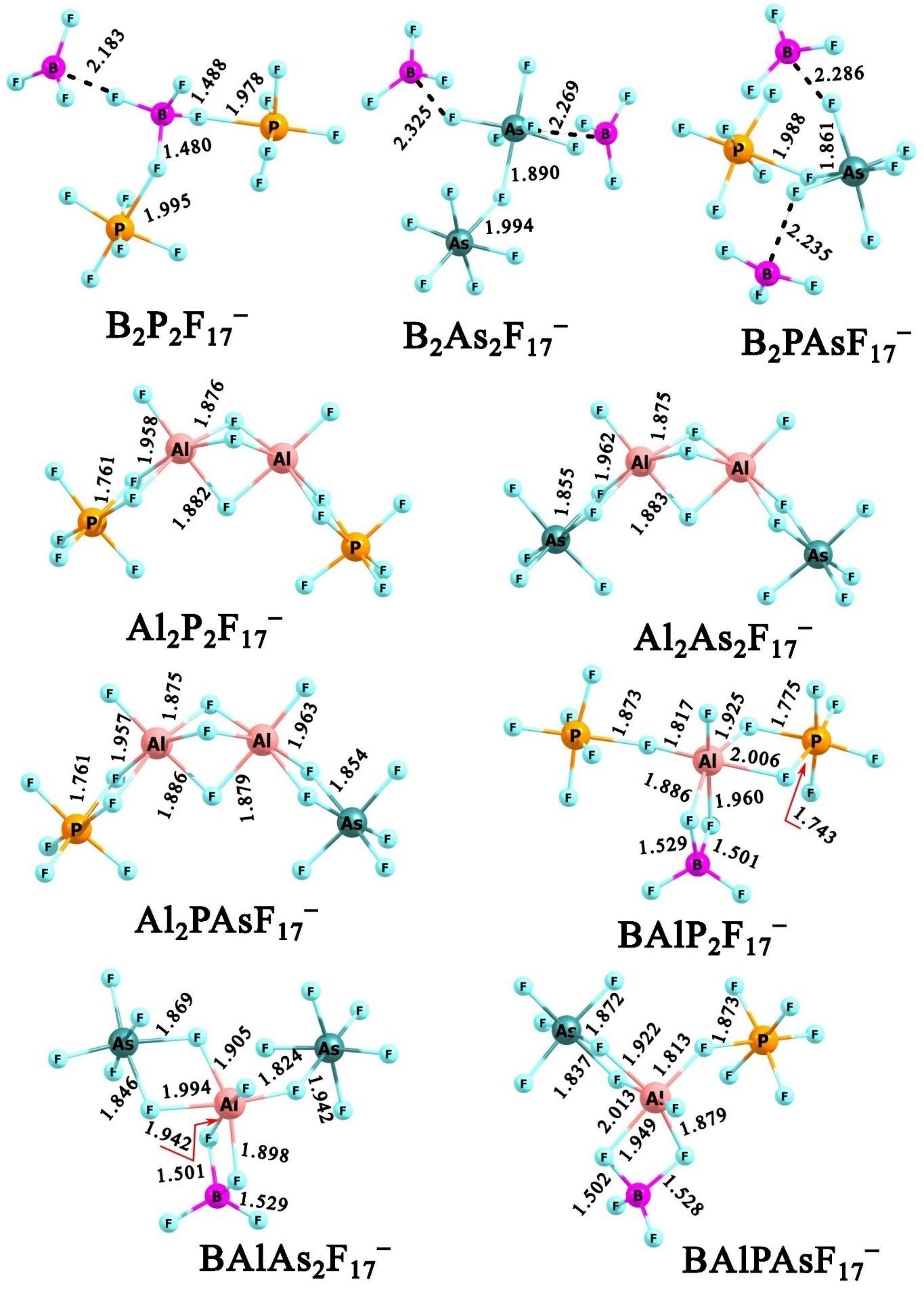

Figure S2. The equilibrium structures of $(\mathbf{X}_2\mathbf{Y}_2F_{17})^-$ anions (where **X**=B and/or Al, **Y**=P and/or As) obtained at MP2/aug-cc-pVDZ level.

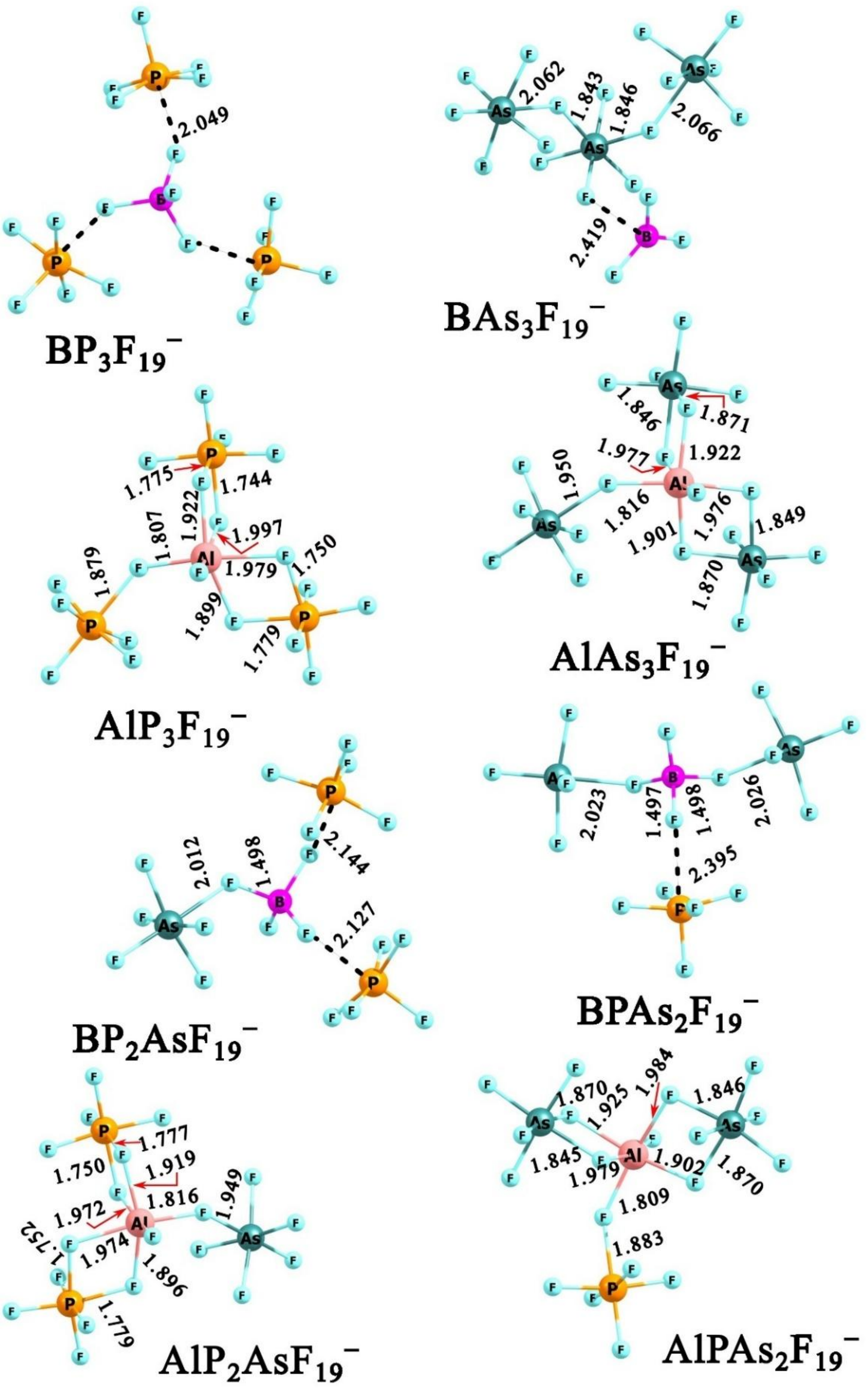

Figure S3. The equilibrium structures of $(\mathbf{XY}_3F_{19})^-$ anions (where **X**=B or Al, **Y**=P and/or As) obtained at MP2/aug-cc-pVDZ level.

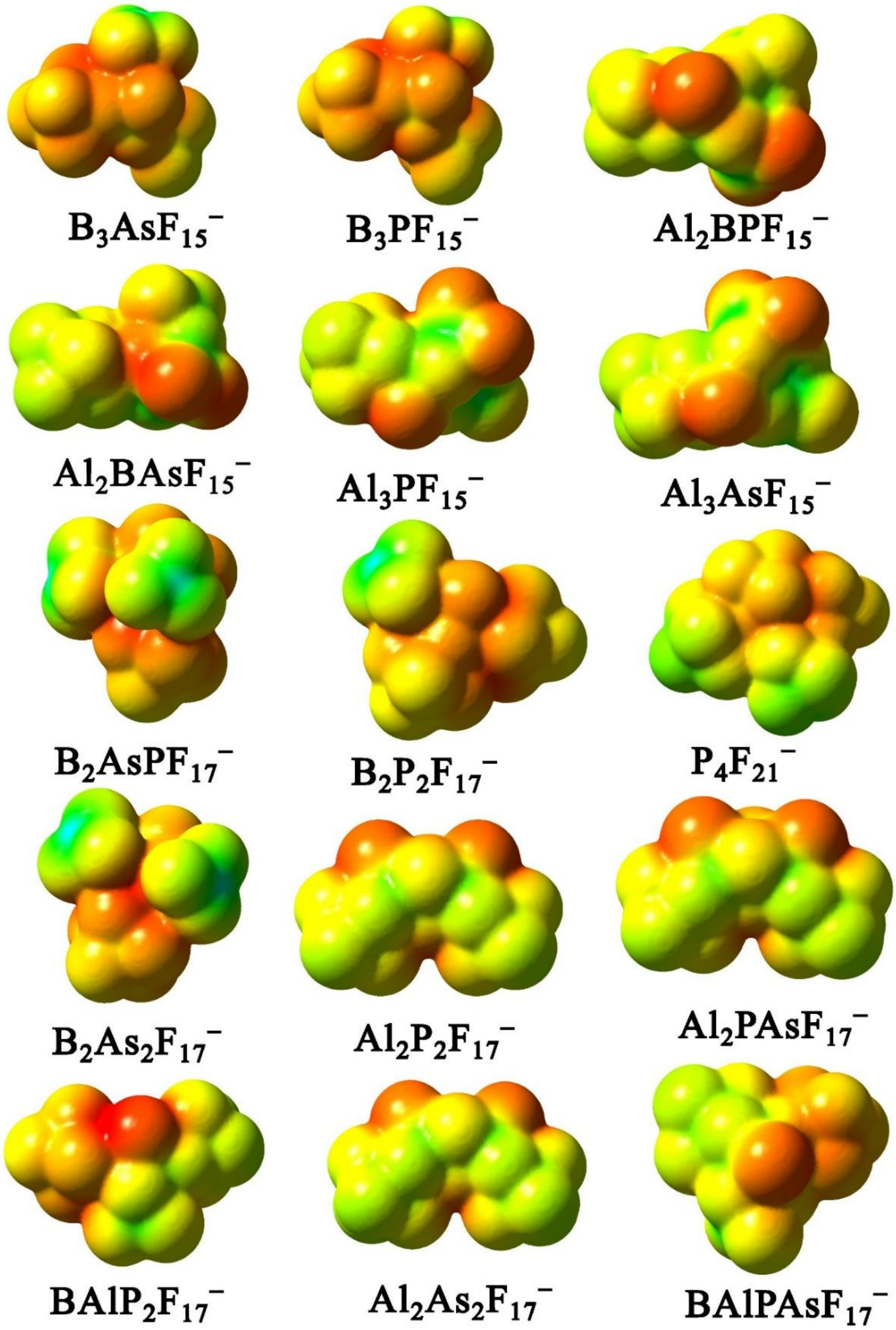

Figure S4. Molecular electrostatic potential (ESP) maps $\mathbf{X}_4F_{13}^-$, $\mathbf{Y}_4F_{21}^-$, and $(\mathbf{X}_n\mathbf{Y}_{n'}F_{\{(3n+5n')+1\}})^-$ anions (where n+n'=4 and **X**=B and/or Al, **Y**=P and/or As) computed from MP2 electron densities and plotted on the 0.001 e/bohr^3 isodensity surface and arranged in order of increasing VDE. Visualization performed on the same scale as in Figure 5 (i.e., from −0.15 to +1 × 10^{-6} a.u.).

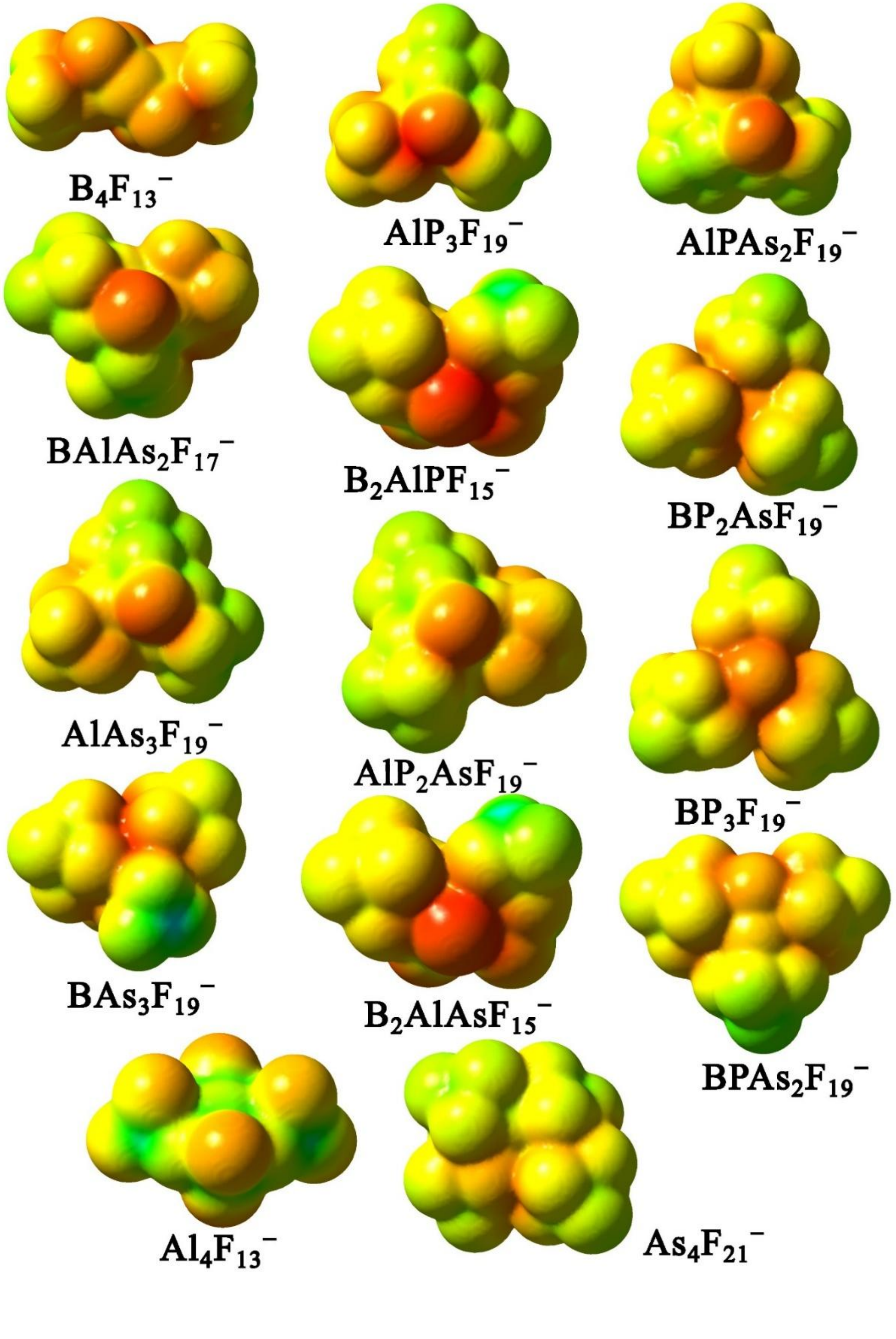

Figure S5. Molecular electrostatic potential (ESP) maps $\mathbf{X}_4F_{13}^-$, $\mathbf{Y}_4F_{21}^-$, and $(\mathbf{X}_n\mathbf{Y}_{n'}F_{\{(3n+5n')+1\}})^-$ anions (where n+n'=4 and **X**=B and/or Al, **Y**=P and/or As) computed from MP2 electron densities and plotted on the 0.001 e/bohr^3 isodensity surface and arranged in order of increasing VDE (continuation). Visualization performed on the same scale as in Figure 5 (i.e., from −0.15 to +1 × 10^{-6} a.u.).
